# Reputation, Co-Regulation, and "Soft Capture": Corporate Political Activity of the Portuguese Food Industry

**DOI:** 10.34172/ijhpm.9495

**Published:** 2026-06-01

**Authors:** Sofia da Costa, Mélissa Mialon, Marta Figueira, Maria João Gregório, Pedro Graça

**Affiliations:** ^1^Epidemiology Research Unit (EPIUnit), Institute of Public Health, University of Porto, Porto, Portugal.; ^2^Faculty of Nutrition and Food Sciences, University of Porto, Porto, Portugal.; ^3^Inserm, Université Paris Cité, Paris, France.; ^4^School of Medicine, Trinity College Dublin, Dublin, Ireland.; ^5^National Healthy Eating Promotion Program, Directorate-General of Health, Lisbon, Portugal.

**Keywords:** Corporate Political Activity, Commercial Determinants of Health, Public Health, Public Policy, Food Industry, Portugal

## Abstract

**Background:**

Corporate political activity (CPA) refers to practices through which commercial actors seek to influence public policy and prioritise their commercial interests. While extensively documented internationally, little empirical evidence exists for Portugal. This study provides the first systematic analysis of the CPA of the food industry in Portugal.

**Methods:**

We conducted a systematic document analysis, following a protocol developed by INFORMAS (International Network for Food and Obesity/Non-Communicable Diseases Research, Monitoring and Action Support), a network that monitors food environments. This was triangulated with semi-structured interviews and illustrated through two policy examples. Twenty-five food industry actors were selected based on market share, trade association membership, and relevance to policy debates. Publicly available materials (January 2022–December 2023) were collected and coded using Ulucanlar’s CPA framework, distinguishing framing and action strategies. Data were triangulated with 18 interviews conducted between July and December 2024 with informants from academia, government, industry, civil society, public health, and the media.

**Results:**

We identified 534 examples of framing and 799 examples of action strategies. Framing was dominated by portrayals of corporations as "good actors" aligned with health, sustainability, and national development. Action strategies focused on reputational management, corporate social responsibility (CSR), and the displacement of public health roles. Legal obstruction and overtly adversarial tactics were absent. Large multinational manufacturers and major retailers accounted for the highest number of coded CPA examples, with trade associations also represented among actors engaging in policy-related activities. Interviewees confirmed these patterns and expressed concerns over informal access to policy-makers, weak state capacity, and reliance on corporate-led initiatives.

**Conclusions:**

The food industry in Portugal primarily relies on reputational and co-regulatory strategies, with limited evidence of overtly confrontational tactics. These findings are consistent with a form of institutionalised influence in which corporate actors engage closely with public authorities and participate in governance processes in ways that may shape the direction and scope of public health policy.

## Background

**Key Messages**

**Implications for policy makers**

- This study shows that commercial actors in the food industry in Portugal operate largely through reputational strategies, co-regulation, and institutional partnerships, creating structural conditions favoring voluntary approaches, limiting independent oversight, and weakening public policy enforcement.
- Limited transparency around lobbying and informal access to policy-makers emerged as a key contextual factor shaping corporate political activity (CPA).
- The frequent use of corporate social responsibility (CSR) initiatives to frame food companies as responsible actors in relation to public health and sustainability points to the need for clearer boundaries around CSR partnerships, particularly in schools, scientific events, and public health campaigns.
- Reliance on industry-led initiatives was most visible in areas where public institutions face resource constraints, underscoring the importance of adequately resourced public institutions that can operate autonomously, without dependence on corporate funding.

**Implications for the public**

The food we eat is shaped not only by personal choices, but also by how powerful companies influence the rules that govern our food system. This study shows that in Portugal, food companies often present themselves as responsible and socially engaged actors in relation to public health, nutrition, the economy, science and sustainability, while at the same time shaping policies in ways that protect their business interests. For example, they sponsor school programmes or nutrition events, but this can blur the line between education and marketing. By highlighting these practices, our research helps explain why some public health measures take so long to be adopted or enforced. Greater transparency, stronger public institutions, and clearer limits on corporate partnerships are needed to ensure that food and nutrition policies truly protect health rather than corporate profits.

Non-communicable diseases (NCDs) are largely preventable, yet they remain the leading cause of morbidity and mortality globally.^1^ They are strongly shaped by patterns of consumption of harmful products, including ultra-processed foods, alcohol, and tobacco.^2^ The drivers of these consumption patterns are conceptualised by the World Health Organization (WHO) as part of the commercial determinants of health (CDoH),^3^ defined as “private sector activities that affect people’s health positively or negatively” through decisions on supply chains, labour conditions, product design and packaging, research funding, lobbying, and other practices.^4^

 Unhealthy commodity industries, such as food, alcohol, and tobacco corporations, have been identified as particularly harmful, since a substantial share of their portfolios comprises products with high profit margins but adverse health impacts.^2^ These industries use coordinated methods to influence public health legislation while defending corporate interests and perpetuating NCDs and other harms on humans and the planet, through plastic pollution, for example.^5^ Focusing on the food sector, its influence extends well beyond consumer choice: through product formulation, marketing strategies, and attempts to shape public policy.^6^ This political activity, known as *corporate political activity* (CPA), refers to “corporate attempts to shape government policy in ways favourable to the firm.”^7^ These mechanisms influence the availability, affordability, and desirability of foods, thereby structuring dietary patterns at the population level.^8^

 International evidence has documented recurring CPA patterns across regions, including common tactics used to influence public health policy.^9-18^ CPA has been recognised as a substantial challenge to NCD prevention efforts.^5^ While these tactics vary in intensity and form, they frequently include framing health problems as matters of individual responsibility, promoting voluntary codes of conduct, sponsoring scientific research, and engaging in corporate social responsibility (CSR) initiatives that symbolically align companies with public interest goals.^5^

 In Portugal, dietary risks are among the leading contributors to morbidity and mortality, with high salt and sugar intake, low fruit and vegetable consumption, and the wide availability of energy-dense, nutrient-poor foods contributing to obesity, diabetes, and cardiovascular disease.^19^ Policy-makers have sought to respond through coordinated nutrition policy. A landmark initiative was the launch of the National Programme for the Promotion of Healthy Eating (PNPAS) in 2012, establishing a long-term agenda for food and nutrition policies, intersectoral action, and public awareness.^20^

 Portugal’s nutrition policies have received consistent international recognition. Monitoring frameworks such as the INFORMAS (International Network for Food and Obesity/Non-Communicable Diseases Research, Monitoring and Action Support) Healthy Food Environment Policy Index and the World Cancer Research Fund International NOURISHING framework have ranked Portugal among the higher-performing European Union countries in areas including food labelling, fiscal measures, marketing restrictions, and public food procurement.^21,22^ The WHO has further described Portugal as a “front-runner” in the European Region.^23^

 Despite these advances, progress in improving diets appears uneven, with some measures facing delays, limited scope, or weak enforcement, as illustrated by the prolonged legislative process and implementation challenges documented for food marketing restrictions in Portugal.^24^ Another particularly salient example is the Food Industry Reformulation Agreement launched in 2019, a voluntary co-regulatory pact between the government and major industry associations aimed at reducing salt, sugar, and trans-fat levels across multiple product categories.^25^ Following negotiations with industry, targets were diluted, and timelines extended compared with the WHO reformulation benchmarks, resulting in less ambitious commitments and a smaller projected health impact; the authors argue that voluntary action is unlikely to be sufficient on its own and should be complemented by mandatory policies.^26^ Evidence from other contexts suggests that industry actors actively shape the design, adoption, and enforcement of nutrition policies, particularly where voluntary agreements and industry-led initiatives often accompany or substitute regulation.^5^

 To date, however, empirical research on the CPA of the food industry in Portugal remains limited. The only related study, conducted in 2019, suggested that the country’s relatively lenient alcohol policies were linked to lobbying by the alcohol sector.^27^

 To address this gap, the present study systematically examines how food industry actors in Portugal deploy CPA framing and action strategies to influence health policy and public discourse. By combining systematic document analysis with semi-structured interviews, we contribute to international efforts to monitor industry influence and promote greater transparency and accountability in food and nutrition policy-making.

## Methods

###  Study Design

 We combined a systematic document analysis with semi-structured interviews to examine the CPA of the food industry in Portugal. The document analysis followed the INFORMAS methodology to guide actor selection, source identification, and data collection.^28^ The analysis of CPA practices was conducted using the framework developed by Ulucanlar et al in 2023.^5^ Semi-structured interviews were used to strengthen validity through triangulation and to capture practices not visible in publicly available information. In addition, we report some of the data through two illustrative examples of CPA being used to shape specific nutrition policies.

###  Definition of the Food Industry

 For this study, the “food industry” included processed food and non-alcoholic beverage manufacturers, fast food companies, food retailers, and national trade associations related to processed food production.^29^

###  Identification of Industry Actors 

 Following the INFORMAS protocol, we first identified relevant industry actors, including the largest processed food manufacturers, the largest non-alcoholic beverages manufacturers, the largest fast food companies, the largest food retailers, in terms of market share, and the major trade associations in the food sector.^29^ Sales volume and market share data for companies were obtained from Market data retrieved from *Euromonitor International*^30^ (Passport database, 2021 edition, the categories “Packaged Food” and “Soft Drinks”), which provided company-level retail value shares.^30^ In addition, all corporate members of the *Federação das Indústrias Portuguesas Agro-Alimentares* (FIPA - Federation of Portuguese Agri-Food Industries), as listed on the organisation’s website in January 2022, were included.^31^ For retailers, we selected the three companies with the largest grocery retail market share in 2021, plus the retailer with the highest year-on-year increase in that year.^32^ This process resulted in 25 industry actors: Associação Portuguesa de Empresas de Distribuição (APED - Portuguese Association of Distribution Companies); Bimbo Donuts Portugal; Bom Petisco; Cerealis; Coca-Cola Portugal; Danone Portugal; Delta Cafés; Ferrero Ibérica; FIPA; Iglo Portugal; Imperial; Jerónimo Martins; Lactogal; Lidl; Mercadona; Nestlé Portugal; Novarroz; Nutrinveste - Sovena; PepsiCo/Matutano; Primor - Charcutaria Prima SA; McDonald’s Portugal; Sonae; Sumol + Compal; Unilever Fima; and Vitacress.

###  Identification of Information Source 

 Phase two of the INFORMAS protocol is the identification of sources of information related to the industry actors selected in phase one.

 First, the official websites of all 25 selected industry actors were systematically reviewed, as these constitute primary sources of corporate communication and disclosure. For each industry actor, the entire official website was systematically reviewed, including all publicly available content such as press releases and corporate disclosures (eg, annual reports and sustainability reports, where available). Given the heterogeneity of corporate websites, no restriction was imposed on specific document types or sections. All publicly available content was considered eligible for inclusion, regardless of whether it appeared in sections such as “About us,” “Sustainability,” “News,” or other webpages. This approach was applied consistently across all companies included in the study.

 Second, a targeted search was conducted within selected governmental, academic, and professional websites to identify content referring to the industry actors included in the study. This step aimed to capture evidence of interactions, partnerships, sponsorships, or policy-related activities not always disclosed on corporate platforms. For this purpose, the name of each of the 25 industry actors was systematically entered into the internal search engines of each selected website, and all relevant pages referring to the actors were reviewed. The following governmental and institutional websites were consulted, as detailed in Supplementary file 1: Directorate-General of Health, PNPAS/Integrated Strategy for the Promotion of Healthy Eating (EIPAS), National Institute of Health, National Parliament, Socialist Party, and Social Democratic Party, Faculty of Nutrition and Food Sciences of the University of Porto (FCNAUP), Faculty of Medicine of the University of Porto, Nova Medical School Lisbon, Institute of Public Health of the University of Porto, School of Biotechnology of the Catholic University of Portugal, Portuguese Association of Nutrition, Portuguese Congress of Nutrition and Food from Portuguese Association of Nutrition, and the Portuguese Order of Nutritionists.

 The first author maintained an archive of source materials, including screenshots for webpages accessed during data collection, to ensure traceability and reproducibility.

###  Data Collection and Analysis

 Phases three and four of the INFORMAS protocol involve the systematic collection and analysis of publicly available information. While the INFORMAS methodology includes a taxonomy for categorising corporate practices, this taxonomy was not used for the analytical phase of the present study. Instead,the CPA framework later developed by Ulucanlar et al was applied.^5^

 This framework was selected because it is based on a comprehensive review of the literature across multiple unhealthy commodity industries, including the one previously applied within INFORMAS-based studies. In addition, it facilitates comparison of findings across countries and across different unhealthy commodity industries.^5^

 Between January and March 2024, the first author (SdC) systematically screened all relevant publicly available materials published between January 2022 and December 2023 across the identified sources. Relevant content was archived through screenshots at the time of collection in order to preserve the material as it appeared during the study period. Coding was conducted directly from these archived materials. Data were analysed using NVivo and organised in Excel. Coding followed the CPA framework developed by Ulucanlar et al,^5^ which distinguishes between (1) framing strategies, describing how corporations shape problem definitions and policy debates, and (2) action strategies, referring to practices that seek to influence policy-makers, the law, evidence, and public support.

 Coding was deductive and iterative. All materials were read in full, and specific excerpts containing references to the taxonomies were coded. Framing strategies and action strategies were coded independently, meaning that the same document could contain multiple coded instances across one or both taxonomies. Where a single text segment reflected more than one CPA strategy, overlapping codes were applied, in line with the framework’s recognition that corporate practices may simultaneously serve multiple strategic purposes.

 For clarity, two types of codes are used throughout the manuscript. Codes in parentheses (eg, F-GA2) refer to the original subcategories of the CPA taxonomy proposed by Ulucanlar et al and are used consistently with the original publication. Codes presented in square brackets (eg, [F.CC10]) are internal identifiers assigned to individual empirical examples and are used exclusively to link the narrative results to the full dataset provided in Supplementary file 2. To enhance transparency, the website from which each example was retrieved is reported for all coded entries in Supplementary file 2. Individual URLs were not systematically archived for each coded text segment; however, all materials were publicly accessible at the time of data collection.

 To ensure reliability, a second author (MF) independently reviewed materials for five actors across both taxonomies; discrepancies were resolved by discussion, though inter-rater agreement was not formally quantified.

###  Semi-structured Interviews

 To complement the document analysis, the first author conducted semi-structured interviews with key informants between July and December 2024. Initial invitations targeted individuals who (*i*) held decision-making or advisory roles in policy, (*ii*) had been publicly involved in academic research in public health, nutrition, and food sciences, or (*iii*) had direct professional or institutional links with food industry actors. We sought representation across six sectors: academia, government, the food industry, civil society organisations, public health institutions, and the media.

 Based on these criteria, 26 invitations were sent initially by the first author in March 2024. A further 10 individuals were identified through snowball sampling when participants suggested additional relevant informants. In total, 18 interviews were conducted, distributed as follows: academia (n = 4), government (n = 4), food industry (n = 6), civil society organisations (n = 2), public health (n = 1), and the media (n = 1). Four participants had overlapping roles across sectors (eg, academia and government or academia and industry).

 All interviews were conducted by the first author, either in person or online (Google Meet), based on the participants’ needs, and lasted approximately 45 minutes. Written informed consent was obtained before participation, and all interviewees agreed for their interviews to be digitally recorded and notes taken. Transcriptions, with pseudonymised information, were generated using an AI tool, Transkriptor, and verified by the first author for accuracy; participants were given the opportunity to review their transcripts.

 The interview guide (See Supplementary file 3) was adapted from previous research,^18^ translated into Portuguese by the first author, and pre-tested with two academic colleagues before data collection to ensure clarity and contextual appropriateness. Due to time constraints, interviews focused primarily on the taxonomy of action strategies, although broader reflections on industry framing strategies also emerged.

###  Illustrative Policy Examples

 In our results section, we report two illustrative policy examples from PNPAS: (*i*) the increase in taxes on sugar-sweetened non-alcoholic beverages (Law No. 42/2016),^33^ and (*ii*) the law imposing restrictions on food advertising to children under 16 years of age (Law No. 30/2019).^34^ These examples emerged inductively, as they were repeatedly referenced in the interviews despite not being explicitly included in the interview guide, underscoring their perceived relevance within the Portuguese policy context.

## Results

 A total of 534 examples of framing strategies and 799 examples of action strategies were identified in the document analysis. Complete datasets are provided in Supplementary file 2, which also includes the categorisation of each entry within the CPA framework. Based on the number of coded CPA examples identified in the document analysis, large global manufacturers (eg, Coca-Cola, Danone, Nestlé) and major retailers (eg, Sonae, Lidl, Jerónimo Martins) accounted for the highest frequency of observed framing and action strategies, while some companies, such as Novarroz and Primor, showed little or no observable activity.

 In addition to the documentary evidence, interviewees referred to a range of CPA-related practices consistent with those identified in the document analysis, while also highlighting additional practices that were not directly prompted by the interview guide. These included spontaneous references to framing, such as the use of economic arguments to justify corporate positions, opposition to front-of-pack labelling framed as a matter of national identity, and concerns about scientific sponsorship and closed policy-making processes.

 In the sections that follow, we first present the distribution of framing and action strategies identified in the document analysis, illustrating them with examples. We then integrate relevant excerpts from interviews, either to triangulate the documentary evidence or to capture additional framings and practices that emerged spontaneously.

###  Framing Strategies 

 Framing strategies were dominated by narratives portraying food industry actors as *“good actors”* (n = 421; 79%), followed by strategies minimising harms as *individual or trivial problems* (n = 68) and individual-focused, corporate-supported solutions (n = 47). Only three examples framed statutory, population-wide measures as “unacceptable,” and no examples were identified of direct attacks on public health actors (Table 1).

**Table 1:** Distribution of Framing Strategies Used by Food Industry Actors in Portugal (2022–2023)

	**The ‘Good’ Actor: Corporations**	**The ‘Bad’ Actors: Proponents of Whole-Population, Statutory Policies**	**The ‘Trivial’ and ‘Individual’ Problem: Created by a Minority of Consumers**	**The Acceptable, ‘Good’ Solution: Individual-Focused, Corporate Supported**	**The Unacceptable, ‘Bad’ Solution: Whole Population, Statutory**	**Total**
APED	12	0	0	1	0	13
Bimbo+Donuts	13	0	1	2	0	16
Bom Petisco	3	0	3	1	0	7
Cerealis	41	0	7	2	0	50
Coca-Cola	43	0	3	6	0	52
Danone	24	0	23	3	0	50
Delta	16	0	1	1	0	18
Ferrero	19	0	1	1	0	21
FIPA	25	0	0	5	3	33
Iglo	3	0	1	0	0	4
Imperial	3	0	1	0	0	4
J. Martins	26	0	8	4	0	38
Lactogal	7	0	4	0	0	11
Lidl	26	0	2	0	0	28
McDonald's	12	0	2	0	0	14
Mercadona	14	0	2	0	0	16
Nestlé	35	0	1	7	0	43
Novarroz	0	0	0	0	0	0
Nutrinveste S.	11	0	0	0	0	11
PepsiCo	17	0	2	2	0	21
Primor	0	0	0	0	0	0
Sonae	24	0	1	7	0	32
Sumol+Compal	28	0	0	5	0	33
Unilever	16	0	0	0	0	16
Vitacress	5	0	0	0	0	5
Total	421	0	68	47	3	534

Abbreviations: APED, Associação Portuguesa de Empresas de Distribuição (Portuguese Association of Distribution Companies); FIPA, Federação das Indústrias Portuguesas Agro-Alimentares (Federation of Portuguese Agri-Food Industries). Note: Frequencies represent the number of coded framing strategies identified in publicly available materials for each actor, classified according to the CPA framework by Ulucanlar et al.

####  The “Good” Actor: Corporations

 Most of the examples found (n = 421) fell within the category *“The ‘Good’ Actor: Corporations.”* Within this category, companies most frequently mobilised narratives that framed themselves as champions of public health (F-GA6), socially responsible actors (F-GAI7), and key contributors to the national economy (F-GA2). Across actors, framing frequently positioned food industry actors as legitimate and aligned with public health and sustainability goals.

 One of the most frequent framing categories used by commercial actors in Portugal portrayed the industry as a key economic actor (F-GA2). Companies highlighted their contribution to gross domestic product, job creation, innovation, and national development. Coca-Cola, for example, framed its operations in Portugal as a major source of employment, stating: *“A total of more than 12 500 jobs (0.25% of the total workforce), over 12 200 indirect jobs. For each direct job in the system in Portugal, another 35 are generated elsewhere”* [F.CC10]. “*Missão Continente ”* (Sonae) positioned itself as a civic educator, supporting food literacy initiatives and public health campaigns framed as *“contributions to a better world”* [F.MC8]. Ferrero drew on themes of family and ethical responsibility, while Bom Petisco emphasised environmental responsibility and sustainable fishing practices in the Azores: “*Preserving the ocean (…) ensuring that no dolphins are harmed (…) ‘Friend of the Sea’ certification*” [F.BP1]. Bimbo+Donuts promoted digital tools such as *“ Avalie a sua dieta (Assess your diet)”* and *“O Prato Saudável (The Healthy Plate),”* framing these as instruments of consumer empowerment [F.B9]. These initiatives emphasised individual responsibility and behaviour change, with limited reference to structural or regulatory determinants of health. Coca-Cola framed its reformulation and product diversification strategy as supportive of public health and Sustainable Development Goals: “*In line with World Health Organization recommendations (…) our beverage actions support United Nations Sustainable Development Goal 3 – Health and Well-being, as well as Goal 17 (Partnerships for the Goals)*” [F.CC21]. Lidl framed its business model as a “*economic, social and environmental responsibility (…) a responsible business model, not solely focused on financial objectives*” [F.LI9].

 Sumol+Compal and Vitacress promoted their agricultural partnerships as contributions to rural revitalisation and local food systems: *“We invest in national fruit (…) supporting the national economy and promoting sustainability by prioritising local sourcing”* [F.SC1] and *“We support local communities (…) donating products, supporting educational initiatives, and engaging in environmental and social projects”* [F.V2].

####  The “Trivial” and “Individual” Problem: Created by a Minority of Consumers 

 A total of 68 examples were identified under the category *“The ‘Trivial’ and ‘Individual’ Problem,”* where health harms were minimised or attributed to consumer misuse. Explicit instances were often subtle rather than direct. In these cases, harms were reframed as a matter of individual behaviour, moderation, or informed choice, thereby shifting responsibility away from product-related or structural determinants. For example, Ferrero emphasised that “*all types of foods can be consumed as part of a varied and balanced diet*” and highlighted the role of consumers in making appropriate dietary choices [F.F6]. Similarly, Coca-Cola stressed individual choice, stating that “*each person can choose*” [F.C20]. This pattern was also reflected in interview data, with one academic interviewee noting that:

*“Companies rarely deny harm; they say the problem is how people consume the products”* [Academic].

####  The Acceptable, “Good” Solution: Individual-Focused, Corporate Supported

 Framing that promoted individual-focused, corporate-supported solutions (n = 47) was also present. Rather than endorsing structural or regulatory interventions, these narratives emphasised education, information provision, and behaviour change, positioning industry actors as key facilitators of healthier choices. Several companies promoted tools and initiatives designed to support individual decision-making. For example, Sonae developed the initiative “*À Roda da Alimentação (Around Food),”* aiming to “*inspire a change in habits and lifestyles*” [F.S22]. Similarly, Nestlé highlighted its efforts to “*provide consumers with transparent and reliable nutritional information*” and to support healthier behaviours through educational programmes targeting children and families [F.N26; F.N28]. Trade associations like APED and FIPA highlighted their participation in “*voluntary codes*” and “*collaborative agreements*,” presenting these as sufficient responses to policy concerns [F.A12; F.FI4; F.FI14].

 “*The industry usually argues that self-regulation is sufficient” *[Government].

####  The Unacceptable, “Bad” Solution: Whole Population, Statutory

 Three examples from FIPA were identified under the category *“The Unacceptable, ‘Bad’ Solution.”* These statements questioned the proportionality, evidentiary basis, or economic impact of regulatory policies, particularly fiscal measures. FIPA described taxes as *“negative discrimination”* [F.FI8], claimed that they *“would have negative effects on consumption, companies and jobs”* [F.FI8], and argued that their *“practical effects lack solid support”* [F.FI13].

####  The “Bad” Actors: Proponents of Whole-Population, Statutory Policies

 No examples were identified in Portugal of the industry directly attacking or discrediting public health actors, civil society organisations, or policy-makers advocating for statutory regulation, an existing framing category in the international taxonomy.

###  Action Strategies

 We identified 799examples of action strategies (Table 2). The most frequent was *“Manage reputations to corporate advantage”* (n = 450; 56%), followed by *“Displace and usurp public health”* (n = 156), *“Manufacture public support for corporate positions”* (n = 94), and *“Shape evidence to manufacture doubt”* (n = 92). Only seven examples involved *“Access and influence policy-making,”* and none concerned *“Use of the law to obstruct policies.”*

**Table 2:** Distribution Of Action Strategies Used by Food Industry Actors in Portugal (2022–2023).

	**Access and Influence Policy-Making**	**Use the Law to Obstruct Policies**	**Manufacture Public Support for Corporate Positions**	**Shape Evidence to Manufacture Doubt**	**Displace and Usurp Public Health**	**Manage Reputations to Corporate Advantage**	**Total**
APED	1	0	1	1	0	5	8
Bimbo+Donuts	0	0	0	0	5	6	11
Bom Petisco	0	0	1	0	0	1	2
Cerealis	0	0	5	5	9	6	25
Coca-Cola	0	0	2	0	8	60	70
Danone	0	0	0	1	20	4	25
Delta	0	0	0	4	0	3	7
Ferrero	0	0	0	0	11	10	21
FIPA	5	0	10	6	0	4	25
Iglo	0	0	0	1	2	6	9
Imperial	0	0	0	0	0	5	5
J. Martins	0	0	4	13	7	13	37
Lactogal	0	0	4	3	2	5	18
Lidl	0	0	3	3	9	84	99
McDonald's	0	0	2	5	11	31	49
Mercadona	1	0	4	5	1	31	42
Nestlé	0	0	18	18	20	55	111
Novarroz	0	0	0	3	1	1	5
Nutrinveste S.	0	0	3	0	1	11	15
PepsiCo	0	0	1	2	4	9	16
Primor	0	0	6	1	3	2	12
Sonae	0	0	24	9	26	65	124
Sumol+Compal	0	0	3	9	16	20	48
Unilever	0	0	1	3	0	6	10
Vitacress	0	0	2	0	0	7	9
Total	7	0	94	92	156	450	799

Abbreviations: APED, Associação Portuguesa de Empresas de Distribuição (Portuguese Association of Distribution Companies); FIPA, Federação das Indústrias Portuguesas Agro-Alimentares (Federation of Portuguese Agri-Food Industries). Note: Frequencies represent the number of coded action strategies identified in publicly available materials for each actor, classified according to the CPA framework by Ulucanlar et al.

 The most active companies were Sonae (n = 124), Nestlé (n = 111), and Lidl (n = 99), while others such as Bom Petisco (n = 2), Novarroz (n = 5), and Imperial (n = 5) showed little activity (Table 2).

####  Access and Influence Policy-making

 Examples identified under this category included its ability to access policy-making spaces and influence regulatory processes from within (n = 7). FIPA reported its participation in European and national platforms, including FoodDrinkEurope, and described its goal of *“ensuring national representation at the European level”* [A.FI1]. At the national level, FIPA stated that it sought to *“promote synergies between the food industry and political decision-makers, partners, and society as a whole”* [A.FI2]. The 2019 Reformulation Agreement *(“ Compromisso Alargado para a Reformulação dos Produtos Alimentares ”*) was described by interviewees as an example of institutionalised co-regulation: an agreement celebrated as progress yet repeatedly cited by interviewees as a measure that pre-empted stronger regulation. This protocol was signed between “*FIPA, APED, and the Ministry of Health, together with several sectoral associations*,” which was presented as a voluntary success story [A.A1].

 Interview data described access to decision-making processes as characterised by limited transparency and reliance on informal networks. Participants referred to revolving door dynamics between public institutions and industry organisations, as well as sustained contact with political institutions and early engagement with emerging policy agendas. Lobbying was framed by industry representatives as a legitimate and systematic activity aimed at shaping political commitments. These accounts suggest that industry presence within policy-making spaces was normalised and embedded within routine interactions.

 “*Objectively, it’s difficult, very difficult (to prove that the industry forced the non-adoption of the salt tax)”* [Public Health].

 “*What we do is approach political power with well-founded positions, based on concrete data whenever available. Lobbying is legitimate. We must take well-prepared positions, defend them, and seek commitments” *[Food Industry].

 “*We present documents to Parliament after each electoral cycle, with key priorities for the industry. We meet with MPs [Members of Parliament], that’s part of democracy. When a new Minister takes office, I try to get to know more about them. I read the party programmes before elections to ensure consistency later”* [Food Industry].

 Industry opposition to regulation was particularly visible in the Nutri-Score debate. In this context, resistance to front-of-pack labelling was framed as a defence of national identity and food culture rather than as opposition to public health objectives, thereby recasting regulatory intervention as culturally inappropriate rather than evidence-based policy.

 “*(…) we did write to the Portuguese government because we disagreed with the measure”* [Food Industry].

####  Use the Law to Obstruct Policies

 No documentary examples were found in Portugal under that category.

####  Manufacture Public Support for Corporate Positions

 Documentary evidence showed multiple examples of the industry manufacturing public support for its positions (n = 94): Nestlé, for example, emphasised coalition-building around front-of-pack labelling, stating that it had *“joined more than 30 researchers, members of parliament, and consumer organisations, as well as other companies”* to promote Nutri-Score at the European level [A.N21]. Similarly, “*Missão Continente ”* developed broad-based educational and partnership initiatives, including the programme “*Escola Missão Continente*,” which aimed to reach *“students, teachers, parents, and the wider school community”* by promoting healthy eating and sustainable consumption [A.S3]. Its campaigns also relied on institutional and media partnerships, with support from organisations such as the Portuguese Nutrition Association, the National School of Public Health, TVI, and the Portuguese Football Federation [A.S2]. Lactogal’s campaign *“Mimosa Vem Lanchar ”* likewise featured *“well-known Portuguese public figures”* and was broadcast live on national television [A.L2].

 These initiatives were described as part of alliance-building and media engagement efforts that extended beyond commercial advertising.

 “*This is one of the strategic areas of lobbying. When we want to engage in lobbying, we must build alliances. That’s how we structure our strategy”* [Food Industry].

 “*Companies often send out press releases, trying to influence the media agenda. The content may be informative, but it’s still promotional; it’s designed to land in the news, not just in the ads”* [Media].

####  Shape Evidence to Manufacture Doubt

 Documentary evidence of the industry shaping evidence included partnerships with academic institutions, support for knowledge production, and sponsorship of scientific events (n = 92). Nestlé, for example, established a collaboration with NOVA Medical School, described as a “*partnership between the food industry and academia*” aimed at involving final-year nutrition students in “*the development of innovative, healthy and sustainable food products*” as part of their curricular training [A.N4; A.N5]. Similarly, Sumol+Compal supported educational resources through the *Centro de Frutologia Compal*, contributing to the development of materials such as an e-book with “*useful recommendations to promote healthy and sustainable eating through fruit consumption*” [A.SC2]. In parallel, Continente sponsored and actively participated in scientific conferences, including satellite symposia at the Portuguese Congress on Food and Nutrition, addressing “*private label nutrition challenges and opportunities*” and “*innovation in private labels in partnership with nutrition*” [A.S99; A.S100]. Industry presence also extended to oral scientific communications [A.S101; A.S102], alongside formal sponsorship of the congress itself [A.S103; A.SC38; A.SC39; A.V8; A.V9; A.C19; A.C20; A.JM24; A.JM25; A.L12; A.L13; A.L12; A.L13; A.LI97; A.LI98; A.N93; A.N94; A.NO4; A.PR10; A.S103].

 “*We naturally try, like any organization, to develop studies that support our position. If the study turns out unfavorably, I won’t go public with it. I’ll change the argument” *[Food Industry].

 “*If I want to convince the government to lower the VAT [value added tax] on food, I’ll need consultants, advisory corporations, to conduct a proper study. The worst thing would be to just say it myself”* [Food Industry].

 At the same time, academic interviewees raised concerns about how industry-funded research and partnerships were communicated and interpreted. The issue was not framed primarily in terms of scientific quality, but in relation to selective presentation, lack of transparency, and the risk that audiences would draw conclusions based on incomplete or biased information. Several interviewees described industry funding of research as creating potential conflicts of interest, particularly when sponsorship was not made visible, and as exploiting structural dependencies of academic institutions on external resources.

 “*It’s not about scientific rigor; these groups have the resources, the technical capacity. The issue is how they present the information, how they communicate it, and how that leads the average consumer to make decisions based on incomplete information”* [Public Health].

 “*We’d say fiber is important, and next thing you know, something ultra-processed is already being marketed based on that. It turns public health messaging into product marketing”* [Academic].

####  Displace and Usurp Public Health

 Documentary evidence indicates that food industry actors engaged in practices that displaced or appropriated public health functions by positioning themselves as providers of nutrition guidance and health-related interventions (n = 156). These activities targeted schools, families, and professional audiences and were framed as contributions to health promotion, thereby occupying spaces traditionally associated with public institutions and public health authorities. Examples included industry-led standards and guidelines that paralleled or substituted public regulation. Nestlé’s “Nestlé for Healthier Kids” programme, implemented in partnership with the Directorate-General for Education, has “*reached millions of children across the country*” and delivers activities through schools, families, and the wider community to promote healthier and more sustainable diets [A.N10]. Similarly, Lidl’s “*Turma Imbatível*” programme, implemented in primary schools and pre-school settings, aimed to “*promote physical activity among children*” while teaching topics such as “*healthy lifestyles, sustainability, and responsible consumption*” [A.LI5]. This initiative was supported by public institutions, including the Directorate-General for Education and the Directorate-General of Health, alongside professional and environmental organisations, reinforcing its legitimacy [A.LI5]. Industry actors also engaged in professional training and capacity-building activities typically associated with public health systems. Nestlé Health Science, for example, co-organised a national course in geriatric nutrition in partnership with medical and scientific societies, bringing together over 130 health professionals to discuss clinical nutrition practices [A.N7].

 “*There were school-based nutrition education programmes from the food industry, one from Nestlé, another by the sugar refining sector. These filled a gap at a time when there was no structured public investment in food education”* [Public Health].

####  Manage Reputations to Corporate Advantage

 Documentary evidence indicates that food industry actors systematically use CSR activities to manage and enhance their public reputation (n = 450). These initiatives associated corporate brands with social value, sustainability, and community engagement, thereby strengthening institutional legitimacy and public credibility.

 Across actors, companies highlighted large-scale social programmes, partnerships with health institutions, and contributions to community well-being. For example, Jerónimo Martins reported “*supporting more than 29 000 individuals in vulnerable contexts and reaching an estimated 4.5 million people through social and health-related initiatives*” [A.JM18]. Similarly, Lidl explicitly positioned its activities within the UN Global Compact and Sustainable Development Goals, including “*gender equality (SDG 5), responsible consumption (SDG 12), and climate action (SDG 13)*” [A.LI74; A.LI75; A.LI70; A.LI34; A.LI72]. Delta Cafés invested in educational and science-based initiatives through the *“Centro de Ciência do Café (Coffee Science Centre),”* an interactive space designed to “*inform, educate, and promote knowledge*” about coffee through cultural and scientific engagement [A.DE2].

 “*We highlight our responsibility. It includes work with schools, teaching kids how to eat better, read nutrition labels, and eat more fruits and vegetables. That content is always produced by nutritionists, and yes, it’s paid for by the company”* [Food Industry].

###  Contextual Features Shaping CPA in Portugal

 Interviewees described several contextual features shaping the environment in which CPA occurs. Policy-making was frequently described as a “closed process” where influence depends on proximity, trust, and personal networks rather than transparent procedures. The lack of disclosure and reporting requirements limits public scrutiny. Another topic that arose was that the weak or fragmented public provision in areas such as nutrition education and health promotion creates spaces that can be occupied by industry-led initiatives.

 “*In the absence of public campaigns, the industry stepped in to fill the gap” *[Public Health].

 “*Self-regulation only becomes relevant because public oversight is weak”* [Civil Society].

*“The problem isn’t collaboration with industry: it’s the lack of a strong and coordinated presence from the State”* [Academic].

 These observations were described by participants as reflecting broader governance features within which CPA occurs.

###  Illustrative Policy Examples

 The dynamics identified above were particularly visible in two legislative measures, which, although adopted several years earlier, continued to emerge in both publicly available materials and interview accounts. These cases illustrate how corporate strategies adapt across contexts, combining framing narratives with concrete actions to protect business interests.

####  Illustrative Policy Example 1 – Sugar-Sweetened Beverage Tax (Law no. 42/2016)

 The introduction of a tax on sugar-sweetened beverages in 2016 was framed as a flagship measure of Portugal’s health policy, aiming to reduce sugar consumption and encourage reformulation.^33^ While public health authorities and civil society organisations presented the tax as a structural intervention to shift consumption patterns, the beverage industry reacted with strong opposition. Industry actors sought to weaken the legitimacy of the measure by reframing its effects and questioning its regulatory rationale. Documentary evidence shows how trade associations framed fiscal measures as unjustified and harmful, arguing that increases in taxation could have “*negative effects on consumption, companies and employment*” and that their “*practical effects lack solid support*” [F.FI8]. These narratives portrayed regulation as economically damaging and scientifically unfounded, thereby challenging the policy’s legitimacy.

 “*We were flooded with so-called hearings by a Portuguese beverage association. They released a report claiming that the reduction in sugar content had nothing to do with the sugar tax; it was just the market responding to consumer demand. It was all framed as evidence but clearly designed to undermine the policy”* [Government].

 In parallel, companies such as Coca-Cola highlighted voluntary reformulation and product diversification strategies, presenting these as aligned with international health recommendations. For example, the company stated that its actions were “*in line with WHO recommendations*” and aimed to support healthier consumption patterns through increased availability of low- and no-sugar products [F.CC21], alongside commitments such as achieving “*at least 50% of sales from low- or no-calorie beverages*” and reporting reductions in sugar content across its portfolio, highlighted their own voluntary reformulation programmes, presenting these as evidence of corporate responsibility while omitting the role of fiscal regulation in driving reformulation [A.CC5; A.CC6; A.CC7].

####  Illustrative Policy Example 2 – Advertising Restrictions to Children (Law no. 30/2019)

 The 2019 law restricting the marketing of unhealthy foods and beverages to children under 16^34^ was another area where CPA was clearly visible in Portugal. Industry actors contested the scope of the law, particularly provisions limiting advertising in sports venues and around schools. This opposition focused on reframing the law as economically harmful and socially inappropriate rather than as a health protection measure.

 “*When we contested the law on advertising to children, especially the part about banning food ads in sports venues, we spoke with the Olympic Committee. They were going to be affected*” [Food Industry].

 By mobilising respected sports organisations, companies sought to frame the law as economically damaging and culturally inappropriate.

 At the same time, corporations such as Ferrero highlighted their participation in voluntary codes, claiming that they “*do not advertise to children under 12*” [F.F3- F.F6], and launched programmes like Kinder Joy of Moving in schools and sports federations [A.F11]. These initiatives repositioned industry action as responsible self-regulation and shifted attention away from statutory restrictions.

 Interviewees reported limited monitoring capacity, which they perceived as enabling continued exposure of children to food advertising despite the formal adoption of the measure.

 “*We saw food ads still running on children’s TV channels even after the law. Monitoring is almost non-existent*” [Civil Society].

## Discussion

 This study provides the first comprehensive analysis of how food industry actors in Portugal use CPA, revealing a predominance of reputational and co-regulatory strategies over adversarial tactics.

 When compared to other contexts internationally, the Portuguese case is notable for the absence of confrontational practices such as litigation or public attacks on health advocates, strategies documented in other countries.^18,35,36^ Instead, the CPA of the food industry in Portugal is characterised by “softer,” institutionalised forms of influence, often embedded in formal partnerships with government bodies. While these approaches foster continuity and cooperation, they may also contribute to reinforcing the structural position of industry actors, limiting opportunities for more ambitious reforms. However, this absence of explicit confrontation should be interpreted with caution, as it may partly reflect the focus on publicly available and corporate-facing materials. Prior cross-sector research has shown that unhealthy commodity industries may engage in public discreditation, legal intimidation, surveillance, and other adversarial tactics directed at researchers and advocates.^37^ As our documentary analysis relied on publicly available corporate and institutional materials, such practices may be less visible in these sources and more likely to emerge in consultation submissions, legal disputes, investigative reporting, or qualitative accounts from affected actors. The absence of such examples in the present dataset should therefore not be interpreted as definitive evidence of their absence in the Portuguese context. In other jurisdictions, CPA research has drawn on lobbying registers, parliamentary submissions, and political donation databases to document strategies of access and influence over policy-making, including in Australia, Chile, and Brazil.^12,36,38^ These transparency mechanisms have enabled researchers to identify forms of access-oriented CPA that are not always visible in corporate self-presentations. The absence of comparable lobbying transparency tools in Portugal constrains the ability to capture such strategies through documentary analysis alone and reinforces the importance of complementary qualitative approaches.

 The intensity and diversity of CPA varied substantially across companies. Large retailers and multinationals such as Sonae, Nestlé, and Lidl deployed extensive repertoires, while smaller actors such as Novarroz and Bom Petisco were less active. These disparities may be explained by company size, visibility, or the presence of dedicated public affairs teams, a pattern consistent with international evidence showing that corporations with greater institutional capacity are typically more active in political engagement.^36,39-41^ One question raised by the lower visibility of CPA among smaller industry actors is whether political engagement is partly delegated to trade associations.

 Findings from this study resonate with evidence on corporate influence from other unhealthy commodity industries in Portugal. For instance, an analysis of the alcohol industry’s political practices found that Portugal’s relatively weak alcohol control policies were “probably associated with industry interference.”^27^ Similar to the food sector, alcohol companies cultivated alliances with government and civil society, framed themselves as responsible actors, and promoted voluntary codes of conduct to prevent statutory regulation. However, compared with the alcohol sector, the food industry’s strategies appear more institutionalised and less confrontational, relying heavily on co-regulation and reputational management. This distinction suggests that while both industries use CPA to protect commercial interests, the food sector has appeared to be more closely integrated into formal policy-making structures.

 CSR initiatives emerge as a central component of CPA in Portugal, frequently framed as voluntary contributions to society or sustainability. However, these actions often yield tangible benefits for companies, both reputational and fiscal. Under the Portuguese *Estatuto dos Benefícios Fiscais (Tax Benefits Code)*,^42^ donations to organisations with public utility status can be deducted at enhanced rates, creating a dual incentive structure where companies simultaneously bolster their public image and reduce their tax burden. CSR may function simultaneously as a reputational strategy and a fiscal optimisation mechanism. Similar patterns have been documented in other industries: in the United States of America, tobacco companies strategically used philanthropy as a form of political influence, with tax-deductible donations serving as substitutes for direct lobbying.^43^ Evidence from the food sector points to comparable practices. In Mexico, soft drink companies funded school and community projects while opposing stronger regulation on sugar-sweetened beverages, using philanthropy to secure political goodwill.^44^ In the UK, food and beverage companies used community sponsorships and CSR initiatives to position themselves as partners in health promotion, even while lobbying against statutory measures such as advertising restrictions and sugar taxes.^35^ More broadly, CSR has been described in the literature as part of a repertoire of political strategies designed to align corporate interests with public policy while constraining regulatory ambition.^43,45^

 Displacement of public health functions was the other mechanism identified. Companies increasingly provide education, guidance, and scientific sponsorship, positioning themselves as public health partners and blurring the boundaries between corporate marketing and health governance. Such practices may contribute to the gradual substitution of public institutions by corporate actors, further embedding private interests in health governance.

 Interview data highlighted ambivalence towards corporate involvement. While some participants acknowledged technical expertise, particularly in reformulation and logistics, others expressed concern that collaboration risks regulatory capture, with voluntary mechanisms serving strategic rather than public health goals. Interview data also revealed tensions around the use of Ulucanlar’s international CPA taxonomies. Criticism of the framework was expressed primarily by interviewees from industry and, to a lesser extent, by academics with institutional or funding links to commercial actors, who perceived the taxonomy as negatively framed or insufficiently recognising actions they considered legitimate or beneficial. These perspectives reflect different actors’ positions and conflicts of interest, rather than limitations of the analytical framework itself.

 Interview findings suggest that CPA is facilitated by broader contextual features of the Portuguese governance environment, institutional proximity, and public–private partnerships in policy-making. Interpreted through the lens of the CDoH, these findings illustrate how corporate power operates through structural and discursive mechanisms embedded in governance systems.^4^

 Overall, these findings depict a political environment where the food industry in Portugal relies less on overt confrontation and more on reputational, collaborative, and institutionalised strategies. While seemingly cooperative, such practices consolidate corporate legitimacy, displace public health functions, and exploit weak institutional frameworks, potentially limiting the scope for more ambitious public health reforms. Importantly, these findings underscore that the adoption of health-protective regulations is not, in itself, sufficient to safeguard public health. Effective implementation requires sustained investment in regulatory enforcement, monitoring mechanisms, and institutional capacity; otherwise, policies risk being weakened in practice through co-regulatory arrangements, limited oversight, or reliance on voluntary compliance.

###  Policy Implications

 Our findings highlight several implications for public health policy in Portugal. First, transparency mechanisms should be reinforced through strong lobbying registers, systematic disclosure of corporate participation in policy-making, and safeguards against conflicts of interest. Interviewees themselves suggested measures such as mandatory disclosure of meetings with policy-makers and clearer accountability rules for public–private partnerships. Evidence from a recent scoping review confirms that such mechanisms are effective in limiting undue corporate influence.^46^

 Second, boundaries should be established for CSR-related partnerships, particularly in education, science, and initiatives targeting vulnerable populations to prevent reputational and political benefits from substituting for regulatory action. As the CDoH literature has shown, CSR often compensates for weaknesses in public institutions, partly created by insufficient public funding and corporate tax avoidance.^5^

 Third, sustained investment in independent public institutions is crucial. Adequate resourcing would enable them to act autonomously, reduce reliance on industry-led initiatives, and ensure that public health priorities are determined in the public interest rather than through corporate agendas.

###  Strengths and Limitations

 This study is the first systematic analysis of the CPA of the food industry in Portugal, combining documentary data (n = 534 framing and 799 action strategies) with 18 semi-structured interviews. By triangulating sources, we were able to capture both observable practices and insider perspectives, enhancing the validity and richness of the analysis. In addition, the use of an internationally recognised taxonomy of CPA allows for comparability with studies in other countries and for other industries, contributing to the global evidence base on industry influence.

 However, some limitations must be acknowledged. First, documentary analysis is constrained by the availability of information; corporate strategies that remain private or informal may not have been captured. Second, while interviews provided valuable insights, they involved a relatively small sample (n = 18) and may not have fully reported the diversity of CPA practices used by the food industry in Portugal. Third, the interpretation of frequency counts and references to “most active” actors should be approached with caution. Differences in observed activity may partly reflect variations in corporate visibility, communication capacity, or reliance on trade associations, rather than differences in actual influence over policy-making. Fourth, although source websites were recorded for each coded example, individual uniform resource locators were not systematically archived. Coding was conducted from screenshots of publicly available webpages captured during data collection. While this approach ensured preservation of content as it appeared at the time, it may limit exact page-level replication.

 The focus on Portugal provides contextual depth but limits generalisability, as CPA strategies are shaped by national institutional and cultural settings. Future research should extend the timeframe, examine internal industry documents, and compare CPA across different sectors and policy topics in Portugal. Further work should also evaluate the effectiveness of mechanisms to address the CPA and conduct cross-country analyses to understand how institutional contexts shape industry influence.Future work could also build on this work by systematically analysing industry submissions to regulatory consultations and policy reviews, which may allow a more comprehensive assessment of confrontational framings and oppositional CPA strategies that are not easily captured through publicly available sources. And finally, this study did not examine the role of government actors or regulatory frameworks in shaping or responding to CPA. Future research should explore these dimensions to better understand interactions between corporate strategies and state capacity.

## Conclusions

 The Portuguese food industry relies primarily on reputational strategies, co-regulation, and institutional partnerships rather than confrontational CPA tactics.

 Observed CPA was concentrated among large retailers, multinationals, and major trade associations. Overall, this form of “soft capture” blurs the boundaries between public and commercial interests, reinforcing corporate legitimacy while constraining public health reform. Strengthening lobbying transparency, clarifying limits on CSR partnerships, and investing in independent public institutions are crucial to rebalance power and protect policy-making from undue corporate influence.

## Acknowledgements

 An earlier version of this work was presented as a conference abstract at the European Public Health Conference 2024 (Lisbon, Portugal). The abstract was published in the *International Journal of Public Health* abstract supplement (available from: https://doi.org/10.1093/eurpub/ckae144.565). We also thank all interview participants for their valuable insights and contributions.

## Disclosure of artificial intelligence (AI) use

 ChatGPT was used for language editing purposes. The authors take full responsibility for the content.

## Ethical issues

 This study received approval from the Ethics Committee of the FCNAUP (reference: 164/2024/CEFCNAUP). This work was also supported by FCT - Fundação para a Ciência e Tecnologia, I.P. through the projects with references UID/4750/2025 and LA/P/0064/2020 and DOI identifiers https://doi.org/10.54499/UID/04750/2025 and https://doi.org/10.54499/LA/P/0064/2020.

## Conflicts of interest

 Pedro Graça is the current Dean of the FCNAUP, and former Director of the PNPAS, Ministry of Health, Portugal. Maria João Gregório is the current Director of the PNPAS and the EIPAS, Directorate-General of Health, Ministry of Health, Portugal. The FCNAUP and the PNPAS/EIPAS are mentioned in some of the documents analysed in this study. Neither Pedro Graça nor Maria João Gregório had any role in data collection or coding of materials related to FCNAUP and PNPAS/EIPAS. Their contribution was limited to methodological guidance, supervision, and critical review of the manuscript. The authors declare no other competing interests.

## Supplementary files


Supplementary file 1. List of Sources of Information Consulted.

Supplementary file 2. Framing and Action CPA Examples of the Portuguese Food Industry.

Supplementary file 3. Interview Guide.
