# Supplementary material for: Reputation, Co-Regulation, and "Soft Capture": Corporate Political Activity of the Portuguese Food Industry
Source: Int J Health Policy Manag. 2026 Jun 1;15:9495. doi: 10.34172/ijhpm.9495 (PMC13729813; doi:10.34172/ijhpm.9495)
Supplement: Supplementary file 1 [file ijhpm-15-9495-s001.pdf]

Article title: Reputation, Co-Regulation, and “Soft Capture”: Corporate Political Activity of the Portuguese Food Industry

Journal name: International Journal of Health Policy and Management (IJHPM)

Authors’ information: Sofia da Costa[1,2]*, Mélissa Mialon[3,4], Marta Figueira[2,5], Maria João Gregório[1,2,5], Pedro Graça[1,2]

[1]Epidemiology Research Unit (EPIUnit), Institute of Public Health, University of Porto, Porto, Portugal.

[2]Faculty of Nutrition and Food Sciences, University of Porto, Porto, Portugal.

[3]Inserm, Université Paris Cité, Paris, France.

[4]School of Medicine, Trinity College Dublin, Dublin, Ireland.

[5]National Healthy Eating Promotion Program, Directorate-General of Health, Lisbon, Portugal.

***Correspondence to:** Sofia da Costa; Email: sofiacarvalhodacosta@gmail.com

Citation: da Costa S, Mialon M, Figueira M, Gregório MJ, Graça P. Reputation, co-regulation, and “soft capture”: corporate political activity of the Portuguese food industry. Int J Health Policy Manag. 2026;15:9495.doi:10.34172/ijhpm.9495

Supplementary file 1. List of Sources of Information Consulted

Industry Materials

Associação Portuguesa de Empresas de Distribuição (APED): aped.pt
Bimbo Donuts Portugal: bimbo.pt, compromissobimbo.bimbo.pt, donuts.pt, bimboglobalrace.com
Bom Petisco: bompetisco.pt
Cerealis: cerealis.pt
Coca-Cola Portugal: cocacolaportugal.pt, coca-colacompany.com, coca-cola.pt
Danone Portugal: danone.pt, danone.com
Delta Cafés: deltacafes.pt, gruponabeiro.com
Ferrero Ibéria: ferrero.pt, ferrerosustainability.com, kinderjoyofmoving.com
Federação das Indústrias Portuguesas Agro-Alimentares (FIPA): fipa.pt
Iglo Portugal: green-cuisine.com, iglo.pt
Imperial: imperial.pt
Jerónimo Martins (Pingo Doce): jeronimomartins.com, pingodoce.pt
Lactogal: lactogal.pt, mimosa.com.pt
Lidl: institucional.lidl.pt, lidl.pt
McDonald's Portugal: fundacaoronaldmcdonald.com, mcdonalds.pt
Mercadona: mercadona.es
Nestlé Portugal: empresa.nestle.pt, saboreiaavida.nestle.pt
Novarroz: novarroz.pt

Nutrinveste - Sovena: nutrinveste.pt, sovenagroup.com
Pepsico Portugal: pepsico.pt
Primor - Charcutaria Prima SA: primor.pt, grupoprimor.pt
Sonae (Continente): sonae.pt, missao.continente.pt, arodadaalimentacao.pt
Sumol+Compal: sumolcompal.pt, sumolcompalportasabertas.pt, compalworld.com
Unilever FIMA: unilever-fima.com, wiop.unilever-fima.com
Vitacress: vitacress.pt

Government Materials
Direção-Geral da Saúde (DGS): dgs.pt
PNPAS / EIPAS (Plano Nacional para a Alimentação Saudável): alimentacaosaudavel.dgs.pt
Instituto Nacional de Saúde Doutor Ricardo Jorge (INSA): insa.min-saude.pt
Parlamento de Portugal: parlamento.pt
Partido Socialista (PS): ps.pt
Partido Social Democrata (PSD): psd.pt

Other Materials
Faculty of Nutrition and Food Sciences, University of Porto: up.pt/fcnaup/pt
Faculty of Medicine, University of Lisbon: ulisboa.pt
Nova Medical School Lisbon: nms.unl.pt
Lisbon School of Health Technology: estesl.ipl.pt
Coimbra School of Health Technology: estesc.ipc.pt
ISPUP (Instituto de Saúde Pública da Universidade do Porto): ispup.up.pt
ESB-UCP (Escola Superior de Biotecnologia, Universidade Católica Portuguesa): esb.ucp.pt
Associação Portuguesa de Nutrição (APN): apn.org.pt, actaportuguesadenutricao.pt
Congresso de Nutrição e Alimentação da Associação Portuguesa de Nutricionistas: cna.org.pt
Ordem dos Nutricionistas: ordemdosnutricionistas.pt
