# Supplementary material for: Reputation, Co-Regulation, and "Soft Capture": Corporate Political Activity of the Portuguese Food Industry
Source: Int J Health Policy Manag. 2026 Jun 1;15:9495. doi: 10.34172/ijhpm.9495 (PMC13729813; doi:10.34172/ijhpm.9495)
Supplement: Supplementary file 2 [file ijhpm-15-9495-s002.pdf]

Article title: Reputation, Co-Regulation, and "Soft Capture": Corporate Political Activity of the Portuguese Food Industry

Journal name: International Journal of Health Policy and Management (IJHPM)

Authors' information: Sofia da Costa[1,2]*, Mélissa Mialon[3,4], Marta Figueira[2,5], Maria João Gregório[1,2,5], Pedro Graça[1,2]

[1]Epidemiology Research Unit (EPIUnit), Institute of Public Health, University of Porto, Porto, Portugal.

[2]Faculty of Nutrition and Food Sciences, University of Porto, Porto, Portugal.

[3]Inserm, Université Paris Cité, Paris, France.

[4]School of Medicine, Trinity College Dublin, Dublin, Ireland.

[5]National Healthy Eating Promotion Program, Directorate-General of Health, Lisbon, Portugal.

***Correspondence to:** Sofia da Costa; Email: sofiacarvalhodacosta@gmail.com

Citation: da Costa S, Mialon M, Figueira M, Gregório MJ, Graça P. Reputation, co-regulation, and "soft capture": corporate political activity of the Portuguese food industry. Int J Health Policy Manag. 2026;15:9495.doi:10.34172/ijhpm.9495

Supplementary file 2. Framing and Action CPA Examples of the Portuguese Food Industry

Framing examples of the Portuguese Food Industry

APED

Website	Code	CPA EXAMPLES
APED	F.A1	A Associação Portuguesa de Empresas de Distribuição (APED) congrega as empresas do setor retalhista, grossista e de comércio eletrónico, que juntas representam mais de 12,4% do Produto Interno Bruto de Portugal. Assumindo-se como a voz ativa e representativa deste setor fundamental para a economia, a sua atuação é pautada pela procura de soluções construtivas para toda a cadeia de valor, que melhoram a competitividade da economia nacional.
APED	F.A2	Ao longo dos últimos anos as suas associadas têm assumido um papel preponderante na formação e requalificação dos seus colaboradores, tendo criado 6 mil novos postos de trabalho.
APED	F.A3	São a força motriz que todos os dias contribui para o desenvolvimento económico do país e, por conseguinte, para a criação de mais emprego.
APED	F.A4	O setor do retalho é, provavelmente, um dos setores que maior contributo tem dado para o modelo de Economia do Futuro. Um modelo fortemente ancorado nos desenvolvimentos tecnológicos que têm feito florescer novas atividades e novos processos.
APED	F.A5	Os resultados obtidos espelham o compromisso e o investimento da Indústria Alimentar e da Distribuição na reformulação de um alargado conjunto de categorias de alimentos, num trabalho multidisciplinar assente numa postura de colaboração, esforço e vontade de entidades do setor público e privado.

BIMBO+DONUTS

Website	Code	CPA EXAMPLES
BIMBO+ DONUTS	F.B1	Alimentos deliciosos e nutritivos nas mãos de todos.
BIMBO+ DONUTS	F.B2	O nosso compromisso A Bimbo Portugal, preocupa-se consigo, com a sua alimentação e a da sua família; é por isso que preparamos alimentos fofos, saborosos, frescos e nutritivos todos os dias. ! Criamos o Compromisso Bimbo para levar os melhores produtos à sua mesa e para garantir que melhoramos continuamente a sua composição nutricional, comunicamos de maneira responsável e transparente, promovemos um estilo de vida saudável e somos responsáveis pelo planeta. Este é o nosso esforço para preparar bons hábitos e permitir que desfrutemos dos pequenos momentos de cada dia. Da nossa família para a sua. Bimbo ®
BIMBO+ DONUTS	F.B3	Compromisso Nutricional Bimbo nasce com a missão de colocar alimentos deliciosos e nutritivos nas mãos de todos. Por esta razão, temos vindo a realizar melhorias na composição nutricional dos nossos produtos e apostando na qualidade dos ingredientes que usamos para a sua elaboração. As iniciativas realizadas têm um objetivo em comum, trazer para a sua mesa os melhores produtos e garantir que, na medida em que a tecnologia nos permita, vamos continuar a melhorar sua composição nutricional.
BIMBO+ DONUTS	F.B4	A Bimbo Portugal, quer cuidar de si
BIMBO+ DONUTS	F.B5	Construir uma empresa sustentável, altamente produtiva e plenamente humana.
BIMBO+ DONUTS	F.B6	Alimentamos um mundo melhor.

BIMBO+ DONUTS	F.B7	Nossos valores: somos uma comunidade; concorremos e ganhamos; agimos com integridade; atingimos resultados; somos colaboradores eficientes; transcendemos e evoluímos no tempo.
BIMBO+ DONUTS	F.B8	Compromisso com o Planeta Cuidar do nosso planeta é responsabilidade de todos, é por isso que a Bimbo Portugal está empenhada em cuidar do nosso ambiente. Para isso, trabalhamos em diferentes áreas para reduzir o nosso impacto ambiental. Para assegurar que os nossos produtos chegam à sua mesa em ótimas condições, as embalagens cumprem uma função fundamental de proteção, que garante a conservação adequada dos alimentos. Assim garantimos a segurança, higiene e preservação da vida útil dos nossos produtos, sendo o plástico um dos materiais com maior adaptação e resposta a estas necessidades. No entanto, sabemos que o fabrico e a gestão dos resíduos plásticos provenientes das embalagens é um desafio para o equilíbrio do planeta. Por isso, confiamos no eco desenho para a nossa aposta em embalagens mais sustentáveis. Trabalhamos em novas linhas estratégicas para a redução dos resíduos provenientes das embalagens. Atualmente, a Bimbo Portugal toma medidas de sustentabilidade:
BIMBO+ DONUTS	F.B9	Compromisso ativo A Bimbo Portugal promove um estilo de vida saudável. Lançamos uma plataforma online com guias para uma alimentação saudável e prática de atividades físicas e bem-estar. Inclui várias ferramentas e desafios que ajudam a consolidar novos hábitos benéficos para a saúde. A ferramenta Avalie a sua dieta analisa a alimentação habitual de cada pessoa, fornecendo dicas para a melhorar. O Prato Saudável mostra como equilibrar almoços e jantares de uma forma simples e visual. Tudo isso em conjunto com menus que facilitam o planeamento de refeições com ideias de receitas equilibradas e alimentos da época.
BIMBO+ DONUTS	F.B10	Compromisso de Transparência Temos orgulho do modo como fazemos as coisas, é por isso que o contamos de forma transparente, comprometidos com os consumidores, com a sua saúde e o seu bem-estar. Acreditamos que todos devemos ter em mãos as informações necessárias para fazer escolhas alimentares de acordo com as necessidades nutricionais, circunstâncias e preferências. A Bimbo Portugal ouve os consumidores e a sociedade. Mantê-los informados e satisfazer as suas necessidades são alguns dos pontos mais importantes para nós.
BIMBO+ DONUTS	F.B11	Muito mais do que uma corrida #RunWithus

BOM PETISCO

Website	Code	CPA EXAMPLES
BOM PETISCO	F.BP1	A mais popular conserva portuguesa, Bom Petisco conta com uma história tão especial quanto o seu atum. A origem da indústria conserveira nacional remonta ao século XIX, quando Vila Real de Santo António era o ponto de encontro das indústrias da pesca e conservação. Foi aqui que, durante mais de um século, a pesca do atum se concentrou, para resultar em conservas que alimentavam milhares de pessoas por todo o mundo. À medida que a indústria se desenvolvia, também a procura aumentava e, gradualmente, a conserva de atum conquistou um lugar privilegiado na mesa de muitas famílias. O crescimento foi tal que, no final do século XX, a costa algarvia começou a mostrar sinais de sobre-exploração. Ao mesmo tempo, o atum deu início a um processo de alteração da rota migratória. Perante esta situação, a indústria conserveira começou a procurar alternativas e é assim que, em 1962, Bom Petisco conquista o seu ADN açoriano. Ponto de passagem privilegiado na rota migratória de muitos animais marinhos, com águas frias e peixe de qualidade ímpar, as ilhas açorianas são o local escolhido para a instalação da fábrica da marca. Atualmente, orgulhamo-nos de poder dizer que parte da nossa produção é feita com peixe fresco, pescado nos Açores.
BOM PETISCO	F.BP2	Preservar o oceano. Porque a sustentabilidade das pescas é uma preocupação de Bom Petisco, a pesca nos Açores rege-se por critérios rigorosos de proteção do ecossistema marinho. A pesca é realizada em colaboração com o POPA – Programa de observação para as pescas dos Açores -, um projeto de monitorização e controlo das pescas. O objetivo é garantir que nenhum golfinho é molestado na nossa atividade pesqueira. A proteção dos golfinhos e sustentabilidade do mar é também assegurada pelo estatuto de “Friend of the Sea”, que visa a redução do impacto humano no equilíbrio do ecossistema marinho.
BOM PETISCO	F.BP3	Bom Petisco conserva o que é bom. É na nossa fábrica em Rabo de Peixe que o atum é enlatado e conservado em óleo, azeite ou água, para depois ser submetido a um processo de esterilização. Sem a utilização de quaisquer conservantes, o resultado é uma conserva 100% natural, muito nutritiva e com um sabor que perdura com o tempo.
BOM PETISCO	F.BP4	O atum, em particular, é um peixe com um perfil nutricional muito interessante. Para além do seu elevado teor proteico, as suas proteínas possuem todos os aminoácidos essenciais – um fator importante, na medida em que o organismo humano não tem a capacidade de produzir estes aminoácidos, estando por isso dependente de alimentos que os forneçam. Os lípidos (ou gorduras) que compõem o atum também se destacam: com uma percentagem reduzida de ácidos gordos saturados e elevados níveis de ácidos gordos polinsaturados, é um alimento rico em ómega 3. Desta forma, revela-se um alimento importante no controlo dos níveis de colesterol no sangue e prevenção de doenças cardiovasculares. A somar a estes benefícios, no atum encontramos ainda uma elevada presença de Ferro, vitaminas e sais minerais, sobretudo, fósforo, cálcio e magnésio.
BOM PETISCO	F.BP5	E tudo, numa lata. Por todas estas razões, as conservas de peixe são uma mais-valia inegável na alimentação de todos. O processo de conservação do atum permite-lhe preservar a qualidade de todos os seus nutrientes, simplificando assim a preparação de refeições equilibradas e completas
BOM PETISCO	F.BP6	Com Petisco alimenta campeões. Como pilar fundamental do bem-estar e saúde do ser humano, a atividade física é essencial para um corpo e mente saudáveis. Após o exercício físico, no entanto, o corpo precisa de restituir os nutrientes consumidos, surgindo por isso a importância de uma alimentação adequada. As proteínas, sendo a base da massa muscular, merecem um lugar de destaque na alimentação de todos os desportistas. Quando consumidas após o exercício físico, contribuem para uma melhor recuperação e manutenção dos músculos, ao contribuírem para a definição da estrutura dos tecidos. Por outro lado, porque apresentam uma grande capacidade de estimulação metabólica, são muito eficazes a saciar o apetite. Uma dieta rica em proteínas permite, assim, controlar os níveis de açúcar e gordura no sangue. Por todas estas razões, o atum Bom Petisco é o companheiro ideal dos desportistas. A escolha acertada para obter refeições nutritivas e convenientes.

CEREALIS

Website	Code	CPA EXAMPLES
CEREALIS	F.C1	Grupo português líder nas massas e farinhas com dimensão internacional, a crescer há 104 anos A Cerealis é um dos maiores grupos portugueses vocacionados para a atividade industrial e comercial do setor agroalimentar, com forte presença em Portugal e em grande dinamização internacional. Focalizado em produtos derivados da transformação de

		cereais, é líder nas massas alimentícias e farinhas industriais, complementando a sua gama com farinhas culinárias, bolachas, cereais de pequeno-almoço e barras de cereais. A Cerealis é detentora das marcas Nacional, Milaneza e Napolitana, emprega 590 colaboradores, e transforma anualmente mais de 440 mil toneladas de cereais nos 4 centros de produção certificados. Os seus mais de 130 produtos de diferentes categorias são hoje comercializados nos cinco continentes.
CEREALIS	F.C2	Enquanto agentes sociais e económicos fundamentais, as empresas têm um papel crítico no alcance dos ODS. São por isso, chamadas a assumir o compromisso de, nas suas atividades, contribuírem ativamente para alcançar as metas estabelecidas.
CEREALIS	F.C3	2.300 clientes , 170 produtos , 200 milhões de faturação , 3.000 fornecedores , +35 novos produtos anualmente
CEREALIS	F.C4	A Cerealis é um Grupo de Português de referência com atividade industrial no setor agroalimentar que produz massas alimentícias, farinhas industriais e culinárias, bolachas e cereais de pequeno almoço. Com forte presença em Portugal e em franco crescimento internacional, a Cerealis comercializa os seus produtos através de Marcas de Fabricante, onde se destacam as reconhecidas marcas Milaneza, Nacional e Harmonia e de Marcas da Distribuição. A Cerealis foi fundada em 1919 e a sua longa história de crescimento e sucesso foi liderada durante 102 anos com empenho e enorme dedicação por 3 gerações das famílias Amorim e Lage. Hoje dá-se um momento importante na história da empresa, que passa a ser totalmente detida pelas famílias Moreira da Silva e Silva Domingues através das respetivas holdings, Teak Capital, BV e Tangor Capital, S.A., cada uma com uma participação de 50% do capital social.
CEREALIS	F.C5	- Somos conscientes do legado empresarial que as famílias fundadoras desenvolveram ao longo de mais de um século.
CEREALIS	F.C6	Assim, a procura de alternativas mais saudáveis e convenientes, tem vindo a ganhar uma maior preponderância, destacando-se alguns movimentos com impactos significativos na indústria alimentar.
CEREALIS	F.C7	Simultaneamente, a indústria alimentar tem respondido com programas muito exigentes de renovação dos seus portefólios, quer seja pela via da reformulação dos seus produtos existentes, quer através do lançamento de novos produtos com uma redução significativa do teor de açúcar, ou com zero teores de açúcares adicionados, dando assim um contributo decisivo para a necessária redução das taxas de ingestão do açúcar.
CEREALIS	F.C8	Na Cerealis acompanhamos a evolução das tendências alimentares, oferecendo soluções de alimentação que ajudam a promover o bem-estar dos nossos consumidores, contribuindo assim para uma dieta completa, variada e rica em nutrientes. A sensibilização dos consumidores e da comunidade para a importância de uma dieta variada e equilibrada, tem sido um compromisso assumido pela Cerealis.
CEREALIS	F.C9	A NOSSA MISSÃO A Cerealis é um Grupo vocacionado para a atividade industrial e comercial do sector agroalimentar, focalizado em produtos derivados da transformação de cereais, assente em práticas de bem fazer, de sustentabilidade e de reforço permanente de competitividade, focalizado nos negócios de massas alimentícias, cereais de pequeno almoço, farinhas industriais e culinárias, onde pretende ser líder, e outros produtos sinergéticos, nomeadamente bolachas e barras de cereais. Ciente da sua responsabilidade na promoção de uma alimentação e estilo de vida saudável, a Cerealis dinamiza uma cultura interna e externa de conhecimento, desenvolvendo produtos nutricionalmente equilibrados. A Cerealis é um Grupo sustentado por pessoas competentes e motivadas.
CEREALIS	F.C10	A NOSSA VISÃO Ser um Grupo Português de referência no setor agroalimentar, que investe na capacidade e excelência industrial, aposta na valorização dos seus recursos humanos e investe na inovação como fatores fundamentais para o seu desenvolvimento. Ambiciona crescer, de forma sustentada, assumindo posições de liderança nos mercados onde atua.
CEREALIS	F.C11	CÓDIGO DE ÉTICA E CONDUTA EMPRESARIAL A Cerealis considera essencial a existência e divulgação de um Código de Ética e Conduta Comercial para todas as Empresas do Grupo e para todos os trabalhadores. Estamos confiantes de que todos os empregados da Cerealis têm vindo a comportar-se de forma ética. Contudo, com a criação deste Código de Ética, os princípios, valores e condutas considerados necessários a todos os trabalhadores e membros da organização são estabelecidos de forma clara e transparente. Esperamos que estas regras se tornem um verdadeiro código de conduta e contribuam para o reforço da imagem e do papel da Cerealis como Grupo de referência na indústria agroalimentar, assim como para reforçar os laços entre todas as partes interessadas, ou seja, acionistas, colaboradores, clientes, fornecedores, parceiros sociais, instituições públicas e a comunidade em geral.
CEREALIS	F.C12	SOMOS SUSTENTADOS POR PESSOAS COMPETENTES E MOTIVADAS. Nós acreditamos que as Pessoas são os elementos chave do Grupo. Ao longo dos anos temos construído uma Política de Recursos Humanos, assente na valorização das pessoas e no desenvolvimento das nossas equipas. Através de um trabalho diário, suportado em programas de Desenvolvimento de Competências, procuramos que os nossos colaboradores, a todos os níveis funcionais, estejam cada vez mais alinhados e comprometidos com a nossa cultura e com as necessidades do nosso negócio, e sejam cada vez mais competentes. Complementarmente e através de uma gestão previsional e estratégica de Recursos Humanos, antecipamos dificuldades, encarando-as como desafios e oportunidades, estimulando soluções de futuro.
CEREALIS	F.C13	Qualidade QUALIDADE E A EFICIÊNCIA OPERACIONAL Na Cerealis promovemos a implementação de Sistemas de Gestão da Qualidade como ferramentas de apoio e sistematização de processos com vista à eficiente otimização dos mesmos, dos recursos e das atividades. A análise de falhas, a definição de causas e de ações corretivas, aliadas á sistematização de ações de melhoria, são o melhor garante do atingimento sustentado de níveis de conformidade e eficiência que a natureza dos produtos e a exigência dos mercados onde atuamos assim nos exigem. A formação contínua em técnicas e ferramentas de Gestão da Qualidade, em Segurança Alimentar e em Boas Práticas da Indústria Alimentar, alicerçadas em definições claras de funções, tarefas, procedimentos e objetivos são a base da orientação de comportamentos e atitudes na construção diária da atividade.
CEREALIS	F.C14	CERTIFICAÇÕES Em todos os centros e atividades produtivas do Grupo, assim como nos processos de comercialização, temos implementados Sistemas de Gestão de Qualidade e de Segurança Alimentar. De destacar a renovação dos certificados de Gestão e Garantia de Qualidade e Segurança Alimentar pelo referencial 'International Food Standard' (IFS) nos processos produtivos de massas alimentícias, farinhas industriais, farinhas de usos culinários, cereais de pequeno-almoço e bolachas. Acompanhando o processo de penetração das nossas marcas nos mercados internacionais, em 2014, obtivemos a certificação Halal na produção de massas, cereais de pequeno-almoço, bolachas e farinhas, e, em 2017, a certificação UTZ mass balance (Cerealis Produtos Alimentares, S.A.). Em 2018, a Cerealis Produtos Alimentares, S.A. obteve a Certificação RSPO mass balance (verifique o nosso progresso em www.rspo.org/members/8091) e, em 2019, a Certificação Kosher (Cerealis Moagens, S.A.).
CEREALIS	F.C15	GESTÃO DE QUALIDADE DO GRUPO A Gestão da Qualidade na Cerealis assenta num sistema transversal a todo o Grupo, consolidado pela análise aos processos que gerem as diversas atividades e famílias de produtos produzidos e

		comercializados pelas áreas de negócio. Este sistema dinâmico e suportado por práticas de melhoria contínua ajusta-se continuamente à evolução tecnológica que o Grupo tem evidenciado, quer do ponto de vista de remodelação e adequação industrial, quer através da investigação e desenvolvimento de novos produtos e processos.
CEREALIS	F.C16	GESTÃO DO IMPACTO AMBIENTAL Com três instalações com Licenciamento Ambiental, na Cerealis estendemos as práticas de redução de desperdícios, reciclagem de resíduos, racionalização de consumos energéticos e políticas de redução de emissões de poeiras a todos os centros produtivos.
CEREALIS	F.C17	ENERGIA E EMISSÕES A energia tem uma importância vital nas economias de hoje, contudo tem um forte impacto ambiental, com especial ênfase nas alterações climáticas e especificamente na qualidade do ar. A tentativa de conciliar estas duas perspetivas passa, entre outros, pela adoção de uma política energética que privilegie uma economia de baixo carbono e um aumento da eficiência energética, com claros benefícios económicos e ambientais. Nos últimos anos investimos fortemente em Planos de Racionalização dos Consumos de Energia com o objetivo de atingir as metas de redução do consumo de Intensidade Energética e Carbónica. Há uma forte preocupação da nossa parte em incorporar a análise energética nas aquisições de novos equipamentos e na construção/remodelação de edifícios e infraestruturas.
CEREALIS	F.C18	A ÁGUA A água é um importante fator de produção e os constrangimentos associados à sua escassez representam um risco para o negócio. As crescentes imposições legais à utilização deste recurso e a progressiva aplicação de custos reais no uso da água tem implicações na competitividade das empresas, o que motiva as organizações a fazerem uma gestão racional e eficiente do consumo de água e de minimização do impacto ambiental da sua utilização.
CEREALIS	F.C19	RESÍDUOS Os resíduos são uma fonte de poluição com impactos no ambiente. A nossa gestão de resíduos considera cada resíduo, não apenas como uma fonte de poluição a reduzir, mas também como um recurso potencial a explorar, por isso procuramos a prevenção da produção de resíduos e na promoção de uma reciclagem ou valorização eficaz.
CEREALIS	F.C20	RUÍDO AMBIENTAL O ruído é uma variável ambiental que requer toda a atenção da Cerealis, dada a sua importância nos mais diversos aspetos, nomeadamente associados à saúde humana e bem estar. Todos os projetos de ampliação ou remodelação de edifícios industriais e alteração de funcionamento de equipamentos mais críticos tiveram a preocupação acústica de modo a garantir a conformidade. As moagens de Lisboa e Porto e a fábrica e a semolaria da Maia, concluíram o licenciamento ambiental, aplicando o comprimento de todos os exigentes requisitos legais. Nos restantes centros não licenciados, devido á política de gestão assente num desenvolvimento sustentado, aplicamos as mesmas boas práticas. Emboras as nossas empresas não estejam certificadas ambientalmente, são aplicados os princípios de gestão de sistema ambientais definidos na ISO 14000.
CEREALIS	F.C21	SEGURANÇA E SAÚDE NO TRABALHO Para nós a segurança e saúde no trabalho é um pilar fundamental. Ao longo dos anos, temos vindo a investir fortemente na formação dos nossos colaboradores e na implementação de medidas de minimização dos riscos. Tendo em vista a redução da sinistralidade laboral, desenvolvemos o programa PREVENIR +. Com este programa pretendemos consciencializar e responsabilizar os colaboradores para a importância de práticas de trabalho seguras, através de equipes de delegados de prevenção dinamizadores das boas práticas e do levantamento de riscos potenciais.
CEREALIS	F.C22	OS MAIS RECENTES LANÇAMENTOS DO GRUPO CEREALIS A dinâmica de inovação no Grupo Cerealis e a eficácia distributiva no mercado interno e na exportação, são duas das alavancas essenciais para o crescimento do Grupo. Procurando surpreender os nossos consumidores e promover o negócio dos nossos clientes, a Cerealis lançou recentemente diversos produtos nas suas diferentes marcas e mercados.
CEREALIS	F.C23	- Estamos cientes da ética prevalente na empresa, sublinhando aqui as relações com os colaboradores. - Reconhecemos a cidadania da empresa e o seu envolvimento com as comunidades locais onde está inserida.
CEREALIS	F.C24	Na Cerealis enquadramos a sustentabilidade numa visão abrangente e holística do conceito, selecionando três pilares que interagem entre si: SOCIAL – ECONÓMICO – AMBIENTAL
CEREALIS	F.C25	Na Cerealis estamos atentos e comprometidos com as novas questões nutricionais da alimentação e impactos ambientais que a produção de alimentos implica.
CEREALIS	F.C26	É neste sentido que a Cerealis tem vindo a contribuir para o aumento da produção e consumo de cereais com origens em Portugal, tendo lançado uma gama de massas da Nacional exclusivamente com trigo duro alentejano.
CEREALIS	F.C27	É também neste âmbito que a Cerealis tem vindo a apostar na utilização de cacau, derivados, óleos e gorduras certificadas, aumentando significativamente, ano após ano, a % de utilização de cacau UTZ e óleos e gorduras RSPO. Na área dos transportes temos vindo a colocar as nossas energias na redução das emissões de F.CO2, tendo para isso contribuído o incremento das operações de backhauling com os nossos principais clientes.
CEREALIS	F.C28	Olhamos o futuro com otimismo, implementando um plano de investimento e de parcerias que visam o reforço da dignidade do trabalho e sustentabilidade económica do Grupo. Queremos continuar a crescer sustentadamente conscientes da responsabilidade que temos pelos impactos diretos e indiretos do que compramos (matérias-primas), e que produzimos (nossas marcas e produtos). Neste sentido, incorporamos planos de ação que procuram promover o consumo e a produção responsável.
CEREALIS	F.C29	Na Cerealis colocamos as nossas energias e conhecimento para que possamos fazer cada vez melhor. Fazer melhores produtos, fazer produtos nutricionalmente mais equilibrados, mais saborosos, industrialmente mais eficientes e que, naturalmente, respondam às tendências do mercado. Inovamos também para acrescentar valor. Acrescentar valor ao longo da cadeia de abastecimento, assim como valor nutricional nos produtos que desenvolvemos, tendo muito claro que só conseguimos crescer se inovarmos permanentemente.
CEREALIS	F.C30	Na Cerealis estamos atentos e comprometidos com as novas questões nutricionais da alimentação e impactos ambientais que a produção de alimentos implica. O facto de as nossas marcas serem marcas de referência, fazendo parte da preferência dos portugueses, apenas vem amplificar o desafio que temos e papel que somos chamados a cumprir: o de continuarmos a inovar para criar produtos que venham ao encontro do melhor conhecimento nutricional e das necessidades de uma sociedade muito diversa e dinâmica, que se quer saudável, obtidos através de uma cadeia de fornecimento que permita mitigar a pressão negativa que a produção dos alimentos coloca nos ecossistemas, com embalagens que garantam a segurança alimentar, com a menor pegada de carbono possível e, por fim mas não menos importante, recicláveis e garantidas as premissa da segurança alimentar, cada vez mais com componentes reciclados. Para isso, prestamos particular atenção a cinco grandes temas que marcam o setor agroalimentar e guiam a atuação da Cerealis:
CEREALIS	F.C31	Garantir a segurança alimentar e promover uma alimentação saudável são, desde sempre, premissas da nossa atuação, fazendo parte da nossa missão. O diálogo e parcerias com todas as partes interessadas Cada vez mais informados, os consumidores procuram produtos que respondam a esses critérios. Por isso, tem sido decisivo o acompanhamento regular das grandes tendências da sociedade com doses ajustadas de sobriedade e de intuição. Deste modo, acautelamos

		que o desenvolvimento dos nossos produtos e iniciativas tenham tanto de inovador como de sustentado. Através das nossas marcas mais representativas e do reconhecimento e confiança estabelecido com os consumidores, temos vindo a contribuir com novos produtos que, ano após ano vêm reforçando a sua relevância no mercado. As tendências que ditam o futuro da alimentação Os Objetivos do Desenvolvimento Sustentável que marcam a Agenda 2030 A importância de contarmos com os processos industriais mais avançados Nunca estes desígnios assumiram um carácter tão coletivo como agora. Para o alcançar existem duas palavras que se interligam: cooperação e inspiração. Consideramos que o contributo da Cerealis para a sociedade só poderá ser alavancado através da cooperação com todos os nossos parceiros. Os grandes desafios conquistam-se com a inspiração que nasce de muitas fontes. Desde logo com os nossos fornecedores, as associações, universidades e, claro, com os nossos clientes e consumidores, aos quais nos sentimos ligados pela preferência pelas nossas marcas, motivando-nos diariamente a criar e a apresentar as melhores soluções para a sua alimentação. 36 37 Como grupo de referência do setor alimentar, cabe-nos também a responsabilidade de usarmos todo o conhecimento que geramos através da nossa experiência, pesquisa e parcerias, na dinamização de uma cultura alimentar sustentável. Destacamos o nosso contributo para esse propósito, através das nossas marcas e de diversas atividades que ano após ano procuramos consolidar.
CEREALIS	F.C32	CRESCIMENTO SUSTENTÁVEL Inspirados em contribuir para uma sociedade mais equilibrada e sustentável Na Cerealis, enquadramos a sustentabilidade numa visão abrangente e holística do conceito, trabalhando transversalmente nos pilares Social, Económico e Ambiental. Para além das preocupações do foro nutricional, os temas de impacto ambiental são uma prioridade. A Cerealis contribui para os Objetivos do Desenvolvimento Sustentável da agenda 2030 das Nações Unidas, priorizando 4 dos 17 objetivos e implementando um conjunto de atividades relacionadas.
CEREALIS	F.C33	TRABALHO DECENTE E CRESCIMENTO ECONÓMICO Na Cerealis sabemos que tudo o que somos e tudo o que fazemos será a soma de pequenos grandes detalhes, a soma de muitas pessoas. Porque tudo conta, cada pessoa, cada ideia, porque … na Cerealis cada grão conta. Olhamos o futuro com otimismo, implementando um plano de investimento e de parcerias que visam o reforço da dignidade do trabalho e sustentabilidade económica do Grupo: • Permanente investimento na inovação e desenvolvimento tecnológico das unidades produtivas como prioridade estratégica, impulsionando o crescimento económico e a qualidade do emprego. • Qualificação dos colaboradores para os novos desafios da digitalização do sector. • Parcerias com escolas para formar estudantes para os desafios do mundo do trabalho.
CEREALIS	F.C34	CONSUMO E PRODUÇÃO RESPONSÁVEIS Queremos continuar a crescer sustentadamente conscientes da responsabilidade que temos pelos impactos diretos e indiretos do que compramos (matérias primas), e que produzimos (nossas marcas e produtos). Neste sentido, incorporamos planos de ação que procuram promover o consumo e a produção responsável: • Critérios de eficiência e redução de impactos ambientais em curso em todas as unidades industriais e ao longo da cadeia de valor, nomeadamente nos fluxos de transporte. • Divulgação ativa da dieta mediterrânica e a sua inclusão nas receitas das marcas. Iniciativa de apoio à produção local de cereais para estímulo da economia local e promoção da qualidade de alimentos 100% nacionais. • Incremento de comprar de matérias primas de produção sustentável (cacau e gorduras).
CEREALIS	F.C35	AÇÃO CONTRA A MUDANÇA GLOBAL DO CLIMA Na nossa atividade como produtor e embalador, dedicamos uma especial atenção à complexa, mas desafiadora gestão da temática das embalagens. Trabalhamos afincadamente no binómio da garantia da preservação da qualidade dos produtos que produzimos e embalamos e, na redução dos seus impactos ambientais. Procurando contribuir para o movimento contra a mudança global do clima, somos cada vez mais eficientes na gestão dos transportes que utilizamos para disponibilizar as nossas marcas e produtos aos nossos clientes e consumidores. Esta ação concertada implica a implementação de planos muito exigentes e ambiciosos para toda a Organização: • Dinamização do modelo de Backhauling em forte parceria com os nossos clientes, aumentando 43% a quota de custo deste modelo nos transportes tais em 2020. • Continuar a trabalhar com os nossos parceiros no sentido de reduzir significativamente os impactos ambientais diretos da não degradação natural de parte das suas embalagens. • Como membro do Pacto Português para os Plásticos, faremos parte de um movimento ativo que deverá garantir que: ◦ 100% das nossas embalagens de plástico serão recicláveis. ◦ 70%, ou mais, das embalagens plásticas serão efetivamente recicladas, através do aumento da recolha e da reciclagem. • Promoveremos atividades de sensibilização e educação aos consumidores (atuais e futuros) para a utilização circular dos plásticos.
CEREALIS	F.C36	O trigo testemunha a evolução da humanidade. Domesticado desde há 10.000 anos no Crescente Fértil e no Médio Oriente, o trigo tornou-se o sustento base das principais civilizações que primeiro emergiram na Mesopotâmia e no Egito. Conquistou a Europa 6.000 a. C. e fez-se agricultura dominante nas mãos do Império Romano. Chegou a ser moeda de troca entre agricultores e forma de pagamento de camponeses. Instigou guerras de poder, já que um celeiro cheio significava um governo forte. Quem dominasse a cultura do trigo garantia o crescimento da população. Por ser um excelente fornecedor de energia, o trigo permanece como um dos pilares da alimentação diária mundial, dando vida ao pão, às massas alimentícias e a milhares de outras criações culinárias. Esta importância dita a preocupação face às alterações do clima. Não só cada grão que nasce continua a ser um grão necessário, como muitos mais terão de nascer para alimentar uma população estimada em 10 mil milhões em 2050.
CEREALIS	F.C37	Atualmente, os cereais constituem a base de uma dieta saudável para grande parte da população mundial. São ricos em hidratos de carbono complexos e têm um teor naturalmente baixo em gordura. A par de outros alimentos incluídos na Dieta Mediterrânica, os cereais, especialmente os integrais, pela sua riqueza nutricional, têm vários benefícios. Tal como recomendado pela Organização Mundial de Saúde, privilegie os cereais integrais de pequeno-almoço sem adição de açúcar. Uma vez que não sofrem a moenda dos cereais refinados a sua percentagem de vitaminas, minerais e fibra é maior, conferindo vantagens ao nível da prevenção de determinadas doenças cardiovasculares, obesidade e diabetes.
CEREALIS	F.C38	A pirâmide da Dieta Mediterrânica é também um guia para a promoção de estilos de vida saudáveis, apresentando orientações para a população adulta saudável. As orientações alimentares dividem-se segundo a frequência: diária, semanal ou ocasional. Na base da pirâmide encontram-se os alimentos de origem vegetal, fornecedores de um conjunto alargado de nutrientes que auxiliam a regular e proteger o organismo. Nos patamares superiores encontram-se os alimentos que devem ser consumidos em menor quantidade e frequência. Cada grupo tem uma mensagem e, segundo este guia, os cereais devem constituir a principal fonte de energia durante o dia, devendo ser consumidos em todas as refeições principais (p.e. pão de mistura ou integral, massas de trigo duro, arroz, couscous ou milho).
CEREALIS	F.C39	4.1. Nutrição A alimentação é a base determinante para uma vida saudável, promovendo bem-estar e o equilíbrio essencial. Neste sentido, a maior preocupação por uma alimentação nutricionalmente equilibrada e saudável é cada vez mais um tema central para os consumidores e, consequentemente para a indústria alimentar. Apesar de ser uma temática transgeracional, todos os estudos demonstram uma maior consciência das gerações mais novas, incluindo-se neste universo a trilogia mágica da saúde e do bem-estar físico e emocional. Assim, a procura de alternativas mais saudáveis

		e convenientes, tem vindo a ganhar uma maior preponderância, destacando-se alguns movimentos com impactos significativos na indústria alimentar.

COCA-COLA

Website	Code	CPA EXAMPLES
COCA-COLA	F.CC1	Fazemos parte de uma empresa europeia, Coca-Cola Europacific Partners, mas investimos, fabricamos, distribuímos e geramos emprego localmente. Temos orgulho em trabalhar para sermos um agente de mudança social positiva nas nossas comunidades.
COCA-COLA	F.CC2	O nosso foco é o cliente e assim estão orientados os nossos mais de 23.000 colaboradores em toda a Europa, 400 deles em Portugal.
COCA-COLA	F.CC3	A nossa ambição: liderar o crescimento rentável e sustentável do sector das bebidas não alcoólicas na Europa Ocidental.
COCA-COLA	F.CC4	Cadeia de Valor Produção: 1 fábrica de produção, vendendo 211 milhões de litros em Portugal
COCA-COLA	F.CC5	Portugal faz parte da divisão ibérica da Coca-Cola Europacific Partners, com fábrica, investimento, distribuição e criação de emprego a nível local.
COCA-COLA	F.CC6	Made in Portugal Há mais de 40 anos a dinamizar a economia portuguesa
COCA-COLA	F.CC7	Mais de 40 anos depois, a fábrica é um exemplo de inovação e sustentabilidade, um importante dinamizador da economia portuguesa. Emprega no país mais de 400 pessoas e contribui para a criação de 5000 postos de trabalho indiretos. Das suas linhas de produção, saem mais de 250 milhões de litros de bebidas por ano, que refrescam residentes e turistas, num total de 37,3 milhões de consumidores em território nacional.
COCA-COLA	F.CC8	Estamos determinados a fazer uma diferença positiva na sociedade, promovendo o desenvolvimento económico nas nossas comunidades locais e reforçando a nossa pegada local. Muitas das nossas comunidades estão a enfrentar desafios sociais significativos, incluindo elevados níveis de desemprego jovem e situações de vulnerabilidade. Queremos dar um contributo positivo, apoiando várias associações locais e ajudando os jovens a obterem emprego, competência e confiança de que necessitam para serem bem-sucedidos. Também queremos ajudar as mulheres que queiram melhorar a sua empregabilidade ou empreender em novos desafios. Também ajudamos a enfrentar estes desafios nos territórios onde operamos, oferecendo aos nossos colaboradores a oportunidade de utilizar até dois dias de trabalho remunerado por ano para realizar atividades de voluntariado nas suas comunidades.
COCA-COLA	F.CC9	O sistema Coca-Cola é um negócio local, que acrescenta valor à economia portuguesa e gera oportunidades de emprego de qualidade.
COCA-COLA	F.CC10	UM TOTAL DE MAIS DE 12.500 EMPREGOS (0,25% DA FORÇA DE TRABALHO TOTAL) 12.200 + postos de trabalho indiretos. Por cada trabalho direto no sistema em Portugal há outros 35 gerados em outros lugares.
COCA-COLA	F.CC11	350 postos de trabalho diretos.
COCA-COLA	F.CC12	Por cada 1€ gasto pelos consumidores de bebidas Coca-Cola em Portugal 0,78 cêntimos revertem para a economia portuguesa.
COCA-COLA	F.CC13	118 M€ pagos a fornecedores portugueses (mais de 62% do total das despesas de 2022)
COCA-COLA	F.CC14	A atividade da Coca-Cola gerou um valor acrescentado de M€ 545 (0,2% do PIB)
COCA-COLA	F.CC15	O pagamento de impostos para atividades relacionadas com a Coca-Cola totalizaram M€ 287
COCA-COLA	F.CC16	+ 53 M€ no comércio grossista e retalhista. Tendo em consideração a inflação, o efeito em termos reais seria um aumento de 23% quando contabilizada a inflação de 8%, entre 2019 e 2022.
COCA-COLA	F.CC17	Os empregos gerados, direta ou indiretamente, pelo sistema em Portugal aumentaram 43%
COCA-COLA	F.CC18	Contribuímos para gerar valor nas comunidades onde operamos: mais de 17.000 eventos em Portugal, Espanha e Andorra
COCA-COLA	F.CC19	A nossa oferta de bebidas progride a cada ano que passa com novas variedades de modo a responder as necessidades atuais dos consumidores. Adaptamo-nos às tendências que promovem estilos de vida saudáveis.
COCA-COLA	F.CC20	Temos como objetivo colocar no mercado bebidas que correspondam às expetativas dos consumidores, de tal forma que possam encontrar a bebida que melhor se adapte ao seu estilo de vida para que cada pessoa possa fazer a melhor escolha, adaptada aos seus gostos e necessidades em cada momento e ocasião.
COCA-COLA	F.CC21	De acordo com as recomendações da Organização Mundial de Saúde (OMS), trabalhamos para ajudar o consumidor a não exceder a ingestão de, no máximo, 10% de calorias provenientes de açúcares livres por dia (adição de açúcares, xaropes, mel, sumos de fruta, etc). A este respeito, estamos a trabalhar com a The Coca-Cola Company e outros engarrafadores para oferecer aos consumidores uma variedade ainda maior de bebidas, que incluem mais opções de bebidas com baixo teor de açúcar ou sem açúcar. A nossa ação em matéria de bebidas ajuda-nos a apoiar o Objetivo de Desenvolvimento Sustentável 3 das Nações Unidas – Saúde e Bem-Estar, assim como a reforçar o objetivo 17 (Alianças para alcançar os objetivos) trabalhando em conjunto com os nossos partners e stakeholders.
COCA-COLA	F.CC22	Esforçamo-nos constantemente por oferecer os melhores produtos aos nossos compradores, com uma vasta gama de opções. Vamos continuar a desenvolver o mercado "light", que terá um crescimento exponencial: atualmente, todos os nossos produtos têm uma versão "0". E também, estamos a apostar em novos formatos como o mini, estamos a trabalhar para cobrir as ocasiões de consumo que estão a ganhar força, como o "on the go" ou a compra online.
COCA-COLA	F.CC23	Continuamos a fazer progressos na redução da quantidade de açúcar nas nossas bebidas, oferecendo uma gama mais vasta de opções. Queremos ajudar o consumidor a não exceder uma ingestão superior a 10% de calorias de açúcares livres por dia (adição de açúcar, xaropes, mel, sumos de frutas), desenvolvendo o nosso portefólio e facilitando decisões mais informadas sobre os seus hábitos de consumo através de etiquetas claras e completas.
COCA-COLA	F.CC24	Já superamos o nosso objetivo de 50% das nossas vendas provenientes de bebidas baixas em calorias ou sem calorias, alcançando 56% em 2019*. E também continuamos a aumentar o nosso investimento na comunicação para reforçar a sensibilização dos consumidores sobre a nossa gama de novas bebidas com baixo teor de açúcar.
COCA-COLA	F.CC25	Em 2019, 56% das nossas vendas foram provenientes de bebidas baixas em calorias ou sem calorias (6 em cada 10 bebidas que vendemos em Espanha foram baixas em calorias ou sem calorias). Atualmente, em Espanha já temos uma alternativa sem adição de açúcar no produtos que compõem o nosso portefólio.
COCA-COLA	F.CC26	Estamos a lançar novos formatos de embalagens, mais pequenos, que podem ajudar os consumidores a controlar a ingestão de calorias e de açúcar. Atualmente, 10% das nossas bebidas são vendidas em embalagens de 250 ml ou menos, e estamos a trabalhar para aumentar este número significativamente até 2025.

COCA-COLA	F.CC27	Queremos facilitar aos consumidores a redução do consumo de açúcar fornecendo informação clara e simples em todas as nossas embalagens, aumentando a quantidade de informação nas embalagens mais pequenas e informando sobre os ingredientes de todas as nossas bebidas. Para ajudar os consumidores a fazerem escolhas informadas, apoiamos esquemas de etiquetagem claros em todos os nossos produtos, em conformidade com a legislação da UE.
COCA-COLA	F.CC28	O nosso caminho para oferecer bebidas sem adição de açúcar começou em 1984 com o lançamento em Espanha da CocaCola sabor Light Sem Açúcar. Em 2019, e mais de 35 anos depois dessa data, já tínhamos quase 120 referências de produtos sem adição de açúcar, light ou zero em todas as nossas categorias de bebidas. Vamos continuar a trabalhar para oferecer sempre novas variedades sem calorias e continuaremos a liderar o mudança no mercado.
COCA-COLA	F.CC29	Garantimos que as nossas práticas comerciais e de marketing correspondem às expetativas dos nossos grupos de interesse e continuamos a manter a nossa política de não publicitar os nossos produtos a crianças com menos de 12 anos de idade. Temos uma política de marketing responsável de não publicitar nenhum dos nossos produtos a menores de 12 anos. Através da UNESDA, a associação europeia da indústria de refrigerantes, comprometemo-nos a não fazer publicidade na imprensa escrita, websites ou durante a emissão de programas especificamente dirigidos a crianças. Além disso, estamos empenhados na eliminação de todas as bebidas com adição de açúcar nas escolas secundárias em toda a Europa.
COCA-COLA	F.CC30	O nosso planeta, as nossas pessoas e as comunidades são muito importantes para nós Temos como objetivo criar um futuro melhor e mais sustentável para todos. Fazer a diferença na vida das pessoas, das comunidades e cuidar do planeta exercendo a nossa atividade da melhor forma. Tornando-nos uma empresa melhor, podemos construir um futuro mais forte e sustentável para todos.
COCA-COLA	F.CC31	As pessoas são importantes Acreditamos que um local de trabalho inclusivo, diverso e igualitário nos fortalece enquanto empresa, permite-nos criar um futuro melhor para colaboradores e comunidades, capacita o acesso à igualdade de oportunidades e gera um sentimento de inclusão nas nossas empresas e na sociedade em geral.
COCA-COLA	F.CC32	Esforçamo-nos para reduzir a água que utilizamos, reciclamo-la e devolvemo-la à natureza.
COCA-COLA	F.CC33	A Coca-Cola, em Portugal, na Espanha e na Europa, continuará a utilizar todo o seu potencial para liderar e ajudar a Companhia a alcançar os seus objetivos globais, com projetos pioneiros e de ponta que continuem a marcar a diferença, tanto nas comunidades locais, como no ambiente.
COCA-COLA	F.CC34	O nosso planeta, as nossas pessoas e as comunidades são muito importantes para nós Temos como objetivo criar um futuro melhor e mais sustentável para todos. Fazer a diferença na vida das pessoas, das comunidades e cuidar do planeta exercendo a nossa atividade da melhor forma. Tornando-nos uma empresa melhor, podemos construir um futuro mais forte e sustentável para todos. Estamos a assumir uma abordagem holística à sustentabilidade com foco na gestão social, ambiental e económica. Só através de uma abordagem abrangente podemos alcançar mudanças ousadas e duradouras.
COCA-COLA	F.CC35	A nossa estratégia de sustentabilidade guia a forma como apoiamos e interagimos com as pessoas que estão por trás das nossas marcas — dos agricultores e colaboradores aos fornecedores, parceiros de engarrafamento, consumidores, clientes e às nossas comunidades — e ajuda-nos a dar um contributo real para a proteção do meio ambiente.
COCA-COLA	F.CC36	Gestão dos recursos hídricos A estratégia para os recursos hídricos que seguimos foca-se na qualidade da água através do reabastecimento local (devolvendo a água às comunidades e à natureza), da promoção de políticas hídricas mais inteligentes e da utilização eficiente e responsável da água em todas as nossas operações e nas comunidades que servimos.
COCA-COLA	F.CC37	Embalagens sustentáveis Temos como meta recolher e reciclar uma garrafa ou lata por cada uma que vendemos, tornar 100% das nossas embalagens recicláveis e usar 50% de materiais reciclados nas nossas garrafas.
COCA-COLA	F.CC38	Os nossos produtos Temos uma vasta oferta de bebidas em várias categorias, porque pretendemos tornarnos uma Empresa Global de Bebidas, oferecendo inúmeras opções adaptadas às necessidades dos consumidores ao longo do dia, incluindo bebidas com menos açúcar e com benefícios nutricionais. Desde água com gás a bebidas lácteas e refrigerantes.
COCA-COLA	F.CC39	Acreditamos que um local de trabalho inclusivo, diverso e igualitário nos fortalece enquanto empresa, permite-nos criar um futuro melhor para colaboradores e comunidades, capacita o acesso à igualdade de oportunidades e gera um sentimento de inclusão nas nossas empresas e na sociedade em geral. Há muito que sabemos que a força da nossa empresa se deve às comunidades que servimos. Para além dos nossos colaboradores, trabalhamos para melhorar a vida dos consumidores, clientes e sociedades em todo o mundo.
COCA-COLA	F.CC40	Diversidade, paridade e inclusão A diversidade, paridade e inclusão estão no centro dos nossos valores e da nossa estratégia de crescimento e desempenham um papel importante (…)
COCA-COLA	F.CC41	Pessoas e comunidades Apoiamos as comunidades em que vivemos e tentamos sempre chegar mais longe, especialmente em tempos de necessidade.
COCA-COLA	F.CC42	Desporto e entretenimento Estabelecer uma ligação com os consumidores através de parcerias no desporto, música, jogos e filmes faz parte do ADN da Coca Cola Company.
COCA-COLA	F.CC43	Sustentabilidade Estamos a avançar no nosso compromisso de alcançar um mundo mais sustentável. Para liderar o caminho para uma economia circular, criando valor no nosso negócio, para os nossos clientes e para a sociedade, atuamos em 6 pilares: bebidas, embalagens, sociedade, água, clima e cadeia de fornecimento.
COCA-COLA	F.CC44	A sustentabilidade está no centro do nosso negócio. Orientados pela estratégia Avançamos, e em conjunto com os nossos colaboradores e parceiros, trabalhamos para fazer a diferença na sociedade e no planeta.
COCA-COLA	F.CC45	O nosso objetivo é alcançar o mais alto nível de excelência no cumprimento do nosso trabalho e chegar ao maior número de pontos de venda, graças à capilaridade do nosso modelo e uma execução de excelência diária baseada na segmentação. O foco no cliente e a ligação ao mercado são os nossos principais pilares: todos os processos, desde o fornecimento até à gestão industrial, são concebidos para melhorar continuamente o serviço e a chegada ao mercado.
COCA-COLA	F.CC46	Obtemos matérias-primas de muito fornecedores diferentes, com os quais mantemos um diálogo abe!o e constante. Em 2019, 90% já cumpriam os nossos Princípios Orientadores para Fornecedores e, no mesmo ano, a maioria das nossas despesas com fornecedores foram com as empresas locais. Acreditamos que as relações a longo prazo e a inovação em toda a nossa cadeia de fornecimento são essenciais para promover o nosso crescimento sustentável. Para estabelecer estas relações sustentáveis e duradouras, temos um modelo de compras baseado na confiança e no valor, seguindo os seguintes passos:
COCA-COLA	F.CC47	Queremos ser um agente de mudança positiva para as pessoas e para o planeta. Através da nossa estratégia de sustentabilidade para a Europa Ocidental, Avançamos, estamos a atuar nas áreas em que sabemos que podemos fazer a diferença e onde os nossos colaboradores e stakeholders estão mais envolvidos.

COCA-COLA	F.CC48	O nosso trabalho na cadeia de fornecimento ajuda a apoiar os seguintes Objetivos de Desenvolvimento Sustentável das Nações Unidas: 2 – Zero fome 8 – Trabalho decente e crescimento económico 10 – Redução das desigualdades 12 – Produçao e consumo responsáveis
COCA-COLA	F.CC49	O crescimento demográfico, o aumento da procura de produtos alimentares e as alterações climáticas estão a exercer uma maior pressão na nossa cadeia de fornecimento. Para produzir as nossas bebidas, dependemos de um fornecimento sustentável de matérias-primas e ingredientes agrícolas de alta qualidade. A nossa missão é assegurar que se obtêm de forma sustentável. Também temos o dever de respeitar e proteger os direitos humanos de todos os que trabalham ao longo da nossa cadeia de fornecimento. O nosso objetivo é assegurar que os nossos fornecedores respeitam o nosso Código de Conduta e tenham um impacto positivo na sociedade, de acordo com os Princípios Orientadores sobre Empresas e Direitos Humanos das Nações Unidas, la Declaração da Organização Internacional do Trabalho sobre os Princípios e Direitos Fundamentais no Trabalho e o Pacto Global das Nações Unidas.
COCA-COLA	F.CC50	A água é o principal ingrediente dos nossos produtos e é essencial para os nossos processos de produção. Por este motivo, promovemos a gestão sustentável da água ao longo de toda a nossa cadeia de valor.
COCA-COLA	F.CC51	Esforçamo-nos por manter um ambiente de trabalho seguro, diversificado e inclusivo, e oferecemos oportunidades de formação e desenvolvimento integral. A nível global, contamos com diversas iniciativas para acompanhar as mulheres no seu desenvolvimento profissional.
COCA-COLA	F.CC52	Queremos ser um agente de mudança positiva na sociedade e nas nossas comunidades.
COCA-COLA	F.CC53	A inclusão e a diversidade estão no coração da nossa estratégia. Estamos a promover uma cultura em que todos possamos assumir a nossa individualidade, sentirmo-nos valorizados e integrados. Promovemos a integração de vários talentos, incluindo femininos e intergeracionais, encorajamos a multiculturalidade, a integração de pessoas com deficiência e o foco na comunidade LGBTI+.
COCA-COLA	F.CC54	Queremos desenvolver uma cultura cujo núcleo seja a inclusão e a diversidade. Isto requer uma mudança real em toda a empresa. Estamos a progredir, mas ainda precisamos de fazer mais no nosso local de trabalho e nas nossas comunidades.
COCA-COLA	F.CC55	Oferecemos um ambiente de trabalho seguro e saudável, onde a diversidade é valorizada e onde as pessoas têm a oportunidade de ter sucesso, tanto a nível pessoal como profissional. Promovemos a estabilidade do emprego com políticas de remuneração e medidas de conciliação que favorecem o desempenho profissional de cada um de nossos funcionários.
COCA-COLA	F.CC56	Estamos determinados a fazer uma diferença positiva na sociedade, promovendo o desenvolvimento económico nas nossas comunidades locais e reforçando a nossa pegada local. Muitas das nossas comunidades estão a enfrentar desafios sociais significativos, incluindo elevados níveis de desemprego jovem e situações de vulnerabilidade. Queremos dar um contributo positivo, apoiando várias associações locais e ajudando os jovens a obterem emprego, competência e confiança de que necessitam para serem bem-sucedidos. Também queremos ajudar as mulheres que queiram melhorar a sua empregabilidade ou empreender em novos desafios. Também ajudamos a enfrentar estes desafios nos territórios onde operamos, oferecendo aos nossos colaboradores a oportunidade de utilizar até dois dias de trabalho remunerado por ano para realizar atividades de voluntariado nas suas comunidades.
COCA-COLA	F.CC57	O nosso negócio depende das pessoas extraordinárias que produzem, vendem e distribuem os nossos produtos todos os dias. O nosso sucesso só é possível com a paixão, o empenho e o talento dos mais de 400 colaboradores que trabalham todos os dias em Portugal, dando sempre o melhor de si. Acreditamos que a diversidade e a inclusão são fatores-chave para a inovação e o crescimento: a nossa visão é sermos uma organização de referência onde diferentes experiências e perspetivas de vida são valorizadas, e a promoção da igualdade de género é uma parte importante para o conseguir. Queremos que, pelo menos, 40% dos nossos cargos diretivos sejam ocupados por mulheres até 2025. Além disso, e para continuarmos a ser um excelente lugar para trabalhar, proporcionamos um ambiente de trabalho seguro e saudável.
COCA-COLA	F.CC58	Queremos fornecer opções de embalagens mais sustentáveis, para que os consumidores possam desfrutar das nossas bebidas. Estamos empenhados numa economia circular através da redução, reutilização e reciclagem de resíduos, com uma gestão das nossas embalagens que contribua para cuidar do nosso planeta. Um dos nossos maiores desafios é a gestão sustentável das nossas embalagens. Com o objetivo de liderar o caminho para uma economia circular, abordamos a gestão sustentável das nossas embalagens a partir de três abordagens principais: reduzir, reutilizar e recuperar. Trabalhamos para utilizar menos material nos nossos processos de produção. Em relação às embalagens que utilizamos, queremos recolher e reciclar todas para que nenhuma acabe como resíduo, especialmente nos oceanos. A nossa ação sobre as embalagens responde aos seguintes Objetivos de Desenvolvimento Sustentável das Nações Unidas (ODS): 8 - Trabalho e crescimento económico 9 - Indústria, inovação e insfraestructura 12 - Produção e consumo responsáveis 14 - Vida subaquática 15 - Vida dos ecossistemas terrestres 17 - Alianças para alcançar os objetivos
COCA-COLA	F.CC59	A água é o principal ingrediente dos nossos produtos e é essencial para o nosso processo de produção. Para assegurar o fornecimento de água sustentável, estamos a trabalhar para reduzir a quantidade que utilizamos nas nossas operações e a proteger as fontes de água locais para as gerações futuras. Na Coca-Cola European Partners dependemos de um fornecimento de água sustentável. No entanto, a deterioração da qualidade da água na nossa cadeia de fornecimento e a escassez causada pela exploração excessiva, a má gestão e os impactos das alterações climáticas tornaram-se grandes problemas para o nosso negócio. Para enfrentar estes desafios e cuidar dos nossos recursos hídricos, promovemos a gestão sustentável de água ao longo de toda a nossa cadeia de valor. As nossas ações em relação à água ajudam-nos a apoiar os seguintes Objetivos de Desenvolvimento Sustentável das Nações Unidas: 6 – Água limpa e saneamento 11 – Cidades e comunidades sustentáveis 15 – Vida dos ecossistemas terrestres 17 – Alianças para alcançar os objetivos.
COCA-COLA	F.CC60	O nosso objetivo é tornar as nossas fábricas de produção tão eficientes quanto possível em termos da sua gestão de água. Reduzimos a água que utilizamos na produção das nossas bebidas, realizando várias medições e desenvolvendo projetos que permitem otimizar o uso da água em todos os nossos processos, incluindo o tratamento das água, limpeza, etc.
COCA-COLA	F.CC61	Na Europa, os consumidores continuam a classificar as alterações climáticas como o principal desafio de sustentabilidade. Estamos a reduzir as nossas emissões para sermos pa!e da solução. Estamos em desempenhar um papel de liderança na abordagem da crise climática, reduzindo as emissões de carbono ao longo da nossa cadeia de valor, na sequência do Acordo de Paris sobre as alterações climáticas de 2015 e juntando-nos aos esforços que estão a ser feitos a nível mundial. Para conseguirmos, reduziremos a quantidade de energia que utilizamos nas nossas próprias atividades e encorajaremos os nossos fornecedores a adotarem iniciativas de redução de carbono. Desde 2010, temos em vigor um ambicioso programa de descarbonização para reduzir as emissões e fazemos melhorias contínuas para reduzir o impacto climático da nossa atividade. Os nossos objetivos de emissão de GEE (Gases com Efeito de Estufa) são apoiados pela ciência e foram validados pela iniciativa Science Based Targets (SBTi). Somos membros da iniciativa

		F.RE100 de The Climate Group e do Corporate Leaders Group. As nossas ações sobre o clima ajudam-nos a apoiar os seguintes Objetivos de Desenvolvimento Sustentável das Nações Unidas: 7 – Energia acessível e não poluente 12 – Produção e consumo responsáveis 13 – Ação pelo clima
COCA-COLA	F.CC62	Para reduzir a pegada de carbono das nossas fábricas e armazéns, concentramo-nos principalmente na redução da quantidade de energia que utilizamos. Trabalhando com os nossos partners da logística, estamos a melhorar as rotas em todos os nossos territórios para reduzir as distâncias que percorremos. Temos equipamentos de refrigeração mais eficientes, adquirindo dispositivos com maior eficiência energética e instalando dispositivos de poupança de energia. As nossas atividades de produção estão também a tornar-se mais eficientes, uma vez que implementámos projetos de iluminação inteligente (graças ao uso de LEDs nas fábricas e nos armazéns), instalámos revestimentos cerâmicos, lâmpadas economizadoras de energia e moldes de baixa pressão nos ventiladores
COCA-COLA	F.CC63	O crescimento demográfico, o aumento da procura de produtos alimentares e as alterações climáticas estão a exercer uma maior pressão na nossa cadeia de fornecimento. Para produzir as nossas bebidas, dependemos de um fornecimento sustentável de matérias-primas e ingredientes agrícolas de alta qualidade. A nossa missão é assegurar que se obtêm de forma sustentável. Também temos o dever de respeitar e proteger os direitos humanos de todos os que trabalham ao longo da nossa cadeia de fornecimento. O nosso objetivo é assegurar que os nossos fornecedores respeitam o nosso Código de Conduta e tenham um impacto positivo na sociedade, de acordo com os Princípios Orientadores sobre Empresas e Direitos Humanos das Nações Unidas, la Declaração da Organização Internacional do Trabalho sobre os Princípios e Direitos Fundamentais no Trabalho e o Pacto Global das Nações Unidas. O nosso trabalho na cadeia de fornecimento ajuda a apoiar os seguintes Objetivos de Desenvolvimento Sustentável das Nações Unidas: 2 – Zero fome 8 – Trabalho decente e crescimento económico 10 – Reduçao das desigualdades 12 – Produçao e consumo responsáveis

DANONE

Website	Code	CPA EXAMPLES
DANONE	F.D1	A Danone está em constante investigação e desenvolvimento de novos produtos, formatos e fórmulas que, de forma diferenciada, permitem à Danone introduzir, constantemente, inovações pioneiras no segmento dos produtos lácteos frescos, colocando sempre, o consumidor no centro das suas decisões. Para tal, temos uma equipa de Inovação e Desenvolvimento (I&D) de novos produtos. Esta equipa tem como principal objetivo entender as necessidades específicas do consumidor e materializá-las em produtos adaptados às suas reais expectativas.
DANONE	F.D2	O Palaiseau Danone Research Daniel Carasso Centre, também conhecido por Danone Research, situa-se em Palaiseau, a sul de Paris, e coordena as atividades de Investigação & Desenvolvimento do Grupo Danone para os produtos lácteos frescos. Inaugurado em 2002, reúne as competências de pesquisa do Grupo e é considerado uma referência e o catalisador da inovação para as várias marcas de produtos lácteos frescos da Danone procurando melhorar, diariamente, a saúde e bem-estar dos consumidores. A Investigação & Desenvolvimento, que integra a estratégia do Grupo Danone e ocupa um papel central no seu modelo de desenvolvimento, assenta em quatro eixos e em áreas de investigação de reconhecido know how. Os quatro eixos da Investigação & Desenvolvimento são: Nutrição, Saúde ativa, Prazer e Acessibilidade dos produtos. As suas áreas de investigação são: Investigação das funcionalidades e benefícios de matérias-primas para a saúde; Fermentos e probióticos; Ciências da nutrição; Conceção de novas fórmulas de produtos; Demonstração científica das alegações nutricionais e de saúde; Estudo das necessidades do consumidor; Análise sensorial. A procura constante de parcerias com os melhores especialistas do mundo permite associar a pesquisa interna com a pesquisa externa, potenciando todo o saber e a evidência científica cada vez mais importante e exigida pelo mercado.
DANONE	F.D3	Comer um iogurte por dia pode ser um delicioso hábito para uma alimentação saudável. Iogurtes cremosos, líquidos, com frutas e de vários sabores. Há sempre um Danone perfeito para cada ocasião.
DANONE	F.D4	Com uma barriga saudável, tudo tem mais flow! Por isso, Activia desafia-te a regressares à tua rotina com o ritmo certo. Porque nenhum outro ajuda mais a tua saúde digestiva*, descobre o poder da barriga no bem-estar e na saúde com o novo Activia Maçã, Aveia e Canela.
DANONE	F.D5	Actimel desafia portugueses a prepararem-se para o Inverno Numa altura em que é especialmente importante ter o sistema imunitário a funcionar corretamente, a Danone apresenta o novo Actimel Vitamina C, a nova proposta da marca para cuidar das defesas
DANONE	F.D6	Depois de um ano e meio de pandemia, em que as famílias portuguesas foram obrigadas a resguardar-se e a abdicar da sua liberdade, agora é o momento de regressar à rotina, sair à rua e partilhar espaços e experiências. Num desconfinamento que ocorre em pleno inverno, uma altura em que naturalmente as nossas defesas são desafiadas, é por isso, e como sempre, fundamental ter o sistema imunitário a funcionar corretamente. Deste modo, também no inverno é importante fazermos uma alimentação variada e equilibrada e termos um estilo de vida saudável, que nos ajude a cuidar das defesas do organismo e a estarmos assim preparados para enfrentar os dias desafiantes que temos pela frente. Neste contexto, a Danone apresenta o novo Actimel Vitamina C, a nova proposta da marca para cuidar das defesas – um leite fermentado que associa as bactérias tradicionais do iogurte ao seu fermento lático L. casei, específico e exclusivo. Para além disso, Actimel Vitamina C, é rico em vitamina C e contém as vitaminas D e F.B6 e o mineral zinco, que ajudam ao normal funcionamento do sistema imunitário!
DANONE	F.D7	Regressa a uma rotina saudável A saúde começa por dentro é a nova proposta de Activia, um movimento que reforça a ideia de que o bem-estar começa por dentro e que muitas vezes negligenciamos o nosso sistema digestivo, que desempenha um papel tão importante para nos sentirmos bem.
DANONE	F.D8	(*) Pessoas com o colesterol elevado devem consultar um médico. Danacol contém esteróis vegetais. Foi demonstrado que os esteróis vegetais reduzem o colesterol no sangue. O colesterol elevado é um factor de risco no desenvolvimento de doenças coronárias. Estas têm múltiplos factores de risco e alterar um desses factores pode, ou não, ter efeitos benéficos. A redução de colesterol é obtida com uma dose diária de 1,5g a 3g de esteróis vegetais. Cada Danacol contém 1,6g de esteróis vegetais. O consumo diário de 1,5g a 2,4g de esteróis vegetais reduz o colesterol LDL de 7% a 10% em 2 a 3 semanas. Deverá ser consumido 1 Danacol por dia dentro de uma alimentação variada e equilibrada e de um modo de vida saudável. O produto pode não ser nutricionalmente adequado para grávidas ou lactantes e menores de 5 anos. Danacol é um alimento, não é um medicamento. Consulte toda a informação na embalagem.
DANONE	F.D9	Ajudar o maior número de pessoas a construir, manter e proteger a sua saúde através da alimentação é o nosso maior objetivo. Temos de começar por produtos de gosto atrativo, de qualidade e segurança superior. Depois, produtos e serviços que ofereçam escolhas saudáveis com a maior densidade nutricional possível. Finalmente, soluções que sirvam

		para um grande número de comunidades, indo de encontro às suas necessidades locais, reforçando a vertente social e cultural.
DANONE	F.D10	A convicção de que a saúde e o bem-estar dependem da alimentação e nutrição é a principal fonte de inspiração da atividade do Grupo Danone. Com efeito, ainda não há muitos anos a nutrição e alimentação eram vistas como preocupações de ordem pessoal, mas a progressão de doenças relacionadas com a falta ou o excesso de consumo de alimentos e nutrientes transformou o que era encarado como uma questão individual num problema da sociedade moderna. A atenção dedicada à comunidade, através de constantes estudos do consumidor ou da troca de informações com profissionais de educação, de saúde e comunicação, revela não só uma atitude de abertura ao exterior, como esse desejo de corresponder cada vez melhor às necessidades do mercado de produtos alimentares. Com o objetivo de dar resposta às crescentes expectativas da sociedade relativamente ao papel da indústria alimentar na promoção da saúde pública, o Grupo Danone concebeu em 2005 a Carta da Alimentação, Nutrição e Saúde. Esta baseia-se em cinco convicções e em nove compromissos, que não são mais do que o documento base que inspira cada uma das empresas que compõem o Grupo, entre as quais a Danone Portugal.
DANONE	F.D11	Como empresa alimentar acreditamos que desempenhamos um papel fundamental na conquista por um impacto positivo na saúde pública e nas questões nutricionais, através dos nossos produtos. Ao comunicar os nossos produtos e serviços, acreditamos que podemos ajudar a educar e sensibilizar todos os consumidores, que confiam em nós diariamente, para a importância de uma dieta e estilo de vida saudáveis. É por isso que na Danone estamos empenhados em adotar uma publicidade responsável de acordo com o Código de Comunicação de Marketing Responsável de Alimentos e Bebidas da Câmara Internacional de Comércio e a legislação nacional. O nosso compromisso de marketing responsável engloba todas as comunicações que fazemos da Danone, especialmente em todos os materiais para comunicar e promover as nossas marcas ou produtos aos consumidores. Para todos eles, estamos empenhados em garantir que sejam legais, honestos e verdadeiros, utilizando apenas alegações que tenham uma base científica sólida por detrás delas: Fornecemos dados nutricionais comprovados que podem permitir aos consumidores e profissionais tomar decisões informadas. Damos especial ênfase à informação prática e fácil de usar para ajudar os consumidores a desenvolverem dietas saudáveis para si próprios e para as suas famílias. Asseguramos que toda a informação sobre os benefícios dos nossos produtos é fiável e numa língua que os consumidores possam compreender.
DANONE	F.D12	Cuidar dos nossos trabalhadores, do seu bem estar e desenvolvimento, fomentando a inclusão, igualdade de oportunidades e justa atribuição de benefícios. O que é uma empresa B Corporation A missão da Danone é levar saúde através da alimentação ao maior número de famílias possível. Dado que nem todas têm condições para ter uma boa alimentação, é nossa responsabilidade tentar ajudar estas famílias mais carenciadas Saiba mais Comunidade Conhecer a comunidade onde nos inserimos, perceber as suas particularidades e carências, para criarmos impacto positivo na mesma. Gestão Enaltecer a natureza do nosso negócio, a nossa missão, valores e ética, num modelo de empresa responsável para com as pessoas e o planeta. Ambiente Trabalhar para que o nosso impacto no meio ambiente seja o menor possível e adotar medidas que possam acelerar e garantir a sua proteção. Clientes Desenvolver uma relação de parceria com produtores, fornecedores e outras relações locais, de longa duração e proximidade. Cuidar e servir da melhor forma os nossos consumidores.
DANONE	F.D13	Ao longo dos últimos 30 anos no mercado português, a Danone deu importantes passos para acompanhar os gostos e preferências dos consumidores portugueses, mas também, fazendo escolhas e opções conscientes, que vão ao encontro de um futuro mais sustentável e de uma preocupação constante e crescente de escolhas ambientais.
DANONE	F.D14	Para concretizar a sua missão, a Danone segue cinco compromissos fundamentais que refletem o seu papel na sociedade: Oferecer produtos que respondem a necessidades e recomendações nutricionais, e gostos; Desenvolver produtos com benefícios cientificamente relevantes, que responda necessidades nutricionais reais; Dar aos consumidores informação clara e praticar uma comunicação responsável; Promover estilos de vida e de alimentação saudáveis; Responder aos maiores desafios de saúde e nutrição.
DANONE	F.D15	Alto teor de vitaminas C e D e fonte de vitamina F.B6, cálcio e zinco para um normal funcionamento do sistema imunitário.
DANONE	F.D16	Novas sobremesas proteicas: Pudins e Mousses. As recompensas que os Real Pros merecem, sem comprometer os resultados já alcançados!
DANONE	F.D17	Actimel tem alto teor de vitamina D e fonte de F.B9, ferro, zinco, que ajudam ao funcionamento do sistema imunitário. Deve ser ingerido numa alimentação e estilo de vida saudável.
DANONE	F.D18	O seu Actimel, agora reforçado com Vitamina C O novo Actimel Vita C contém 1/3 da dose diária recomendada de Vitamina C e 30% da dose diária recomendada de Vitamina D.
DANONE	F.D19	Nova gama Actimel menos açúcares Apresentamos os novos Actimel, com menos açúcar e mais fruta, mas com o benefício de sempre para o sistema imunitário.
DANONE	F.D20	Ajuda as tuas defesas com Actimel Ajuda as tuas defesas com Actimel, agora com uma nova fórmula com alto teor em Vitamina D, além de Vitamina F.B9, Ferro e Zinco, que ajudam ao normal funcionamento do teu sistema imunitário.
DANONE	F.D21	NOVA FÓRMULA TRIPLO ZERO, SEM AÇÚCARES ADICIONADOS, LACTOSE E MATÉRIA GORDA ESTE VERÃO, CUIDAR DA TUA SAÚDE DIGESTIVA É MAIS LEVE
DANONE	F.D22	Depois de apresentar a sua nova gama de iogurtes ricos em proteína, Corpos Danone reforça a oferta com dois novos sabores - Corpos Danone +Proteína Líquido de Manga e Chia e Sólido de Maracujá e Chia. Mantendo o compromisso de apresentar produtos baixos em gordura e sem açúcares adicionados (contêm apenas açúcares naturalmente presentes), os dois novos sabores combinam fruta e chia - as sementes das novas tendências que dão um outro nível à textura do produto. A marca, que apresentou este Verão uma imagem totalmente renovada, defende que cada corpo pode ser impressionante não pela forma como é, mas por aquilo que faz quando se sente bem e saudável. Com combinações de sabores entusiasmantes, o benefício da proteína presente e produtos baixos em gordura, esta gama pode ser o aliado perfeito para o seu dia agitado. Um corpo saudável faz coisas impressionantes. E Corpos Danone* continua a ajudar-nos a cuidar dele, como parte de uma alimentação saudável. *Corpos Danone +Proteína é rico em proteínas que contribuem para o crescimento e manutenção da massa muscular. Deverá ser consumidor integrado no âmbito de uma alimentação saudável e de um estilo de vida saudável.
DANONE	F.D23	Os esteróis vegetais presentes em Danacol reduzem o seu colesterol de forma 100% natural. Estes ajudam a bloquear parcialmente a absorção de colesterol de modo a impedir que parte chegue ao sangue, ajudando-o a reduzir até 10%. Danacol deverá ser consumido integrado numa alimentação variada e equilibrada e num modo de vida saudável.
DANONE	F.D24	A marca Danone surgiu há 100 anos quando um pai criou um alimento simples, nutritivo e natural para ajudar o seu filho a ter uma alimentação mais saudável: o iogurte. Hoje já são milhares de famílias confiam na Danone para lhes

		providenciar essa mesma alimentação saudável, para ajudar a cuidar de si e dos seus. O nosso iogurte parte de uma receita simples – leite e fermentos naturais – aos quais acrescentamos os melhores ingredientes para gerar uma diversidade de sabores e texturas que tornam o nosso iogurte num prazer natural.
DANONE	F.D25	Reforçamos o nosso compromisso com a nutrição Depois das celebrações dos 100 anos, em 2019, a marca Danone entra numa nova fase em que procura reforçar o seu compromisso em levar saúde através da alimentação ao maior número de famílias portuguesas.
DANONE	F.D26	Sem açúcar / 70% de proteína whey / sem lactose.
DANONE	F.D27	Em Portugal, segundo dados da Fundação Portuguesa de Cardiologia, mais de 58% da população desconhece os seus níveis de colesterol. Por isso, na data em que se assinala o Dia Mundial do Coração, Danacol (re)lembra que é urgente adotar um estilo de vida saudável, estar atento a fatores de risco que possam potenciar doenças cardiovasculares e incluir na nossa dieta, opões alimentares adequadas.
DANONE	F.D28	Ideal para os pequeninos comerem sozinhos sem se sujarem demasiado, Danonino apresenta saborosas gamas de iogurtes líquidos, polpas e queijinhos nas variedades que mais gostam, com fórmulas adicionadas de cálcio e vitamina D e ainda em embalagens à sua medida e com divertidas coleções para que possam desenvolver a sua imaginação. Danonino é um alimento adaptado às necessidades nutricionais dos teus filhos, sendo fonte de proteínas, cálcio e vitamina D, necessários para o crescimento e desenvolvimento normais dos ossos das crianças.
DANONE	F.D29	Agora, os mais pequenos podem juntar-se aos seus heróis de quatro patas preferidos e fazer parte das suas missões mais importantes! Deliciem-se com estes Danoninos com cálcio, Vitamina D e de classificação B no Nutri-Score - ideal para um lanche de heróis – e estarão prontos para salvar o dia!
DANONE	F.D30	Reforçamos o nosso compromisso com a nutrição
DANONE	F.D31	Depois das celebrações dos 100 anos, em 2019, a marca Danone entra numa nova fase em que procura reforçar o seu compromisso em levar saúde através da alimentação ao maior número de famílias portuguesas.
DANONE	F.D32	(…) concretizar a sua missão, a Danone segue cinco compromissos fundamentais que refletem o seu papel na sociedade: Oferecer produtos que respondem a necessidades e recomendações nutricionais, e gostos; Desenvolver produtos com benefícios cientificamente relevantes, que responda necessidades nutricionais reais; Dar aos consumidores informação clara e praticar uma comunicação responsável; Promover estilos de vida e de alimentação saudáveis; Responder aos maiores desafios de saúde e nutrição.

DELTA

Website	Code	CPA EXAMPLES
DELTA	F.DE1	GLOBALMENTE PORTUGUÊS Desde o início, o fundador da Delta Cafés teve a preocupação em desenvolver uma marca com projeção mundial, preparada para a conquista de novos consumidores, com uma visão clara – estar no top 10 das marcas de café mundiais.
DELTA	F.DE2	Pretendemos continuar a ter um papel ativo na construção de valor para a sociedade, contribuindo para a adoção de comportamentos mais responsáveis, acrescentando simultaneamente valor aos vários momentos de consumo e de partilha proporcionados pelo café. Um caminho de sustentabilidade é por isso prioritário para o Grupo e para todas as suas marcas. A sustentabilidade está no nosso ADN e trabalhamos continuamente para a conciliação entre o modelo de crescimento económico e o modelo de sustentabilidade de todas as nossas empresas de forma a assegurar o desenvolvimento sustentado do nosso negócio.
DELTA	F.DE3	Pretendemos continuar a ter um papel ativo na construção de valor para a sociedade, contribuindo para a adoção de comportamentos mais responsáveis, acrescentando simultaneamente valor aos vários momentos de consumo e de partilha proporcionados pelo café. Um caminho de sustentabilidade é por isso prioritário para o Grupo e para todas as suas marcas. A sustentabilidade está no nosso ADN e trabalhamos continuamente para a conciliação entre o modelo de crescimento económico e o modelo de sustentabilidade de todas as nossas empresas de forma a assegurar o desenvolvimento sustentado do nosso negócio.
DELTA	F.DE4	Este sentido de comunidade dentro do Grupo Nabeiro é algo intrínseco para todos os nossos colaboradores, clientes e parceiros, por isso: Priorizamos a preservação das origens assegurando a qualidade de vida da população que depende da cultura do café; Apoiamos e incentivamos o empreendedorismo assim como projetos com impactos transformacionais nas comunidades onde estamos.
DELTA	F.DE5	Divergir é correr riscos, avançar em direção ao falhanço ou ao sucesso, sabendo que em ambos os caminhos há algo de valor para trazer de volta a casa. A nossa cultura foi fundada na audácia, enraizada no nosso ADN e inspirada pelo longo e sólido percurso do Grupo Nabeiro. Uma cultura que acredita na geração de Propriedade Intelectual e na Inovação como uma responsabilidade partilhada, uma procura de futuro, sempre presente na mente de cada membro da nossa equipa.
DELTA	F.DE6	Das atividades de investigação, à vigilância tecnológica e de mercado, do insight à ideia, do protótipo à industrialização do produto, esta equipa conta com todas as valências para o desenvolvimento de novos produtos
DELTA	F.DE7	O NOSSO COMPROMISSO O nosso contínuo compromisso com a sustentabilidade é uma manifestação viva dos valores do nosso fundador. Continuar a assegurar a nossa rentabilidade económica, reduzindo o impacto ambiental e maximizando o impacto social positivo, é a nossa maior determinação. Será este o nosso futuro, materializado pela nossa estratégia de sustentabilidade global, desenhada em torno da nossa contribuição para os Objetivos de Desenvolvimento Sustentável (ODS).
DELTA	F.DE8	Acreditamos que uma visão, a longo prazo, centrada na valorização das pessoas, orientada pela sustentabilidade e inovação no nosso negócio, reforçará a nossa liderança no mundo.
DELTA	F.DE9	Corresponder às expectativas dos nossos consumidores assegurando a total satisfação dos nossos clientes, através de um modelo de negócio assente na criação e partilha de valor sustentável. Uma gestão de rosto humano é um compromisso fundamental, de forma a cumpri-lo encaramos o futuro com responsabilidade e respeito pelas próximas gerações.
DELTA	F.DE10	Acreditamos nas pessoas, e é por elas que damos diariamente o nosso melhor. A relação de proximidade que nos une, traduz-se no conhecimento das necessidades e motivações dos nossos consumidores, clientes e parceiros.
DELTA	F.DE11	PRETENDEMOS ASSIM: ASSEGURAR A NOSSA RENTABILIDADE ECONÓMICA, REDUZINDO O IMPACTO AMBIENTAL E MAXIMIZANDO O IMPACTO SOCIAL POSITIVO CONTRIBUIR CONTINUAMENTE COM IDEIAS E SOLUÇÕES DE FORMA A ALCANÇAR OS OBJETIVOS DE

		DESENVOLVIMENTO SUSTENTÁVEL REFORÇAR A SUSTENTABILIDADE DAS COMUNIDADES PRODUTORAS DE CAFÉ PROMOVER ATIVAMENTE A SUSTENTABILIDADE NOS NOSSOS PROJETOS, PROCESSOS E INFRAESTRUTURAS CAPACITAR AS PRÓXIMAS GERAÇÕES, VALORIZANDO O SEU PAPEL COMO CONSTRUTORAS DO AMANHÃ
DELTA	F.DE12	Este sentido de comunidade dentro do Grupo Nabeiro é algo intrínseco para todos os nossos colaboradores, clientes e parceiros, por isso: Priorizamos a preservação das origens assegurando a qualidade de vida da população que depende da cultura do café; Apoiamos e incentivamos o empreendedorismo assim como projetos com impactos transformacionais nas comunidades onde estamos.
DELTA	F.DE13	A estratégia de desenvolvimento do nosso negócio depende de uma equipa formada por pessoas com as competências necessárias e com a motivação para nos fazer crescer. De forma estruturada, investimos no recrutamento de pessoas qualificadas e na capacitação dos nossos colaboradores. A sua valorização e bem-estar pessoal é uma prioridade para nós e é por isso que a gestão de rosto humano continua a ser um dos fatores distintivos da nossa cultura empresarial.
DELTA	F.DE14	SER UM EMPREGADOR DE REFERÊNCIA, CONTRIBUINDO PARA A CRIAÇÃO DE EMPREGO E UM AMBIENTE DE TRABALHO POSITIVO O nosso contributo: Temos uma cultura de proximidade e de valorização de talento.
DELTA	F.DE15	A escassez de recursos naturais, conjugada com o crescimento populacional global, está a ditar a substituição de modelos produtivos lineares por modelos centrados na circularidade dos materiais. Estamos comprometidos com este novo paradigma e emprenhados na identificação de oportunidades que nos permitam aumentar a circularidade dos materiais presentes na cadeia de valor.
DELTA	F.DE16	O CAFÉ AO GOSTO DOS PORTUGUESES A relação com cada cliente é única. Disponibilizamos diferentes soluções, de forma a satisfazer as necessidades dos nossos clientes e a proporcionar um café Delta ao gosto dos portugueses .
DELTA	F.DE17	Tudo começou no Alentejo, na fronteira com Espanha. Em 1961, numa terra chamada Campo Maior, dentro de um pequeno armazém de 50m2, Rui Nabeiro começou o seu negócio com a torra de 30 kilos de café por dia. E assim, cria a marca de café mais conhecida dos portugueses, a Delta Cafés. 20 anos passados, é inaugurada a maior Torrefação da Península Ibérica e, durante as décadas seguintes, com o crescimento sustentado do negócio, vão surgindo as várias empresas que integram hoje o Grupo Nabeiro.
DELTA	F.DE18	ASSEGURAR A NOSSA RENTABILIDADE ECONÓMICA, REDUZINDO O IMPACTO AMBIENTAL E MAXIMIZANDO O IMPACTO SOCIAL POSITIVO CONTRIBUIR CONTINUAMENTE COM IDEIAS E SOLUÇÕES DE FORMA A ALCANÇAR OS OBJETIVOS DE DESENVOLVIMENTO SUSTENTÁVEL REFORÇAR A SUSTENTABILIDADE DAS COMUNIDADES PRODUTORAS DE CAFÉ PROMOVER ATIVAMENTE A SUSTENTABILIDADE NOS NOSSOS PROJETOS, PROCESSOS E INFRAESTRUTURAS CAPACITAR AS PRÓXIMAS GERAÇÕES, VALORIZANDO O SEU PAPEL COMO CONSTRUTORAS DO AMANHÃ

FERRERO

Website	Code	CPA EXAMPLES
FERRERO	F.F1	Ferrero começou a sua história na pequena cidade de Alba, no Piemonte, Itália, em 1946. Hoje, com uma faturação consolidada superior a 12,3 mil milhões de euros, a Ferrero encontra-se entre os líderes de mercado em alimentos embalados doces em todo o mundo. O Grupo Ferrero está presente em todo o mundo com mais de 37.000 pessoas e 31 unidades de produção. Ferrero é o produtor de muitas marcas icónicas que são amadas geração após geração, incluindo Nutella, Ferrero Rocher, Tic Tac, Kinder e Raffaello, e que são comercializadas em mais de 170 países
FERRERO	F.F2	O Grupo Ferrero está presente na Península Ibérica desde 1988. A Ferrero Ibérica tem uma faturação anual de 208 milhões de euros e vendas de 19 000 toneladas de produto. O quadro é formado por 162 pessoas, entre Espanha e Portugal
FERRERO	F.F3	A Fundação Ferrero está a tornar-se cada vez mais ativa a nível cultural, trabalhando com outras entidades locais, nacionais e internacionais, apoiando diversas ações duradouras concebidas para proporcionarem vantagens para toda a sociedade.
FERRERO	F.F4	No contexto global de atenção crescente a tópicos como nutrição e actividade física, a nossa estratégia de pesquisa e investimentos produtivos são focados na criação de produtos de alta qualidade, cuidadosamente desenvolvidos em termos de valor nutricional e porções, para que possam ser integrados em uma dieta equilibrada, com atenção especial às necessidades das crianças e das famílias
FERRERO	F.F5	O aumento da preocupação a nível mundial com a alimentação, a nutrição e a atividade física tem um enorme peso para a comunidade internacional dos produtos alimentares e das bebidas, da qual a Ferrero faz parte. Uma comunicação comercial responsável pode ajudar os consumidores a fazerem escolhas adequadas sobre os produtos alimentares que compram e consomem, dentro de um estilo de vida saudável e ativo. Ao enviar mensagens comerciais que apoiem os princípios de nutrição adequada, alimentação equilibrada, atividade física e escolha pessoal, o setor poderá assumir um papel importante.
FERRERO	F.F6	Acreditamos que todos os tipos de alimentos podem ser consumidos como parte de uma dieta variada e equilibrada, sem excluir determinados alimentos, ingredientes ou nutrientes específicos. Um dos nossos objetivos empresariais consiste em ajudar os consumidores a realizar escolhas alimentares adequadas e a adotar uma dieta variada e equilibrada que lhes proporcione nutrientes essenciais e uma quantidade correta de energia de todos os tipos de alimentos.
FERRERO	F.F7	Estamos empenhados em realizar uma comunicação responsável que ajude os consumidores a realizar escolhas adequadas sobre a comida e bebida que compram e consomem.
FERRERO	F.F8	A comunicação e a rotulagem ajudam os consumidores a compreender o valor da nutrição, da dieta e do movimento para a consecução de um estilo de vida saudável e ativo.
FERRERO	F.F9	RESPONSABILIDADE SOCIAL DO GRUPO De momento, a Ferrero é uma das maiores empresas do mundo pela reputação que tem. Certamente que tal deve-se também às iniciativas de responsabilidade social do Grupo. Estas resumem-se na estratégia de RSC da Ferrero, que se centra na mais alta qualidade e inovação, na comunicação transparente, no cuidado com as pessoas que fizeram e ainda fazem a história do Grupo, no apoio às comunidades locais, na promoção de estilos de vida ativos entre os jovens e no forte compromisso com práticas agrícolas sustentáveis e proteção ambiental.
FERRERO	F.F10	A nossa atenção constante às necessidades dos consumidores está no alicerce da política da empresa, que se inspira pela máxima transparência e excelência na qualidade. A nossa lealdade para com os consumidores e a confiança que

		depositam nos nossos produtos ao comprá-los diariamente estão no cerne da relação duradoura que desfrutamos com eles. As práticas da empresa baseiam-se na confiança em uma confiança mútua entre colegas, na dedicação total e na transparência para com o Grupo, bem como para com todos os atores da sociedade civil com os quais interagimos diariamente.
FERRERO	F.F11	Com base no respeito pelo princípio da igualdade de tratamento, promovemos o desenvolvimento profissional e pessoal dos nossos recursos humanos. Isso também reforça os nossos fortes laços com as comunidades locais em territórios onde operamos. Estamos comprometidos a evitar qualquer forma de discriminação no que toca às práticas trabalhistas em empresas do Grupo, desde o momento inicial de recrutamento. O principal objetivo da nossa estratégia trabalhista é estabelecer relações duradouras, inspiradas nos princípios de crescimento profissional e reconhecimento de mérito. Garantimos aos nossos funcionários liberdade de pertencer a sindicatos; reconhecemos o papel dos sindicatos em assuntos contratuais. A protecção da saúde humana e o respeito pelo meio ambiente estão no cerne de nossas práticas operacionais. Gerenciamos as instalações pela utilização de recursos sustentáveis como água, energia, matérias-primas e outros materiais, incluindo o uso de recursos renováveis. Buscamos reduzir o impacto ambiental de todas as formas, especificamente pela redução de emissões, eliminação e gestão correcta de resíduos. Apoiamos a protecção à dignidade humana, bem como o respeito absoluto e incondicional dos direitos humanos, onde quer que as empresas do Grupo Ferrero operem. Apoiamos a abolição da exploração do trabalho infantil e do trabalho forçado e coercivo no âmbito de nossa esfera de influência e ao longo de toda a cadeia produtiva, por meios legais específicos e estreitas relações com as comunidades locais.
FERRERO	F.F12	As nossas comunicações com os consumidores, incluindo a publicidade, respeitam os valores da dignidade humana, da família e das crianças, de acordo com os nossos princípios morais e éticos. Eles baseiam-se no aconselhamento quanto à utilização adequada dos nossos produtos e inspiram-se na promoção de um estilo de vida saudável. As nossas práticas de defesa em relação às instituições internacionais, nacionais e locais baseiam-se em sólidos conhecimentos científicos; inspiram-se sempre na integridade intelectual e comportam
FERRERO	F.F13	(…) se empreendedorismo pode ser vinculado à palavra "iniciativa", valor que por si só incorpora os conceitos de empresa e empreendedorismo, que impulsiona a agir por iniciativa própria para maximizar os resultados. A diferença entre uma estratégia brilhante e êxito reside na capacidade de estabelecer uma visão clara, em ser proativo com os investimentos, em estar pronto para os empreendimentos, em ser excelente ao executá-los. O Grupo Ferrero é extremamente bem-sucedido devido à sua cultura sólida e profundamente enraizada de "aprender fazendo". Um elemento crucial da sustentabilidade no modelo da Ferrero é o acúmulo de habilidades técnicas e o conhecimento dos produtos, mercados e consumidores ao longo do tempo, que conduz a empresa do "criar valor" ao fortalecimento do "valor da criação". Uma grande entidade coletiva orientada pela confiança no progresso e pela capacidade de imaginar um amanhã melhor do que o hoje. Isso porque o mundo sempre será conquistado por aqueles que desejam sucesso e afirmação e aqueles que têm determinação para almejar a excelência de maneira contínua.
FERRERO	F.F14	Nós identificamo-nos com o lema: "Trabalhar, criar, doar", concebido e apresentado por Michele Ferrero no início das atividades empreendedoras. Em essência, encorajamos a "ética do fazer" acima da "prática de aparecer". Essa mesma filosofia tem inspirado e ainda inspira: A Fundação Ferrero, dedicada à manutenção das relações do Grupo com os aposentados por meio de programas sociais e culturais, bem como ao fortalecimento dos laços com as comunidades locais em que operamos; ! ! O "Progetto Imprenditoriale Michele Ferrero", baseado em investimentos e utilização dos recursos obtidos para melhorar as condições de vida de comunidades de países em desenvolvimento, com especial atenção às crianças; ! Todo o Grupo Ferrero está constantemente envolvido em atividades de responsabilidade social como parte integrante da própria maneira de ser e de operar, desde o início.
FERRERO	F.F15	Desde a fundação da empresa familiar em 1946, em Alba, na Itália, a Ferrero esteve sempre dedicada a assumir compromissos sólidos e a prestar a máxima atenção às pessoas e ao planeta. Vai desde prestar atenção às pessoas que fizeram e continuam a fazer a história da Ferrero, garantindo um local de trabalho diverso e gratificante, e promovendo estilos de vida ativos entre os jovens e as suas famílias, até ao nosso compromisso firme com as práticas agrícolas sustentáveis, a proteção do ambiente e o apoio das comunidades locais. Esta tendência faz parte do nosso ADN e está simbolicamente representada numa carta que Michele Ferrero enviou aos empregados da empresa em 1957, quando assumiu a direção da mesma.
FERRERO	F.F16	O cuidado das pessoas que trabalham para e com a Ferrero está profundamente enraizado nos nossos valores como empresa familiar. O respeito pelos direitos humanos de todas e cada uma das pessoas da nossa empresa e das comunidades em que operamos é e sempre foi um dos nossos valores fundamentais. Estamos empenhados no respeito e promoção dos direitos humanos, e na prevenção e mitigação de qualquer impacto negativo das nossas operações, através de uma abordagem aos direitos humanos baseada na devida diligência e nos Princípios Orientadores das Nações Unidas sobre Negócios e Direitos Humanos, na Carta Internacional dos Direitos Humanos, e nas Convenções Fundamentais da Organização Internacional do Trabalho (OIT).
FERRERO	F.F17	A Ferrero trabalha para garantir que todas as nossas cadeias de abastecimento são justas, sustentáveis e transparentes. Durante muitos anos, construímos relações a longo prazo com os nossos fornecedores, dado acreditarmos que a parceria e a colaboração são essenciais para garantir o sucesso do nosso compromisso com as aquisições responsáveis.
FERRERO	F.F18	Em junho de 2021, a Ferrero atualizou a sua Carta do Óleo de Palma originalmente publicada em 2013. A nova Carta define a nossa ambição de contarmos com uma indústria do óleo de palma que seja benéfica tanto para as pessoas como para a natureza, enfrentando os desafios com ações que envolvam os fornecedores, e que vão mais além das mais exigentes normas de certificação. A Carta foi promovida com base na atual viagem de aprendizagem da empresa e com as visões extraídas de uma estreita colaboração entre as partes interessadas a todos os níveis, dos fornecedores às ONG, passando por parceiros do âmbito académico.
FERRERO	F.F19	A Carta do Cacau define a nossa visão para uma indústria do cacau verdadeiramente sustentável que, através da melhoria contínua, seja benéfica para as pessoas e para a natureza e crie valor para todos. A Carta traça os nossos compromissos a longo prazo, com objetivos a médio prazo específicos (2023).
FERRERO	F.F20	Em linha com o Código de Fornecedores, em janeiro de 2021, a Ferrero publicou uma Carta dedicada às avelãs. Esta Carta aplica-se a todo o aprovisionamento de avelãs, com uma abordagem assente em garantir o cumprimento das nossas políticas em toda a cadeia de valor e na nossa convicção de que temos de “ir mais além” da conformidade, identificando compromissos específicos de acordo com um conjunto de prioridades e iniciativas.
FERRERO	F.F21	A emergência climática exige que os negócios reforcem o seu compromisso com a redução das emissões de carbono e ajudem a limitar as alterações climáticas

FERRERO	F.F22	A água é um precioso recurso partilhado que sustém comunidades, ecossistemas e a biodiversidade. Mas os recursos de água potável estão submetidos a uma pressão cada vez maior pelos impactos das alterações climáticas e pelas exigências competitivas das comunidades, da agricultura e da indústria.
FERRERO	F.F23	A e%ciência energética é a nossa principal alavanca para a redução de emissões, bem como, ao mesmo tempo, para a diminuição dos custos e para a melhoria do desempenho das nossas atividades. Dedicamos ativos e projetos à otimização das nossas atividades, de acordo com as melhores técnicas disponíveis. Os projetos são identi%cados através de avaliações cientí%cas e de procedimentos como auditorias energéticas, avaliações técnicas, ateliers para os empregados e a partilha de melhores práticas através dos nossos auditores internos e de especialistas em energia.
FERRERO	F.F24	As embalagens representam um dos maiores desa/os que a indústria alimentar e das bebidas enfrenta atualmente.
FERRERO	F.F25	Estamos conscientes de que a cadeia de abastecimento de óleo de palma enfrenta importantes desa@os ambientais, relacionados principalmente com o seu impacto em termos de desQorestação. É por esse motivo que a Ferrero está empenhada em liderar o processo de transformação sustentável do setor do óleo de palma, e foi uma das primeiras empresas globais a estabelecer uma cadeia de abastecimento segregada de óleo de palma, 100% certi@cada pela RSPO.
FERRERO	F.F26	Ferrero acredita que uns quadros de pessoal diversos e inclusivos são cruciais para o sucesso a longo prazo da sua atividade. Um dos seus objetivos consiste em motivar os seus empregados e melhorar a sua capacidade de tomada de decisões, a sua orientação para o consumidor e o seu envolvimento.
FERRERO	F.F27	O projeto empresarial Michele Ferrero inclui empresas comerciais para todos os efeitos: o objetivo destas empresas é, portanto, conseguir lucro. Contudo, elas atuam com espírito “social” porque visam criar postos de trabalho nas áreas menos desenvolvidas dos países emergentes. Para além disso, estas empresas implementam projetos e iniciativas de natureza humanitária e social, que visam velar pela saúde e pelo desenvolvimento educativo e social de crianças e jovens adultos nesses países, independentemente do conseguimento, ou não conseguimento, de resultados positivos de balanço.
FERRERO	F.F28	A proteção da saúde e segurança no trabalho é um valor central da Ferrero. Organizamos diversas iniciativas para aumentar a consciencialização sobre temas de saúde e segurança em atividades ocupacionais.
FERRERO	F.F29	Como primeiros consumidores dos produtos Ferrero e primeiros embaixadores da abordagem Ferrero à sustentabilidade, a criatividade dos nossos empregados é crucial para a partilha das melhores práticas.
FERRERO	F.F30	O nosso compromisso com a proteção do meio ambiente abrange as comunidades das áreas em que operamos. Apoiamo-las e encorajamos os nossos empregados a dedicarem o seu tempo a iniciativas que protejam a biodiversidade e melhorem o meio ambiente local.
FERRERO	F.F31	A nossa abordagem ecológica ao design de embalagens desempenha um papel crucial na consecução dos nossos compromissos com a sustentabilidade. E estamos a aplicá-la para melhorar as nossas embalagens a nível global.
FERRERO	F.F32	As nossas atividades a nível global encorajam os empregados da Ferrero a trabalhar com comunidades locais e com as jovens gerações para proteger o meio ambiente.
FERRERO	F.F33	A prática do desporto representa um momento muito importante no crescimento das crianças e dos jovens, que no desporto encontram valores positivos e a possibilidade de um desenvolvimento físico e psíquico são e controlado.
FERRERO	F.F34	Aproximámo-nos das famílias através da promoção da alegria do movimento
FERRERO	F.F35	Compromisso com a autorregulação voluntária
FERRERO	F.F36	PROMOVER UM CONSUMO RESPONSÁVEL Preocupamo-nos com o consumo responsável dos nossos produtos. Esforçamo-nos por oferecer produtos da mais alta qualidade e frescura, empenhando-nos constantemente por comunicarmos de forma responsável.
FERRERO	F.F37	Todo o Grupo Ferrero está constantemente envolvido em atividades de responsabilidade social como parte integrante da própria maneira de ser e de operar, desde o início.

FIPA

Website	Code	CPA EXAMPLES
FIPA	F.FI1	Adequação da política fiscal à competitividade;
FIPA	F.FI2	De que temos de continuar a reforçar o valor da indústria agroalimentar para o país - quer no mercado interno quer na projeção internacional -, apostando em pilares estratégicos como a investigação, desenvolvimento e inovação de produto e de modelos de negócios, a transição digital, a sustentabilidade ambiental e o reforço da união de toda a fileira agroalimentar para otimização e valorização da produção nacional.
FIPA	F.FI3	Nunca é de mais afirmar que as empresas do setor têm tido um papel central quer no contexto económico nacional quer no abastecimento alimentar assente na qualidade, diversidade e segurança.
FIPA	F.FI4	Este é, de facto, um claro exemplo do que incessantemente defendemos, ou seja, é prova de que os bons resultados se atingem quando há cooperação e abertura para o diálogo. Da mesma forma, defendemo-lo no debate sobre economia circular - promovido pela CIP - Confederação Empresarial de Portugal –, sublinhando que, para o sucesso da sua concretização na cadeia de valor alimentar, a circularidade deve ter por base uma abordagem integrada não só por parte das empresas, mas também política, governamental e científica.
FIPA	F.FI5	A indústria agroalimentar é um setor estratégico para Portugal, quer ao nível económico, do emprego, do desenvolvimento regional, quer da autossuficiência alimentar, entre outros. Os próximos anos não irão certamente abrandar a complexidade dos desafios que se têm vindo a colocar a toda a economia nacional e, em particular, à indústria agroalimentar.
FIPA	F.FI6	A indústria de alimentos e bebidas é a maior indústria de transformação da Europa, o maior empregador e um dos principais players da nossa economia
FIPA	F.FI7	CONTRIBUIÇÃO ECONÓMICA É a indústria transformadora que mais contribui para a economia nacional, tanto em Volume de Negócios (17,6 mil milhões de euros) como em Valor Acrescentado Bruto (3,4 mil milhões de euros). CONTRIBUIÇÃO PARA O EMPREGO É a segunda indústria transformadora que mais emprega em Portugal, sendo responsável por mais de 119.000 postos de trabalho diretos e cerca 500.000 indiretos. de CONTRIBUIÇÃO PARA A BALANÇA COMERCIAL Tem contribuído para o equilíbrio da balança comercial, registando na última década uma taxa de crescimento das exportações superior às importações. Mantendo o nível de crescimento dos dois fluxos, esta indústria poderá vir a tornar-se exportadora líquida. CONTRIBUIÇÃO PARA O CRESCIMENTO Tem mantido, nos últimos anos, um desempenho acima da média da economia nacional, existindo boas expectativas de crescimento na próxima década por parte dos gestores e empresários do setor. CONTRIBUIÇÃO PARA A SUSTENTABILIDADE

		DE OUTROS SETORES ECONÓMICOS Tem impacto, direto e indireto, elevado noutros setores da economia portuguesa, com destaque para os setores a montante da cadeia de valor. CONTRIBUIÇÃO PARA O DESENVOLVIMENTO REGIONAL Assume uma grande importância no desenvolvimento do tecido empresarial e criação de emprego nas zonas menos desenvolvidas do país. CONTRIBUIÇÃO PARA A AUTOSSUFICIÊNCIA ALIMENTAR É fundamental para a afirmação do potencial de evolução da nossa autossuficiência alimentar e para garantir a sustentabilidade do consumo nacional.
FIPA	F.FI8	FIPA tem procurado munir os decisores políticos com informação credível e apoiada na realidade do mercado para evidenciar o efeito negativo que o aumento desajustado da carga fiscal tem sobre o consumo, as empresas e o emprego. Depois de uma subida abrupta do IVA de várias categorias e da implementação do imposto especial (IEC) sobre as bebidas açucaradas, tem surgido a ameaça de novos impostos similares, cujos efeitos práticos não têm sólida sustentação. Estes promovem a discriminação negativa de alguns alimentos e bebidas e colocam em causa, de forma generalizada, a reputação de um setor estratégico para a economia, promotor de altos padrões de segurança dos alimentos e com necessidade permanente de inovação.
FIPA	F.FI9	A indústria portuguesa agroalimentar tem sido a indústria transformadora que mais tem contribuído para a economia nacional.
FIPA	F.FI10	Acima de tudo, é um setor estratégico para a prossecução dos objetivos a nível económico e social, seja pelo seu papel dinamizador da fileira e do desenvolvimento rural, pela criação de emprego ou pela preservação da identidade cultural.
FIPA	F.FI11	"a inclusão da Alimentação na titularidade de um ministério vem ao encontro das propostas que a FIPA tem vindo a apresentar, por considerarmos essencial a valorização do setor agroalimentar em virtude da sua forte contribuição tanto para a economia nacional como para a sociedade".
FIPA	F.FI12	Neste enquadramento, consideramos essencial continuarmos o caminho do diálogo, tanto com o Governo como com o Parlamento. Acreditamos que o sentido de cooperação será fulcral não só para fazer face aos tempos de enorme imprevisibilidade em que todos nos encontramos, mas, acima de tudo, para a definição de uma estratégia alimentar consolidada para um futuro no qual não abrandarão os desafios económicos, ambientais e sociais.
FIPA	F.FI14	Durante as últimas legislaturas, a FIPA tem sido parceira incontornável na definição das políticas nacionais para a internacionalização do setor agroalimentar, tendo apresentado aos sucessivos Governos os seus "Contributos para uma Visão Estratégica".
FIPA	F.FI15	Acima de tudo, é um setor estratégico para a prossecução dos objetivos a nível económico e social, seja pelo seu papel dinamizador da fileira e do desenvolvimento rural, pela criação de emprego ou pela preservação da identidade cultural.
FIPA	F.FI16	Contribuir para a produção de informação robusta, com base no conhecimento científico e tecnológico relevante, para promover uma comunicação eficaz, por forma a assegurar que todos os assuntos relacionados com a política agroalimentar são geridos de forma holística.
FIPA	F.FI17	1. Promoção de hábitos alimentares saudáveis;
FIPA	F.FI18	Promover, em conjunto com as nossas associadas, a criação de uma envolvente na qual todas as empresas do setor agroalimentar, independentemente da sua dimensão, possam acompanhar os constantes desafios colocados pelos consumidores e, ao mesmo tempo, estejam aptas a competir por um crescimento sustentável, inteligente e inclusivo.
FIPA	F.FI19	O Nosso Papel Contribuir para afirmar a posição competitiva da indústria agroalimentar – numa combinação entre a tradição e inovação – reforçando a confiança e o seu reconhecido papel no dia-a-dia dos consumidores, ao fornecer produtos seguros, nutritivos, acessíveis e sustentáveis. Defender e promover o interesse das suas associadas em áreas como a qualidade e segurança alimentar, nutrição e saúde, confiança e informação ao consumidores, exportação e internacionalização, investigação, desenvolvimento e inovação e sustentabilidade. Contribuir para a produção de informação robusta, com base no conhecimento científico e tecnológico relevante, para promover uma comunicação eficaz, por forma a assegurar que todos os assuntos relacionados com a política agroalimentar são geridos de forma holística. Trabalhar em estreita colaboração com os principais parceiros, de forma a promover uma resposta global e integrada aos desafios colocados pelas complexas mudanças sociais, económicas e ambientais.
FIPA	F.FI20	CONSUMIDOR | Promover sinergias entre a indústria agroalimentar e os decisores políticos, parceiros e sociedade em geral, com vista à promoção de escolhas informadas e estilos de vida saudáveis. COMPETITIVIDADE | Promover um compromisso nacional para a indústria agroalimentar que permita reforçar a competitividade, alavancar o crescimento externo, fomentar o emprego e assegurar a confiança dos consumidores. INOVAÇÃO | Defender a criação de condições que permitam intensificar a aposta em investigação, desenvolvimento e inovação já levadas a cabo pela indústria agroalimentar e defender, igualmente, um enquadramento legal que tenha por base comprovadas evidências científicas. SUSTENTABILIDADE | Mobilizar toda a envolvente para uma abordagem sensata à relação única que a indústria agroalimentar tem com todos os intervenientes da cadeia de valor, garantindo o equilíbrio entre a preservação dos recursos e a competitividade a longo-prazo, numa perspetiva económica, social e ambiental.
FIPA	F.FI21	Transversalmente as nossas prioridades são: 1. Promoção de hábitos alimentares saudáveis; 2. Equilíbrio na relação entre fornecedores e distribuidores; 3. Suporte à exportação e internacionalização; 4. Adequação da política fiscal à competitividade; 5. Estratégia pública de incentivos à inovação; 6. Transição para a economia circular.
FIPA	F.FI22	De que temos de continuar a reforçar o valor da indústria agroalimentar para o país - quer no mercado interno quer na projeção internacional -, apostando em pilares estratégicos como a investigação, desenvolvimento e inovação de produto e de modelos de negócios, a transição digital, a sustentabilidade ambiental e o reforço da união de toda a fileira agroalimentar para otimização e valorização da produção nacional.
FIPA	F.FI23	A indústria portuguesa agroalimentar entrou na segunda década deste século focada no aumento da sua competitividade, procurando dar uma resposta cada vez mais consistente aos desafios colocados pelos consumidores nacionais e batendo recordes nos mercados de exportação.
FIPA	F.FI24	A Indústria Portuguesa Agroalimentar tem sido confrontada com enormes desafios, nomeadamente ao nível dos seus fatores de competitividade e da sua relação com a sociedade e o meio ambiente. De forma particular, o caminho para a exportação e a internacionalização das empresas nacionais, como forma de conquista de novas oportunidades, torna-se uma inevitabilidade.
FIPA	F.FI25	A Estratégia "Do Prado ao Prato" agrega vários desafios de sustentabilidade para a indústria agroalimentar, multiplicando-se por áreas como o impacte ambiental, as alterações climáticas, a biodiversidade, a segurança no abastecimento, a qualidade alimentar e nutricional e a distribuição justa de valor.

IGLO

Website	Code	CPA EXAMPLES
IGLO	F.I1	E temos uma missão: tornar acessível a todas as pessoas os benefícios dos vegetais e garantir que oferecemos produtos deliciosos aos portugueses. Queremos promover melhores hábitos alimentares para todos, a começar por ti! Hábitos que fazem bem à nossa saúde e ao planeta. Parece simples, mas nem sempre é fácil pôr boas intenções em prática. É por isso que criamos produtos deliciosos e nutritivos para quando tens pouco tempo ou simplesmente não sabes como criar uma receita deliciosa repleta de vegetais. A nossa gama Iglo Green Cuisine garante produtos saborosos, com tudo de bom dos vegetais, um benefÍcio para o bem-estar de todas as famílias e com os olhos postos no futuro de todos nós.
IGLO	F.I2	A comida congelada é uma excelente escolha para um estilo de vida sustentável. É versátil, está sempre disponível e mantém-se fresca durante mais tempo. É também tão nutritiva como os produtos frescos e ajuda a reduzir o desperdício alimentar. Estamos a fazer progressos para contribuir para promover dietas mais saudáveis e sustentáveis. Pescamos o nosso peixe e marisco usando práticas sustentáveis há mais de 20 anos. Produzimos ainda a maioria dos nossos vegetais de acordo com princípios de agricultura sustentável. Estamos continuamente a desenvolver a nossa agenda de sustentabilidade, repensando o nosso papel no mundo e o impacto que temos enquanto empresa. É por isso que colocamos a sustentabilidade no seio da nossa atividade:
IGLO	F.I3	Queremos inspirar as famílias a optarem por uma dieta mais equilibrada e a terem um estilo de vida mais saudável. Graças à nossa vasta gama de peixe, vegetais e frango estamos numa excelente posição para o fazer. Todas as nossas receitas são avaliadas usando a nossa Ferramenta de Perfil Nutricional personalizada, que confirmou que 80% dos nossos produtos são "escolhas saudáveis".
IGLO	F.I4	Trabalhar em conjunto para um futuro melhor O mundo está em rápida transformação e temos de trabalhar em conjunto para assegurar que os nossos recursos são usados da melhor forma para assegurarmos o futuro do nosso planeta.
IGLO	F.I5	Peixe para todos Para salvaguardar o futuro dos nossos oceanos, temos um compromisso para com a pesca sustentável. Assim, sempre que escolhe o nosso peixe, escolhe peixe que é bom para todos. Para as Pessoas, para o Planeta, para o Futuro.
IGLO	F.I6	Desenvolvemos uma estratégia de sustentabilidade que nos permite criar um futuro em que as pessoas e o planeta possam prosperar. Disponibilizar produtos com um sabor delicioso e provenientes de fontes sustentáveis é crucial em tudo o que fazemos. Desde os nossos douradinhos de peixe às nossas nutritivas ervilhas, os consumidores podem usufruir diariamente de uma vasta gama de produtos produzidos responsavelmente.
IGLO	F.I7	Obtemos e produzimos os nossos produtos com cuidado e respeito pelas pessoas e pelo ambiente. Focamo-nos em peixe e marisco provenientes de fontes responsáveis e sustentáveis e os nossos padrões de agricultura sustentável estão em expansão, passando de práticas regionais a padrões globais.
IGLO	F.I8	Estamos a reduzir o nosso impacto no ambiente focando-nos na gestão de resíduos, água e energia. A sustentabilidade é importante para os nossos colaboradores, razão pela qual trabalhamos em conjunto rumo a um futuro mais sustentável. Fazemos todos os esforços para assegurar a igualdade e a diversidade entre os nossos colaboradores.
IGLO	F.I9	O peixe é bom para a nossa saúde* e para a boa saúde dos oceanos. Aqui, na Iglo, pescamos com responsabilidade enquanto ajudamos a preservar os recursos marinhos e habitats naturais, para que as gerações futuras também possam desfrutar do melhor peixe. Assim, sempre que escolhe o nosso peixe, escolhe peixe que é bom para todos. Para as Pessoas, para o Planeta, para o Futuro.
IGLO	F.I10	PESCAMOS SEMPRE COM RESPONSABILIDADE Temos orgulho em ser transparentes sobre onde e como pescamos, protegendo os recursos futuros ao trabalharmos com aqueles que partilham o nosso sentido de responsabilidade pelos recursos marinhos, pelos habitats e pelos ecossistemas.
IGLO	F.I11	Para quem procura uma alimentação mais saudável, nutritiva e sustentável, apresentamos uma nova gama de produtos revolucionários e surpreendentes, 100% aptos para vegetarianos ou para quem quer adicionar mais vegetais ao seu diaa-dia.
IGLO	F.I12	Queremos que cada vez mais pessoas provem os nossos produtos, por isso desenvolvemos estas receitas especialmente formuladas para pessoas com intolerância ao glúten, mantendo todo o sabor que caracteriza os produtos Iglo.
IGLO	F.I13	Com Green Cuisine é fácil fazeres a tua parte em prol do meio ambiente e de uma alimentação saudável, seja substituindo os clássicos nuggets por uns igualmente deliciosos nuggets 0% frango, o teu hambúrger favorito por um hambúrger 0% carne, ou simplesmente por optares por uns fingers repletos de espinafres, ou umas deliciosas veggie bowls. Green Cuisine torna a preparação de uma refeição repleta de vegetais em algo delicioso e simples de preparar.
IGLO	F.I14	Refeições inspiradoras e escolhas saudáveis Encorajamos os nossos consumidores a comerem um bocadinho melhor todos os dias com a nossa oferta variada de produtos deliciosos
IGLO	F.I15	Inovar por um futuro mais sustentável Impulsionamos a inovação na área alimentar e continuamos a otimizar a nossa gama para ajudar os consumidores a fazerem escolhas alimentares mais sustentáveis e saudáveis.
IGLO	F.I16	Estamos empenhados no nosso compromisso com o futuro do planeta fornecendo alimentos e refeições mais sustentáveis, acessíveis a todos. Aqui encontrarás algumas das muitas maneiras como os novos produtos Iglo Green Cuisine contribuem para um planeta melhor e mais saudável.
IGLO	F.I17	Os Nossos Objectivos Desenvolvemos continuamente uma estratégia de sustentabilidade, repensando o nosso papel no mundo e o impacto que temos enquanto empresa. É por isso que colocamos a sustentabilidade no seio da nossa atividade.
IGLO	F.I18	Alimentos congelados ajudam o planeta Sabia que 1/3 de todos os alimentos são desperdiçados? A diminuição do desperdício alimentar é apenas um dos benefícios dos alimentos congelados Frozen Foods Help the Planet: Did you know that 1/3 of all food is wasted? Decreasing food waste is just one of the benefits of frozen foods.
IGLO	F.I19	PESCAMOS SEMPRE COM RESPONSABILIDADE Temos orgulho em ser transparentes sobre onde e como pescamos, protegendo os recursos futuros ao trabalharmos com aqueles que partilham o nosso sentido de responsabilidade pelos recursos marinhos, pelos habitats e pelos ecossistemas.
IGLO	F.I20	Trabalhar arduamente para reduzir o desperdício Além de reduzirmos o desperdício alimentar, trabalhamos arduamente para reduzir o desperdício na nossa cadeia de abastecimento.

IMPERIAL

Website	Code	CPA EXAMPLES
IMPERIAL	F.IM1	Vamos ajudar a nossa economia neste período especial. Consumir e divulgar as marcas portuguesas é proteger a nossa economia, reduzir o desemprego e o impacto social e económico desta crise. Partilhe as suas escolhas com a hashtag: #EuEscolhoMarcasPortuguesas #portugal #economia

IMPERIAL	F.IM2	A Regina é a marca portuguesa de chocolates mais reconhecida e a mais popular em Portugal, recolhendo o afeto de gerações inteiras.
IMPERIAL	F.IM3	É uma marca associada a valores de tradição e qualidade, fruto da confiança que os consumidores lhe atribuem e que permite alcançar excelentes resultados na confeção de deliciosas sobremesas de chocolate.
IMPERIAL	F.IM4	Coma chocolate: Um alimento que melhora a sua vida

JERÓNIMO MARTINS

Website	Code	CPA EXAMPLES
JERÓNIMO MARTINS	F.JM1	"Acreditamos em fazer a diferença pela criação de valor de uma forma sustentável e socialmente responsável."
JERÓNIMO MARTINS	F.JM2	Queremos criar valor, mas não a qualquer preço. Os nossos 230 anos de história, provam que sabemos olhar para o longo prazo enquanto cuidamos do presente. Temos consciência da importância do respeito pelos recursos naturais, da preservação da biodiversidade e da procura de uma melhor qualidade de vida.
JERÓNIMO MARTINS	F.JM3	Como especialista alimentar, temos o poder de influenciar práticas e processos com impacto direto e relevante no ambiente e nas pessoas. É por isso que assumimos uma relação equilibrada entre prosperidade económica, desenvolvimento social e preservação ambiental. Temos a responsabilidade de adotar boas práticas e elevar os padrões de qualidade ao longo de toda a cadeia de valor, em torno dos pilares que definimos na nossa estratégia de Responsabilidade Corporativa e que são transversais a todas as Companhias do Grupo.
JERÓNIMO MARTINS	F.JM4	ALIMENTAR A SAÚDE Como especialistas alimentares promovemos uma alimentação saudável, o consumo responsável e produtos acessíveis.
JERÓNIMO MARTINS	F.JM5	Estamos conscientes da nossa responsabilidade em ajudar os consumidores a tomarem decisões que contribuam para um estilo de vida saudável e que ajudem a evitar doenças que são problemas sérios de saúde pública como a diabetes, a osteoporose e as cardiovasculares.
JERÓNIMO MARTINS	F.JM6	A prioridade das reformulações recai sobre produtos de grande consumo, que sejam percecionados como benéficos para a saúde ou estejam entre os mais consumidos por públicos infantis. Desta forma estamos a contribuir para uma saúde pública de qualidade.
JERÓNIMO MARTINS	F.JM7	Estamos empenhados em oferecer soluções para responder às diferentes tendências de consumo. Consumidores adeptos de estilos de vida mais saudáveis, que procurem soluções alimentares de conveniência com perfil nutricional equilibrado ou que tenham necessidades e/ou preferências dietéticas específicas encontram uma resposta nas nossas lojas. Temos, por isso, o compromisso de desenvolver, em todos os países onde operamos, alternativas alimentares destinadas a grupos específicos de consumidores, como soluções vegan, sem lactose e/ou sem glúten.
JERÓNIMO MARTINS	F.JM8	O Grupo mantém a aposta em disponibilizar produtos que respondam às necessidades e preferências dos consumidores enquanto alarga também a oferta de alimentos que contribuam para padrões de consumo mais saudáveis.
JERÓNIMO MARTINS	F.JM9	Procuramos que os produtos destinados a públicos infantis tenham um perfil nutricional saudável e melhor que o benchmark de cada mercado.
JERÓNIMO MARTINS	F.JM10	Promovemos decisões de compra responsáveis e informadas através de uma comunicação transparente e proativa com os consumidores.
JERÓNIMO MARTINS	F.JM11	Adotamos várias estratégias de comunicação com os nossos clientes no sentido de os sensibilizar para opções de alimentação saudável e escolhas sustentáveis.
JERÓNIMO MARTINS	F.JM12	Porque a Distribuição Alimentar é a nossa paixão, desenvolvemos uma política nutricional que promove a adoção de estilos de vida saudáveis. Na qualidade de especialista em Distribuição Alimentar internacional, o Grupo Jerónimo Martins está consciente das suas responsabilidades ao ajudar os consumidores a tomarem decisões responsáveis com um impacto positivo sobre os seus estilos de vida, através do desenvolvimento e promoção de uma oferta alimentar segura, saudável, nutritiva e acessível. Como membro fidedigno da sociedade e no âmbito da Responsabilidade Corporativa do Grupo, Jerónimo Martins quer contribuir também para a prevenção de doenças relacionadas com a alimentação, como a obesidade, a diabetes, a osteoporose e as doenças cardiovasculares, funcionando como um agente ativo no combate global contra problemas de saúde pública sérios.
JERÓNIMO MARTINS	F.JM13	A nossa visão: Enquanto especialistas alimentares, trabalhamos todos os dias para continuarmos a merecer a preferência de milhares de portugueses, através de produtos de qualidade, inovadores, seguros e que sejam benéficos para a sua saúde. Conscientes das tendências de consumo atuais, a nossa responsabilidade estende-se também à oferta de soluções para consumidores com necessidades e ou preferências alimentares específicas como celíacos, vegans e vegetarianos, ou que procurem produtos sem gordura ou sem açúcares. A promoção de hábitos alimentares mais saudáveis e o papel ativo que queremos ter nas escolhas informadas dos nossos clientes são outras das nossas prioridades. Nos produtos de Marca Própria Pingo Doce, disponibilizamos informação clara e transparente. Para além da informação legal, como os alergénios ou ingredientes passíveis de intolerância, destacamos ainda informação nutricional relevante, como a presença de vitaminas e fibras ou os valores nutricionais por porção. Além dos rótulos são usados outros meios de comunicação como forma de contribuir para decisões informadas pelos nossos consumidores. Mesmo depois de chegarem às prateleiras, os produtos de marca Pingo Doce continuam a ser acompanhados e analisados, de modo a garantir a sua máxima qualidade e segurança. O acompanhamento destes produtos pressupõe ainda, sempre que possível, que sejam feitas melhorias ao seu perfil nutricional, que passam pela redução gradual do teor de sal, açúcar e gorduras – mantendo sempre o foco no sabor –, pela diminuição do uso de aditivos como corantes e conservantes; pela adequação do tamanho das porções e/ou pela inclusão de ingredientes como vitaminas e minerais. Promovemos a Dieta Mediterrânica como padrão alimentar de referência e desenvolvemos ações e programas em prol de hábitos alimentares mais saudáveis e estilos de vida ativos.
JERÓNIMO MARTINS	F.JM14	Ofereceremos produtos de qualidade, seguros e a preços competitivos aos nossos consumidores. Ao mesmo tempo, procuramos minimizar os impactes desses produtos sobre os ecossistemas.
JERÓNIMO MARTINS	F.JM15	Trabalhamos todos os dias para oferecer alimentação de qualidade a preços acessíveis, criando valor de forma sustentável e responsável. É este o nosso compromisso presente e futuro. Isabel Pinto Diretora-Geral Pingo Doce
JERÓNIMO MARTINS	F.JM16	Reformulações A prioridade das reformulações recai sobre produtos de grande consumo, que sejam percecionados como benéficos para a saúde ou estejam entre os mais consumidos por públicos infantis. Desta forma estamos a contribuir para uma saúde pública de qualidade. Temos reduzido os teores de sal, gordura, gordura saturada, açúcar, conservantes e outros ingredientes artificiais ou potencialmente geradores de alergias, adicionando, sempre que

		possível, maiores quantidades de fibra, fruta e micronutrientes. O objetivo é prevenir e combater doenças relacionadas com a alimentação, entre as quais a obesidade, a diabetes, a osteoporose e as cardiovasculares.
JERÓNIMO MARTINS	F.JM17	Soluções Diferenciadas Estamos empenhados em oferecer soluções para responder às diferentes tendências de consumo. Consumidores adeptos de estilos de vida mais saudáveis, que procurem soluções alimentares de conveniência com perfil nutricional equilibrado ou que tenham necessidades e/ou preferências dietéticas específicas encontram uma resposta nas nossas lojas. Temos, por isso, o compromisso de desenvolver, em todos os países onde operamos, alternativas alimentares destinadas a grupos específicos de consumidores, como soluções vegan, sem lactose e/ou sem glúten.
JERÓNIMO MARTINS	F.JM18	Para responder a estes princípios, a política de desenvolvimento de produtos Marca Própria do Grupo Jerónimo Martins baseia-se em seis pilares: • Perfil nutricional • Ingredientes • Rotulagem • Tamanho das porções • Melhoria contínua • Comunicação Além disso, o Grupo dá especial atenção ao sal, gordura (saturada e ácidos gordos trans), açúcar, aditivos, enriquecimento de micro nutrientes, tamanho das porções e composição nutricional por porção na rotulagem do produto. Todas as ações levadas a cabo no âmbito da Política Nutricional do Grupo serão monitorizadas e comunicadas de modo a avaliar o seu impacto e exatidão.
JERÓNIMO MARTINS	F.JM19	Em todos os países, desenvolver alternativas alimentares destinadas a consumidores com necessidades/preferências alimentares específicas, como soluções veganas, sem lactose e/ou sem glúten. Garantir que os produtos destinados a públicos infantis têm um perfil nutricional superior ao benchmark (ou best in class), de acordo com o país de operação.
JERÓNIMO MARTINS	F.JM20	Enquanto especialistas alimentares, trabalhamos todos os dias para continuarmos a merecer a preferência de milhares de portugueses, através de produtos de qualidade, inovadores, seguros e que sejam benéficos para a sua saúde. Conscientes das tendências de consumo atuais, a nossa responsabilidade estende-se também à oferta de soluções para consumidores com necessidades e ou preferências alimentares específicas como celíacos, vegans e vegetarianos, ou que procurem produtos sem gordura ou sem açúcares. A promoção de hábitos alimentares mais saudáveis e o papel ativo que queremos ter nas escolhas informadas dos nossos clientes são outras das nossas prioridades. Nos produtos de Marca Própria Pingo Doce, disponibilizamos informação clara e transparente. Para além da informação legal, como os alergénios ou ingredientes passíveis de intolerância, destacamos ainda informação nutricional relevante, como a presença de vitaminas e fibras ou os valores nutricionais por porção. Além dos rótulos são usados outros meios de comunicação como forma de contribuir para decisões informadas pelos nossos consumidores. Mesmo depois de chegarem às prateleiras, os produtos de marca Pingo Doce continuam a ser acompanhados e analisados, de modo a garantir a sua máxima qualidade e segurança. O acompanhamento destes produtos pressupõe ainda, sempre que possível, que sejam feitas melhorias ao seu perfil nutricional, que passam pela redução gradual do teor de sal, açúcar e gorduras – mantendo sempre o foco no sabor –, pela diminuição do uso de aditivos como corantes e conservantes; pela adequação do tamanho das porções e/ou pela inclusão de ingredientes como vitaminas e minerais. Promovemos a Dieta Mediterrânica como padrão alimentar de referência e desenvolvemos ações e programas em prol de hábitos alimentares mais saudáveis e estilos de vida ativos.
JERÓNIMO MARTINS	F.JM21	Promover a saúde pela alimentação Trabalhamos para oferecer diariamente produtos de qualidade, seguros, saudáveis, nutricionalmente equilibrados e diversificados. Disponibilizamos também alimentos que se adaptam aos consumidores com necessidades ou preferências específicas. A nossa estratégia assenta em: • apostar na qualidade e inovação das Marcas Próprias; • garantir a segurança alimentar; • prestar um serviço de confiança; • disponibilizar informação nutricional completa sobre os produtos comercializados.
JERÓNIMO MARTINS	F.JM22	Os nossos cincos pilares de responsabilidade: 1) promover a saúde pela alimentação; 2) respeitar o ambiente; 3) comprar com responsabilidade; 4) apoiar as comunidades envolventes; 5) ser um empregador de referência

LACTOGAL

Website	Code	CPA EXAMPLES
LACTOGAL	F.L1	Trabalhamos para o bem-estar através da alimentação.
LACTOGAL	F.L2	Estamos cá para continuar a trabalhar para o bem-estar através da alimentação. Estamos cá para, com a responsabilidade que nos caracteriza, manter o empenho em assegurar que os nossos produtos não faltem à mesa dos portugueses. Estamos cá, cientes das dificuldades com que nos deparamos, mas unidos pelo esforço e pela dedicação da nossa equipa que nos faz acreditar que é possível ultrapassar este desafio. Estamos cá porque os lacticínios são alimentos essenciais à saúde e ao bem-estar de todos e, por isso, não podemos parar.
LACTOGAL	F.L3	Na Lactogal, nunca esquecendo o nosso lema “Trabalhamos para o bem-alimentar”, mantemo-nos sempre alinhados pela nossa Missão que é fazer as pessoas sentirem-se bem ao longo da vida, alimentando-se com produtos pensados para uma alimentação equilibrada, onde o leite é determinante.
LACTOGAL	F.L4	A incomparável riqueza nutricional do leite distribui-se de diferentes formas pelos seus derivados. Pela sua riqueza, versatilidade e prazer que proporciona, o leite mantém viva a conclusão já registada por Hipócrates: "Leite é um alimento muito próximo da perfeição". O leite é um alimento de absoluta importância do ponto de vista nutricional porque se integra facilmente na alimentação diária e porque é um alimento versátil do ponto de vista físicoquímico. Além disso, apresenta a conjuntura ideal para ser adaptado de acordo com as necessidades e preferências individuais.
LACTOGAL	F.L5	A Lactogal assume o compromisso do desenvolvimento sustentável na sua atividade. O consumo de recursos naturais e de energia elétrica, o tratamento e a reutilização de águas residuais, a diminuição de produção de resíduos e um aumento da valorização são uma preocupação constante na atividade diária da Lactogal.
LACTOGAL	F.L6	Produtos responsáveis Ao tirar um produto da prateleira na loja, tem nas suas mãos mais do que possa pensar. Com a sua decisão de compra, pode contribuir para o desenvolvimento sustentável da sociedade. É por isso que criamos transparência e oferecemos produtos mais sustentáveis ao melhor preço.
LACTOGAL	F.L7	Apoio aos Produtores Nacionais Estamos a fazer Mais para apoiar os nossos fornecedores. Seja na certificação dos seus produtos, na exportação dos nossos artigos nacionais ou na melhoria dos seus padrões sociais, atuamos de forma transversal e justa para garantir a sua sustentabilidade a longo prazo.
LACTOGAL	F.L8	Estamos cá para continuar a trabalhar para o bem-estar através da alimentação. Estamos cá para, com a responsabilidade que nos caracteriza, manter o empenho em assegurar que os nossos produtos não faltem à mesa dos portugueses. Estamos cá, cientes das dificuldades com que nos deparamos, mas unidos pelo esforço e pela dedicação da nossa equipa que nos faz acreditar que é possível ultrapassar este desafio. Estamos cá porque os lacticínios são alimentos essenciais à saúde e ao bem-estar de todos e, por isso, não podemos parar.

LACTOGAL	F.L9	A Lactogal assume o compromisso do desenvolvimento sustentável na sua atividade. O consumo de recursos naturais e de energia elétrica, o tratamento e a reutilização de águas residuais, a diminuição de produção de resíduos e um aumento da valorização são uma preocupação constante na atividade diária da Lactogal.

LIDL

Website	Code	CPA EXAMPLES
LIDL	F.LI1	Mais para a Comunidade O nosso negócio marca a vida quotidiana de um grande número de pessoas. Como parceiro, empregador e ator social, temos uma responsabilidade a este nível. Por isso, em todas as áreas, estamos empenhados em assegurar boas condições de vida e de trabalho, bem como uma coexistência justa.
LIDL	F.LI2	Princípios Corporativos Sendo uma empresa representada internacionalmente, estamos conscientes da nossa dimensão e presença pública. Respeitamos a diversidade cultural e reconhecemos a variedade dos seus valores e das suas tradições. O nosso trabalho está caracterizado pelos nossos princípios da empresa e de conduta.
LIDL	F.LI3	MAIS PARA A COMUNIDADE – PARA UMA SOCIEDADE JUSTA! Do trabalhador do armazém ao agricultor, do responsável pelas compras ao colaborador na caixa – o Lidl marca a vida quotidiana de muitas pessoas em todo o mundo. É graças ao nosso compromisso diário que alcançamos a qualidade em que os nossos clientes confiam quando fazem as suas compras, tornando ainda mais importante considerar as necessidades das pessoas em tudo o que fazemos. Como empregador, parceiro de negócios e ator social, temos uma responsabilidade acrescida. Por isso, fazemos mais para a Comunidade. Queremos continuar a proporcionar aos nossos colaboradores o melhor ambiente de trabalho para a responsabilidade pessoal, igualdade de oportunidades, trabalho de equipa e alto desempenho. Na nossa cadeia de fornecimento, asseguramos o respeito pelos direitos humanos, assim como pelas normas laborais e de bem-estar animal. Além disso, sensibilizamos os nossos colaboradores e os nossos clientes a agirem de forma mais responsável e sustentável.
LIDL	F.LI4	apoio ao crescimento económico dos nossos parceiros promove a sua resiliência e capacidade produtiva, inovação e, consequentemente, a diferenciação do seu produto. Pretendemos apoiar a produção nacional e incentivar o seu crescimento no mercado interno e externo, assim como promover o desenvolvimento local, através de produtos DOP (Denominação de Origem Protegida) e IGP (Indicação Geográfica Protegida).
LIDL	F.LI5	Temos vindo a reforçar o nosso papel preponderante no crescimento da economia nacional, através da ajuda e impulso à exportação de produtos portugueses para os mercados onde estamos presentes, atuando como interlocutor e dando apoio em todas as etapas do processo de exportação, sendo atualmente a maior rede de distribuição na Europa.
LIDL	F.LI6	Queremos continuar a potenciar a exportação de produtos nacionais, abrindo os mercados externos aos nossos fornecedores e assumindo a posição de exportador sempre que existem produtos alimentares portugueses que não estão a ser absorvidos pelo mercado português.
LIDL	F.LI7	Mais para si Trabalhamos todos os dias para oferecer um sortido atrativo e mais sustentável aos nossos clientes. Promovemos também o consumo consciente e de uma dieta saudável com produtos adequados, apoiados por parceiros de certificação credíveis.
LIDL	F.LI8	MAIS LIDL – PARA UMA OFERTA MAIS SUSTENTÁVEL E MAIS SAUDÁVEL Menos açúcar, mais produtos biológicos e, numa palavra, mais qualidade. Trabalhamos todos os dias para cumprir os nossos próprios padrões de qualidade e os dos nossos clientes. Para o conseguirmos, contamos com produtos que não são apenas saborosos, mas também promovem a saúde e protegem o ambiente. Para o Lidl, assumir responsabilidade significa acompanhar os nossos clientes no caminho para um estilo de vida mais sustentável e mais saudável. Por isso, desenhamos o nosso sortido para ser mais para si. Com a nossa crescente seleção de produtos de alta qualidade e mais sustentáveis, permitimos-lhe tomar decisões de compra mais conscientes. Para o conseguirmos, contamos com certificações transparentes e credíveis, assim como rótulos de produtos que lhe fornecem orientação no momento da compra.
LIDL	F.LI9	Combinar valores e sucesso Enquanto multinacional, focamo-nos num modelo de negócio responsável e não apenas num objetivo financeiro. Simplicidade e foco no cliente, utilização responsável dos recursos e tratamento respeitoso de clientes, colaboradores e parceiros de negócios são o que guia as nossas ações diariamente. Estes valores estão definidos nos nossos princípios corporativos e de liderança, tal como no nosso código de conduta. No nosso trabalho diário, assumimos responsabilidade económica, social e ambiental. O Lidl Portugal foi, por exemplo, premiado pelo terceiro ano consecutivo "Retailer of the Year 20202021" em Portugal pela Grande Consumo, em consonância com o seu parceiro Q&A. Também as nossas marcas próprias têm sido distinguidas, sendo que, em 2023, as marcas Da Minha Terra (distinguida em duas categorias), Solevita, Fumadinho e Cien foram eleitas "Marca Nº 1", como ainda a Milbona "Boa Escolha" na "Escolha do Consumidor". Todos os nossos colaboradores - seja na sede, nos centros logísticos ou nas lojas - estão unidos por valores comuns e pela motivação de tornar o Lidl melhor todos os dias. Saiba mais sobre os nossos princípios corporativos aqui. Oferecemos aos nossos colaboradores um modelo de negócio promissor e a possibilidade de construirem a sua própria carreira de sucesso. Parcerias de confiança, justiça e respeito pela diversidade de culturas desempenham um papel importante neste processo. Trabalhamos continuamente para desenvolver os talentos dos nossos colaboradores, investindo na sua formação e desenvolvimento.
LIDL	F.LI10	Mais para o planeta", "Mais para a comunidade" e "Mais para si" descrevem a postura com que queremos estar. Com os nossos seis temas centrais - "Respeitar a biodiversidade", "Proteger o clima", "Conservar os recursos", "Agir de forma justa", "Promover a saúde" e "Envolver através do diálogo" - concretizamos esta promessa. A nível regional e global. Empenhados e conscientes.
LIDL	F.LI11	Os inúmeros prémios que recebemos em diversas áreas demonstram que estamos no bom caminho - desde o nosso compromisso enquanto empregador até à sustentabilidade e responsabilidade social. E estes não são casos isolados, como o demonstra o facto de termos sido nomeados, pelo terceiro ano consecutivo nas categorias supermercados e lojas físicas, “A Melhor Loja de Portugal” 2020-2021, e ainda distinguidos como “Retailer of The Year” 2020-202
LIDL	F.LI12	O Lidl leva a qualidade a sério. Qualidade esta confirmada pelos prémios que temos vindo a receber em toda a nossa oferta. Orgulhamo-nos dos nossos produtos Bio, Sem Lactose, Sustentável, Vegan e Clássico, premiados em 29 categorias, tendo sido a insígnia premiada em mais categorias, e da distinção recebida pelo Sabor do Ano 2023, em 32 dos nossos produtos. Também em 2023, as marcas Da Minha Terra, Solevita, Fumadinho e Cien foram distinguidas com o prémio Escolha do Consumidor.
LIDL	F.LI13	No ano de 2019, o grupo Lidl foi eleito pelo segundo ano consecutivo na categoria supermercados, “A Melhor Loja de Portugal”, com a loja de Alcobaça – Estrada de Leiria a alcançar o maior número de votos foi consagrado “A Melhor Loja de Portugal – Loja” e ainda distinguido como “Retailer of the Year 2019-2020”

LIDL	F.LI14	MAIS LIDL – PARA A PROTEÇÃO CLIMÁTICA!
LIDL	F.LI15	MAIS LIDL – PARA UMA UTILIZAÇÃO RESPONSÁVEL DOS RECURSOS NATURAIS!
LIDL	F.LI16	A natureza assegura o sustento das gerações futuras, a qualidade e a disponibilidade dos nossos produtos. Por isso, estamos empenhados na proteção do ambiente – para o nosso clima, para a biodiversidade e para a utilização consciente dos recursos naturais.
LIDL	F.LI17	Assumimos o nosso dever social e ecológico quer na nossa própria empresa, quer nas relações com os nossos parceiros comerciais.
LIDL	F.LI18	MAIS LIDL – PARA UM PLANETA COM FUTURO O comércio de produtos alimentares e de mercadorias deve assentar em práticas com o menor impacto possível para o ambiente. Só assim conseguimos garantir a qualidade e a disponibilidade dos nossos produtos a longo prazo. Ao mesmo tempo, sabemos que os nossos bens comerciais têm um impacto no nosso planeta – desde a extração de matérias-primas e a sua transformação posterior em produtos até ao transporte, ao consumo e à eliminação e gestão dos resíduos gerados. A nossa responsabilidade resulta daqui: Faremos mais para o planeta. Só então poderemos oferecer aos nossos clientes um sortido atrativo a longo prazo e assegurar também a nossa viabilidade futura. Contribuímos para a proteção do ambiente com objetivos e medidas concretas. Estes incluem a utilização racional dos recursos naturais, a implementação de medidas de proteção climática ao longo de toda a cadeia de valor e a contribuição ativa para a proteção dos ecossistemas através das nossas atividades comerciais.
LIDL	F.LI19	Ao tirar um produto da prateleira na loja, tem nas suas mãos mais do que possa pensar. Com a sua decisão de compra, pode contribuir para o desenvolvimento sustentável da sociedade. É por isso que criamos transparência e oferecemos produtos mais sustentáveis ao melhor preço.
LIDL	F.LI20	Reforçamos as nossas parcerias com fornecedores portugueses segundo três eixos: desenvolvimento económico, exportação e certificação. Com este apoio, garantimos a sustentabilidade da produção nacional e a qualidade dos nossos produtos.
LIDL	F.LI21	No âmbito da nossa política de gestão de resíduos, temos como missão gerir todos os resíduos produzidos pela operação do Lidl de forma adequada, privilegiando, sempre que possível, a sua valorização e a identificação de alternativas que promovam a economia circular. Os principais resíduos do Lidl são: papel e cartão, plástico, metais, madeiras, equipamentos elétricos e eletrónicos fora de uso e resíduos indiferenciado
LIDL	F.LI22	Mais para o planeta", "Mais para a comunidade" e "Mais para si" descrevem a postura com que queremos estar. Com os nossos seis temas centrais - "Respeitar a biodiversidade", "Proteger o clima", "Conservar os recursos", "Agir de forma justa", "Promover a saúde" e "Envolver através do diálogo" - concretizamos esta promessa. A nível regional e global. Empenhados e conscientes.
LIDL	F.LI23	Combinar valores e sucesso Enquanto multinacional, focamo-nos num modelo de negócio responsável e não apenas num objetivo financeiro. Simplicidade e foco no cliente, utilização responsável dos recursos e tratamento respeitoso de clientes, colaboradores e parceiros de negócios são o que guia as nossas ações diariamente. Estes valores estão definidos nos nossos princípios corporativos e de liderança, tal como no nosso código de conduta. No nosso trabalho diário, assumimos responsabilidade económica, social e ambiental. O Lidl Portugal foi, por exemplo, premiado pelo terceiro ano consecutivo "Retailer of the Year 20202021" em Portugal pela Grande Consumo, em consonância com o seu parceiro Q&A. Também as nossas marcas próprias têm sido distinguidas, sendo que, em 2023, as marcas Da Minha Terra (distinguida em duas categorias), Solevita, Fumadinho e Cien foram eleitas "Marca Nº 1", como ainda a Milbona "Boa Escolha" na "Escolha do Consumidor". Todos os nossos colaboradores - seja na sede, nos centros logísticos ou nas lojas - estão unidos por valores comuns e pela motivação de tornar o Lidl melhor todos os dias. Saiba mais sobre os nossos princípios corporativos aqui. Oferecemos aos nossos colaboradores um modelo de negócio promissor e a possibilidade de construirem a sua própria carreira de sucesso. Parcerias de confiança, justiça e respeito pela diversidade de culturas desempenham um papel importante neste processo. Trabalhamos continuamente para desenvolver os talentos dos nossos colaboradores, investindo na sua formação e desenvolvimento.
LIDL	F.LI24	Os inúmeros prémios que recebemos em diversas áreas demonstram que estamos no bom caminho - desde o nosso compromisso enquanto empregador até à sustentabilidade e responsabilidade social. E estes não são casos isolados, como o demonstra o facto de termos sido nomeados, pelo terceiro ano consecutivo nas categorias supermercados e lojas físicas, "A Melhor Loja de Portugal" 2020-2021, e ainda distinguidos como "Retailer of The Year" 2020-202
LIDL	F.LI25	O Lidl leva a qualidade a sério. Qualidade esta confirmada pelos prémios que temos vindo a receber em toda a nossa oferta. Orgulhamo-nos dos nossos produtos Bio, Sem Lactose, Sustentável, Vegan e Clássico, premiados em 29 categorias, tendo sido a insígnia premiada em mais categorias, e da distinção recebida pelo Sabor do Ano 2023, em 32 dos nossos produtos. Também em 2023, as marcas Da Minha Terra, Solevita, Fumadinho e Cien foram distinguidas com o prémio Escolha do Consumidor.
LIDL	F.LI26	Promoção do Bem. O Lidl mobilizou os consumidores para escolher as suas compras de forma responsável, apoiando a concretização de projetos de solidariedade social. Com a ajuda de todos os promotores do bem que compraram o azeite, o açúcar, o bolo rei e o café solidários, o Lidl entregou, em 2017, 406 mil euros a cinco IPSS, um valor superior ao conseguido no ano anterior (368 mil euros divididos pelas IPSS APAV, ACAPO, Acreditar, Rutis e APEXA), o que refletiu uma maior disposição dos portugueses para ajudar o próximo.

MCDONALDS

Website	Code	CPA EXAMPLES
MCDONALDS	F.MC1	O conhecimento sobre Portugal e as aprendizagens que temos feito ao longo dos anos, com as comunidades das quais fazemos parte, valorizam o património, e a história que escrevemos e dão-nos pistas para o futuro.
MCDONALDS	F.MC2	Atualmente, dispomos de uma oferta alargada e diversificada com mais de 100 produtos , disponíveis , para ir ao encontro das necessidades e expetativas do nosso consumidor.
MCDONALDS	F.MC3	A integração de frutas e vegetais demonstra a preocupação com a oferta de uma variedade que permita a adultos e crianças escolherem o que preferirem, através de diversas alternativas na combinação dos menus:
MCDONALDS	F.MC4	Estar e cuidar como um bom vizinho apoiando as comunidades está no nosso ADN , , e é para nós uma prioridade e um compromisso.
MCDONALDS	F.MC5	Usando a nossa continuar a investir e escala e responsabilidade contribuir para impactar , queremos positivamente a sociedade , procurando fazer a diferença em todas as comunidades locais onde estamos integrados.
MCDONALDS	F.MC6	Sabemos que o nosso negócio só pode prosperar se as comunidades também prosperarem e ser um bom vizinho é para nós fundamental .

MCDONALDS	F.MC7	A McDonald's faz parte de muitas comunidades em todo o mundo e, com isso, surge a responsabilidade de ajudar a apoiar as pessoas e comunidades, especialmente em momentos de necessidade. Pensar globalmente e agir localmente não é apenas a coisa certa a fazer, ajuda-nos a estarmos ligados com os nossos clientes, colaboradores e com a sociedade em geral.
MCDONALDS	F.MC8	Oferecemos uma desenvolvimento das nossas "formação para a vida" que as prepara para um futuro dentro ou fora da empresa.
MCDONALDS	F.MC9	A McDonald's é uma porta aberta a todos. Promovemos locais de trabalho inclusivos, onde todos se sintam seguros, respeitados e valorizados
MCDONALDS	F.MC10	Estamos aqui para sustentável para as comunidades do presente e do futuro. cuidar, preservar e escrever uma história mais
MCDONALDS	F.MC11	Não é por acaso que somos vizinhos aqui. Aqui é onde queremos estar. É onde pertencemos. O nosso lugar é aqui. Junto de quem nos procura pelos bons momentos que servimos. Mas também de quem beneficia por aquilo que fazemos. O nosso propósito é fazer bem. Fazer bem a quem também está aqui. Às pessoas, às famílias e à comunidade deste lugar. De cada lugar. Estamos aqui para fazer bem aos produtores nacionais. Para dar valor e futuro a quem nos fornece, diariamente, qualidade e confiança. Estamos aqui para fazer bem às pessoas e ao emprego. Para incluir, formar e desenvolver quem connosco quer fazer bem. Estamos aqui para fazer bem a quem faz o bem. Para nutrir e apoiar iniciativas que melhoram a vida de muitos. E estamos aqui para fazer bem a este lugar, como fazem os bons vizinhos. Para cuidar e preservar. Para escrever uma história mais sustentável. O que é importante aqui é importante para nós. É isso que nos leva a retribuir aqui tudo o que aqui nos dão. Porque mais do que aqui estar, é aqui que queremos viver. McDonald's. Aqui para fazer bem.
MCDONALDS	F.MC12	Procuramos sempre mais sustentabilidade no que oferecemos. Sabemos que as tendências alimentares estão em constante evolução e que os nossos consumidores procuram produtos de elevada qualidade, mas também de variedade. Por isso, temos vindo a trabalhar, interna e externamente, para promover uma cadeia de valor cada vez mais sustentável.

MERCADONA

Website	Code	CPA EXAMPLES
MERCADONA	F.M1	"As empresas e a sociedade não podem viver de costas voltadas. É nossa obrigação contribuir para o seu bem-estar, para que esta valorize a necessidade de ter mais e melhores empresas todos os dias"
MERCADONA	F.M2	A Mercadona mantém ativo um Plano de Ação Social próprio com o qual, ano após ano, reforça os seus vínculos com a sociedade através de valores que considera irrenunciáveis, como o diálogo, a transparência e a escuta ativa. Com estes valores, constrói relações estáveis nas zonas em que está presente, aposta na proximidade e estreita os vínculos para detetar as suas necessidades específicas e oferecer soluções concretas, fruto do consenso e do entendimento mútuo.
MERCADONA	F.M3	A Mercadona tem consciência de que cada vez mais pessoas investem numa alimentação saudável, motivo pelo qual tenta ajudar os seus "Chefes" com um sortido baseado no seu critério de qualidade de estar bom e ser bom. Em 2023, continuou a melhorar e a introduzir produtos indispensáveis que, quando bem combinados e adicionados a bons hábitos diários, são a chave para uma alimentação saudável, rica, variada e equilibrada.
MERCADONA	F.M4	Para proporcionar aos seus "Chefes" a possibilidade de elaborar uma dieta equilibrada, além da variedade do seu sortido, dos conselhos, das receitas e da própria prescrição dos colaboradores, a empresa introduziu melhorias na rotulagem que contribuem para esse mesmo fim.
MERCADONA	F.M5	A Mercadona mantém um compromisso constante com os ambientes em que está presente e promove, ano após ano, iniciativas sociais, ambientais e de governação responsáveis que têm um impacto positivo na sua atividade.
MERCADONA	F.M6	O compromisso com o bem-estar animal da Mercadona vê-se igualmente refletido na responsabilidade que os seus fornecedores assumem nesta matéria
MERCADONA	F.M7	Consciente de que a agricultura sustentável é uma necessidade ética, social, económica e ambiental irrenunciável, colabora estreitamente com os fornecedores deste setor, não só para garantir a segurança alimentar nestes produtos, mas também para promover ecossistemas saudáveis e apoiar a gestão sustentável da terra, da água e dos recursos naturais
MERCADONA	F.M8	A Mercadona promove o uso consciente dos recursos naturais e estabelece, através do seu Sistema de Gestão Ambiental, políticas e estratégias responsáveis que reforçam, ano após ano, a sustentabilidade da empresa. Desta forma, a gestão ambiental gira à volta de três eixos principais: o uso da energia, o uso da água e a proteção da biodiversidade.
MERCADONA	F.M9	ENCHER A BARRIGA "Prescrever ao consumidor final produtos / soluções que satisfaçam as suas necessidades de comida, bebida, cuidados pessoais, cuidados do lar e cuidados dos animais… ... garantindo sempre qualidade efetiva (seguro e saudável), máximo serviço (sustentável), mínimo orçamento e mínimo tempo."
MERCADONA	F.M10	A Mercadona realiza importantes esforços para impulsionar um modelo de gestão baseado na produção e no consumo responsáveis, que passa pela otimização dos seus processos, maximizando o valor dos produtos, materiais e recursos, com o objetivo de os poder manter no circuito da economia durante o maior tempo possível e, assim, garantir o seu máximo aproveitamento. Também tem o objetivo de reduzir a geração de resíduos sempre que for possível. E quando não é possível, procura soluções diferenciadoras e inovadoras que permitam recuperá-los e voltar a introduzi-los no ciclo produtivo.
MERCADONA	F.M11	Mercadona fatura 31.041 milhões, mais 11%, e in\ação reduziu a margem e a rentabilidade

NESTLÉ

Website	Code	CPA EXAMPLES
NESTLÉ	F.N1	A Nestlé está em Portugal desde 1923, fazendo parte da vida dos portugueses há 100 anos. Com mais de 90 marcas e produtos para todas as idades e etapas da vida e com o propósito de desenvolver o poder da alimentação para melhorar a qualidade de vida de todos, hoje e para as gerações futuras.
NESTLÉ	F.N2	"2023 é o ano do centenário. 100 anos a fazer parte da casa e dos corações dos Portugueses"
NESTLÉ	F.N3	Estamos a esforçar-nos por criar em conjunto um futuro resiliente para o nosso planeta e as pessoas que nele habitam. É por isso que nos comprometemos a fazer avançar sistemas alimentares regenerativos em grande escala.
NESTLÉ	F.N4	A nossa ambição é ajudar 50 milhões de crianças a terem uma vida mais saudável.
NESTLÉ	F.N5	Acreditamos no poder da alimentação para melhorar a qualidade de vida. Uma boa alimentação nutre e delicia os sentidos. Ajuda as crianças a crescerem saudáveis, os animais de companhia a desenvolveremse, os pais a envelhecerem de forma saudável e todos a desfrutarem a vida de forma plena. Uma boa alimentação respeita igualmente o nosso

		planeta e protege os recursos para as futuras gerações. Mas as mudanças são cada vez mais rápidas e sabemos que o que hoje é bom não será bom o suficiente no futuro. Considere o desafio de satisfazer as necessidades nutricionais de 10 mil milhões de pessoas até 2050, de forma responsável e sustentável. Irá exigir mudanças significativas e algumas outras, quiçá, radicais. Na Nestlé, exploramos constantemente e desejamos alargar os limites do possível quando falamos de alimentos, bebidas e soluções nutricionais para a saúde, com o objetivo de melhorar a qualidade de vida e contribuir para um futuro mais saudável.
NESTLÉ	F.N6	A Nestlé é a maior Companhia de alimentação e bebidas ao nível mundial. Há mais de 150 anos que aplicamos o nosso conhecimento em Nutrição, Saúde e Bem-Estar para ajudar as pessoas, famílias e animais de companhia a viver vidas mais felizes e saudáveis. Desenvolvemos o poder da alimentação para melhorar a qualidade de vida de todos, hoje e para as gerações futuras. Em busca do prazer dos alimentos. Em busca de uma saúde melhor. Em busca de uma nutrição de qualidade e acessível a todos. Em busca de uma preparação de alimentos e bebidas cada vez mais fácil. Em busca da proteção e melhoria dos nossos recursos naturais.
NESTLÉ	F.N7	Há mais de 150 anos que aplicamos o nosso conhecimento em Nutrição, Saúde e Bem-Estar para ajudar as pessoas, famílias e animais de companhia a viver vidas mais felizes e saudáveis. Desenvolvemos o poder da alimentação para melhorar a qualidade de vida de todos, hoje e para as gerações futuras. Em busca do prazer dos alimentos. Em busca de uma saúde melhor. Em busca de uma nutrição de qualidade e acessível a todos. Em busca de uma preparação de alimentos e bebidas cada vez mais fácil. Em busca da proteção e melhoria dos nossos recursos naturais.
NESTLÉ	F.N8	A Nestlé está em Portugal desde 1923, fazendo parte da vida dos portugueses há 100 anos. Com mais de 90 marcas e produtos para todas as idades e etapas da vida e com o propósito de desenvolver o poder da alimentação para melhorar a qualidade de vida de todos, hoje e para as gerações futuras.
NESTLÉ	F.N9	A forma de atuar da Nestlé privilegia três áreas de impacto: Pessoas, Famílias e Animais de Companhia Pessoas e Comunidades , Pessoas e o Planeta. É através destas três áreas que a Nestlé partilha valor ao longo da sua cadeia de produção, desde a matéria-prima , ao consumidor final. Com uma estratégia de longo prazo, a Nestlé tem o seu foco em satisfazer as necessidades dos consumidores, centrada no respeito pelo futuro e sempre alicerçada em fortes compromissos com a sustentabilidade ambiental de todas as operações.
NESTLÉ	F.N10	Nutrição, saúde e bem-estar Guiados pelo nosso compromisso com a Nutrição, Saúde e Bem-estar, o nosso objetivo é fornecer as escolhas mais saborosas e saudáveis em todas a nossas categorias de produto. Trabalhamos para aumentar o valor nutricional dos nossos produtos. Esforçamo-nos por melhorar a saúde geral e a acessibilidade do nosso portfólio. As nossas capacidades de inovação estabelecidas desde há longo tempo ajudam-nos a inovar rápida e agilmente. Estamos comprometidos em fornecer informação nutricional transparente e em encorajar estilos de vida saudáveis. Expressamo-lo por meio da nossa assinatura “Somos a Nestlé, the Good food, Good life company”.
NESTLÉ	F.N11	A nossa comunicação ambiciona ter um impacto positivo na vida dos consumidores. Estamos comprometidos com uma comunicação comercial de confiança que permita que os consumidores façam escolhas informadas e que promova dietas mais saudáveis. Representamos os alimentos de forma a não encorajar o sobreconsumo. As nossas alegações de saúde são cientificamente suportadas. A nossa comunicação dirigida a crianças promove estilos de vida saudáveis e atividade física.
NESTLÉ	F.N12	Estamos comprometidos em, através de práticas de comunicação comercial responsáveis, comunicar às crianças apenas escolhas que as ajudem a obter uma dieta nutritiva.
NESTLÉ	F.N13	Acreditamos no poder da alimentação para melhorar a qualidade de vida. É essa crença que alimenta o nosso compromisso de usar a nossa escala global, os nossos recursos e a nossa experiência para contribuir para um futuro mais saudável para as pessoas e para o planeta.
NESTLÉ	F.N14	A iniciativa Nestlé por Crianças mais Saudáveis reúne todos os esforços para apoiar os pais e cuidadores na “jornada” de criar crianças saudáveis. Em todo o mundo, a Nestlé está a lançar mais alimentos e bebidas nutritivos para dar resposta às necessidades nutricionais das crianças, enquanto promove a educação alimentar através de programas escolares, bem como dicas práticas para o dia a dia e receitas saudáveis para os pais.
NESTLÉ	F.N15	A nutrição é a chave para assegurar o desenvolvimento saudável das crianças. Contudo, como sempre, a teoria é bem diferente da prática. Encorajar as crianças a comer mais vegetais e frutas, assegurando que as suas refeições sejam tão nutritivas quanto possível, e incorporando diferentes fontes de proteína – incluindo opções de origem vegetal – pode, por vezes, ser um desafio para os pais e cuidadores.
NESTLÉ	F.N16	Na Nestlé, acreditamos no poder da alimentação para melhorar a qualidade de vida de todos. Orientados por este propósito, procuramos constantemente ultrapassar os
NESTLÉ	F.N17	A nossa ambição para 2030 é atingir zero impacte ambiental nas nossas operações.
NESTLÉ	F.N18	Estamos empenhados em implementar práticas agrícolas sustentáveis que contribuem para uma produção resiliente a longo prazo, proporcionando meios de subsistência sustentáveis, redução de emissões de gases com efeito de estufa, proteção da biodiversidade e conservação e reposição dos recursos naturais. Acreditamos que os agricultores devem auferir um nível de rendimento suficiente que lhes permita manter um nível de vida normal.
NESTLÉ	F.N19	(…) comprometemo-nos com práticas comerciais sustentáveis ao longo de toda a nossa cadeia de valor. Inspirados pela natureza, estamos comprometidos em desenvolver uma economia circular através do desenvolvimento de materiais de embalagem recicláveis ou reutilizáveis, reduzindo as perdas e o desperdício de alimentos e explorando modelos circulares de negócio.
NESTLÉ	F.N20	A ameaça das alterações climáticas e ambientais, como a degradação das florestas, da terra, do solo e das águas, está a afetar os agricultores e as comunidades. Esta ameaça põe as condições de vida dos agricultores em risco e prejudica a acessibilidade e a disponibilidade de alimentos de qualidade. Temos de ajudar a proteger e a restaurar os recursos do nosso planeta. Isto exige uma mudança transformadora. É por esse motivo que a Nestlé está a embarcar numa jornada rumo à regeneração, para ajudar a proteger, renovar e restaurar o meio ambiente, melhorar os meios de subsistência dos agricultores e aumentar a resiliência e o bem-estar das comunidades e dos nossos consumidores. Acreditamos que cada um de nós tem um papel a desempenhar para ajudar a proteger o nosso futuro. Independentemente da idade, género, nacionalidade ou origem - juntos, podemos fazer a diferença. Tanto hoje como para as gerações futuras. A agricultura regenerativa é o nosso primeiro passo, mas não vamos ficar por aqui.
NESTLÉ	F.N21	As alternativas proteicas de origem vegetal estão a mudar a forma como vemos a alimentação! Agora já existem hambúrgueres à base de soja, alternativas vegetais ao peixe e marisco constituídas, por exemplo, por proteína de ervilha e bebidas vegetais, que podem ser o complemento perfeito para o seu café ou para a sua taça de cereais. Escolher estas alternativas pode ser o início de uma jornada rumo a uma alimentação mais sustentável.

NESTLÉ	F.N22	No centro de uma boa alimentação está a qualidade dos ingredientes, as pessoas que os produzem e os solos e os ecossistemas onde são cultivados. Proteger os produtores e o ambiente onde trabalham garante o sucesso de longo prazo dos negócios da Nestlé.
NESTLÉ	F.N23	Em Portugal, a Nestlé conta com mais de 900 fornecedores locais das mais variadas áreas de atividade, sendo esta também uma forma de criar e partilhar valor com a sociedade portuguesa, de apoiar a resiliência do tecido económico nacional e das comunidades onde as empresas estão inseridas.
NESTLÉ	F.N24	A nossa comunicação dirigida a crianças promove estilos de vida saudáveis e atividade física
NESTLÉ	F.N25	Estamos comprometidos em, através de práticas de comunicação comercial responsáveis, comunicar às crianças apenas escolhas que as ajudem a obter uma dieta nutritiva.
NESTLÉ	F.N26	O nosso objetivo é providenciar aos consumidores informação nutricional transparente e fiável, através de rotulagem baseada em ciência e num formato que os ajude a realizarem escolhas informadas sobre os
NESTLÉ	F.N27	Envolver as crianças na preparação das refeições leva-as a adotar hábitos alimentares mais saudáveis – em suma, a qualidade da sua alimentação é superior, tornando-as mais aptas a comer frutas e vegetais. É por isso que incentivamos e advogamos a partilha do momento da preparação e confeção das refeições, pelos pais e crianças.
NESTLÉ	F.N28	iniciativa Nestlé por Crianças mais Saudáveis reúne todos os esforços para apoiar os pais e cuidadores na "jornada" de criar crianças saudáveis. Em todo o mundo, a Nestlé está a lançar mais alimentos e bebidas nutritivos para dar resposta às necessidades nutricionais das crianças, enquanto promove a educação alimentar através de programas escolares, bem como dicas práticas para o dia a dia e receitas saudáveis para os pais.
NESTLÉ	F.N29	ALIMENTAR O CONHECIMENTO É SAUDÁVEL.
NESTLÉ	F.N30	A nossa comunicação ambiciona ter um impacto positivo na vida dos consumidores. Estamos comprometidos com uma comunicação comercial de confiança que permita que os consumidores façam escolhas informadas e que promova dietas mais saudáveis. Representamos os alimentos de forma a não encorajar o sobreconsumo. As nossas alegações de saúde são cientificamente suportadas. A nossa comunicação dirigida a crianças promove estilos de vida saudáveis e atividade física

NUTRIENVESTE

Website	Code	CPA EXAMPLES
NUTRIENVESTE SOVENA	F.NU1	Crescer com um propósito é alimentar o futuro. A nossa estratégia FEEDING FUTURES FOR A PLANET THAT PROSPERS AND FOR PEOPLE THAT THRIVE assenta no nosso propósito e impulsiona a Sovena para uma melhoria constante. Promover uma alimentação cada vez mais diversificada e sustentável através da qualidade e inovação dos nossos produtos, da utilização de uma agricultura que proteja a natureza e de uma transformação eficiente e circular. Promovemos uma equipa de funcionários empenhados e conectados, uma comunidade e consumidores informados acerca de dietas diversas. A nossa mais recente avaliação de materialidade deu lugar à definição da nossa estratégia, estruturada em cinco compromissos e com dois potenciadores fundamentais que garantem o nosso sucesso: os mais elevados padrões de QUALIDADE e SEGURANÇA ALIMENTAR e uma forte cultura de INOVAÇÃO e DESENVOLVIMENTO.
NUTRIENVESTE SOVENA	F.NU2	A No s s a E s s ê n c i a Vamos continuar a cultivar. A nossa natureza é plantar possibilidades que dão frutos. Criamos laços entre a terra e a mesa, entre aqueles que cultivam e aqueles que comem. Preferimos reinventar antes de inventar, sabendo que às vezes, "trazer de volta" é a atitude mais revolucionária. Fazemos a economia circular girar e escolhemos ser diretos em relação à ordem natural das coisas. Somos aqueles que perseguem as memórias complexas, harmoniosas e duradouras, que podem ser saboreadas. Celebramos a tolerância alimentar e trazemos as discussões para cima da mesa. Insistimos em acrescentar valor à cadeia, com decisões sustentadas e sustentáveis. Procuramos o equilíbrio para todos, planeta Terra incluído. E cultivamos alimentos, juntamente com um compromisso: Após mais de cem anos a crescer, queremos alimentar os próximos cem.
NUTRIENVESTE SOVENA	F.NU3	Da terra para o lagar, do lagar para o prato, conseguimos desenvolver ciclos de produção completos e sustentados.
NUTRIENVESTE SOVENA	F.NU4	Começamos o nosso caminho de transformação aqui e não deixamos os nossos créditos por mãos alheias. Acreditamos que um bom alimento será sempre - e cada vez mais - valorizado. Fornecemos sementes para a plantação das oleaginosas e compramos sementes no final da colheita para a produção e refinação dos óleos.
NUTRIENVESTE SOVENA	F.NU5	Oferecemos qualidade, embalagens inovadoras e funcionais para diferentes segmentos e variedades adaptadas às exigências alimentares e de saúde. Estamos também atentos às tendências e hábitos de consumo de diferentes culturas, o que nos permite renovar de forma contínua a nossa oferta, aumentando a nossa relevância.
NUTRIENVESTE SOVENA	F.NU6	Queremos crescer além daquilo que somos hoje e preservar o que sempre fizemos bem, para sermos mais relevantes amanhã. Continuaremos a produzir bons alimentos sem truques. Depois de 100 anos de crescimento, chegou a altura de alimentarmos os próximos 100. É por isso que desenvolvemos as nossas atividades de forma responsável, assegurando as últimas certificações de segurança alimentar, aplicando as melhores práticas de sustentabilidade e reforçando a nossa aposta na investigação, desenvolvimento e inovação. É também por isso que atuamos com responsabilidade ambiental, reduzindo o impacto das atividades que desenvolvemos, através de uma gestão eficiente da energia, dos resíduos e subprodutos que geramos. E, finalmente, é por isso que promovemos a saúde e nutrição junto das nossas pessoas, inclusive as que trabalham conosco para que o sabor e saúde cheguem a todas as mesas.
NUTRIENVESTE SOVENA	F.NU7	A relação do nosso Grupo com o seu meio envolvente, seja ele ecológico ou humano, faz-se de forma responsável e ética, tal como indicado no Código de Ética e Conduta do Grupo Sovena, que rege a nossa atuação em toda a cadeia de fornecimento, tendo em conta todas as partes interessadas, colaboradores, fornecedores, clientes e comunidade.
NUTRIENVESTE SOVENA	F.NU8	O Bom, Pela Terra" é a assinatura do novo posicionamento de Oliveira da Serra. O novo posicionamento surge para assinalar o início de uma nova era, que pretendemos ser pautada por um ciclo de mudanças. Este não é um novo propósito, é sim o despertar da razão de existir de Oliveira da Serra, porque sempre fomos da terra e para a Terra. O nosso objetivo é criar impacto positivo em todo o ciclo da alimentação, compromisso assente na assinatura "O Bom, pela Terra".

PEPSICO

Website	Code	CPA EXAMPLES

PEPSICO	F.P1	A PEPSICO ESTAMOS CENTRADOS EM LIBERTAR TODO O NOSSO POTENCIAL PARA UM CRESCIMENTO ACELERADO E SUSTENTÁVEL, ADOTANDO UMA NOVA MISSÃO E VISÃO QUE DENOMINAMOS WINNING WITH PURPOSE
PEPSICO	F.P2	Criando momentos felizes com os nossos deliciosos produtos e experiências únicas junto das nossas marcas. PARA OS NOSSOS CONSUMIDORES: PARA OS NOSSOS CLIENTES: Ao sermos o melhor parceiro possível, impulsionando a inovação e proporcionando um nível de crescimento incomparável na nossa indústria. PARA OS NOSSOS PARCEIROS E PARA AS NOSSAS COMUNIDADES: Criando novas oportunidades de trabalho, proporcionando novas competências e construindo em conjunto carreiras de sucesso num local de trabalho diverso e inclusivo. PARA O NOSSO PLANETA: Conservando os recursos valiosos da nossa natureza e promovendo um planeta mais sustentável para as gerações futuras. PARA OS NOSSOS ACCIONISTAS: Distribuindo retorno do investimento e adotando a melhor gestão corporativa possível.
PEPSICO	F.P3	Integrando propósito na nossa estratégia de negócio e fazendo ainda mais pelo planeta e pelas nossas pessoas. Aproveitando a nossa escala, alcance e experiência nas áreas onde podemos ter um maior impacto como a Agricultura, Água, Embalagens, Produtos, Clima e Pessoas, para ajudar assim a construir um Sistema Alimentar mais sustentável.
PEPSICO	F.P4	Atualmente, o sistema alimentar global contribui com nutrição, oportunidade económica, comodidade e lazer para mais pessoas do que nunca, mas enfrenta pressões significativas que ameaçam a sua capacidade futura para ir ao encontro das necessidades de uma população global em crescimento. Na PepsiCo, acreditamos que há uma oportunidade de mudar a forma como o mundo produz, distribui, consome e descarta alimentos e bebidas, a fim de enfrentar os desafios partilhados que enfrentamos.
PEPSICO	F.P5	Pretendemos utilizar a nossa escala, alcance e expertise para ajudar a construir um sistema alimentar mais sustentável; um sistema que possa atender às necessidades humanas de nutrição e prazer, e continuar a impulsionar o desenvolvimento económico e social, sem exceder os limites natural do planeta.
PEPSICO	F.P6	À medida que concentramos os nossos esforços em tornar-nos uma empresa Melhor, ajudamos a cultivar este potencial em todo o mundo, liderando o caminho para um sistema alimentar mais sustentável, que passa por investir numa nutrição sustentável, no acesso a água potável, numa gestão eficaz de resíduos e no empoderamento das mulheres.
PEPSICO	F.P7	A água potável é fundamental para a dignidade humana, saúde e prosperidade económica. Também é essencial para construir um sistema alimentar mais sustentável.
PEPSICO	F.P8	Ao longo de décadas a colaborar com parceiros locais e a investir para fazer a diferença, através da PepsiCo Foundation, observámos o incrível potencial das comunidades. À medida que concentramos os nossos esforços em tornar-nos uma empresa Melhor, ajudamos a cultivar este potencial em todo o mundo, liderando o caminho para um sistema alimentar mais sustentável, que passa por investir numa nutrição sustentável, no acesso a água potável, numa gestão eficaz de resíduos e no empoderamento das mulheres.
PEPSICO	F.P9	Para que o nosso sistema alimentar se torne mais sustentável a longo prazo, devemos trabalhar em conjunto para combater a atual crise de plásticos, de forma a responder às necessidades da comunidade e maximizar os resultados sociais positivos.
PEPSICO	F.P10	A nutrição é a base de um sistema alimentar mais sustentável. Com uma alimentação correta, as comunidades florescem: as crianças recebem uma melhor educação, as economias crescem, as comunidades prosperam e as pessoas experienciam vidas melhores e mais saudáveis.
PEPSICO	F.P11	Atualmente, o sistema alimentar global contribui com nutrição, oportunidade económica, comodidade e lazer para mais pessoas do que nunca, mas enfrenta pressões significativas que ameaçam a sua capacidade futura para ir ao encontro das necessidades de uma população global em crescimento. Na PepsiCo, acreditamos que há uma oportunidade de mudar a forma como o mundo produz, distribui, consome e descarta alimentos e bebidas, a fim de enfrentar os desafios partilhados que enfrentamos. Pretendemos utilizar a nossa escala, alcance e expertise para ajudar a construir um sistema alimentar mais sustentável; um sistema que possa atender às necessidades humanas de nutrição e prazer, e continuar a impulsionar o desenvolvimento económico e social, sem exceder os limites natural do planeta. Além de ser a coisa certa a fazer, trabalhar para realizar esse propósito também é positivo para o nosso negócio. Os locais onde o nosso sistema alimentar possa estar a funcionar de forma pouco sustentável, pode criar riscos financeiros, operacionais para a nossa empresa, assim como para as cadeias de fornecimento. Em contrapartida, abordar os riscos dentro da nossa cadeia de valor e um sistema alimentar mais vasto podem efetivamente criar novas oportunidades para uma vantagem competitiva e futuro crescimento no mercado, apoiando simultaneamente as comunidades em que operamos

SONAE

Website	Code	CPA EXAMPLES
SONAE	F.S1	Crescer em Comunidade
SONAE	F.S2	O Objetivo é reduzir o impacto dos excedentes alimentares de loja através da criação de um mercado de economia circular que dinamize o processo de doações a parceiros sociais e de vendas a negócios circundantes.
SONAE	F.S3	Há mais de uma década que o Continente foi pioneiro em Portugal ao adotar este sistema de informação nutricional nos seus produtos de marca própria. A Missão Continente revolucionou, assim, a leitura dos produtos. Indo ao encontro de um consumidor cada vez mais atento e exigente, o Semáforo Nutricional representa hoje, para muitos dos clientes, um critério decisivo no momento da compra.
SONAE	F.S4	O Continente continua particularmente focado na adoção de hábitos alimentares equilibrados e na meta de redução do consumo excessivo de sal, açúcares e gorduras, em conjunto com os seus parceiros, sejam Fornecedores, Centros de Investigação, Universidades e até com os próprios Clientes, que são convidados a testar produtos antes do seu lançamento.
SONAE	F.S5	Ao longo dos últimos anos, seja através das suas próprias iniciativas, seja através da participação em campanhas e projetos externos, a Missão Continente tem alinhado a sua atuação com a agenda do Desenvolvimento Sustentável.
SONAE	F.S6	Procuramos desenvolver um futuro mais justo, feliz e sustentável, assente em três grandes eixos de atuação – Alimentação, Pessoas e Planeta.
SONAE	F.S7	Desde cedo assumimos um papel transformador na sociedade, procurando sempre encontrar as melhores formas de o fazer, sem nunca esquecer a conjuntura e a resposta a situações de emergência social nacional com que nos vamos deparando José Fortunato, Presidente da Missão Continente
SONAE	F.S8	Consigo criamos um impacto positivo na vida de todos. Fazemos com que pequenos gestos resultem em grandes iniciativas. Levamos esperança a quem mais precisa. Descobrimos o herói que há em cada um de nós. Contribuímos

		para um planeta mais sustentável. Estimulamos hábitos saudáveis nas novas gerações. A sua voz é a nossa voz. Juntos somos um e chegamos mais longe.
SONAE	F.S9	O nosso caminho é feito de determinação. Ajudando positivamente a transformar o dia a dia das comunidades. Sensibilizando e mobilizando para fazer as melhores escolhas. Prosseguindo os objetivos de Desenvolvimento Sustentável das Nações Unidas. Estando junto dos que mais precisam nos momentos difíceis. Respeitando a #alimentação, as #pessoas e o #planeta. A Missão Continente? Somos todos nós, todos os dias
SONAE	F.S10	Queremos construir um futuro sustentável a partir de um passado brilhante: Continuar a apoiar as comunidades locais com uma resposta rápida e proativa a situações urgentes. Reforçar o nosso papel transformador junto dos mais novos. Sensibilizar e mobilizar para escolhas mais conscientes, rumo a um mundo melhor.
SONAE	F.S11	Continuamos focados em estar sempre ao lado de quem mais precisa, atuando de forma rápida no combate a situações de emergência social
SONAE	F.S12	A Missão Continente tem como compromisso a Sustentabilidade, contribuindo positivamente para as comunidades em que se insere e atuando em três eixos: Alimentação, Pessoas e Planeta.
SONAE	F.S13	A prossecução dos investimentos em matéria de desempenho ambiental das últimas décadas tem realmente permitido implementar melhorias significativas nas operações diárias da marca:
SONAE	F.S14	A Missão Continente pretende contribuir para a redução do desperdício alimentar, sensibilizando para a adoção de hábitos de consumo mais conscientes e sustentáveis.
SONAE	F.S15	O Continente tem apostado fortemente em iniciativas de economia circular, assumindo-nos como promotores de mudança de comportamentos.
SONAE	F.S16	Esta conferência é aberta a todos e totalmente gratuita, pois consideramos que o acesso e partilha de informação, a interação dos diferentes setores, assim como a literacia alimentar podem resolver problemas como a fome, a má nutrição e o desperdício alimentar.
SONAE	F.S17	Os recursos naturais esgotam-se, mas os comportamentos responsáveis de cada pessoa devem permanecer. Enquanto agente de mudança, temos de dar continuidade a um desempenho ambiental de excelência, envolvendo todas as pessoas possíveis numa causa comum de sustentabilidade ambiental.
SONAE	F.S18	O desenvolvimento socioeconómico e o aumento da qualidade de vida do ser humano provocam um crescimento do consumo que conduz a uma maior utilização dos recursos e à produção de elevadas quantidades de resíduos.
SONAE	F.S19	A nossa missão é criar condições para a melhoria da qualidade de vida e satisfação pessoal e profissional dos nossos colaboradores. Acreditamos que promover a sua satisfação, sentido de união e bemestar é o fator chave do nosso sucesso. É com esta premissa que são pensados e desenvolvidos projetos, que pretendem ir ao encontro das necessidades da nossa comunidade interna.
SONAE	F.S20	Saiba como a literacia alimentar é importante para a promoção da alimentação saudável e aumento da qualidade de vida!
SONAE	F.S21	(…) objetivo de capacitar e aumentar a literacia alimentar
SONAE	F.S22	O movimento À Roda da Alimentação é uma iniciativa do Continente e dos seus especialistas, que nasceu da vontade de inspirar uma mudança de hábitos e de estilos de vida, para ajudar a melhorar a vida de todos os portugueses.
SONAE	F.S23	(…) sensibilizar a população para a importância de um estilo de vida saudável, sobretudo através da alimentação.

SUMOL+COMPAL

Website	Code	CPA EXAMPLES
SUMOL+COMPAL	F.SC1	PREFERIMOS E INVESTIMOS NA FRUTA NACIONAL Apoiamos a economia nacional e potenciamos a sustentabilidade do país preferindo frutas e vegetais locais. Ao reduzirmos o tempo de transporte e distribuição entre a origem e o consumo, reduzimos também o nível de emissões poluentes ao mesmo tempo que preservamos a qualidade nutricional dos alime
SUMOL+COMPAL	F.SC2	Proteger o amanhã significa proteger o planeta hoje. Os nossos produtores parceiros trabalham segundo as melhores práticas agrícolas. Privilegiamos a fruta proveniente de produção integrada ou biológica.
SUMOL+COMPAL	F.SC3	Contribuímos para a sustentabilidade económica dos nossos parceiros com o compromisso de redução do desperdício da fruta
SUMOL+COMPAL	F.SC4	A ingestão adequada de fruta e hortícolas, tal como preconizado em ferramentas de educação alimentar como a Roda da Alimentação Mediterrânica, reduz o risco de algumas doenças como, por exemplo, as cardiovasculares, o cancro do estômago e colorretal, a obesidade e a diabetes. Na verdade, o consumo de fruta, enquadrado num estilo de vida saudável, garante o aporte de nutrientes reguladores, tais como vitaminas, minerais, fibras alimentares e, ainda, compostos fenólicos que, apesar de não serem nutrientes, são substâncias essenciais para a saúde. Com este e-book, pretende-se transmitir os benefícios do consumo diário de fruta e explorar diferentes formas de a incluir na alimentação diá
SUMOL+COMPAL	F.SC5	HÁ 70 ANOS QUE TRABALHAMOS A PENSAR NO AMANHÃ ...e ainda agora começámos AS NOSSAS FRUTAS E VEGETAIS O amanhã importa A NOSSA PRODUÇÃO OS NOSSOS PRODUTOS AS NOSSAS EMBALAGENS A NOSSA RETRIBUIÇÃO
SUMOL+COMPAL	F.SC6	CRIAMOS PARCERIAS PARA A SUSTENTABILIDADE DOS FRUTICULTORES Contribuímos para a sustentabilidade económica dos nossos parceiros com o compromisso de redução do desperdício da fruta Estabelecemos relações fortes e duradouras com os produtores, de Pêssego da Cova da Beira, Pera Rocha do Oeste, Tomate do Ribatejo, algumas das quais duram há mais de 30 anos. Assim, conseguimos oferecer-lhes a segurança do escoamento das suas produções.
SUMOL+COMPAL	F.SC7	ESTABELECEMOS UM CÓDIGO DE CONDUTA NA RELAÇÃO COM OS PRODUTORES Comprometemo-nos a estabelecer um comércio justo com todos os nossos parceiros comerciais. E, tal como nós, os nossos fornecedores comprometem-se a respeitar o ambiente, os seus trabalhadores e a garantir a qualidade e segurança dos produtos.
SUMOL+COMPAL	F.SC8	UTILIZAMOS EMBALAGENS AMIGAS DO AMBIENTE Trabalhamos em conjunto com os nossos parceiros para que as nossas embalagens sejam cada vez mais sustentáveis, permitindo continuar a melhorar a nossa pegada ecológica.
SUMOL+COMPAL	F.SC9	FSC - FOREST STEWARDSHIP COUNCIL Todas as embalagens da Tetra Pak proveem de madeira certificada pelo Forest Stewardship Council ou de outras fontes controladas. As florestas certificadas pelo FSC cumprem todos os princípios ambientais pelos quais nos regemos, assim como o processo de transformação e produção do cartão das embalagens.

SUMOL+COMPAL	F.SC10	COOPERAMOS COM O SETOR AGRÍCOLA NACIONAL Contribuímos ativamente para o aumento da competitividade da fruta nacional. O Centro de Frutologia Compal fomenta a criação de uma rede de contactos numa estratégia integradora dos agricultores com parceiros chave do setor. Assim, potenciamos novas colaborações e parcerias, ajudando a desenvolver o setor.
SUMOL+COMPAL	F.SC11	APOIAMOS AS COMUNIDADES LOCAIS Em 2021, apoiámos cerca de 60 organizações não governamentais com mais de 280 000 litros de produtos, contribuindo para o auxílio às famílias das nossas comunidades.
SUMOL+COMPAL	F.SC12	CENTRO DE FRUTOLOGIA COMPAL é um projeto único em Portugal, que visa estimular a inovação no sector frutícola ao longo da cadeia de valor com o objetivo de valorizar a fruta nacional nas vertentes da produção, da transformação e do consumo, através de iniciativas de investigação, formação e/ou sensibilização. Com espírito colaborativo e multidisciplinar, o Centro reúne entidades e organizações do sector, públicas e privadas, governamentais, empresariais e associativas, que ajudam a robustecer todos os conteúdos gerados e partilhados entre os formadores e os fruticultores
SUMOL+COMPAL	F.SC13	É URGENTE PROMOVER O CONSUMO DE FRUTA PELOS PORTUGUESES ...e com esse propósito o Observatório do Centro de Frutologia Compal propõe-se produzir e disseminar conhecimento sobre a fruta e os seus produtos derivados para sensibilizar e educar para a importância do seu consumo.
SUMOL+COMPAL	F.SC14	POMAR PEDAGÓGICO O Pomar Pedagógico do Centro de Frutologia Compal é o 1.º pomar pedagógico interativo que, em meio urbano, ensina sobre a produção de fruta em Portugal e sobre os benefícios e a importância da fruta para a saúde e para a nutrição. Complementando os formatos de pedagogia em sala, o Centro de Frutologia Compal convida as famílias e as crianças a saírem para o ar livre e para o terreno para adquirirem no Pomar conhecimento sobre fruta.
SUMOL+COMPAL	F.SC15	MAPA DAS FRUTAS DE PORTUGAL O Centro de Frutologia Compal em conjunto com o Centro de Informação Geoespacial do Exército (CIGeoE) desenvolveu o primeiro Mapa das Frutas de Portugal para que todas as crianças conheçam a origem da fruta portuguesa. O Mapa das Frutas de Portugal representa as áreas das frutas de Denominação de Origem Protegida e Indicação Geográfica Protegida que existem em Portugal. À semelhança de exemplos como o Mapa dos Rios ou o Mapa das Serras de Portugal, o Centro de Frutologia Compal alegrase por apresentar o primeiro Mapa das Frutas de Portugal feito com todo o rigor científico e em simultâneo orientado para a utilização pedagógica em sala de aula (ou fora dela). Apoio institucional da Direção Geral de Agricultura e Desenvolvimento Rural.
SUMOL+COMPAL	F.SC16	E-BOOK "COLHER SABER" A alimentação sustentável e a importância da fruta estão na ordem do dia e pequenas escolhas do nosso dia-a-dia poderão fazer toda a diferença. Para alertar e sensibilizar para esta temática, a Associação Portuguesa de Nutrição (APN) elaborou com o apoio do Centro de Frutologia Compal, o E-Book "Colher Saber" com recomendações úteis para promover uma alimentação saudável e sustentável, através do consumo de fruta
SUMOL+COMPAL	F.SC17	COMPAL DOA 1000 PEQUENOS-ALMOÇOS A QUEM MAIS PRECISA Jogo da Compal permitiu aos utilizadores entregar pequenos-almoços a crianças desfavorecidas.
SUMOL+COMPAL	F.SC18	Atualmente o mundo apresenta vários desequilíbrios – emergência climática, biodiversidade em colapso, desigualdade social, crise económica e geopolítica, entre outros –, que se traduzem em grandes desafios para a humanidade, empresas e cidadãos. Temos, por isso, que garantir que as soluções ganham a corrida aos problemas. Na SUMOL+COMPAL vemos a sustentabilidade como uma fonte de oportunidades para fomentar o crescimento contínuo do nosso negócio, e encaramos cada desa"o como catalisador para a melhoria contínua da nossa atividade. Enquadrada na orientação estratégica da empresa, a nossa Agenda de Sustentabilidade 2030 permite-nos conciliar o caminho de criação de valor económico com um modelo de gestão responsável e equilibrado a nível ambiental, social e de governance. A Agenda de Sustentabilidade 2030 da SUMOL+COMPAL as prioridades estratégicas da empresa e estabelece os seus compromissos para os próximos anos.
SUMOL+COMPAL	F.SC19	A nossa Agenda subscreve 8 dos 17 Objetivos de Desenvolvimento Sustentável das Nações Unidas para 2030.
SUMOL+COMPAL	F.SC20	Chegou o mais Fresco dos sumos embalados. Feito de fruta exclusivamente portuguesa, Compal Fresco sabe ao que sabe a fruta acabada de espremer. E numa embalagem única produzida com material 100% reciclado, o que é natural fica ainda melhor.
SUMOL+COMPAL	F.SC21	COMO SÃO FEITOS OS SUMOS DA COMPAL? A nossa principal matéria-prima é a fruta, os seus concentrados ou polpas. Temos sumos à base de concentrados e outros à base de polpas. Algumas das polpas de sabores populares como o pêssego e a pera são feitas na nossa fábrica, ou seja, recebemos os camiões com fruta directamente do pomar e extraímos a sua polpa, sempre respeitando os mais altos padrões de qualidade. Uma vez obtida a polpa ou o concentrado, fazemos o blend, ou seja, a mistura que irá resultar no sumo final. Finalmente a pasteurização e o embalamento que é feito em rigorosas condições de assepsia. Em suma: Receção da Fruta Seleção Lavagem Transformação em polpa Formulação Pasteurização Enchimento da embalagem Embalamento (embalagem secundária) Paletização Chegada do produto final aos Estabelecimentos
SUMOL+COMPAL	F.SC22	O QUE É UMA DOSE DE FRUTA? Uma dose de fruta é definida entre 110-130g de fruta. No caso de Compal Essencial Dose de Fruta, consideramos a equivalência a 115g de fruta.
SUMOL+COMPAL	F.SC23	100% FRUTA É fruta e nada mais do que fruta, sem tirar nem pôr. Só assim garantimos o sabor inconfundível e 100% original da fruta cuidadosamente selecionada com todo o amor à Natureza. Sem açúcares adicionados.
SUMOL+COMPAL	F.SC24	BOM DIA Para dias desafiantes, manhãs fortalecedoras. A gama Bom Dia conta com uma variedade de produtos saciantes resultado da combinação entre um elevado teor de fruta e ingredientes distintos. Sem qualquer tipo de açúcar ou edulcorante adicionado.

UNILEVER

Website	Code	CPA EXAMPLES
UNILEVER	F.U1	Estamos numa posição estratégica para melhorar a saúde, higiene e nutrição de milhões de pessoas em todo o mundo todos os dias. Através das nossas marcas e produtos orientados para objectivos específicos, e enfrentando as barreiras que retêm as pessoas, podemos ajudar as pessoas a realizar o seu potencial ao mesmo tempo que fazemos crescer o nosso negócio.
UNILEVER	F.U2	Todos merecem o acesso a uma boa alimentação. Future Foods constitui o nosso novo compromisso para ajudar na transição para dietas mais saudáveis, para reduzir o impacto ambiental da cadeia alimentar e aumentar o nosso portfólio de produtos à base de plantas.
UNILEVER	F.U3	Queremos fazer mais coisas positivas para o nosso planeta e para a nossa sociedade – não apenas limitar as coisas más. Queremos agir sobre os problemas sociais e ambientais que o mundo enfrenta e queremos melhorar as vidas das pessoas através dos nossos produtos.

UNILEVER	F.U4	Proteger e rejuvenescer a natureza A natureza é a nossa maior aliada. Estamos numa missão para construir um futuro positivo para a natureza. Estamos a trabalhar para eliminar a desflorestação da nossa cadeia de abastecimento e para proteger e regenerar a terra, as florestas e os oceanos.
UNILEVER	F.U5	Um mundo livre de desperdícios A cultura do descartável de hoje tem de acabar. Estamos a trabalhar para manter o desperdício na economia e fora do ambiente através de objectivos ambiciosos para reinventar a forma como utilizamos as embalagens de plástico e reduzir os resíduos alimentares.
UNILEVER	F.U6	Estabelecemos uma agenda de sustentabilidade ambiciosa para abordar as questões com que os nossos consumidores e partes interessadas se preocupam profundamente – tais como as alterações climáticas, a poluição plástica e a desigualdade.
UNILEVER	F.U7	Queremos fazer mais coisas positivas para o nosso planeta e para a nossa sociedade – não apenas limitar as coisas más. Queremos agir sobre os problemas sociais e ambientais que o mundo enfrenta e queremos melhorar as vidas das pessoas através dos nossos produtos.
UNILEVER	F.U8	Proteger e rejuvenescer a natureza A natureza é a nossa maior aliada. Estamos numa missão para construir um futuro positivo para a natureza. Estamos a trabalhar para eliminar a desflorestação da nossa cadeia de abastecimento e para proteger e regenerar a terra, as florestas e os oceanos.
UNILEVER	F.U9	Um mundo livre de desperdícios A cultura do descartável de hoje tem de acabar. Estamos a trabalhar para manter o desperdício na economia e fora do ambiente através de objectivos ambiciosos para reinventar a forma como utilizamos as embalagens de plástico e reduzir os resíduos alimentares.
UNILEVER	F.U10	Além de conduzir a nossa própria pesquisa científica pioneira, trabalhamos intimamente com as principais autoridades mundiais, incluindo reguladores, cientistas governamentais e peritos académicos. Esta colaboração garante que utilizamos sempre os avanços científicos mais recentes dentro das nossas avaliações de segurança e sustentabilidade ambiental.
UNILEVER	F.U11	Somos orientados pelo nosso propósito: contribuir para que todas as pessoas vivam de forma sustentável

VITACRESS

Website	Number	CPA EXAMPLES
VITACRESS	F.V1	Cuidar do ambiente faz parte da nossa natureza.
VITACRESS	F.V2	A Vitacress assume a sua responsabilidade na relação com as pessoas e com a comunidade envolvente através de protocolos de colaboração com várias instituições a nível nacional. É neste sentido que doamos produtos para cabazes sociais, integramos pessoas portadoras de deficiência em contexto de trabalho, instalamos hortas pedagógicas, apoiamos eventos de desenvolvimento local e projetos de proteção ambiental, apoiamos a formação curricular de jovens em idade escolar e envolvemonos em projetos de investigação científica.
VITACRESS	F.V3	Em Portugal, escrevemos uma história de frescura. Para nós, a qualidade máxima é o mínimo que queremos proporcionar aos nossos clientes e isso reflete-se na confiança que a marca Vitacress tem conquistado desde sempre…
VITACRESS	F.V4	Na Vitacress a sustentabilidade assenta em quatro políticas que permitem garantir um impacto positivo e o não comprometimento dos recursos que serão necessários às gerações futuras.

Action examples of the Portuguese Food Industry

APED

Website	Code	CPA EXAMPLES
APED	A.A1	O processo de reformulação dos produtos alimentares em Portugal desenvolvido entre 2018 e 2021 levou a uma redução global de 11,5% e de 11,1% no teor médio de sal e de açúcar (g/100 g), respetivamente, nos produtos abrangidos por este compromisso. Este é o resultado preliminar do trabalho desenvolvido no âmbito do protocolo de reformulação nutricional assinado pela Federação das Indústrias Portuguesas Agro-Alimentares (FIPA), APED e um conjunto de associações setoriais com o Ministério da Saúde e a Direção-Geral da Saúde.
APED	A.A2	APED participa em debate sobre Dieta Mediterrânica na Lisbon Food Affair
APED	A.A3	No âmbito do Dia Internacional da Consciencialização sobre Perdas e Desperdício Alimentar, que se assinala no dia 29 de setembro, o Movimento Unidos Contra o Desperdício Alimentar promove uma sessão dedicada ao tema. A APED integra o Movimento Unidos Contra o Desperdício enquanto membro fundador, dando continuidade ao trabalho já desenvolvido pelo setor ao nível da eficiência dos processos internos que potenciam a redução do desperdício alimentar e da promoção do consumo responsável. O setor do Retalho e Distribuição é parte ativa no combate ao desperdício alimentar, quer ao nível da operação diária e logística, quer ao nível de parcerias com instituições de apoio social para doação dos excedentes, como também no âmbito da sensibilização do consumidor. Os Associados da APED desenvolvem medidas práticas para promover a eficiência dos seus processos internos e a redução do desperdício alimentar, tendo em vista os objetivos e orientações traçados no Pacto Ecológico Europeu e Plano de Ação para uma Economia Circular, bem como os Objetivos de Desenvolvimento Sustentável para 2030. Em 2022, os Associados da APED do Retalho Alimentar foram responsáveis pela doação de mais de 26 mil toneladas de produtos alimentares a instituições sociais, representado um aumento de 12% por comparação com o ano anterior. Estima-se também que, em 2022, evitou-se o desperdício de mais de 27 mil toneladas de produtos que estavam prestes a atingir a data de validade, através de um circuito para facilitar o seu consumo em condições de segurança, o que corresponde a um aumento de 11% face ao valor estimado para o ano anterior. Estão abrangidas 2.631 lojas com esta medida.
APED	A.A4	APED é uma das entidades parceiras de Pós-graduação em Comunicação e Inovação em Gestão da Qualidade e Segurança Alimentar
APED	A.A5	APED reforça compromisso de combater o desperdício alimentar.
APED	A.A6	APED reúne empresas, ONG e académicos em debate sobre sustentabilide.
PARLAMENTO	A.A7	Comissão de Agricultura e Pescas: Jantar Encontro APED EuroCommerce Entidades Promotoras APED - Associação Portuguesa de Empresas de Distribuição Deslocação realizada a convite A Lisboa de 2022-09-29 a 2022-10-29

INSA	A.INSA1	Produtos alimentares com menos 11% do teor de sal e açúcar em três anos: O processo de reformulação dos produtos alimentares é um compromisso entre o Estado, aqui representado pelo Ministério da Saúde e a DGS, e as principais associações do setor alimentar, Federação das Indústrias Portuguesas Agro-Alimentares (FIPA), Associação Portuguesa de Empresas de Distribuição (APED) e outras associações sectoriais.
INSA	A.INSA2	No próximo dia 15 de fevereiro 2022, pelas 15 horas, a Direção-Geral da Saúde (DGS) organiza o webinar "Redução do teor de sal e açúcar nos alimentos", onde serão apresentados os resultados do Progresso da Reformulação dos Alimentos em Portugal durante o período de 2018 a 2021, uma iniciativa da DGS, Instituto Nacional de Saúde Doutor Ricardo Jorge (INSA), Federação das Indústrias Portuguesas Agro-alimentares (FIPA), Associação Portuguesas de Empresas de Distribuição (APED) e NielsenIQ. A melhoria da composição nutricional dos alimentos disponíveis insere-se no âmbito da estratégia alimentar e nutricional nacional desenvolvida pelo Programa Nacional para a Promoção da Alimentação Saudável (PNPAS) e pela Estratégia Integrada para a Promoção da Alimentação Saudável (EIPAS), sendo a reformulação dos produtos alimentares em Portugal um amplo compromisso formalizado em 2019 entre as entidades envolvidas.
PNPAS	A.PNPAS1	Os parceiros do PNPAS | Instituições do setor agroalimentar: FIPA, APED
PS	A.PS1	Após a aprovação da medida, o primeiro-ministro assinou ontem, em Lisboa, com a CAP (Confederação dos Agricultores de Portugal) e com a APED (Associação Portuguesa das Empresas de Distribuição), um pacto para que a proposta governamental de taxa zero para cerca de 44 produtos alimentares essenciais vá em frente, numa altura em que os preços de muitos bens alimentares essenciais têm conhecido subidas vertiginosas nas últimas semanas.

BIMBO+DONUTS

Website	Code	CPA EXAMPLES
BIMBO+ DONUTS	A.B1	Todos os nossos produtos apresentam um esquema de rotulagem na frente da embalagem, para facilitar a compreensão da sua informação nutricional. Este esquema baseia-se no GDA (em português, Valores Diários de Referência), e indica a percentagem de energia e de nutrientes (lípidos, ácidos gordos saturados, açúcares e sal) que fornece uma porção de consumo, de acordo com uma ingestão de referência para um adulto médio de 2000 kcal.
BIMBO+ DONUTS	A.B2	Compromiso ativo A Bimbo Portugal promove um estilo de vida saudável. Lançamos uma plataforma online com guias para uma alimentação saudável e prática de atividades físicas e bem-estar. Inclui várias ferramentas e desafios que ajudam a consolidar novos hábitos benéficos para a saúde.
BIMBO+ DONUTS	A.B3	A ferramenta 'Avalie a sua dieta' analisa a alimentação habitual de cada pessoa, fornecendo dicas para a melhorar. O Prato Saudável mostra como equilibrar almoços e jantares de uma forma simples e visual. Tudo isso em conjunto com menus que facilitam o planeamento de refeições com ideias de receitas equilibradas e alimentos da época.
BIMBO+ DONUTS	A.B4	Desafio 21 dias Junte-se aos desafios! Quer adquirir um bom hábito? Dizem que, para se conseguir um hábito, é necessário realizar e repetir as ações por 21 dias. Prepare-se para seguir uma dieta mais saudável, aprender a cozinhar melhor, juntar bem-estar ao seu dia-a-dia e treinar para estar em forma.
BIMBO+ DONUTS	A.B5	Desafio alimentação saudável #BimboNutriChallenge Nestes 21 dias vai aprender a equilibrar melhor os seus almoços e jantares, com o prato saudável. Nós vamos Guiá-lo nas principais refeições. O pequeno-almoço e lanche fica à sua escolha. A primeira semana será dedicada aos vegetais e às frutas, a segunda aos alimentos proteicos e a terceira aos alimentos que fornecem hidratos de carbono. Lembre-se que todas as partes que compõem o prato saudável deve estar completas.
BIMBO+ DONUTS	A.B6	O nosso compromisso A Bimbo Portugal, preocupa-se consigo, com a sua alimentação e a da sua família; é por isso que preparamos alimentos fofos, saborosos, frescos e nutritivos todos os dias. ! Criamos o Compromisso Bimbo para levar os melhores produtos à sua mesa e para garantir que melhoramos continuamente a sua composição nutricional, comunicamos de maneira responsável e transparente, promovemos um estilo de vida saudável e somos responsáveis pelo planeta. Este é o nosso esforço para preparar bons hábitos e permitir que desfrutemos dos pequenos momentos de cada dia. Da nossa família para a sua. Bimbo ®
BIMBO+ DONUTS	A.B7	Ferramentas alimentares Sabe se cumpre as recomendações de uma dieta saudável? Precisa de um menu que facilite o planeamento da sua dieta? Conhece as vantagens oferecidas pelo Prato Saudável para uma dieta equilibrada? Descubra com as nossas ferramentas alimentares.
BIMBO+ DONUTS	A.B8	Compromisso com o Planeta Cuidar do nosso planeta é responsabilidade de todos, é por isso que a Bimbo Portugal está empenhada em cuidar do nosso ambiente. Para isso, trabalhamos em diferentes áreas para reduzir o nosso impacto ambiental. Para assegurar que os nossos produtos chegam à sua mesa em ótimas condições, as embalagens cumprem uma função fundamental de proteção, que garante a conservação adequada dos alimentos. Assim garantimos a segurança, higiene e preservação da vida útil dos nossos produtos, sendo o plástico um dos materiais com maior adaptação e resposta a estas necessidades. No entanto, sabemos que o fabrico e a gestão dos resíduos plásticos provenientes das embalagens é um desafio para o equilíbrio do planeta. Por isso, confiamos no ecodesenho para a nossa aposta em embalagens mais sustentáveis.
BIMBO+ DONUTS	A.B9	Compromisso de Transparência Temos orgulho do modo como fazemos as coisas, é por isso que o contamos de forma transparente, comprometidos com os consumidores, com a sua saúde e o seu bem-estar. Acreditamos que todos devemos ter em mãos as informações necessárias para fazer escolhas alimentares de acordo com as necessidades nutricionais, circunstâncias e preferências. A Bimbo Portugal ouve os consumidores e a sociedade. Mantê-los informados e satisfazer as suas necessidades são alguns dos pontos mais importantes para nós.
BIMBO+ DONUTS	A.B10	A Global Energy Race é um dos maiores eventos desportivos e familiares realizados a nível internacional. Com presença em 22 países, conta com mais de 1 milhão de participantes em todo o mundo. Em Portugal, é realizada na cidade de Lisboa, com 2 modalidades: corrida de 10 km e caminhada de 5 km, para incentivar a participação de toda a família. (…) Este evento tem como objetivo promover os valores do Grupo Bimbo. Em primeiro lugar, a promoção de um estilo de vida saudável junto dos participantes, que permita desfrutar de um dia de prazer e diversão com familiares e amigos. Em segundo lugar, o espírito solidário: por cada quilómetro percorrido, são doadas duas fatias de pão a bancos de alimentos e outras instituições carenciadas. A nível global e pelo terceiro ano consecutivo, em 2018 foram entregues 1.4 milhões de fatias de pão a Bancos Alimentares, como resultado dos quilómetros percorridos por todos os participantes. Na sua edição anterior, a Global Energy Race reuniu mais de 110 mil participantes a nível mundial e mais de 7.000 só em Portugal e Espanha
BIMBO+ DONUTS	A.B11	Todos os anos organizamos a Global Energy Race, uma corrida que é realizada em 36 cidades de 22 países e que incentiva a participação das famílias, com distâncias de 5 km e 10 km; em todo o mundo, participam mais de 100 000

		atletas de todas as idades. Na Península Ibérica, esta corrida é realizada em Madrid, Barcelona e Lisboa, reunindo mais de 10 000 atletas.
ESB	A.ESB1	A Escola Superior de Biotecnologia desenvolve intercâmbios com múltiplas instituições dentro e fora de Portugal, incluindo centros de investigação, universidades, empresas e unidades de saúde: Modelo Continente Hipermercados, Bimbo, Danone Portugal, Imperial Produtos Alimentares, Nestlé Portugal, Primor Charcutaria Prima, Sonae, Cerealis, Vitacress Portugal

BOM PETISCO

Website	Code	CPA EXAMPLES
BOM PETISCO	A.BP1	Preservar o oceano. Porque a sustentabilidade das pescas é uma preocupação de Bom Petisco, a pesca nos Açores rege-se por critérios rigorosos de proteção do ecossistema marinho. A pesca é realizada em colaboração com o POPA – Programa de observação para as pescas dos Açores -, um projeto de monitorização e controlo das pescas. O objetivo é garantir que nenhum golfinho é molestado na nossa atividade pesqueira. A proteção dos golfinhos e sustentabilidade do mar é também assegurada pelo estatuto de "Friend of the Sea", que visa a redução do impacto humano no equilíbrio do ecossistema marinho.
FMUL	A.FMUL1	O TASTY já conta com a participação entusiasta de 12 doentes que, de forma aleatória, foram alocados ao grupo de controlo ou ao grupo de intervenção. Os doentes que constituem o grupo de intervenção são convidados a seguir um padrão alimentar tipicamente mediterrânico enriquecido em alimentos fermentados, durante 12 semanas, sob acompanhamento por nutricionistas. A intervenção nutricional que implementamos no TASTY vai muito além da entrega de um plano alimentar. Passa também por promover a literacia nesta área e capacitar os doentes para melhores escolhas alimentares e melhores técnicas culinárias. Contamos com a ajuda de vários parceiros para pôr em prática este plano ambicioso, nomeadamente o Pingo Doce, que entrega aos doentes semanalmente cabazes de alimentos selecionados por nós, com a Sovena, que oferece azeite virgem extra Oliveira da Serra (não pode faltar na Dieta Mediterrânica!), com a Nestlé, que oferece os leites fermentados Kefir, e com diversas empresas da indústria das conservas (Bom Petisco, Minerva, Esip e a Cofisa) que oferecem conservas de peixe gordo (sardinha e cavala em azeite virgem extra ou em tomate).

CEREALIS

Website	Code	CPA EXAMPLES
CEREALIS	A.C1	A Milaneza procedeu à entrega de um donativo de 15.000€ à Fundação Portuguesa de Cardiologia, resultado da campanha de angariação de 30 cêntimos por cada massa Natura Corações Pró-vita com betaglucanovendida.
CEREALIS	A.C2	Um conjunto de parcerias de investigação e comunicação dedicadas a estilos de vida saudável e prevenção de doenças.
CEREALIS	A.C3	Nacional apoia vice-campeões do Mundo que representam Portugal nos Jogos Olímpicos
CEREALIS	A.C4	Milaneza celebra o Dia Mundial das Massas terça-feira, 26 outubro 2021 A base da alimentação… está (também) no pão! quarta-feira, 20 outubro 2021 Protocolo entre a Milaneza, marca do grupo Cerealis, e a NOVA Medical School vai permitir estudar o impacto do consumo de massa no índice glicémico.
CEREALIS	A.C5	Gabinete de Nutrição GABINETE DE NUTRIÇÃO CEREALIS O Gabinete de Nutrição Cerealis é um serviço que tem como principal objetivo a promoção da educação alimentar. Queremos ajudá-lo nas suas tomadas de decisão, na procura de uma alimentação mais saudável e equilibrada. Os principais eixos de intervenção do nosso Gabinete são: 1. Garantir o acesso a uma informação adequada sobre hábitos alimentares saudáveis; 2. Contribuir no processo de ajustamento no sentido de uma dieta alimentar mais equilibrada e saudável; 3. Esclarecer dúvidas e falsos mitos sobre alimentação. Escreva-nos e esclareça as suas dúvidas sobre alimentação, através do contacto: Gabinete.NutricaoCerealis@cerealis.pt Tentaremos responder a todas as questões, durante os dias úteis, das 9h às 13h e das 14h às 18h.
CEREALIS	A.C6	Programas gerais específicos de Nutrição para o público externo e interno.
CEREALIS	A.C7	Criamos produtos saudáveis: Contribuímos para uma dieta completa, variada e rica em nutrientes.
CEREALIS	A.C8	A FAMÍLIA ZERO CRESCEU Com sabor, prazer e equilíbrio … mas sem açúcares adicionados! Comece o dia com o sabor inconfundível dos Corn Flakes, com a leveza dos cereais +Linha da Nacional ou faça uma pausa com as Mini Cookies.
CEREALIS	A.C9	PAPAS DE AVEIA NACIONAL COM MENOS 30% DE AÇÚCAR Com canela ou com framboesas, estas Papas de Aveia são saborosas, práticas e muito fáceis de preparar! As Papas de Aveia são uma verdadeira fonte de energia, uma solução inovadora e nutricionalmente equilibrada, que pode ser consumida por toda a família e em diferentes momentos do dia.
CEREALIS	A.C10	(…) a procura de alternativas mais saudáveis e convenientes, tem vindo a ganhar uma maior preponderância, destacando-se alguns movimentos com impactos significativos na indústria alimentar: a revolução veggie; a redução do açúcar; os cereiais integrais; a nutrição na cerealis.
CEREALIS	A.C11	A redução de açúcar Enquadrado neste compromisso destacamos os principais esforços e resultados:<br>1. Redução de açúcar na categoria de cereais de pequeno-almoço.<br>Nos últimos 5 anos, a Cerealis reduziu em 22% o consumo de açúcar. Foram – 510 toneladas de açúcar consumidas (mais de 100 milhões de pacotes de açúcar retirados nos nossos cereais de pequeno-almoço).<br>2. Redução de sal na categoria de cereais de pequeno-almoço.<br>Nos últimos 5 anos retiramos 27 toneladas de sal dos nossos cereais de pequeno-almoço. Uma redução de 38%.<br>3. Disseminação de informação séria e credível<br>Investimos no serviço de informação e aconselhamento através do Centro de Nutrição de cada uma das plataformas das nossas marcas, na divulgação de dicas nas suas páginas online, na promoção de uma plataforma dedicada à nutrição para desportistas na página online da Milaneza, na utilização do Software Movelife aplicado no extenso conjunto de receitas das marcas, facilitando assim a classificação nutricional da receita depois de concluída, assim como da divulgação de documentação técnica de suporte à promoção de boas práticas alimentares.<br>4. Promoção de uma cultura nutricional interna<br>Procuramos assegurar que a nutrição faz parte da nossa cultura interna, acreditando que conhecimento nutricional contribui para o desenvolvimento e melhor qualidade de vida dos nossos colaboradores. É neste sentido que a

		nutrição faz parte do plano de formação geral interno. Simultaneamente, na Cerealis disponibilizamos um programa de apoio nutricional gratuito a todos os colaboradores.
CEREALIS	A.C12	CORAÇÕES PRÓ-VITA MILANEZA Pró-vita com beta-glucano vendida. As massas Corações Pró-vita com beta-glucano da gama Milaneza Natura, são indicadas para quem procura o equilíbrio através de uma alimentação cuidada, com especial enfase para quem necessita de ajuda para controlar o colesterol. Reduz o colesterol e conquista um lugar no seu coração. Cada embalagem contribui com €0,30 para a Fundação Portuguesa de Cardiologia.
CEREALIS	A.C13	Na categoria de bolachas destacamos os projetos de redução e ou substituição de gorduras com menor teor de saturados, prosseguindo um conjunto de iniciativas no sentido da melhoria dos seus índices nutricionais, nomeadamente: - Substituição de gordura de palma por óleo de girassol alto oleico em várias referências, reduzindo a incorporação de gordura saturada na ordem dos 40%; - Redução, até ao limite de 10%, do teor de gordura das receitas; - Maior utilização do azeite virgem extra, gordura rica em ácidos gordos monoinsaturados e vitamina E (com função antioxidante sobre as paredes das artérias).
CEREALIS	A.C14	CEREALIS MANTÉM-SE 100% PORTUGUESA As famílias Amorim e Lage chegaram a acordo com as famílias Moreira da Silva e Silva Domingues para a venda de 100% do Grupo Cerealis.
CEREALIS	A.C15	A Cerealis e as suas marcas Milaneza e Nacional aderem ao movimento "Unidos Contra o Desperdício", um movimento cívico que une a sociedade num combate ativo e positivo ao desperdício alimentar.
CEREALIS	A.C16	Com três instalações com Licenciamento Ambiental, na Cerealis estendemos as práticas de redução de desperdícios, reciclagem de resíduos, racionalização de consumos energéticos e políticas de redução de emissões de poeiras a todos os centros produtivos.
CEREALIS	A.C17	REPRESENTAÇÃO DO GRUPO EM ASSOCIAÇÕES E INSTITUIÇÕES Através das diferentes empresas do Grupo, garantimos um conjunto de parcerias com diversas associações e instituições nas quais mantemos uma representação Associações e Instituições com representação das empresas do Grupo Cerealis.
CEREALIS	A.C18	A Cerealis e as suas marcas Milaneza e Nacional aderem ao movimento "Unidos Contra o Desperdício", um movimento cívico que une a sociedade num combate ativo e positivo ao desperdício alimentar.
APN	A.C19	Patrocínio Congresso Alimentação e Nutrição 2022 através da marca Milaneza.
APN	A.C20	Patrocínio Congresso Alimentação e Nutrição 2023 através da marca Milaneza.
APN	A.C21	Patrocínio Congresso Alimentação e Nutrição 2023 através da marca Nacional.
NMS	A.C22	Prémio CEREALIS em Química dos Alimentos 2022: Ao abrigo da parceria entre a NMS e CEREALIS
NMS	A.C23	Prémio CEREALIS em Química dos Alimentos 2023: Ao abrigo da parceria entre a NMS e CEREALIS
FCNAUP	A.FCNAUP1	Identificam-se algumas das instituições, nacionais e internacionais, que acolheram estágios curriculares de finalistas da FCNAUP: Cerealis
ESB	A.ESB1	A Escola Superior de Biotecnologia desenvolve intercâmbios com múltiplas instituições dentro e fora de Portugal, incluindo centros de investigação, universidades, empresas e unidades de saúde: Modelo Continente Hipermercados, Bimbo, Danone Portugal, Imperial Produtos Alimentares, Nestlé Portugal, Primor Charcutaria Prima, Sonae, Cerealis, Vitacress Portugal
ESB	A.ESB2	A Escola Superior de Biotecnologia tem apostado na criação e desenvolvimento de parcerias com a indústria e outras organizações a nível nacional e internacional, sendo esta uma via privilegiada para fortalecer a transferência de conhecimento e tecnologia e de manter o diálogo com o mundo profissional. O resultado dessa aposta é visível e transparece em múltiplas formas de colaboração entre a ESB e diversas empresas, sempre mediadas pelos nossos estudantes dos vários níveis académicos (licenciatura, mestrado, doutoramento). Abaixo descrevem-se alguns exemplos que justificam o bom reconhecimento dos diplomados da ESB no mercado de trabalho: Cerealis, Continente, Delta, FIPA, SONAE MC,
ESB	A.ESB3	Lançado em outubro de 2021, num evento público, o INSURE.hub está já a cumprir o seu grande objetivo: criar um ecossistema vibrante, nacional e internacional, de conhecimento transdisciplinar de âmbito circular, sustentável e regenerativo. São já mais de 30 as entidades que se associaram a esta iniciativa que resulta da mobilização da Universidade Católica Portuguesa, no Porto, através das suas Faculdades - Católica Porto Business School e Escola Superior de Biotecnologia - e da Planetiers New Generation, em conjunto com organizações nacionais e internacionais que se constituem como líderes de pensamento e agentes de transformação para a Sustentabilidade e a Regeneração: Cerealis, Imperial, Vitacress
ESB	A.ESB4	A Semana Aberta é uma iniciativa da Escola Superior de Biotecnologia que se realiza desde 1992 e se repete todos os anos na primavera. Em 2024 a 32ª edição decorre nos dias 10, 11 e 12 de abril e está disponível sem custos para as escolas secundárias – todos os alunos dos cursos de ciências e tecnologias são bem vindos. As inscrições devem decorrer preferencialmente até 22 de março de 2024. Patrocinadores: Cerealis, Nestlé, Sovena

COCA-COLA

Website	Code	CPA EXAMPLES
Coca-cola	A.CC1	Mantemos uma estreita relação com a indústria agroalimentar, participando em diferentes fóruns que nos ajudam a acompanhar as tendências, a manter uma relação com outras empresas e instituições do setor, e a colaborar com as suas experiências na melhoria de sistema e processos.
Coca-cola	A.CC2	Também pertencemos a várias associações do setor da alimentação e das bebidas, assim como algumas associações empresariais, colaborando com muitas outras.
Coca-cola	A.CC3	Temos uma política de marketing responsável de não publicitar nenhum dos nossos produtos a menores de 12 anos. Através da UNESDA, a associação europeia da indústria de refrigerantes, comprometemo-nos a não fazer publicidade na imprensa escrita, websites ou durante a emissão de programas especificamente dirigidos a crianças. Além disso, estamos empenhados na eliminação de todas as bebidas com adição de açúcar nas escolas secundárias em toda a Europa.
Coca-cola	A.CC4	Coca-Cola Zero Zero Os nossos dias estão cada vez mais acelerados e stressantes, devido às logísticas que o dia a dia exige. A Coca-Cola Zero Zero, sem açúcar e cafeína, vem com o propósito de oferecer um momento para relaxares e para que possas saborear o momento a qualquer hora do dia
Coca-cola	A.CC5	Pretendemos ter pelo menos 50% das nossas vendas a pa!ir de bebidas baixas em calorias ou sem calorias até 2025. Estamos também a facilitar aos consumidores a redução do consumo de açúcar, fornecendo informações simples sobre cada produto e tornando as embalagens mais pequenas e adequadas para cada utilização.
Coca-cola	A.CC6	24,6% de redução média de açúcar nas nossas bebidas, desde 2015
Coca-cola	A.CC7	45,5% das bebidas vendidas em 2022 foram bebidas baixas em calorias ou sem calorias

Coca-cola	A.CC8	Para todas as marcas que comercializamos temos as variantes de baixo teor ou sem qualquer adição de açúcar e desde 2015 já reduzimos em 24,6% a média de açúcar presente nas nossas bebidas.
Coca-cola	A.CC9	Atualmente, os consumidores procuram uma oferta mais vasta de bebidas, com mais opções capazes de se adaptarem a uma maior variedade de momentos e ocasiões de consumo, e com as quais também podem gerir melhor a quantidade de açúcar que consomem. De acordo com as recomendações da Organização Mundial de Saúde (OMS), trabalhamos para ajudar o consumidor a não exceder a ingestão de, no máximo, 10% de calorias provenientes de açúcares livres por dia (adição de açúcares, xaropes, mel, sumos de fruta, etc). A este respeito, estamos a trabalhar com a The Coca-Cola Company e outros engarrafadores para oferecer aos consumidores uma variedade ainda maior de bebidas, que incluem mais opções de bebidas com baixo teor de açúcar ou sem açúcar. A nossa ação em matéria de bebidas ajuda-nos a apoiar o Objetivo de Desenvolvimento Sustentável 3 das Nações Unidas – Saúde e Bem-Estar, assim como a reforçar o objetivo 17 (Alianças para alcançar os objetivos) trabalhando em conjunto com os nossos pa!ners e stakeholders.
Coca-cola	A.CC10	JUNTA-TE À COCA-COLA PARA AJUDAR OS PALHAÇOS D'OPITAL Este ano a Coca-Cola uniu forças com a associação Palhaços D'Opital, para proporcionar alegria e sorrisos àqueles que vão passar esta data nos hospitais parceiros. Vem ajudar-nos a levar mais de 70 000 sorrisos aos que mais necessitam.
Coca-cola	A.CC11	No Wonderland, poderás deixar uma mensagem de Natal para alguém em um hospital parceiro e/ou ajudar a Coca-Cola a contribuir para esta causa através de um código QR que vai estar disponível no nosso espaço. Esse código QR vai-te dar um "Presente" na nossa App que vais poder trocar por um sorriso (esse sorriso representa uma contribuição monetária da Coca-Cola para esta iniciativa). Com a tua ajuda vamos conseguir distribuir mais de 70 000 sorrisos. Partilha esta inciativa com os teus familiares e amigos - o mundo precisa de mais Pais Natais.
Coca-cola	A.CC12	O mundo precisa de mais Pais Natais e, por isso, este ano a Coca-Cola convida-te a embarcar numa viagem mágica e solidária para despertares o Pai Natal que há em ti, incentivando as boas ações e relembrando os valores do Natal. Traz a tua família e vem conhecer o nosso espaço no parque Eduardo VII, entre o dia 30 de Novembro e 1 de Janeiro.
Coca-cola	A.CC13	Queremos que as nossas embalagens contenham 50% de PET reciclado até 2022, o que evitaria o consumo de mais de 20.000 toneladas de plástico virgem por ano. Estamos assim adiantados em relação ao prazo previsto (inicialmente para 2025) e mantemos o nosso objetivo de alcançar 100% de plástico reciclado ou renovável no futuro.
Coca-cola	A.CC14	Estamos determinados a marcar uma diferença positiva na sociedade ao promover a inclusão, a diversidade e o desenvolvimento económico, tanto nos nossos locais de trabalho como nas comunidades locais. Promovendo uma cultura diversificada e inclusiva, estamos a trabalhar para que as mulheres ocupem, pelo menos, 40% dos nossos cargos diretivos até 2025. Além disso, ajudamos a abordar as questões sociais dentro dos territórios onde operamos através do voluntariado dos nossos colaboradores e ao apoio a associações locais, com especial enfoque em programas que procuram melhorar a empregabilidade das mulheres e dos jovens em situações menos favoráveis, dando-lhes as competências e a confiança que necessitam para serem bem-sucedidos.
Coca-cola	A.CC15	Queremos reduzir a água que utilizamos nos nossos processos de produção em 20% até 2025, assim como abordar os impactos que a sua utilização tem na nossa cadeia de fornecimento. O nosso objetivo é devolver 100% da água nas nossas bebidas, especialmente nas zonas de stress hídrico, trabalhando com parceiros locais.
Coca-cola	A.CC16	Estamos a reduzir as emissões de gases com efeito de estufa em toda a nossa cadeia de valor, com o objetivo de reduzir as nossas emissões diretas de carbono em 50% até 2025 (desde 2010 já as reduzimos 29,9%). Temos também equipamentos de refrigeração e de iluminação, entre outros, mais energicamente eficientes. Também nos comprometemos a utilizar 100% de eletricidade renovável e a reduzir as emissões em toda a nossa cadeia de valor em 35% (desde 2010, a redução já é de 16,1%).
Coca-cola	A.CC17	Em Portugal, em 2019, foram realizadas 652 horas de voluntariado pelos colaboradores. Promovemos a saúde e a segurança dos colaboradores ao proporcionar um ambiente seguro de trabalho, saudável e produtivo, e facilitando-lhes a pa!icipação em vários eventos e programas. Estamos convencidos da impo!ância de educar e formar as pessoas da nossa equipa: através de diferentes iniciativas, os colaboradores CCEP podem crescer profissionalmente connosco, desenvolvendo a sua carreira profissional e pessoal. Em 2019, foi ministrada uma média de 10,5 horas de formação por colaborador.
Coca-cola	A.CC18	O BORA Mulheres, o nosso programa para empoderar e capacitar as mulheres com as ferramentas necessárias para o empreendedorismo já envolveu a nível ibérico quase 14.000 mulheres desde 2016. Com as formações do programa, ajudamo-las a melhorar a sua empregabilidade e a que se possam tornar o motor económico nas suas comunidades.
Coca-cola	A.CC19	Em 2019, lançámos o nosso plano de voluntariado empresarial: I Love Voluntariado. Com ele, reforçamos o nosso compromisso de contribuir positivamente para a sociedade, promovendo a pa!icipação dos nossos colaboradores em projetos ou boas causas, contando com dois dias de trabalho remunerado por ano para colaborar nesta iniciativa. O nosso objetivo é que até 2025 ofereçamos o equivalente a 100.000 horas de voluntariado empresarial por ano.
Coca-cola	A.CC20	Reduzimos a utilização de matérias-primas, aliviando as nossas embalagens e fabricando-as com materiais mais sustentáveis. Por exemplo, as nossas latas de alumínio são 60% mais leves do que há 30 anos. Além disso, estamos a trabalhar para reduzir o uso de plástico no packaging secundário de latas e garrafas, substituindo os anéis agrupadores de plástico nos nossos formatos de latas de 330 ml por agrupadores de ca!ão, poupando assim 35,3t de plástico durante 2019. Também substituímos o filme retrátil nos packs de latas de 200 ml por cartão certficado PEFC, o que nos permitiu eliminar a utilização de 5,15t de plástico em 2019.
Coca-cola	A.CC21	Coca-Cola assinala um ano de parceria com o Desporto Adaptado do Futebol Clube do Porto
Coca-cola	A.CC22	Coca-Cola Portugal lança podcast "sustentável" com Ana Isabel Arroja
Coca-cola	A.CC23	Programa 'Bora Jovens apoia mais de 140 jovens
Coca-cola	A.CC24	Programa Mares Circulares reúne mais de 9 mil pa!icipantes e recolhe 6,5 toneladas de resíduos
Coca-cola	A.CC25	Mares Circulares já formou cerca de 10.000 cidadãos e fez 96 intervenções com 8,7 toneladas de resíduos recolhidos
Coca-cola	A.CC26	Festivais de verão com Coca-Cola
Coca-cola	A.CC27	Utilizaremos eletricidade 100% renovável em todos os mercados até 2030
Coca-cola	A.CC28	100% do carbono dos fornecedores estratégicos devem estabelecer objectivos baseados cientificamente até 2023 (Europa) e 2025 (API)
Coca-cola	A.CC29	Asseguraremos que 100% das nossas embalagens sejam recicláveis até 2025 50% de rPET nas nossas embalagens PET na Europa até 2023. Nos outros mercados até 2025 Parar de utilizar plástico virgem derivado de petróleo nas nossas embalagens até 2030 Recolher e reciclar uma garrafa ou uma lata por cada uma vendida até 2030 Estabelecer objectivos de água em todos os locais de fabrico Reporemos 100% da água que utilizamos nas nossas bebidas 100% de utilização regenerativa da água em "locais líder" até 2030 100% dos principais ingredientes agrícolas e matérias-primas são provenientes de fontes sustentáveis

Coca-cola	A.CC30	100% dos fornecedores devem ser abrangidos pelos nossos Princípios Orientadores para Fornecedores - incluindo sustentabilidade, ética e direitos humanos
Coca-cola	A.CC3 1	Reduzir o açúcar: em 10% na Europa até 2025C Até 2030, mais de 50% das vendas serão provenientes de bebidas sem ou de baixas calorias (Europa até 2025)E 45% dos cargos de gestão deverão ser ocupados por mulheres até 2030 Um terço da nossa força de trabalho será constituída por mulheres até 2030 10% da nossa força de trabalho representada por pessoas portadoras de deficiência até 2030
Coca-cola	A.CC3 2	Apoiar o desenvolvimento de competências de 500 000 pessoas que enfrentam obstáculos no mercado de trabalho até 2030
Coca-cola	A.CC33	Zero emissões de gases com efeito de estufa até 2040
Coca-cola	A.CC34	Redução de emissões de gases com efeito de estufa Reduzir emissões em 30% até 2032
Coca-cola	A.CC35	Em 2022 registamos uma redução na emissões de gases de estufa de cerca de 20,4% desde 2019 e diminuímos o rácio de utlização de energia no fabrico, sendo apenas dispendido 0,34 MJ por cada litro produzido.
Coca-cola	A.CC36	Toda a energia utilizada na fábrica é proveniente de fontes renováveis.
Coca-cola	A.CC37	Reconhecemos o nosso papel na luta contra as alterações climáticas e a necessidade de reduzir o nosso impacto ambiental. Por isso, estabelecemos na Europa Ocidental o objetivo de alcançar a neutralidade carbónica até 2040 e de reduzir as nossas emissões absolutas de Gases com Efeito de Estufa (GEE) em 30% em toda a nossa cadeia de valor até 2030.
Coca-cola	A.CC38	Desde 2018 que 100% da energia elétrica contratada na nossa fábrica de Azeitão é certificada de origem renovável (eletricidade verde). Em 2021 trocamos toda a nossa frota comercial por veiculos hibridos e em 2022 instalamos a primeira fase do nosso parque fotovoltaico com uma área de 10.682 m2.
Coca-cola	A.CC39	Este novo parque fotovoltaico irá melhorar a eficiência e o desempenho da nossa unidade industrial em Portugal com uma poupança de 2750 MWH evitando a emissão de cerca de 500 toneladas de A.CO2 por ano. Prevêmos iniciar a instalação da segunda fase deste parque fotovoltaico durante o ano de 2023 acrescentado mais 11.000 m2, totalizando 21.682m2 solar produzida nas nossas instalações.
Coca-cola	A.CC40	Nos últimos três anos investimos cerca de 3,857M€ em novos equipamentos de frio energeticamente mais eficientes, o que resultou numa redução de 46% neste tipo de equipamentos e uma poupança de cerca de 42.000Mwh (-41%).
Coca-cola	A.CC41	Em 2022 instalamos a primeira fase do parque fotovoltaico da fábrica de Azeitão. Com uma área de 10.682 m2 no topo das instalações fabris permitiu uma poupança de 2750 MWH e evita a emissão de 500 toneladas de A.CO2 por ano. Prevemos iniciar a segunda fase de instalação durante o ano de 2023, com a ocupação de mais 11.000 m2
Coca-cola	A.CC42	Em 2022 continuámos a apostar na nossa estratégia de sustentabilidade que visa alcançar "Um mundo sem resíduos". 99,2% das nossas embalagens são recicláveis e 31,9% do PET total utilizado já é rPET.
Coca-cola	A.CC43	Conseguimos recolher para reciclagem, por via local ou com parceiros nacionais, 35,5% do total de embalagens que colocamos no mercado.
Coca-cola	A.CC44	Uma vez que as nossas embalagens são recicláveis, podemos recuperá-las, reincorporando-as no ciclo produtivo e fechar o círculo rumo à economia circular. Estamos a trabalhar junto com a indústria e o retalho para implementação do sistema de depósito e reembolso que nos permitirá recolher 90% das embalagens colocadas no mercado.
Coca-cola	A.CC45	Criado em 2018, Mares Circulares é um projeto que promove a sensibilização e formação ambiental para uma cidadania ativa, com foco na economia circular e na limpeza de resíduos em ambientes aquáticos e fundos marinhos. Nas suas quatro edições, já foi possível recolher cerca de 8,7 toneladas de resíduos e contar com mais de 7000 voluntários nas iintervenções em praias e ambientes aquáticos.
Coca-cola	A.CC46	Em 2022 demos mais um passo no nosso caminho para a eliminação da utilização de plástico virgem à base de óleo. Atingimos a meta de 100% rPET nas embalagens de 500 ml e de todas as bebidas sem gás, eliminando assim a utilização de plástico virgem nestas embalagens.
Coca-cola	A.CC47	Promovemos a gestão eficiente e a redução do uso de água em processos de produção.
Coca-cola	A.CC48	100% das nossas instalações contam com planos de proteção das fontes de água e avaliações da sua vulnerabilidade.
Coca-cola	A.CC49	A nossa taxa de utilização de água em 2022 foi de 1,55l por cada litro produzido de bebida e utlizamos a nossa ETAR para devolver 100% das nossas águas residuais em segurança à natureza.
Coca-cola	A.CC50	Apostamos na inovação constante e na promoção da sua circularidade, tanto pela redução do seu consumo como pela sua reutilização noutros processos. Além disto, são realizadas avaliações periódicas para conhecer e avaliar a quantidade e a qualidade da água disponível na nossa fábrica, a quantidade de chuva recebida pelos aquíferos e nascentes e os possíveis riscos derivados de fenómenos meteorológicos extremos ou desastres naturais.
Coca-cola	A.CC51	Em 2022 conseguimos melhorar o nosso rácio de utlização de água para 1,55l de água utilizada para cada litro de bebida produzida. Este valor representa uma redução de cerca de 4% na água utilizada para produção de cada litro de bebida em comparação com 2020 e 30% em relação a 2010.
Coca-cola	A.CC52	O nosso Código de Conduta e os Princípios Orientadores para Fornecedores constituem as bases de todo o Sistema Coca-Cola de modo a garantir o cumprimento dos nossos compromissos em questões de sustentabilidade na nossa relação com os fornecedores.
Coca-cola	A.CC53	100% do nosso açúcar provém de fornecedores que cumprem com os nossos Princípios Orientadores de Agricultura Sustentável (POAS)
Coca-cola	A.CC54	Fomos incluídos pelo quinto ano consecutivo na 'CDP A List – Supplier Engagement Leaderboard' pelo forte compromisso dos nossos fornecedores no combate às alterações climáticas.
Coca-cola	A.CC55	As parcerias com fornecedores são fundamentais para alcançarmos a neutralidade carbónica até 2040 e reduzir as nossas emissões de gases com efeito estufa em 30%, até 2030 (vs. 2019)
Coca-cola	A.CC56	Uma grande parte das nossas emissões está relacionada com a nossa cadeia de fornecimento e estamos a trabalhar em estreita colaboração com os nossos fornecedores para os ajudar a desenvolver os seus próprios planos de redução de carbono e conseguirem fazer a transição para eletricidade 100% renovável.
Coca-cola	A.CC57	98,1% das nossas despesas foram efectuadas com fornecedores abrangidos pelos nossos "Príncipios orientadores para fornecedores" (POPF). Continuamos a trabalhar com os nossos fornecedores para construir uma cadeia de abastecimento sustentável, resiliente e de baixo carbono e nos ultimos três anos conseguimos aumentar em cerca de 9% os nossos gastos em fornecedores que cumprem os nossos POPF .
Coca-cola	A.CC58	Em 2022 conseguimos uma redução média de 24,6 do açúcar no nosso portfolio de bebidas em relação a 2015. 45,5% das bebidas vendidas em 2022 foram bebidas baixas em calorias ou sem calorias. Em Portugal desde 2010 já lançamos 44 novos produtos sem ou baixos em calorias.
Coca-cola	A.CC59	Em 2022, 30,6% dos nossos cargos de gestão sénior e superior são ocupados por mulheres.

Coca-cola	A.CC60	'Bora Jovens é um programa de empregabilidade jovem, criado pela Coca-Cola e implementado pela ONG Ajuda em Ação, destinado a jovens em risco de exclusão social, com o objetivo de os capacitar para facilitar a sua integração no mercado de trabalho. Já capacitou mais de 200 participantes e integrou mais de 80 jovens, contando com os nossos clientes como parceiros integradores.
Coca-cola	A.CC61	Apoiámos também dezenas de instituições através de programas ambientais e sociais e ajudámos mais de 60 associações com um apoio equivalente a cerca de 400.000 latas.
Coca-cola	A.CC62	O 'BORA Mulheres é um programa de empreendedorismo feminino, criado pela Coca-Cola e desenvolvido pelo Impact Hub, dirigido a mulheres que queiram lançar a sua ideia de negócio ou desenvolver o seu projeto.
Coca-cola		Entre 2019 e 2022, o projeto apoiou cerca de 1.500 empreendedoras, capacitando e dando as ferramentas necessárias para entrarem no mundo do empreendedorismo com confiança e sucesso.
Coca-cola	A.CC63	"Avançamos" no nosso objetivo de reduzir o uso de plástico nas nossas embalagens. Atualmente, as nossas garrafas de plástico já têm 25% de plástico reciclado (rPet) e o objetivo é aumentar essa percentagem para 50% em apenas dois anos. No último ano, investimos mais de 180 milhões em eco inovação para liderar a transição para uma economia circular, reduzir a nossa pegada de carbono e criar um mundo mais sustentável através do nosso negócio.
Coca-cola	A.CC64	Desde 2019, todas as garrafas PET da marca Coca-Cola têm tampas de plástico com uma mensagem dirigida diretamente ao consumidor para o encorajar a reciclar "Por favor, recicla-me".
Coca-cola	A.CC65	Reduziremos em 20% a água que utilizamos na produção e abordaremos os impactos da água na nossa cadeia de fornecimento. Entre 2010 e 2019, já conseguimos uma redução no consumo de 28,8% (-5% só em 2019). Em 2019, o rácio de uso de água por litro foi de 1,59 litros.
Coca-cola	A.CC66	Reduziremos as emissões de gases com efeito de estufa do nosso negócio principal em 50%. Com uma redução de 42,05% desde 2010, já estamos pe!o!
Coca-cola	A.CC67	Asseguraremos que 100% dos nossos principais ingredientes agrícolas e matérias-primas sejam provenientes de fontes sustentáveis antes do final de 2020.
Coca-cola	A.CC68	"Conversas Sustentáveis" é o novo podcast powered by Coca-Cola, que pretende dar palco aos grandes protagonistas nacionais que estão a contribuir para moldar um futuro mais sustentável.
Coca-cola	A.CC69	A Coca-Cola celebra este ano 45 anos em Portugal. A primeira garrafa foi vendida no Chiado, a 4 de julho de 1977, e bom… o resto é história! Os próximos 45 anos serão, sem dúvida, repletos de novos desafios ao nível da sustentabilidade e da economia. Nestas "Conversas Sustentáveis", com moderação da locutora de rádio Ana Isabel Arroja, vamos conhecer cinco representantes de iniciativas po!uguesas que atuam com o objetivo de alcançar um futuro mais "verde"!

DANONE

Website	Code	CPA EXAMPLES
DANONE	A.D1	A Nutrição Médica Avançada Nutricia é movida por um forte propósito: ser pioneira nas descobertas nutricionais que ajudem os pacientes a viver mais tempo de forma saudável. A Nutricia trabalha em conjunto com a comunidade científica no desenvolvimento de alimentos baseados em evidências para necessidades médicas especiais, que contribuam positivamente e comprovadamente para a saúde de pacientes, de todas as idades.
DANONE	A.D2	Neste contexto, a Danone apresenta o novo Actimel Vitamina C, a nova proposta da marca para cuidar das defesas – um leite fermentado que associa as bactérias tradicionais do iogurte ao seu fermento lático L. casei, específico e exclusivo. Para além disso, Actimel Vitamina C, é rico em vitamina C e contém as vitaminas D e A.B6 e o mineral zinco, que ajudam ao normal funcionamento do sistema imunitário!
DANONE	A.D3	Os esteróis vegetais presentes em Danacol reduzem o seu colesterol de forma 100% natural. Estes ajudam a bloquear parcialmente a absorção de colesterol de modo a impedir que parte chegue ao sangue, ajudando-o a reduzir até 10%. Danacol deverá ser consumido integrado numa alimentação variada e equilibrada e num modo de vida saudável.
DANONE	A.D4	Desde então, a Danone, como membro fundador do compromisso da UE (2007) e signatária da política global da International Food and Beverage Alliance (IFBA) sobre Comunicações de Marketing Responsável para Crianças, tem restringido as comunicações a crianças com menos de 12 anos de idade, de modo a que só recebam informações sobre os nossos produtos que estejam em conformidade com: Disposições legais, diretrizes nacionais ou normas comuns de nutrição industrial amplamente aceites, estabelecidas a nível regional ou local. Critérios nutricionais comuns do Compromisso da UE, na ausência de tais normas. No que diz respeito à comercialização para crianças: Não divulgar mensagens enganosas Não enfraquecer a influência dos pais Nenhuma sugestão de tempo / senso de urgência ou pressão para minimizar os preços Nenhuma exploração da imaginação ou inexperiência de uma criança Não encorajar hábitos alimentares insalubres Não esbater as fronteiras entre marketing e branding
DANONE	A.D5	Na Danone estamos empenhados em promover produtos saudáveis, acima de tudo, através do investimento em marketing. Uma elevada percentagem das nossas vendas provém de categorias de produtos saudáveis e, graças ao nosso empenho na nutrição, esperamos que os recursos financeiros que dedicamos à comunicação destes produtos e alternativas saudáveis aumentem globalmente nos próximos anos. Além disso, devido aos nossos compromissos nutricionais, esperamos que as nossas apostas de marketing em produtos mais saudáveis e alternativas aumentem globalmente nos próximos anos.
DANONE	A.D6	Comunicação e Marketing responsável Como empresa alimentar acreditamos que desempenhamos um papel fundamental na conquista por um impacto positivo na saúde pública e nas questões nutricionais, através dos nossos produtos. Ao comunicar os nossos produtos e serviços, acreditamos que podemos ajudar a educar e sensibilizar todos os consumidores, que confiam em nós diariamente, para a importância de uma dieta e estilo de vida saudáveis. É por isso que na Danone estamos empenhados em adotar uma publicidade responsável de acordo com o Código de Comunicação de Marketing Responsável de Alimentos e Bebidas da Câmara Internacional de Comércio e a legislação nacional. O nosso compromisso de marketing responsável engloba todas as comunicações que fazemos da Danone, especialmente em todos os materiais para comunicar e promover as nossas marcas ou produtos aos consumidores. Para todos eles, estamos empenhados em garantir que sejam legais, honestos e verdadeiros, utilizando apenas alegações que tenham uma base científica sólida por detrás delas: Fornecemos dados nutricionais comprovados que podem permitir aos consumidores e profissionais tomar decisões informadas. Damos especial ênfase à informação prática e fácil de usar para ajudar os consumidores a desenvolverem dietas saudáveis para si próprios e para as suas famílias. Asseguramos que toda a informação sobre os benefícios dos nossos produtos é fiável e numa língua que os consumidores possam compreender.

DANONE	A.D7	Na Danone, apoiamos as recomendações de saúde pública da Organização Mundial de Saúde (OMS) que advogam o aleitamento materno exclusivo durante os primeiros seis meses de vida e a continuação da amamentação durante até dois anos depois, combinado com a introdução segura de alimentos complementares apropriados. Publicámos um documento de posição sobre saúde e nutrição para os primeiros 1000 dias, baseado em duas convicções principais: que a amamentação deve ser protegida e promovida; e que a melhor nutrição para mães, bebés e crianças pequenas deve ser assegurada. Além disso, apoiamos os objetivos e princípios do Código Internacional de Comercialização de Substitutos do Leite Materno da OMS e temos uma política global rigorosa que rege as nossas ações para não promover produtos ou fórmulas infantis para crianças dos 0-6 meses, em qualquer parte do mundo, mesmo que as leis locais o permitam.
DANONE	A.D8	Na Danone adotámos uma diretiva corporativa interna, aplicável a todas as nossas áreas de negócio, especialmente concebida para garantir a consistência, credibilidade e precisão científica das mensagens de saúde e nutrição transmitidas através de qualquer comunicação. Por conseguinte, todas as nossas comunicações comerciais que incluam propriedades sanitárias ou nutricionais dos nossos produtos que sejam dirigidas a consumidores, clientes ou profissionais de saúde, incluindo campanhas publicitárias e promocionais em que uma marca Danone seja nomeada ou implícita, são validadas internamente de acordo com esta diretiva. As comunicações obedecem a quadros regulamentares, práticas locais e códigos de boas práticas e de ética. As comunicações são consistentes com a imagem da marca em questão e com os compromissos nutricionais da Danone. As alegações nutricionais ou de saúde são apoiadas por provas científicas. Para assegurar a consistência e a solidez de todas as comunicações da Danone aos consumidores e profissionais de saúde, são estabelecidas e implementadas diretrizes de comunicação em conformidade com o quadro regulamentar local.
DANONE	A.D9	Desde 2007 que na Danone nos comprometemos voluntariamente a fornecer informação nutricional extensa aos consumidores, tal como o quadro nutricional detalhado no verso de cada produto, informação nutricional resumida na frente de cada embalagem, e um contacto para o consumidor obter informação nutricional e sobre o produto (website, call center...) Portanto, para todos os nossos produtos destinados à população em geral, em áreas geográficas onde não entre em conflito com os regulamentos locais de rotulagem, na Danone estamos empenhados em indicar nas nossas embalagens: Os níveis recomendados de ingestão de nutrientes a consumir diariamente para uma dieta saudável. O tamanho da porção em embalagens com várias porções para indicar o número de porções ou porções nelas contidas. Informação nutricional detalhada no verso da embalagem, incluindo energia, gordura (da qual saturada), hidratos de carbono (dos quais açúcares), fibras, proteínas e sal; de 3 formas diferentes (por 100g / 100ml; por por porção; por% das doses de referência por porção). O valor energético e respetiva percentagem que representa tendo por base referência energética diária para um adulto médio ou a classificação Nutriscore do produto. De acordo com o nosso compromisso para com a transparência do consumidor, estamos progressivamente a implementar a rotulagem Nutri-Score nos nossos produtos lácteos e vegetais, bem como nas nossas águas.
DANONE	A.D10	5 exercícios aeróbicos para ajudar a diminuir o colesterol alto
DANONE	A.D11	Como reduzir o colesterol alto?
DANONE	A.D12	#THEUPGRADE BY YOPRO DESAFIA ATLETAS A ELEVAR OS TREINOS AO PRÓXIMO NÍVEL Reforço muscular, nutrição e recuperação são os três pilares fundamentais do próximo programa desportivo (só) para Real Pros
DANONE	A.D13	BOOSTER SUMMER EDITION PROMETE MANTER-VOS ATIVOS DURANTE O VERÃO Nilton Bala, Inês Abrantes, Bruno Salgueiro, Paulo Teixeira e Kiko Cabrita trazem treinos curtos, práticos e intensos, para continuarem a exercitar-se durante as férias
DANONE	A.D14	BOOSTER: CONHEÇA O NOVO DESAFIO DE YOPRO Tem saudades do seu PT? Nós tratamos do assunto. Durante as próximas semanas, a YoPRO desafia-o a participar no #BOOSTER, um programa com vários ciclos de treino que será liderado por atletas como Nilton Bala, Paulo Teixeira ou Bruno Salgueiro.
DANONE	A.D15	YOPRO DESAFIA TODOS OS REAL PROS A DESCOBRIR O 6AM CLUB O 6AM Club é a nova aplicação da YoPRO que lhe propõe apenas um objetivo: Vencer a Preguiça. A marca lança o desafio de treinar de forma incansável num novo horário: às 6h da manhã.
DANONE	A.D16	Tipos de treino: como o exercício físico contribui para um estilo de vida saudável
DANONE	A.D17	A convicção de que a saúde e o bem-estar dependem da alimentação e nutrição é a principal fonte de inspiração da atividade do Grupo Danone. Com efeito, ainda não há muitos anos a nutrição e alimentação eram vistas como preocupações de ordem pessoal, mas a progressão de doenças relacionadas com a falta ou o excesso de consumo de alimentos e nutrientes transformou o que era encarado como uma questão individual num problema da sociedade moderna. A atenção dedicada à comunidade, através de constantes estudos do consumidor ou da troca de informações com profissionais de educação, de saúde e comunicação, revela não só uma atitude de abertura ao exterior, como esse desejo de corresponder cada vez melhor às necessidades do mercado de produtos alimentares. Com o objetivo de dar resposta às crescentes expectativas da sociedade relativamente ao papel da indústria alimentar na promoção da saúde pública, o Grupo Danone concebeu em 2005 a Carta da Alimentação, Nutrição e Saúde. Esta baseia-se em cinco convicções e em nove compromissos, que não são mais do que o documento base que inspira cada uma das empresas que compõem o Grupo, entre as quais a Danone Portugal.
DANONE	A.D18	Mantendo o compromisso de apresentar produtos baixos em gordura e sem açúcares adicionados (contêm apenas açúcares naturalmente presentes), os dois novos sabores combinam fruta e chia - as sementes das novas tendências que dão um outro nível à textura do produto.
DANONE	A.D19	Um corpo saudável faz coisas impressionantes. É por isso que Corpos Danone nos convida a cuidar de nós e do nosso corpo sem abdicar daquilo que nos faz sentir bem, todos os dias*. Ciente de que o foco dos consumidores está, cada vez mais, no equilíbrio e naturalidade das suas escolhas, Corpos Danone apresenta agora duas novidades: uma fórmula de líquidos totalmente melhorada e uma nova gama NATUR.
DANONE	A.D20	Dia 29 de setembro celebra-se o Dia Mundial do Coração. A pensar nisso, e porque é urgente lembrar a importância de cuidar da bomba mais importante do corpo humano, Danacol reforça o alerta com uma novidade: a chegada de um novo formato, ainda mais económico e para consumo diário!
DANONE	A.D21	Ideal para os pequeninos comerem sozinhos sem se sujarem demasiado, Danonino apresenta saborosas gamas de iogurtes líquidos, polpas e queijinhos nas variedades que mais gostam, com fórmulas adicionadas de cálcio e vitamina D e ainda em embalagens à sua medida e com divertidas coleções para que possam desenvolver a sua imaginação. Danonino é um alimento adaptado às necessidades nutricionais dos teus filhos, sendo fonte de proteínas, cálcio e vitamina D, necessários para o crescimento e desenvolvimento normais dos ossos das crianças.
DANONE	A.D22	Nós acreditamos que ao ajudar mães e bebés a ter uma nutrição correta nos primeiros 1000 dias – desde o início da gravidez até ao bebé ter dois anos – estamos a contribuir positivamente para a sua saúde a curto e longo prazo.

		Juntamente com os pais, sempre acreditámos na importância da nutrição para bebés e crianças, incluindo a amamentação. Estamos agora a expandir os nossos horizontes dando ainda mais suporte para cada fase de desenvolvimento durante os primeiros 1000 dias de vida de um bebé. Em conjunto com apoio nutricional através da educação e de serviços, estamos a introduzir em vários mercados, produtos nutritivos para as mulheres grávidas e em fase da amamentação.
DANONE	A.D23	YoPRO é o teu aliado, porque alimenta os teus músculos de forma natural e deliciosa, e é o Nº1 em Portugal!
DANONE	A.D24	Claúdia Vieira diz sim a Activia e começa o ano com uma mensagem positiva Esta é a altura certa para começar uma revolução interior. Para dizer sim! A Cláudia Vieira, nova embaixadora de Activia, já aceitou o desafio.
DANONE	A.D25	A água é essencial para a vida na terra, é uma parte essencial do corpo humano e também essencial para completar a missão da Danone Águas, "Inspirar pessoas rumo a uma hidratação mais saudável". A nossa missão é construída na convicção de que o que bebemos durante o dia tem impacto na nossa saúde e que a água é a bebida mais saudável que podemos beber.
NMS	A.D26	Prémio DANONE Nutricia em Nutrição Artificial 2022: Ao abrigo da parceria entre a NMS e a NESTLÉ
ESB	A.ESB1	A Escola Superior de Biotecnologia desenvolve intercâmbios com múltiplas instituições dentro e fora de Portugal, incluindo centros de investigação, universidades, empresas e unidades de saúde: Modelo Continente Hipermercados, Bimbo, Danone Portugal, Imperial Produtos Alimentares, Nestlé Portugal, Primor Charcutaria Prima, Sonae, Cerealis, Vitacress Portugal

DELTA

Website	Code	CPA EXAMPLES
DELTA	A.DE1	No âmbito da sua atuação na Investigação Científica, a Diverge é responsável por diversas publicações científicas: "Chlorogenic acids composition and the impact of in vitro gastrointestinal digestion on espresso coffee from single-dose capsule Food Research International"; "Impact of microwave-assisted extraction on roasted coffee carbohydrates, caffeine, chlorogenic acids and coloured compounds Food Research International"; "Carbohydrate content, dietary fibre and melanoidins: Composition of espresso from single-dose coffee capsules Food Research International"; "Application of solid-phase extraction to brewed coffee caffeine and organic acid determination by UV/HPLC Journal of Food Composition and Analysis"; "Chapter 22: Coffee Comprehensive Analytical Chemistry"; "Stable isotope analysis for green coffee bean: A possible method for geographic origin discrimination Journal of Food Composition and Analysis".
DELTA	A.DE2	O Centro de Ciência do Café é um espaço único na Europa que nasce no coração do Alentejo, naquela que é considerada a capital do café, a Vila de Campo Maior. Ver, Tocar, Fazer e Aprender misturam-se com os aromas da torrefação, despertando novas formas de aprendizagem para todas as idades. Um espaço interativo, de caráter informativo, didático, cultural e científico, onde os visitantes podem encontrar respostas às suas questões relacionadas com o café, interagindo com equipamentos e exposições. Foi pensado e criado para promover e valorizar o património cultural português e o acervo relacionado com o café.
DELTA	A.DE3	PROCURAMOS CONSTRUIR RELAÇÕES PRÓXIMAS COM A COMUNIDADE ACADÉMICA E ABRIMOS A NOSSA PORTA A QUEM QUEIRA CONHECER E APRENDER CONNOSCO, AJUDANDO DESSA FORMA A CONSTRUIR O FUTURO DO GRUPO, A NOSSO LADO. De forma a responder aos pedidos de colaboração para trabalhos académicos que recebemos, foi criado este canal de forma a garantir uma resposta adequada e atempada aos mesmos, sejam estes trabalhos, teses ou projetos de âmbito académico e queira ter por base, ou como objeto de estudo, o Grupo Nabeiro e/ou algumas das nossas marcas/áreas de negócio. Para avaliar a nossa capacidade de resposta aos pedidos de colaboração e informação que recebemos por parte da comunidade académica, elaborámos um formulário que nos irá ajudar a garantir que todos os pedidos recebem uma resposta atempada e estruturada.
DELTA	A.DE4	O QUE ESTAMOS A FAZER PARA MUDAR O MUNDO? Pretendemos continuar a ter um papel ativo na construção de valor para a sociedade, contribuindo para a adoção de comportamentos mais responsáveis, acrescentando simultaneamente valor aos vários momentos de consumo e de partilha proporcionados pelo café. Um caminho de sustentabilidade é por isso prioritário para a Delta Cafés. A sustentabilidade está no nosso ADN e trabalhamos continuamente para a conciliação entre o modelo de crescimento económico e o modelo de sustentabilidade da nossa marca de forma a assegurar o desenvolvimento sustentado do nosso negócio.
DELTA	A.DE5	RECONHECEMOS A IMPORTÂNCIA DE DAR DE VOLTA . CRIÁMOS A Associação Coração Delta Neste ano fizemos crescer o nosso impacto na comunidade com o Coração Delta e a inauguração do Centro Pós-Graduação Comendador Rui Nabeiro.
DELTA	A.DE6	No âmbito da nossa atuação através da Associação Coração Delta, desenvolvemos diferentes projetos na vertente educacional enquadrados no objetivo de Empreendedorismo Responsável: Centro Educativo Alice Nabeiro (CEAN) Inaugurado em 2007 em Campo Maior, foi criado com o objetivo de aplicar práticas pedagógicas que promovem a criatividade e a imaginação de forma dinâmica. Destinado a crianças entre os 3 e os 12 anos, o CEAN aposta numa formação integrada e alargada através de projetos e experiências inovadoras, criando condições para a evolução de um novo paradigma educativo. Manuais de empreendedorismo "Ter Ideias para Mudar o Mundo" Adaptados aos diferentes níveis de desenvolvimento, estes manuais reconhecidos pela OCDE, estimulam a produção de ideias ou projetos empreendedores de crianças dos 3 aos 12 anos, e dos 13 aos 18. Pretende-se com estes manuais que os professores adquiram competências para que possam transmitir às crianças as áreas do conhecimento empreendedor, possibilitando o desenvolvimento de ideias em projeto. Os manuais "Ter Ideias para Mudar o Mundo" foram também objeto de dinamização nas escolas que aceitaram o desafio, tendo sido adotados em vários estabelecimentos de ensino tanto em Portugal como na região espanhola da Extremadura.
DELTA	A.DE7	Delta Cafés, patrocinador do Citómetro do Laboratório de Imunologia.
ESB	A.ESB2	A Escola Superior de Biotecnologia tem apostado na criação e desenvolvimento de parcerias com a indústria e outras organizações a nível nacional e internacional, sendo esta uma via privilegiada para fortalecer a transferência de conhecimento e tecnologia e de manter o diálogo com o mundo profissional. O resultado dessa aposta é visível e transparece em múltiplas formas de colaboração entre a ESB e diversas empresas, sempre mediadas pelos nossos estudantes dos vários níveis académicos (licenciatura, mestrado, doutoramento). Abaixo descrevem-se alguns exemplos que justificam o bom reconhecimento dos diplomados da ESB no mercado de trabalho: Cerealis, Continente, Delta, FIPA, SONAE MC,

FERRERO

Website	Code	CPA EXAMPLES
FERRERO	A.F1	ALIANÇA INTERNACIONAL DE ALIMENTOS E BEBIDAS Como membro da Aliança Internacional de Alimentos e Bebidas (IFBA), juntamente com as principais empresas da indústria alimentar, comprometemo-nos à autorregulação e à aplicação do "Enquadramento para comunicações de marketing de alimentos e bebidas responsáveis" adotado pela Câmara de Comércio Internacional (CCI), assim como os códigos de autorregulação regionais e nacionais relevantes.
FERRERO	A.F2	FEDERAÇÃO MUNDIAL DE ANUNCIANTES A Ferrero é também membro da Federação Mundial de Anunciantes (WFA), uma organização internacional que promove as boas-práticas e a eficiência no âmbito da publicidade responsável. Estamos em crer que uma autorregulação eficaz serve os interesses dos consumidores ao proporcionar comunicações verazes e precisas. Reconhecemos igualmente a necessidade de reforçar e retificar os anúncios que não se adequem a estes compromissos.
FERRERO	A.F3	EU PLEDGE Na União Europeia, a Ferrero é membro fundador do EU Pledge, assumindo o compromisso de evitar comunicações de marketing de produto dirigidas a crianças menores de 13 anos. Este tipo de compromissos permite às pessoas e às organizações alertarem para possíveis casos de não conformidade por parte das comunicações comerciais dos membros com os compromissos assumidos. Poderá encontrar mais informações aqui. Clique aqui para descarregar a versão completa do documento "Princípios de Publicidade e Marketing Ferrero
FERRERO	A.F4	A Ferrero acredita que uma autorregulamentação eficaz da publicidade fornece uma estrutura de valor para melhor servir o interesse do consumidor em receber comunicações verdadeiras e precisas. É por isso que a Ferrero, tanto individualmente como pelas Associações a que pertence (como a WFA - World Federation of Advertisers [Federação Mundial de Anunciantes] e a IFBA - International Food & Beverage Alliance [Aliança Internacional de Alimentos e Bebidas]), participa ativamente do processo de autorregulamentação em nível nacional, bem como em nível internacional.
FERRERO	A.F5	Marketing para Crianças definida pela IFBA - International Food & Beverage Alliance [Aliança Internacional de Alimentos e Bebidas], da qual somos membros. Por conseguinte, a Ferrero se compromete em todo o mundo a evitar comunicações de marketing de produto voltadas principalmente para crianças menores de treze anos nos seguintes meios de cobertura de mídia: TV, rádio, mídia impressa, cinema, mídia on-line (incluindo sites de propriedade da empresa), DVD/CD-ROM, marketing direto, posicionamento de produtos, jogos interativos, marketing ao ar livre, marketing móvel e por SMS.
FERRERO	A.F6	Marketing responsável dirigido às crianças Como compromisso global, aplicamos a Política Global da IFBA sobre comunicação de marketing e publicidade dirigida às crianças. Isto signi@ca que não realizamos publicidade dos nossos produtos na televisão, na rádio, na imprensa, no cinema, em DVD/CD-Rom ou online (incluindo nos sítios na Internet da nossa propriedade e nas redes sociais), nem mediante marketing direto, colocação de produtos, jogos interativos, marketing em exteriores, ou campanhas por telemóvel, incluindo por SMS, para audiências primariamente compostas por crianças com menos de 12 anos. Na Europa, assumimos a iniciativa do Compromisso UE, que reXete os parâmetros do nosso compromisso global e fomenta uma abordagem responsável à publicidade de produtos alimentares destinada a crianças com menos de 12 anos nos mesmos meios.
FERRERO	A.F7	KINDER: JOY OF MOVING O projecto Kinder Joy of moving nasce com o objectivo de promover a prática desportiva entre as crianças e os jovens, entendida como parte de um processo educativo que se complementa com hábitos de alimentação saudável e equilibrada. A prática do desporto representa um momento muito importante no crescimento das crianças e dos jovens, que no desporto encontram valores positivos e a possibilidade de um desenvolvimento físico e psíquico são e controlado. "Kinder Joy of moving" é o grande projecto de apoio de KINDER voleibol, o basquetebol e a vela, e que têm possibilidade de atuação a nível escolar. Para esse fim, Kinder Joy of moving estabeleceu acordos de colaboração com as várias federações desportivas e outras organizações que fomentam a prática do desporto a nível escolar, através de programas de iniciação ao desporto durante o ano lectivo. Em Portugal, Kinder Joy of moving é patrocinador da: - Federação Portuguesa de Voleibol (FPV) - Federação Portuguesa de Basquetebol (FPB) - Fundação ECOMAR
FERRERO	A.F8	O programa Kinder Joy of Moving é um projeto internacional de responsabilidade social orientado para promover um estilo de vida ativo a todas as crianças e às suas famílias, através de uma abordagem alegre à atividade física.
FERRERO	A.F9	O objetivo do projeto Kinder Joy of moving consiste na criação de ferramentas inovadoras que permitam motivar as crianças do mundo inteiro a moverem-se, e adquiriu um novo significado no âmbito da pandemia da Covid-19, encorajando o trabalho de equipa e um sentido de pertença a associações de desporto infantil e centros de ensino, bem como espalhando a alegria do movimento para manter as crianças ativas durante o confinamento.
FERRERO	A.F10	UMA ATITUDE POSITIVA EM RELAÇÃO AO MOVIMENTO FARÁ DAS CRIANÇAS DE HOJE ADULTOS MELHORES AMANHÃ. Kinder Joy of moving aproxima com alegria as crianças e suas famílias da atividade física. Todas as nossas iniciativas são inspiradas em um método inovador e cientientificamente reconhecido, baseado no conceito de brincar e capaz de promover não apenas o desenvolvimento motor, mas também cognitivo, emocional e social.
FERRERO	A.F11	O Kinder Joy of moving iniciou suas atividades em 2006 em Portugal. Ao longo dos anos o programa colaborou com diferentes Federações Nacionais de Esportes como a Federação Portuguesa de Atletismo, a Federação Portuguesa de Natação, a Federação Portuguesa de Basquete e várias instituições, como o município de Lisboa com o qual o KJOM tem colaborado para a organização das Olimpíadas Estudantis locais (Olisipiades). Atualmente o KJOM colabora com a Federação Portuguesa de Vôlei com a qual realiza os programas esportivos escolares GIRAVÔLEI, GIRA PLUS e GIRA PRAIA, que permitem que mais de 140.000 crianças e adolescentes pratiquem vôlei todos os anos em suas modalidades (indoor, vôlei de praia). Através da Fundación Ecomar, realizamos programas todos os verões em dezenas de clubes náuticos para possibilitar que as crianças se aproximem do universo marinho e possam apreciar sua importância, navegando e realizando atividades educativas sobre a importância de preservar o mar e a costa. Ao longo dos anos, centenas de milhares de crianças e seus familiares tiveram a oportunidade de experimentar várias atividades através do KJOM
FERRERO	A.F12	135.000 crianças 100 eventos
FERRERO	A.F13	PROTEGER O AMBIENTE Estamos a trabalhar para impulsar a eficiência ambiental nas nossas operações, reduzindo emissões e melhorando a circularidade das nossas embalagens.

FERRERO	A.F14	SUSTENTABILIDADE DO APROVISIONAMENTO DE INGREDIENTES Com os nossos programas, empoderamos os agricultores no fim da cadeia de fornecimento, aumentando a transparência e conservando as respetivas envolventes naturais.
FERRERO	A.F15	Estamos empenhados no respeito e promoção dos direitos humanos, e na prevenção e mitigação de qualquer impacto negativo das nossas operações, através de uma abordagem aos direitos humanos baseada na devida diligência e nos Princípios Orientadores das Nações Unidas sobre Negócios e Direitos Humanos, na Carta Internacional dos Direitos Humanos, e nas Convenções Fundamentais da Organização Internacional do Trabalho (OIT).
FERRERO	A.F16	O Código baseia-se nos princípios da Ferrero, cumpre o Código de Ética do Grupo e define as práticas da Ferrero nas seguintes áreas: 1. Excelência na qualidade e segurança dos produtos; 2. Compromisso com a proteção dos direitos humanos; 3. Proteção e sustentabilidade ambiental; 4. Garantia das condições do local de trabalho; 5. Integridade comercial.

FIPA

Website	Code	CPA EXAMPLES
FIPA	A.FI1	Diálogo Europeu Posicionar a indústria agroalimentar nacional no contexto europeu e assegurar a sua representatividade na União Europeia é um dos focos da FIPA. Como tal, trabalhamos em conjunto com as nossas congéneres europeias, no seio da FoodDrinkEurope, a confederação europeia das indústrias agroalimentares.
FIPA	A.FI2	CONSUMIDOR | Promover sinergias entre a indústria agroalimentar e os decisores políticos, parceiros e sociedade em geral, com vista à promoção de escolhas informadas e estilos de vida saudáveis.
FIPA	A.FI3	FIPA em audição Parlamentar FIPA, enquanto membro da PARCA - Plataforma de Acompanhamento das Relações na Cadeia Agroalimentar, foi ouvida, a pedido do PSD, na Comissão de Agricultura e Pescas.
FIPA	A.FI4	CONSUMIDOR | Promover sinergias entre a indústria agroalimentar e os decisores políticos, parceiros e sociedade em geral, com vista à promoção de escolhas informadas e estilos de vida saudáveis.
FIPA	A.FI5	Trabalhar em estreita colaboração com os principais parceiros, de forma a promover uma resposta global e integrada aos desafios colocados pelas complexas mudanças sociais, económicas e ambientais.
FIPA	A.FI6	Cooperação e colaboração Conquistar objetivos e a confiança dos parceiros passa pelo trabalho de estreita colaboração e cooperação. É desta forma que a FIPA tem vindo a integrar diferentes fóruns e organizações: 8º CONGRESSO FIPA FILIAÇÕES CIP – Confederação Empresarial de Portugal A.GS1 Portugal Fórum do Consumo Aliança Contra a Fome e Má Nutrição Portugal AINIA - Centro Tecnológico LIFE RENDER | RESULTADOS PARTICIPAÇÕES INSTITUCIONAIS CCDM - Centro de Competências da Dieta Mediterrânica | Membro do Conselho Dinamizador para a Salvaguarda e Promoção da Dieta Mediterrânica CETRAD - Centre of Transdisciplinary Development Studies da UTAD | Membro do Fórum de stakeholders CNCDA - Comissão Nacional de Combate ao Desperdício Alimentar | Membro do Painel Consultivo Missão Continente | Membro do Conselho Consultivo da Comissão Especializada “Missão Continente Consciente” PARCA – Plataforma de Acompanhamento das Relações na Cadeia Agro-Alimentar | Membro PDR 2020 – Programa de Desenvolvimento Rural 2014-2020 | Membro da Comissão de Acompanhamento PortFIR – Portal de Informação Alimentar | Membro do Grupo Operacional Rede Portuguesa sobre Informação Microbiológica de Alimentos | Membro do Grupo Operacional Consultivo
FIPA	A.FI7	O "Spread European Safety - European Economic Interest Grouping" (SPES GEIE) é um Agrupamento Europeu de Interesse Económico, fundado por 13 federações alimentares europeias, entre as quais a FIPA. O objetivo do SPES é facilitar e desenvolver as atividades dos seus membros relevantes para a promoção e execução a nível da União Europeia de estudos sobre a segurança e a qualidade dos alimentos ao longo da cadeia alimentar, bem como a utilização e divulgação dos resultados relevantes.
FIPA	A.FI8	O FoodSafety4EU pretende desenvolver e lançar novas ferramentas e uma plataforma digital para promover a segurança dos alimentos e apoiar a construção do futuro Sistema de Segurança Alimentar da Europa. Será desenvolvido por um consórcio europeu composto por um conjunto de intervenientes de 23 países que operam, direta e indiretamente, na cadeia alimentar, desde autoridades de segurança alimentar, investigadores, federações da indústria agroalimentar, instituições europeias e representantes da sociedade civil. O projeto será desenvolvido ao longo de três anos (2021-2023) é financiado pelo fundo europeu HORIZONTE 2020 e tem a coordenação do Consiglio Nazionale Delle Ricerche da Itália.
FIPA	A.FI9	Cooperação entre indústria e saúde A Associação Internacional de Adoçantes junta-se à Federação Internacional de Diabetes para assinalar o Dia Mundial da Diabetes com campanha mundial focada na prevenção e no acesso a cuidados.
FIPA	A.FI10	Defender e promover o interesse das suas associadas em áreas como a qualidade e segurança alimentar, nutrição e saúde, confiança e informação ao consumidores, exportação e internacionalização, investigação, desenvolvimento e inovação e sustentabilidade.
FIPA	A.FI11	A FIPA associa-se aos Prémios “Viver Saudável”, uma iniciativa que pretende distinguir o que melhor se faz na nutrição em Portugal e os nutricionistas. Os vencedores desta 1ª edição serão conhecidos em novembro.
FIPA	A.FI12	FIPA debate nutrição e alimentação A FIPA marca presença na 20ª edição do Congresso de Nutrição e Alimentação, que decorre em setembro, com uma reflexão sobre sustentabilidade e inovação nos sistemas alimentares do futuro.
FIPA	A.FI13	Indústria e nutrição lado-alado Esta foi a perspetiva abordada pela FIPA no webinar “NutriSessions”, promovido pela revista Viver Saudável, dedicado ao tema “(R)Evolução Industrial: Oportunidades para os nutricionistas”.
FIPA	A.FI14	PA debate futuro da alimentação A FIPA, em colaboração com a Deloitte, promove o webinar “Futuro da Alimentação – perspetivas, tendências, caminhos”, que acontece a 20 de maio, entre as 9h30 e as 11h00, e que conta com a participação da APED, CAP e Ordem dos Nutricionistas.
FIPA	A.FI15	Alimentar com sustentabilidade? A que nos devemos referir quando falamos de “alimentar com sustentabilidade”? Esta foi uma das questões abordadas pela FIPA no VIII Encontro de Nutrição e Alimentação, organizado pela ANEN.
FIPA	A.FI16	Durante as últimas legislaturas, a FIPA tem sido parceira incontornável na definição das políticas nacionais para a internacionalização do setor agroalimentar, tendo apresentado aos sucessivos Governos os seus “Contributos para uma Visão Estratégica”.
FIPA	A.FI17	Pós-Graduação Sustentabilidade Alimentar Instituto Universitário de Ciências da Saúde lança primeira edição da Pós-Graduação sobre Sustentabilidade Alimentar já em janeiro de 2024.
FIPA	A.FI18	REFORMULAÇÃO NUTRICIONAL O progresso da reformulação nutricional dos alimentos em Portugal é apresentado a 15 de fevereiro, numa sessão que conta com participação da FIPA, APED, DGS, INSA e da NielsenIQ.

FIPA	A.FI19	ALIMENTOS COM MENOS 11% DE SAL E AÇÚCAR Este é o resultado preliminar do trabalho desenvolvido no âmbito do protocolo de reformulação nutricional assinado pela FIPA, APED e um conjunto de associações setoriais com o Ministério da Saúde e a DGS.
FIPA	A.FI20	REFORMULAÇÃO EM DESTAQUE A FoodDrinkEurope aborda o empenho da indústria agroalimentar europeia na reformulação nutricional, na recente edição da sua newsletter. Portugal está entre os destaques.
FIPA	A.FI21	FIPA no combate à fome e má nutrição FIPA participou, na qualidade de associado fundador, na Assembleia Geral da Aliança Contra a Fome e Má Nutrição em Portugal que se realizou no dia 20 de novembro.
FIPA	A.FI22	Apresentadas recomendações para pescado FIPA presente na sessão de apresentação pública das recomendações para o consumo de pescado para a população portuguesa.
FIPA	A.FI23	FIPA e APED promovem informação ao consumidor Está disponível a nova edição do "Guia de Informação ao Consumidor", que conta com um conjunto de orientações práticas sobre as regras de rotulagem dos alimentos.
FIPA	A.FI24	FIPA apoia Bimbo Global Race "Sétima edição da Bimbo Global Race" volta a acontecer na vertente física, na Avenida Ribeira das Naus, em Lisboa, a 25 de setembro.
PARLAMENTO	A.PAR1	Audição Parlamentar Nº 34-CAPes-XV: Audição, a requerimento do Grupo Parlamentar do PSD, dos representantes dos membros da "PARCA - Plataforma de Acompanhamento das Relações na Cadeia Agroalimentar" sobre o estado de transparência, equilíbrio e equidade nas relações comerciais entre os diversos intervenientes ao longo da cadeia agroalimentar (produção, transformação e distribuição), designadamente na repartição de valor
INSA	A.INSA1	Produtos alimentares com menos 11% do teor de sal e açúcar em três anos: O processo de reformulação dos produtos alimentares é um compromisso entre o Estado, aqui representado pelo Ministério da Saúde e a DGS, e as principais associações do setor alimentar, Federação das Indústrias Portuguesas Agro-Alimentares (FIPA), Associação Portuguesa de Empresas de Distribuição (APED) e outras associações sectoriais.
INSA	A.INSA2	No próximo dia 15 de fevereiro 2022, pelas 15 horas, a Direção-Geral da Saúde (DGS) organiza o webinar "Redução do teor de sal e açúcar nos alimentos", onde serão apresentados os resultados do Progresso da Reformulação dos Alimentos em Portugal durante o período de 2018 a 2021, uma iniciativa da DGS, Instituto Nacional de Saúde Doutor Ricardo Jorge (INSA), Federação das Indústrias Portuguesas Agro-alimentares (FIPA), Associação Portuguesas de Empresas de Distribuição (APED) e NielsenIQ. A melhoria da composição nutricional dos alimentos disponíveis insere-se no âmbito da estratégia alimentar e nutricional nacional desenvolvida pelo Programa Nacional para a Promoção da Alimentação Saudável (PNPAS) e pela Estratégia Integrada para a Promoção da Alimentação Saudável (EIPAS), sendo a reformulação dos produtos alimentares em Portugal um amplo compromisso formalizado em 2019 entre as entidades envolvidas.
PNPAS	A.PNPAS1	Os parceiros do PNPAS | Instituições do setor agroalimentar: FIPA, APED
ESB	A.ESB2	A Escola Superior de Biotecnologia tem apostado na criação e desenvolvimento de parcerias com a indústria e outras organizações a nível nacional e internacional, sendo esta uma via privilegiada para fortalecer a transferência de conhecimento e tecnologia e de manter o diálogo com o mundo profissional. O resultado dessa aposta é visível e transparece em múltiplas formas de colaboração entre a ESB e diversas empresas, sempre mediadas pelos nossos estudantes dos vários níveis académicos (licenciatura, mestrado, doutoramento). Abaixo descrevem-se alguns exemplos que justificam o bom reconhecimento dos diplomados da ESB no mercado de trabalho: Cerealis, Continente, Delta, FIPA, SONAE MC,

IGLO

Website	Code	CPA EXAMPLES
IGLO	A.I1	Comer uma dieta saudável e equilibrada é fundamental para uma boa saúde. Uma dieta flexitariana, também chamada 'vegetarianismo parcial' ou 'semivegetarianismo', pode ser equilibrada e saudável e os produtos Iglo podem ser facilmente incorporados. De uma forma simplificada, a dieta flexitariana é uma dieta baseada em produtos vegetais que permite o consumo de produtos animais como carne ou peixe. (...) Por exemplo, porque não experimenta uma reconfortante sopa de ervilhas acompanhada de pão integral, um almoço ligeiro composto por uma salada de quinoa e espinafre ou um jantar gratificante de caril de grão-de-bico com arroz - tudo delicioso, fornecendo um conjunto completo de aminoácidos essenciais. Para conhecer a nossa gama completa de produtos vegetais, visite a nossa página.
IGLO	A.I2	Melhor Nutrição: Queremos inspirar as famílias a optarem por uma dieta mais equilibrada e a terem um estilo de vida mais saudável. Graças à nossa vasta gama de peixe, vegetais e frango estamos numa excelente posição para o fazer. Todas as nossas receitas são avaliadas usando a nossa Ferramenta de Perfil Nutricional personalizada, que confirmou que 80% dos nossos produtos são "escolhas saudáveis".
IGLO	A.I3	Com Green Cuisine é fácil fazeres a tua parte em prol do meio ambiente e de uma alimentação saudável, seja substituindo os clássicos nuggets por uns igualmente deliciosos nuggets 0% frango, o teu hambúrger favorito por um hambúrger 0% carne, ou simplesmente por optares por uns fingers repletos de espinafres, ou umas deliciosas veggie bowls. Green Cuisine torna a preparação de uma refeição repleta de vegetais em algo delicioso e simples de preparar. Bem-vindos à Era Vegetal!
IGLO	A.I4	PEGADA DE CARBONO A Proteína Vegetal Iglo Green Cuisine tem, em média, uma pegada 80% mais pequena comparada com a da carne vermelha.
IGLO	A.I5	RECURSOS TERRESTRES Em média, a gama de Proteína Vegetal Iglo Green Cuisine usa menos 71% de terra que a carne vermelha.
IGLO	A.I6	CONSUMO DE ÁGUA A nossa gama de Proteína Vegetal Iglo Green Cuisine tem um consumo de água, em média, 77% menor comparado com a carne vermelha.
IGLO	A.I7	Iglo e o Futuro dos Oceanos Somos o maior utilizador do rótulo ecológico do MSC, a nível global, e, no ano passado, celebrámos 20 anos de trabalho em conjunto.
IGLO	A.I8	"A Bordo com o Capitão" é uma ação de educação ambiental da Iglo, que visa sensibilizar o público infantojuvenil, dos 6-12 anos, para o problema da pesca excessiva e para o consumo responsável de peixe. O projeto materializa-se através da presença em escolas nacionais durante o ano letivo, aliando as temáticas promovidas ao programa escolar, bem ao longo de todo o ano civil em diferentes meios com o intuito de levar esta mensagem às famílias. Embarque nesta viagem por um ecossistema marinho mais sustentável!
IGLO	A.I9	Convidar os mais novos a tornarem-se verdadeiros marinheiros da sustentabilidade e a embarcar numa jornada épica rumo à pesca responsável. É este o cerne da iniciativa "A Bordo com o Capitão", em parceria com a ANP|WWF, que vai entregar, a mais de trezentas escolas portuguesas, um divertido jogo que ajuda as crianças a Xcarem a conhecer as

		principais espécies dos nossos mares, rios e estuários, o que as ameaça e ainda a perceber o que podem fazer no seu dia-a-dia para as proteger, divulgou em comunicado. Segundo a mesma fonte, através de jogabilidade estratégica, trabalho em equipa e resolução de desafios, "A Bordo com o Capitão" é um projeto "inovador que visa sensibilizar e educar crianças de todo o país para a importância da pesca sustentável, da conservação das espécies marinhas e de água doce e para a diferença que as suas escolhas alimentares podem fazer". Com este jogo de cartas interativo, a Iglo e a ANP|WWF trazem assim o tema da sustentabilidade nos ecossistemas aquáticos para a sala de aula de uma forma dinâmica e divertida. O projeto vai ainda percorrer as escolas da região da Grande Lisboa, ao longo do ano letivo, num roadshow educativo com ações que aliam a temática da sustentabilidade dos mares, rios e estuários ao programa escolar. "Uma aventura épica, com a presença do Capitão Iglo, para angariar marinheiros da sustentabilidade!", sublinha a nota. No total, acrescenta a mesma fonte, a iniciativa vai impactar 537 turmas e mais de 11 mil alunos. "Com desafios únicos que incentivam as crianças a usarem a sua criatividade, pensamento estratégico e conhecimento sobre a pesca sustentável e conservação das espécies para resolvê-los, 'A Bordo com o Capitão' é indicado para os alunos do 1.º ciclo". A ANP|WWF apoia desde o primeiro momento esta iniciativa, tendo participado na curadoria e revisão dos conteúdos. "A educação ambiental e a literacia dos oceanos representam pilares fundamentais para a construção de um planeta onde as pessoas vivam em harmonia com a natureza. A iniciativa da Iglo demonstra o potencial alavancador das empresas para a promoção da cidadania afetiva e aumento da literacia ambiental, integrando a natureza na 'sala de aula' e permitindo o desenvolvimento de novas competências através da experimentação e dinamização de ferramentas pedagógicas inovadoras", disse Cátia Nunes, Coordenadora do Programa de Educação da ANP|WWF. "A pesca excessiva é um dos problemas que os oceanos enfrentam e que a nós, Iglo, mais nos preocupa. Ao longo dos anos, a busca por vezes desregrada por recursos marinhos tem vindo a pôr em risco a sobrevivência dos mesmos, pelo que é urgente e essencial apostar e valorizar práticas de pesca sustentável certiFIcada que garantam a boa renovação e continuidade das espécies" refere Inês Teixeira, Diretora de Marketing da Iglo, acrescentando que "o trabalho com as escolas e com as gerações mais jovens, que estão muito mais despertas para estes temas, é fundamental e é um dos eixos de atuação da nossa estratégia de sustentabilidade, através da qual a Iglo quer contribuir para uma melhor educação sobre a problemática da conservação das espécies marítimas e as ações que todos podemos tomar." "A Bordo com o Capitão" inclui ainda outras atividades pedagógicas que estão disponíveis para crianças, pais e educadores em iglo.pt/a-bordo-com-o-capitao Um jogo que alerta para a sustentabilidade dos oceanos Criado e desenvolvido pela KICO – Kids Communication Agency, agência criativa focada na comunicação para crianças do 0 aos 14, que alia o pensamento criativo à pedagogia, cada carta do jogo "A Bordo com o Capitão" representa um animal ou um conceito relacionado com o ecossistema aquático, com informação sobre a espécie e um desafio que as crianças devem resolver num curto espaço de tempo. A sustentabilidade é o eixo central do jogo, por isso, a quantidade de cartas de cada espécie no jogo resete também a existência destas espécies marinhas na natureza e a escolha do baralho implica que as crianças tenham de optar entre espécies mais ou menos ameaçadas, com base no seu estado de conservação. Em 2022, a Iglo lançou o livro "Os peixes que fugiram da história", que procurou sensibilizar as crianças para esta problemática, pondo a claro as consequências da pesca excessiva. "Mas se,há um ano, os peixes fugiram da história, em 2023 e 2024 vamos aprender como podemos trazê-los de volta, dando continuidade ao nosso investimento na sensibilização dos mais novos para a temática da sustentabilidade dos oceanos e da pesca sustentável", afirma a Diretora de Marketing da Iglo. Membro fundador do MSC (Marine Stewardship Council), bem como praticante e promotora da pesca sustentável, a Iglo tem como objetivo ajudar as pessoas a fazer escolhas de alimentação nutritivas e sustentáveis. "A Bordo com o Capitão" é mais uma iniciativa rumo a essa consciencialização.

IMPERIAL

Website	Code	CPA EXAMPLES
IMPERIAL	A.IM1	Vamos ajudar a nossa economia neste período especial. Consumir e divulgar as marcas portuguesas é proteger a nossa economia, reduzir o desemprego e o impacto social e económico desta crise. Partilhe as suas escolhas com a hashtag: #EuEscolhoMarcasPortuguesas #portugal #economia
IMPERIAL	A.IM2	Coma chocolate: Um alimento que melhora a sua vida
IMPERIAL	A.IM3	A Regina é a marca portuguesa de chocolates mais reconhecida e a mais popular em Portugal, recolhendo o afeto de gerações inteiras.
ESB	A.ESB1	A Escola Superior de Biotecnologia desenvolve intercâmbios com múltiplas instituições dentro e fora de Portugal, incluindo centros de investigação, universidades, empresas e unidades de saúde: Modelo Continente Hipermercados, Bimbo, Danone Portugal, Imperial Produtos Alimentares, Nestlé Portugal, Primor Charcutaria Prima, Sonae, Cerealis, Vitacress Portugal
ESB	A.ESB3	Lançado em outubro de 2021, num evento público, o INSURE.hub está já a cumprir o seu grande objetivo: criar um ecossistema vibrante, nacional e internacional, de conhecimento transdisciplinar de âmbito circular, sustentável e regenerativo. São já mais de 30 as entidades que se associaram a esta iniciativa que resulta da mobilização da Universidade Católica Portuguesa, no Porto, através das suas Faculdades - Católica Porto Business School e Escola Superior de Biotecnologia - e da Planetiers New Generation, em conjunto com organizações nacionais e internacionais que se constituem como líderes de pensamento e agentes de transformação para a Sustentabilidade e a Regeneração: Cerealis, Imperial, Vitacress

JERÓNIMO MARTINS

Website	Code	CPA EXAMPLES
JERÓNIMO MARTINS	A.JM1	O Pingo Doce é um membro ativo nas comissões técnicas dedicadas à qualidade alimentar da Associação Portuguesa de Empresas de Distribuição (APED).
JERÓNIMO MARTINS	A.JM2	Com o objetivo de promover a saúde através da alimentação, fomentando escolhas informadas e bons hábitos alimentares, desenvolvemos, em parceria com a CUF, o programa "A Saúde Alimenta-se". Esta iniciativa dá a conhecer

		as propriedades de algumas frutas e legumes, bem como o papel que podem desempenhar numa alimentação diversificada e equilibrada, ajudando a selecionar aqueles que mais se adequam a cada estilo de vida
JERÓNIMO MARTINS	A.JM3	Foi dada continuidade às parcerias com organizações que visam contribuir para uma alimentação saudável, das quais se destacam: − a divulgação de receitas no site da Direcção-Geral de Saúde, no âmbito do Programa Nacional de Promoção da Alimentação Saudável (PNPAS) e de artigos sobre Dieta Mediterrânica, com a participação de responsáveis do PNPAS, na revista bimestral "Sabe Bem" do Pingo Doce. Estes artigos são também divulgados no blog do PNPAS "Nutrimento"; − a identificação de produtos Pingo Doce sem glúten, com a Associação Portuguesa de Celíacos; − a Fundação Portuguesa de Cardiologia, para a identificação de produtos de charcutaria com menor teor de gordura e sal com o símbolo "Escolha Saudável"; − a Faculdade de Nutrição e Alimentação da Universidade do Porto para a caracterização do consumo português, a nivel regional, de produtos contribuidores para a aquisição de sódio e potássio, e para a promoção da Dieta Mediterrânica e de exercício físico multicomponente nas faixas etárias mais envelhecidas.
JERÓNIMO MARTINS	A.JM4	Conferência Dieta Mediterrânica à Portuguesa A Dieta Mediterrânica está classificada como Património Cultural Imaterial da Humanidade pela UNESCO e merece ser mais divulgada, compreendida e acarinhada pelos portugueses. Foi com este propósito que o Grupo Jerónimo Martins organizou a Conferência Dieta Mediterrânica à Portuguesa. O evento decorreu no dia 16 de outubro de 2020 e contou com o apoio institucional da Câmara Municipal de Lisboa (no âmbito da Lisboa Capital Verde Europeia 2020), da Faculdade de Ciências da Nutrição e Alimentação da Universidade do Porto e teve o patrocínio científico da Direção-Geral da Saúde. A curadoria científica esteve a cargo de Pedro Graça, diretor da Faculdade de Ciências da Nutrição e Alimentação da Universidade do Porto. Alinhamento: Pedro Soares dos Santos | Presidente do Grupo Jerónimo Martins; José Sá Fernandes | Vareador Ambiente | Clima e Energia | Estrutura Verde – Câmara Municipal de Lisboa; Paulo Farinha Marques | Arquiteto paisagista e professor associado na Faculdade de Ciências da Universidade do Porto; Alejandro Santos | Docente e investigador da Faculdade de Ciências da Nutrição e Alimentação da Universidade do Porto; Maria João Gregório | Diretora do Programa Nacional para a Promoção da Alimentação Saudável da Direção-Geral da Saúde; Mónica Truninger | Socióloga e investigadora auxiliar no Instituto de Ciências Sociais da Universidade de Lisboa; Pedro Graça | Diretor da Faculdade de Ciências da Nutrição e Alimentação da Universidade do Porto; Maria do Céu Antunes | Ministra da Agricultura
JERÓNIMO MARTINS	A.JM5	Sensibilização Adotamos várias estratégias de comunicação com os nossos clientes no sentido de os sensibilizar para opções de alimentação saudável e escolhas sustentáveis. JULIANA Há mais de dez anos que os princípios da dieta mediterrânica, classificada como Património Cultural Imaterial da Humanidade pela UNESCO (Organização das Nações Unidas para a Educação, a Ciência e a Cultura), estão na base do desenvolvimento de produtos e da comunicação do Pingo Doce. Desde 2020, e na sequência da conferência Dieta Mediterrânica à Portuguesa organizada pelo Grupo, que o Pingo Doce abraçou uma nova marca – Juliana – como elemento agregador da oferta alimentar relacionada com a dieta mediterrânica. LIVROS E REVISTAS A revista bimestral do Pingo Doce "Sabe Bem", com uma tiragem média de 150 mil exemplares, é um dos principais meios de comunicação da dieta mediterrânica, contendo receitas que também incentivam o reaproveitamento de excedentes alimentares. (…) Em 2019, 2020 e 2021 o Pingo Doce editou um livro de receitas, todas relacionadas com os princípios da dieta mediterrânica, com tiragens de centenas de milhar de exemplares. (…) REDES SOCIAIS E WEBSITES Os conselhos de confeção e sugestões de acompanhamento com vegetais ou fruta são também divulgados nos websites e nas redes sociais das Companhias. São ainda publicadas receitas que incentivam o reaproveitamento de excedentes alimentares.
JERÓNIMO MARTINS	A.JM6	Menos Sal Portugal Em parceria com a CUF, desenvolvemos o Programa Menos Sal Portugal que visa alertar para o consumo excessivo de sal e promover a redução da sua ingestão através da mudança de hábitos alimentares.
JERÓNIMO MARTINS	A.JM7	Programa Menos Sal Portugal O programa integra um estudo científico coordenado pelo Professor Jorge Polónia da Faculdade de Medicina da Universidade do Porto e pela Professora Conceição Calhau da Nova Medical School. Este estudo avaliou o impacto na saúde dos portugueses de um programa de educação de hábitos alimentares saudáveis, que privilegiam a redução do consumo de sal.<br><br>Resultados do Estudo Científico Pela primeira vez em Portugal, realizou-se um estudo populacional de alteração de hábitos alimentares, com monitorização rigorosa do consumo de sal e de potássio e das suas consequências diretas na saúde. Os investigadores concluíram que a diminuição da ingestão de sal e o aumento da ingestão de potássio, a par da mudança dos padrões alimentares, estão diretamente associados a uma signi1cativa redução da pressão arterial e a potenciais benefícios cardiovasculares. Com os ganhos em saúde obtidos com a redução da ingestão de sal, muitos doentes hipertensos poderão evitar iniciar medicação para a hipertensão arterial ou até mesmo conseguir reduzir a medicação. Pela redução da ingestão de sal, em média, os participantes reduziram a pressão arterial (SBP) em 2,1 mm hg. Os cinco maiores estudos observacionais internacionais indicam que pequenas diferenças no índice SBP resultam numa signi1cativa redução do risco de desenvolvimento de doenças cardiovasculares, diminuindo a probabilidade de morte por enfarte em 10%, a mortalidade por doenças cardiovasculares em 9% e outras causas de mortalidade em 7%. Esta conclusão mostra que é fundamental que os portugueses reduzam para metade o consumo de sal de forma a prevenirem-se das doenças cardiovasculares.
JERÓNIMO MARTINS	A.JM8	NUTRI-SCORE Em 2021, o Pingo Doce e a Biedronka adotaram o Nutri-Score, uma simbologia na face frontal dos produtos que é mais intuitiva sobre a qualidade nutricional de alimentos pré-embalados, que estabelece uma classificação nutricional de A (qualidade nutricional elevada) a E (produtos que devem ser consumidos com menor frequência), sendo acompanhada de um código de cores. Nestes dois países, em 2022 o número de artigos com esta simbologia cresceu 249% face ao ano anterior, para os 565 artigos.
JERÓNIMO MARTINS	A.JM9	Nutri-Score Uma das principais preocupações do Pingo Doce é ajudar o consumidor a fazer opções alimentares de forma consciente e informada. Um papel que assume ativamente, através do pilar estratégico "Promover a saúde pela alimentação". Desde 2006, que o Pingo Doce introduziu voluntariamente a informação nutricional nas embalagens dos seus produtos de Marca Própria, indicando a percentagem de Valores de Referência médios para adultos e crianças. Agora, com a inclusão do Nutri-Score, o cliente pode fazer uma leitura rápida do perfil nutricional do produto sem nunca perder a informação completa que consta da tabela nutricional.
JERÓNIMO MARTINS	A.JM10	Desenvolvimento de produtos de Marca Própria e perecíveis;

		Reformulações Nutricionais; As linhas orientadoras do desenvolvimento de produtos de Marca Própria e perecíveis reforçam os princípios enunciados na nossa Política Nutricional ; Restrições quanto à utilização de corantes, conservantes e outros aditivos sintéticos supérfluos ; Quantidades máximas admitidas de alguns ingredientes como o sal, açúcar e gordura ; Estratégias de reformulação nutricional ; Diversidade na oferta e desenvolvimento de produtos para pessoas de diferentes faixas etárias e com necessidades ou preferências nutricionais específicaS ; Materiais de embalagem permitidos para contacto com géneros alimentares ; Rótulos que incluam informação clara e intuitiva aos clientes sobre saúde, nutrição, porções e promoção de estilos de vida saudáveis ; Planos de monitorização de produto (designadamente testes sensoriais), auditorias e controlos laboratoriais.
JERÓNIMO MARTINS	A.JM11	PORTUGAL Em 2022, foram feitas 70 reformulações nutricionais aos produtos de Marca Própria do Pingo Doce e do Recheio. Destaque para as mais de 200 toneladas de açúcar que deixaram de entrar no mercado, como resultado da revisão das receitas de 58 produtos (como a gama de iogurtes das Marcas Próprias Pingo Doce e Amanhecer e os cereais Chocolocos, Cookie Bitz e Estrelinhas) e para a remoção de 1,8 toneladas de gordura e 60,2 de gordura saturada. No caso da gordura saturada, foi dada especial atenção às batatas fritas, bolachas e molhos (reduções entre os 29% e os 84%). O sal foi revisto em 16 produtos, o que resultou em 16,9 toneladas que deixaram de ser consumidas pelos nossos clientes. A remoção de corantes artificiais foi feita na totalidade dos produtos de Marca Própria. Às bolachas Zoo Pingo Doce, muito consumidas por públicos infantis, adicionaram-se 1,5 toneladas de fibra e retiraram-se 1,8 toneladas de gordura.
JERÓNIMO MARTINS	A.JM12	Em Portugal e na Polónia, garantir que 90% dos produtos de Marca Própria não contêm corantes artificiais nos seus ingredientes diretos, até 2023.
JERÓNIMO MARTINS	A.JM13	EIPAS n.º 1a): Redução de açúcares totais e teor de sal em cereais matinais: − redução do teor médio de açúcares totais em 10% até 2022 (linha de base de 23,44g/100g). EIPAS n.º 1b): Redução do teor de açúcares totais e teor de sal nos cereais de pequeno-almoço: − redução do teor de sal para um máximo de 1g/100g em cereais de pequenoalmoço para crianças até 2022 (valorbase de 0,71g/100g); − redução do teor médio de sal para 1g/100g em cereais matinais até 2022 (valor-base de 0,71g/100g). EIPAS n.º 2a): Redução do teor de sal em refeições pré-embaladas prontas a consumir refrigeradas ou congeladas Takeaway (Top 5 Marcas Próprias Vendas) – Lasanha Bolognesa, Arroz de Pato, Bacalhau à Brás, Bacalhau com Natas e Pizzas: − − refeições: 1g de sal/100g até 2022; refeições: 0,9g/100g até 2023. EIPAS n.º 2b): Redução do teor de sal em refeições pré-embaladas prontas a consumir refrigeradas ou congeladas Takeaway (Top 5 Marcas Próprias Vendas) – Lasanha Bolognesa, Arroz de Pato, Bacalhau à Brás, Bacalhau com Natas e Pizzas: − pizzas: redução de 10% no teor médio de sal até 2022 (valor-base 1,78g/100g). EIPAS n.º 3: Redução do açúcar total em refrigerantes: − redução média dos açúcares totais em 10% até 2022 (valor-base de 5,94g/ml). EIPAS n.º 4: Redução de açúcares adicionados em Iogurtes e Queijos Aromatizados, Leites Fermentados e Leites Aromatizados (excluem-se os produtos sem adição de açúcar e/ou edulcorantes): EIPAS n.º 5: Redução do teor de sal em batatas fritas e snacks salgados (inclui cereais ou salgados de batata): − teor médio de sal em 12% até 2022 (valorbase 1,25g/100g). EIPAS n.º 6: Redução de açúcares totais em néctares (exceto néctares de frutas sem adição de açúcar ou adoçantes) EIPAS n.º 7: Redução do teor de sal no pão de fabrico próprio: − 1g de sal/100g de pão até 2021. EIPAS n.º 8: Redução do teor de sal nas Sopas de Marca Própria (excluem-se as sopas com adição de proteína): − - 0,3g de sal/100g de sopa até 2023 (média ponderada). Em progresso. A média dos teores totais de açúcares das referências de néctares encontrava-se 16% acima do valor base e da média de mercado. teor médio de açúcares totais em 7% até 2023 (linha de base de 8,72g/ml, valor revisto em Março 2018)
JERÓNIMO MARTINS	A.JM14	(…) procuramos que os produtos destinados a públicos infantis tenham um perfil nutricional saudável e melhor que o benchmark de cada mercado. Em Portugal, 20% dos artigos Pingo Doce e Amanhecer mais consumidos por públicos infantis tinham perfis melhores ao benchmark e 80% um perfil igual. No Pingo Doce o destaque vai para os sumos vegetais Veggie sem adição de açúcar e para as massas com formatos de animais destinadas a crianças.
JERÓNIMO MARTINS	A.JM15	A qualidade e a segurança alimentar dos produtos que vendemos são a base do capital de reputação das nossas insígnias. É através dos nossos técnicos, dos auditores externos e de laboratórios independentes e acreditados, bem como do nosso próprio laboratório de biologia molecular — que, desde 2021, verifica a autenticidade dos ingredientes para prevenir a fraude alimentar — que zelamos pela qualidade e segurança dos nossos produtos de Marca Própria e perecíveis.
JERÓNIMO MARTINS	A.JM16	Para concretizar esta política, o Grupo Jerónimo Martins está orientado pelos princípios/recomendações da Organização Mundial da Saúde (OMS):<br><br>• Garantir a segurança dos produtos através da implementação de esquemas adequados para o controlo da cadeia de abastecimento alimentar, assegurando um elevado nível de proteção e confiança dos consumidores;<br>• Garantir o cumprimento da legislação;<br>• Oferecer uma grande variedade de produtos, de modo a permitir uma alimentação equilibrada e variada;<br>• Aumentar a disponibilidade e acessibilidade, nomeadamente em termos de preço, dos produtos com um perfil nutricional saudável;<br>• Melhorar continuamente o perfil nutricional dos produtos;<br>• Identificar os produtos com informações úteis, completas e fidedignas: de leitura fácil, concisas e compreensíveis;<br>• Responder às necessidades dos consumidores com informações responsáveis e fidedignas sobre a presença de OGM, alergénios, etc.;<br>• Trabalhar em colaboração com as autoridades relevantes de modo a divulgar os programas de nutrição nacionais;<br>• Realizar campanhas para consumidores sobre valor nutricional e segurança alimentar que incentivem a adoção de padrões alimentares saudáveis;<br>• Ajudar os consumidores a preparar os produtos de uma forma mais saudável; • Desenvolver produtos para pessoas com necessidades alimentares especiais;<br>• Levar a cabo práticas de marketing responsáveis, especialmente no que diz respeito a crianças.
JERÓNIMO MARTINS	A.JM17	Garantir que pelo menos 80% das compras de produtos alimentares do Grupo Jerónimo Martins são realizadas a fornecedores locais.
JERÓNIMO MARTINS	A.JM18	Em 2022, as várias Companhias do Grupo apoiaram mais de 29 mil pessoas de contextos vulneráveis no que respeita à sua saúde e alimentação saudável. Com base na metodologia B4SI, estima-se que os apoios do Grupo (essencialmente focados em responder a emergências sociais e bem-estar social) tenham impactado cerca de 4,5 milhões de pessoas.

JERÓNIMO MARTINS	A.JM19	As lojas com protocolo estabelecido com instituições locais para o encaminhamento de géneros alimentares totalizavam 2.551 no final de 2022. Este valor representa 75% das lojas da Companhia.
JERÓNIMO MARTINS	A.JM20	Dieta Mediterrânica Há mais de 10 anos que defendemos e promovemos a Dieta Mediterrânica. Continuamos a trabalhar afincadamente quer através do sortido que oferecemos aos nossos clientes, quer através das novas receitas que são desenvolvidas pela nossa equipa de nutricionistas e pelos nossos Chefs. São já mais de 800 as receitas que promovem a Dieta Mediterrânica e que são partilhadas na revista Sabe Bem, que pode consultar aqui. Classificada como Património Mundial e Imaterial da Humanidade pela UNESCO, mais do que uma dieta, é um estilo de vida, que promove uma alimentação equilibrada e em que a mesa de refeições se torna o melhor lugar para a partilha de bons momentos com a família e com os amigos.
JERÓNIMO MARTINS	A.JM21	FUNDAÇÃO PORTUGUESA DE CARDIOLOGIA No âmbito desta parceria, o símbolo "Uma Escolha Saudável" surge em produtos de charcutaria, facilitando a identificação de produtos do Pingo Doce com baixo teor de gordura e sal. Desta forma contribui-se para uma alimentação mais equilibrada e benéfica para a saúde cardiovascular.
JERÓNIMO MARTINS	A.JM22	ASSOCIAÇÃO PORTUGUESA DE CELÍACOS O Pingo Doce colabora com a Associação Portuguesa de Celíacos para identificar os produtos de Marca Própria sem glúten. O website do Pingo Doce disponibiliza uma lista informativa sobre estes produtos.
APN	A.JM23	Dieta Mediterrânica: ciclo de webinares 2023 em parceria com Pingo Doce | APN
APN	A.JM24	Patrocínio Congresso Alimentação e Nutrição 2022 | APN
APN	A.JM25	Patrocínio Congresso Alimentação e Nutrição 2023 | APN
FMUL	A.FMUL1	O TASTY já conta com a participação entusiasta de 12 doentes que, de forma aleatória, foram alocados ao grupo de controlo ou ao grupo de intervenção. Os doentes que constituem o grupo de intervenção são convidados a seguir um padrão alimentar tipicamente mediterrânico enriquecido em alimentos fermentados, durante 12 semanas, sob acompanhamento por nutricionistas. A intervenção nutricional que implementamos no TASTY vai muito além da entrega de um plano alimentar. Passa também por promover a literacia nesta área e capacitar os doentes para melhores escolhas alimentares e melhores técnicas culinárias. Contamos com a ajuda de vários parceiros para pôr em prática este plano ambicioso, nomeadamente o Pingo Doce, que entrega aos doentes semanalmente cabazes de alimentos selecionados por nós, com a Sovena, que oferece azeite virgem extra Oliveira da Serra (não pode faltar na Dieta Mediterrânica!), com a Nestlé, que oferece os leites fermentados Kefir, e com diversas empresas da indústria das conservas (Bom Petisco, Minerva, Esip e a Cofisa) que oferecem conservas de peixe gordo (sardinha e cavala em azeite virgem extra ou em tomate).
PNPAS	A.PNPAS2	Os parceiros do PNPAS | Empresas (projetos locais): Missão Continente, Gang dos frescos LIDL, Sabe bem Pingo Doce
FCNAUP	A.FCNAUP2	A FCNAUP promove visitas de estudo a instituições ou empresas em contexto de aprendizagem, criando momentos de partilha de experiências e necessidades profissionais: Novarroz, Cozinha Central do Pingo Doce

LACTOGAL

Website	Code	CPA EXAMPLES
LACTOGAL	A.L1	Parcerias Empresariais e Económicas<br>AEP - Associação Empresarial de Portugal Câmara Hispano Portuguesa FIPA - Federação das Industrias Portuguesas Agro-Alimentares
LACTOGAL	A.L2	Mimosa Vem Lanchar" contará, ainda, com a presença de 'rostos' bem conhecidos dos portugueses como Andreia Rodrigues, Ricardo Pereira e Francisco Garcia que vão estar em direto na SIC, a partir do "Mimosa Vem Lanchar".
LACTOGAL	A.L3	Conhecimento ESB-UCP Escola Superior de Biotecnologia da Universidade Católica A Lactogal é membro do Conselho de Orientação Estratégico da Escola Superior de Biotecnologia da Universidade Católica Portuguesa COE-ESB/UCP.
LACTOGAL	A.L4	A origem da obesidade infantil resulta da combinação de uma série de factores: factores de ordem alimentar – quando uma criança consome mais calorias do que aquelas que gasta, resultando este "saldo positivo" numa alimentação hipercalórica; A origem da obesidade infantil resulta da combinação de uma série de factores: factores de ordem alimentar – quando uma criança consome mais calorias do que aquelas que gasta, resultando este "saldo positivo" numa alimentação hipercalórica; factores de ordem ambiental e cultural – quando a criança está inserida numa família que não tem hábitos de actividade física; quando mora num local onde não existem espaços para correr, saltar, trepar; quando o carro é o único transporte utilizado; ou quando a criança não é incentivada a fazer exercício físico; factores de ordem ambiental e cultural; teste; teste... factores de ordem genética – a hereditariedade também influência o balanço genético de cada pessoa. É por isso muito importante que a promoção de hábitos saudáveis se faça desde a infância.
LACTOGAL	A.L5	A Manteiga Magra Matinal tem menos 45% de gordura e de calorias, quando comparada com uma manteiga com sal, e entrega um sabor único e suave. A escolha perfeita para quem não abdica do prazer da manteiga, numa opção com menos gordura e mais fácil de barrar.
LACTOGAL	A.L6	Com uma embalagem simbólica que celebra a magia do Natal e materializa o movimento, a Mimosa abraçou o projeto de apoio e incentivo aos estudos de crianças e jovens acompanhados pela Acreditar - Associação de Pais e Amigos de Crianças com Cancro. Com o "Leite de Pai Natal", a Mimosa vai oferecer 10 bolsas de estudo a crianças e jovens acompanhados pela Acreditar.
LACTOGAL	A.L7	E porque acreditamos que os lácteos devem estar presentes na alimentação diária de todos, realizamos múltiplos apoios e donativos em produtos, essencialmente de leite e iogurtes, dirigidos a Instituições Particulares de Solidariedade e a outras instituições sem fins lucrativos, com o objetivo de suprimir necessidades alimentares de famílias e utentes carenciados.
LACTOGAL	A.L8	Desde o início da pandemia do COVID-19 e da declaração do estado de Emergência em Portugal, que a realidade operacional dos apoios aos mais carenciados se alterou. Existem mais famílias a necessitar de ajuda e o funcionamento da prestação de apoio social modificou-se. Com o encerramento, mesmo que temporário, de algumas instituições, aquelas que se mantêm em funcionamento deparam-se com um grande aumento nos pedidos que diariamente lhes chegam. Na Lactogal, cientes do nosso compromisso com a comunidade, adaptamo-nos a esta situação de forma a acompanhar as necessidades mais urgentes dos portugueses e a nova realidade nas instituições. Mesmo neste contexto difícil, intensificamos o apoio a quem mais necessita e apenas durante esta fase da pandemia e estamos a apoiar 71 instituições, entre as quais 12 hospitais. Os donativos realizados, durante o estado de emergência, foram de mais de 148,5 toneladas de produto num valor total de mais de 221 mil euros, abrangendo todo o território nacional, incluindo as regiões autónomas. A pandemia trouxe, também, necessidades extraordinárias, como, por exemplo, o apoio aos

		profissionais de saúde, sujeitos a um enorme esforço na linha da frente no combate à pandemia. Priorizarmos, também, os apoios a estas equipas e chegamos a mais de 21.500 profissionais de saúde.
LACTOGAL	A.L9	Sob o mote "Juntos vamos cuidar e ajudar as famílias e crianças da ACREDITAR", esta parceria solidária da Mimosa com a Associação de Pais e Amigos de Crianças com Cancro – ACREDITAR - irá apoiar as mais de 300 famílias residentes nas casas de acolhimento temporárias desta associação, nomeadamente no Porto, em Coimbra e em Lisboa.
LACTOGAL	A.L10	Durante o ano de 2022, a Lactogal apoiou mais de 100 beneficiários maioritariamente de apoio social, localizados em Portugal Continental e ilhas. Estes donativos em produtos, produzidos e comercializados pela Empresa, alimentam famílias e utentes carenciados. Sendo os laticínios um grupo alimentar que deve fazer parte de uma alimentação equilibrada em todas as fases da vida, a Lactogal acredita que todos devem ter estes alimentos presentes na sua alimentação diária. Durante o ano de 2022 o valor destes apoios ascenderam os 1,3 milhões de euros correspondentes a, aproximadamente, 3,6 milhões de unidades de produto.
APN	A.L11	XXXI Congresso de Alimentação e Nutrição 2022<br>SIMPÓSIO SATÉLITE > MIMOSA<br>GERAÇÃO ALPHA: NUTRIÇÃO, EDUCAÇÃO E INOVAÇÃO<br>Fernanda Cruz*, Centro de Nutrição e Alimentação Mimosa<br>Moderadora: Teresa Mariano, APN
APN	A.L12	Patrocínio Congresso Alimentação e Nutrição 2022
APN	A.L13	Patrocínio Congresso Alimentação e Nutrição 2023

LIDL

Website	Code	CPA EXAMPLES
LIDL	A.LI1	No Lidl somos fãs de um estilo de vida saudável e apoiamos vários eventos desportivos. De norte a sul de Portugal, não perca o nosso calendário de competições desportivas e participe nos seus eventos favoritos. Seguimos juntos por um estilo de vida mais saudável.
LIDL	A.LI2	Prova desportiva à vista? Temos o plano de treino ideal para si
LIDL	A.LI3	TURMA IMBATÍVEL ESTÁ DE REGRESSO ÀS ESCOLAS E DESAFIA ALUNOS A PROJETAREM O ESPAÇO DESPORTIVO IDEAL COM MAIS DE 128 MIL ALUNOS IMPACTADOS, A TURMA IMBATÍVEL ESTÁ DE REGRESSO ÀS ESCOLAS NACIONAIS
LIDL	A.LI4	Plano alimentar - Susana Francisco nutricionista
LIDL	A.LI5	A TURMA IMBATÍVEL ESTÁ DE VOLTA! Turma Imbatível está de volta às escolas do 1º Ciclo e Pré-escolar! Inscreva-se na edição 2023/2024 até ao dia 31 de janeiro. No ano letivo 2023/2024, a Turma Imbatível vai promover a atividade física junto das crianças, através dos Jogos Imbatíveis, um jogo de tabuleiro que, além de pôr todos a mexer, vai também ensinar sobre os temas da Alimentação Sustentável, Vida Saudável, Sustentabilidade e Consumo Responsável. A Turma Imbatível conta com o apoio da Direção-Geral da Educação (DGE), Direção-Geral da Saúde (DGS), Associação Natureza Portugal, em associação com a WWF (ANP | WWF), Associação Portuguesa de Nutrição (APN), Associação Nacional de Produtores de Pera Rocha (ANP), Agência Portuguesa do Ambiente (APA) e da Confederação dos Agricultores de Portugal (CAP), e tem como objetivo promover estilos de vida mais saudáveis e sustentáveis entre os mais novo.
LIDL	A.LI6	Estreia do Lidl Portugal como patrocinador premium do festival de música de Vilar de Mouros, com duas lojas "pop-up" e um Palco Lidl.
LIDL	A.LI7	Maratona do Porto, Meia maratona de Évora, São silvestre de Lisboa, São Silvestre Cidade do Porto, Lidl Swim Challenge, Meia Marotona do Porto, Triatlo de São Martinho do Porto, X Triatlo Longo de Caminha
LIDL	A.LI8	Entre 2021 e 2022, segundo o seu Relatório de Sustentabilidade: MAIS DE 20% DOS ARTIGOS DE MARCA PRÓPRIA DO LIDL CONTAVAM COM CERTIFICAÇÕES AMBIENTAIS E SOCIAIS
LIDL	A.LI9	LIDL ENTREGA 300.000 EUROS PARA AJUDAR A COMBATER POBREZA HABITACIONAL – E DESAFIA OS PORTUGUESES A FAZEREM-NO TAMBÉM, 'ARREDONDANDO' AS SUAS COMPRAS
LIDL	A.LI10	GRUPO LIDL REDUZ AS SUAS EMISSÕES OPERACIONAIS DE A.CO2 EM 60%
LIDL	A.LI11	LIDL PORTUGAL TRANSFORMA REDES DE PESCA EM 2 MIL SACOS DE FIBRAS RECICLADAS
LIDL	A.LI12	LIDL PORTUGAL ASSUME O COMPROMISSO PARA UMA GESTÃO MAIS RESPONSÁVEL DE ÁGUA NAS SUAS FRUTAS E LEGUMES
LIDL	A.LI13	PROJETO TRANSFORMAR, DO LIDL PORTUGAL, RETIRA MAIS DE 227 TONELADAS DE PLÁSTICO DAS PRAIAS E DO MAR, EM APENAS 6 ANOS
LIDL	A.LI14	APOIO À COMUNIDADE: LIDL ABRE CANDIDATURAS PARA PROJETOS INOVADORES DE APOIO À HABITAÇÃO
LIDL	A.LI15	LIDL PORTUGAL AUMENTA PARA 9,60€ O SUBSÍDIO DE ALIMENTAÇÃO DE TODOS OS SEUS COLABORADORES, JÁ EM OUTUBRO
LIDL	A.LI16	LIDL DOOU 1,9 MIL TONELADAS DE ALIMENTOS NO PRIMEIRO SEMESTRE DE 2023
LIDL	A.LI17	LIDL RETIRA PLÁSTICO DAS PRAIAS E TRANSFORMA REDES DE PESCA ABANDONADAS, EM PROL DA BIODIVERSIDADE NACIONAL
LIDL	A.LI18	A TURMA IMBATÍVEL MUDOU O MUNDO DE QUASE 27 MIL ALUNOS, TORNANDO-O MAIS SUSTENTÁVEL
LIDL	A.LI19	LIDL PROMOVE DEBATE SOBRE APOIO À PRODUÇÃO NACIONAL, COM PRODUTORES E ESPECIALISTAS, NA FEIRA NACIONAL DE AGRICULTURA
LIDL	A.LI20	LIDL CONTINUA A INVESTIR NA ELETRIFICAÇÃO DA SUA FROTA, GARANTINDO COMPROMISSO COM A SUSTENTABILIDADE
LIDL	A.LI21	LIDL TORNA-SE NO PATROCINADOR PRINCIPAL DA EQUIPA LIDL-TREK DO UCI WORLD TOUR
LIDL	A.LI22	MÃOS À SOBRA' É A NOVA WEBSÉRIE DO LIDL SOBRE DESPERDÍCIO ALIMENTAR
LIDL	A.LI23	LIDL REDUZ PREÇO NAS FARINHAS COM E SEM FERMENTO, COMBATENDO O CENÁRIO DE INFLAÇÃO
LIDL	A.LI24	LIDL COMEMORA 2º ANIVERSÁRIO DA APP LIDL PLUS COM APOIO DE 36.000 EUROS AOS BOMBEIROS
LIDL	A.LI25	LIDL ADERE À CAMPANHA DO BANCO ALIMENTAR CONTRA A FOME, REFORÇANDO O SEU APOIO A QUEM MAIS PRECISA
LIDL	A.LI26	LIDL EVITA O DESPERDÍCIO DE 7 MILHÕES DE PRODUTOS ALIMENTARES EM 2 ANOS

LIDL	A.LI27	LIDL CONTINUA A APOIAR OS PORTUGUESES E REDUZ PREÇO NO ÓLEO ALIMENTAR E DE GIRASSOL EM 14,5%
LIDL	A.LI28	LIDL JUNTA-SE MAIS UMA VEZ À CRUZ VERMELHA PORTUGUESA NA AJUDA A QUEM MAIS PRECISA
LIDL	A.LI29	LIDL PORTUGAL APOSTA EM PREÇÁRIOS DIGITAIS, PERMITINDO, POR ANO, UMA POUPANÇA DE PELO MENOS 108 TONELADAS DE PAPEL EM TODAS AS SUAS LOJAS
LIDL	A.LI30	SUSTENTABILIDADE É O FOCO DA NOVA CAMPANHA DO LIDL QUE DEMONSTRA O FÁCIL ACESSO À MESMA NAS LOJAS DA INSÍGNIA
LIDL	A.LI31	LIDL CONTINUA A INVESTIR NA MOBILIDADE VERDE EM PORTUGAL: SÃO JÁ 173 LOJAS COM POSTOS DE CARREGAMENTO ELÉTRICO
LIDL	A.LI32	EM 2022 LIDL AJUDOU A EXPORTAR 27 MIL TONELADAS DE FRUTAS E LEGUMES APOIANDO MAIS DE 500 PRODUTORES NACIONAIS
LIDL	A.LI33	DOAÇÃO CONJUNTA DAS EMPRESAS DO GRUPO SCHWARZ: UM MILHÃO DE EUROS NA AJUDA DE EMERGÊNCIA PARA A TURQUIA E A SÍRIA
LIDL	A.LI34	LIDL ASSUME NOVOS COMPROMISSOS PARA UMA ALIMENTAÇÃO CONSCIENTE
LIDL	A.LI35	LIDL DOOU 3,7 MIL TONELADAS DE ALIMENTOS EM 2022, AJUDANDO MAIS DE 330 MIL PESSOAS
LIDL	A.LI36	LIDL LIDERA RANKING DAS MARCAS DE RETALHO ALIMENTAR COM MELHOR REPUTAÇÃO EM PORTUGAL, CONSIDERADA 'MARCA DE EXCELÊNCIA'
LIDL	A.LI37	LIDL DISTINGUIDO NOVAMENTE COMO UMA DAS MELHORES EMPRESAS PARA TRABALHAR EM PORTUGAL EM 2023
LIDL	A.LI38	LIDL É A INSÍGNIA DE RETALHO MAIS PREMIADA NA ESCOLHA DO CONSUMIDOR
LIDL	A.LI39	"CALENDÁRIO DOS SONHOS" CONCRETIZA MAIS 40 DESEJOS DE COLABORADORES DO LIDL ESTE NATAL
LIDL	A.LI40	LIDL INAUGURA MAIS DUAS LOJAS A NORTE E CRIA QUASE 40 NOVOS POSTOS DE TRABALHO
LIDL	A.LI41	COM MAIS DE 128 MIL ALUNOS IMPACTADOS, A TURMA IMBATÍVEL ESTÁ DE REGRESSO ÀS ESCOLAS NACIONAIS
LIDL	A.LI42	LIDL DOA 250 MIL EUROS PARA AJUDAR PORTUGUESES EM RISCO SOCIAL, DURANTE 1 ANO
LIDL	A.LI43	LIDL MODERNIZA LOJA DE LEIRIA E CRIA 12 NOVOS POSTOS DE TRABALHO
LIDL	A.LI44	LIDL ADERE, A PARTIR DE AMANHÃ, À CAMPANHA DO BANCO ALIMENTAR CONTRA A FOME, REFORÇANDO O SEU APOIO A QUEM MAIS PRECISA
LIDL	A.LI45	LIDL INVESTE 17 MILHÕES DE EUROS NA MODERNIZAÇÃO DE MAIS 4 LOJAS, REFORÇANDO O SEU COMPROMISSO COM PORTUGAL
LIDL	A.LI47	LIDL PORTUGAL AUMENTA EM 10% O SALÁRIO A TODOS OS SEUS OPERADORES DE LOJA E ENTREPOSTO
LIDL	A.LI48	LIDL APOIA HOSPITAL FERNANDO FONSECA NA REQUALIFICAÇÃO DE ESPAÇOS PEDIÁTRICOS
LIDL	A.LI49	LIDL E BPI LANÇAM OPERAÇÃO DE FINANCIAMENTO SUSTENTÁVEL NO VALOR DE 50 MILHÕES DE EUROS
LIDL	A.LI50	LIDL INAUGURA LOJA EM LINDA-A-VELHA E CRIA 22 NOVOS POSTOS DE TRABALHO
LIDL	A.LI51	LIDL E ELECTRÃO RETIRAM MAIS DE 180 TONELADAS DE PLÁSTICO DAS PRAIAS PORTUGUESAS, EM CINCO ANOS
LIDL	A.LI52	LIDL, EM PARCERIA COM A REMAX, TRAZ DE VOLTA CONCURSO QUE PROCURA AJUDAR OS PORTUGUESES A COMPRAREM A CASA DOS SEUS SONHOS
LIDL	A.LI53	LIDL ASSINA PROTOCOLO COM A CRUZ VERMELHA PORTUGUESA, REFORÇANDO O SEU APOIO A QUEM MAIS PRECISA
LIDL	A.LI54	LIDL E A MARINHA PORTUGUESA ASSINAM PARCERIA EM PROL DA PRESERVAÇÃO DOS ECOSSISTEMAS MARINHOS
LIDL	A.LI55	LIDL JUNTA-SE À CÂMARA MUNICIPAL DE VILA DO CONDE PARA APOIAR A COMUNIDADE UCRANIANA
LIDL	A.LI56	LIDL: ESCULTURAS FEITAS DE PLÁSTICO ALERTAM PARA A POLUIÇÃO DAS PRAIAS E OCEANOS
LIDL	A.LI57	LIDL INVESTE 200 MILHÕES DE EUROS DE NORTE A SUL DO PAÍS EM 2022
LIDL	A.LI58	LIDL JUNTA-SE À CRUZ VERMELHA PORTUGUESA NA AJUDA ÀS FAMÍLIAS CARENCIADAS
LIDL	A.LI59	LIDL LIMPA PLÁSTICO DAS PRAIAS PORTUGUESAS E TRANSFORMA-O EM TÊXTEIS E ARTE
LIDL	A.LI60	LIDL MARCA PRESENÇA NA FEIRA NACIONAL DE AGRICULTURA, MOSTRANDO O SEU APOIO À PRODUÇÃO NACIONAL
LIDL	A.LI61	LIDL ANTECIPA PAGAMENTO A FORNECEDORES, REFORÇANDO O SEU APOIO AOS PRODUTORES NACIONAIS
LIDL	A.LI62	LIDL VAI DUPLICAR A AJUDA DOS PORTUGUESES AO BANCO ALIMENTAR CONTRA A FOME, A PARTIR DE AMANHÃ
LIDL	A.LI63	LIDL EVITA O DESPERDÍCIO DE 3 MILHÕES DE PRODUTOS ALIMENTARES EM APENAS 1 ANO
LIDL	A.LI64	LIDL ENTREGA 361.000 EUROS A 10 PROJETOS QUE PROCURAM COMBATER DESIGUALDADES SOCIAIS
LIDL	A.LI65	LIDL VOLTA A ESTAR AO LADO DOS PORTUGUESES E LANÇA CONCURSO COM MAIS DE 430.000 EUROS PARA AJUDAR NO ORÇAMENTO FAMILIAR MENSA
LIDL	A.LI66	AJUDA RÁPIDA PARA A UCRÂNIA: GRUPO SCHWARZ DOA BENS NO VALOR DE 10 MILHÕES DE EUROS
LIDL	A.LI67	O PROJETO TRANSFORMAR, DO LIDL E DO ELECTRÃO, RETIRA MAIS DE 110 TONELADAS DE PLÁSTICO DAS PRAIAS PORTUGUESAS EM APENAS 04 ANOS
LIDL	A.LI68	LIDL LANÇA 2ª EDIÇÃO DOS 'LIDL STARS', QUE APOIARÁ JOVENS ATLETAS NA CAMINHADA PARA OS JOGOS OLÍMPICOS DE 2024
LIDL	A.LI67	LIDL VOLTA A SER A INSÍGNIA MAIS PREMIADA NO "SABOR DO ANO", PELO QUARTO ANO CONSECUTIVO
LIDL	A.LI68	LIDL DISTINGUIDO COMO UMA DAS MELHORES EMPRESAS PARA TRABALHAR EM PORTUGAL EM 2022
LIDL	A.LI69	A 'TURMA IMBATÍVEL' DO LIDL FAZ 10 ANOS E REGRESSA ÀS ESCOLAS PARA ENSINAR, ATRAVÉS DE UM JOGO, SUSTENTABILIDADE ÀS CRIANÇAS

LIDL	A.LI70	LIDL DISTINGUIDO PELAS SUAS PRÁTICAS EM PROL DA SUSTENTABILIDADE
LIDL	A.LI71	Através do Projeto Lidl Stars pretendemos apoiar jovens promessas do desporto nacional, em diferentes modalidades. Em 2018, demos início a este projeto inovador, que reforça o nosso compromisso em promover estilos de vida saudáveis. A primeira edição, de 2018 a 2021, foi um sucesso e ditou a continuidade da iniciativa. Desta vez, serão 14 os atletas a integrar a família Lidl Stars. Praticam modalidades diferentes, do atletismo ao ciclismo, mas têm um objetivo em comum: representar Portugal nos Jogos Olímpicos de Paris, em 2024. Para os ajudar a realizar esse sonho, fornecemos-lhes uma bolsa de apoio que incide na alimentação, mas também nas propinas e material escolar, na aquisição de material desportivo e custos relacionados com competições e estágios. A todos os atletas da 2.ª edição do Lidl Stars: boa sorte!
LIDL	A.LI72	A nível nacional, vamos reforçar o nosso compromisso com a proteção climática, através da redução de, pelo menos, 70% das nossas emissões de gases com efeito de estufa, decorrentes diretamente da nossa operação (âmbito 1 e 2), até 2030, face a 2019.
LIDL	A.LI73	Ao longo dos anos, temos vindo a promover no Lidl projetos que desenvolvam de forma sustentável a economia social, com o objetivo de nos posicionarmos como um parceiro para aqueles que trabalham com as populações menos favorecidas. Alguns destes projetos foram o Movimento Mais para Todos, em parceria com a SIC Esperança, e a Promoção do Bem, que tiveram como objetivo apoiar instituições sociais e as comunidades locais onde se inserem.
LIDL	A.LI74	A nossa participação no UN Global Compact (UNGC) - a maior e mais importante iniciativa de cidadania empresarial do mundo - complementa este trabalho e fornece uma estrutura para a multiplicidade de atividades e padrões já desenvolvidos e implementados como parte de nosso compromisso com a sustentabilidade. Para nós, responsabilidade e sustentabilidade não são apenas uma promessa: estas fornecem uma base vinculativa para as nossas ações e são a chave para o futuro sucesso e crescimento do Lidl.
LIDL	A.LI75	AO SERMOS O PRIMEIRO RETALHISTA ALIMENTAR PORTUGUÊS A ASSINAR A INICIATIVA DA UN, ESTAMOS A ESTABELECER UM MARCO NA PRÁTICA PARA A IGUALDADE DE GÉNERO
LIDL	A.LI76	LIDL INVESTE 14 MILHÕES DE EUROS EM DUAS NOVAS LOJAS E CRIA QUASE 40 NOVOS POSTOS DE TRABALHO
LIDL	A.LI77	FARO: LIDL INVESTE 7 MILHÕES EM NOVA LOJA E CRIA 20 NOVOS POSTOS DE TRABALHO
LIDL	A.LI78	VAE DE CAMBRA: LIDL INVESTE 7 MILHÕES EM NOVA LOJA E CRIA 20 NOVOS POSTOS DE TRABALHO
LIDL	A.LI79	NOS ÚLTIMOS 8 ANOS, O LIDL GEROU QUASE 20 MIL MILHÕES DE EUROS PARA PORTUGAL, CONTRIBUINDO PARA UM AUMENTO MÉDIO ANUAL DE 12% NA CRIAÇÃO DE NOVOS POSTOS DE TRABALHO
LIDL	A.LI80	EXPORTAÇÕES DO LIDL PORTUGAL COM IMPACTO DE 245 MILHÕES DE EUROS NA PRODUÇÃO NACIONAL EM 2022
LIDL	A.LI81	LIDL INVESTE 110 MILHÕES DE EUROS NUM NOVO ENTREPOSTO EM LOURES, CRIANDO MAIS DE 200 NOVOS POSTOS DE TRABALHO
LIDL	A.LI82	EM 2022 LIDL AJUDOU A EXPORTAR 27 MIL TONELADAS DE FRUTAS E LEGUMES APOIANDO MAIS DE 500 PRODUTORES NACIONAIS
LIDL	A.LI83	NOS ÚLTIMOS 3 ANOS, O LIDL GEROU MAIS DE 7 MIL MILHÕES DE EUROS PARA PORTUGAL, CONTRIBUINDO PARA CRIAÇÃO E MANUTENÇÃO DE CERCA DE 60.000 POSTOS DE TRABALHO EM 2021
LIDL	A.LI84	LIDL MODERNIZA MAIS 4 DAS SUAS LOJAS, NUM INVESTIMENTO DE 20 MILHÕES DE EUROS
LIDL	A.LI85	LIDL INVESTE 200 MILHÕES DE EUROS DE NORTE A SUL DO PAÍS EM 2022
LIDL	A.LI86	EXPORTAÇÕES DO LIDL PORTUGAL REPRESENTAM 2% DAS EXPORTAÇÕES NACIONAIS DE PRODUTOS ALIMENTARES
LIDL	A.LI87	LIDL MARCA PRESENÇA NA FEIRA NACIONAL DE AGRICULTURA, MOSTRANDO O SEU APOIO À PRODUÇÃO NACIONAL
LIDL	A.LI88	LIDL ANTECIPA PAGAMENTO A FORNECEDORES, REFORÇANDO O SEU APOIO AOS PRODUTORES NACIONAIS
LIDL	A.LI89	LIDL CONTINUA A INVESTIR MAIS DE 21 MILHÕES DE EUROS DE NORTE A SUL DO PAÍS, REFORÇANDO O SEU COMPROMISSO COM PORTUGAL
LIDL	A.LI90	LIDL INICIA RECRUTAMENTO DE 150 COLABORADORES PARA AS SUAS LOJAS NA MADEIRA
LIDL	A.LI91	Assumimos o desperdício alimentar como tema prioritário para o Lidl. A nossa estratégia centra-se na sensibilização dos consumidores para o tema do desperdício alimentar e simultaneamente na doação de bens alimentares para o combate à fome. Assim nasceu o programa Realimenta, que tem o objetivo de fazer chegar a quem mais precisa os nossos produtos em condições de consumo, mas que não obedecem a todas as condições de comercialização. Aliamos assim a nossa responsabilidade social ao combate à fome.
LIDL	A.LI92	Banco Alimentar Desde 2011 a colaborar com o Banco Alimentar Contra a Fome, o Lidl já entregou mais de 328 toneladas de produtos (que representam 393.158 unidades) a esta causa, apenas em campanhas vale, com especial destaque para leite (130,43 toneladas/ 130.426 unidades), esparguete (70,27 toneladas/ 70.268 unidades), grão-de-bico (39,64 toneladas/ 39.644 unidades) e azeite (24,28 toneladas/ 32.374 unidades). Os 21 Bancos Alimentares Portugueses em atividade recolhem e distribuem várias dezenas de milhares de toneladas de produtos e apoiam ao longo do ano, cerca de 2460 instituições de solidariedade social, que entregam refeições confecionadas ou cabazes de alimentos a cerca de 373 mil pessoas.
LIDL	A.LI93	Face à atual conjuntura socioeconómica, e ciente das crescentes dificuldades sentidas pelos portugueses, estámos a ajudar a pagar as suas contas do lar, a alimentar os seus filhos, e a combater situações de isolamento, violência doméstica ou sem-abrigo. Em parceria com a Cruz Vermelha Portuguesa, apoiámos 272 famílias, num total de 538 pessoas, que se encontram ao abrigo desta instituição. Esta ajuda permite marcar a diferença nas suas vidas durante 1 ano. Num contexto particularmente difícil, como o que atravessamos, em que as assimetrias sociais são cada vez mais acentuadas, agravadas por um cenário de inflação preocupante, mantemos o compromisso com a comunidade, que já nos caracteriza: doámos 250 mil euros à Cruz Vermelha Portuguesa, permitindo à instituição apoiar, com respostas específicas, várias causas que afetam os portugueses, durante um ano, beneficiando 272 famílias carenciadas e pessoas mais vulneráveis, ou em situação de risco, num total de 538 pessoas.
LIDL	A.LI94	O Lidl contribui regularmente com donativos para algumas instituições: Liga dos Bombeiros Portugueses e Doações Entrajuda

LIDL	A.LI95	Em outubro de 2021, lançámos a 2ª edição do projeto "Da Minha Terra", com novidades relativamente às áreas de incidência abrangidas pela iniciativa, alargando a todas as categorias de produto, exceto frutas e legumes, onde já temos em Portugal um leque de fornecedores significativo, com quem estabelecemos relações duradouras e de verdadeira confiança. Assim, voltámos a desafiar os produtores nacionais a apresentarem os seus artigos, com a possibilidade de integrarem o nosso portfólio de fornecedores. Nesta edição, foram recebidas 161 candidaturas, de norte a sul do país, nas mais diversas categorias.
APN	A.LI96	Patrocínio Congresso Alimentação e Nutrição 2022
APN	A.LI98	Patrocínio Congresso Alimentação e Nutrição 2023
PNPAS	A.PNPAS2	Os parceiros do PNPAS | Empresas (projetos locais): Missão Continente, Gang dos frescos LIDL, Sabe bem Pingo Doce

MCDONALDS

Website	Code	CPA EXAMPLES
MCDONALDS	A.MC1	Promovemos o consumo de fruta e vegetais, através da implementação de diversas iniciativas. Por exemplo, através de parcerias com a Associação Portuguesa de Nutrição e com a Nutri Ventures, mediante campanhas pedagógicas que decorreram em todos os restaurantes nacionais.
MCDONALDS	A.MC2	A McDonald's tem evoluído no sentido de acompanhar as tendências da sociedade e, por isso, temos vindo a alargar permanentemente a variedade dos nossos menus. Esta oferta diversificada e equilibrada de produtos, que inclui carne (de vaca e frango), peixe, uma opção vegetariana à base de vegetais e quinoa, fruta, sopa, vegetais e cereais, engloba produtos de todos os grupos alimentares para diferentes alturas do dia (pequeno-almoço, almoço, jantar ou lanche). Para além disso, destacamos a oferta de produtos aptos para celíacos , que se encontram disponíveis em todos os nossos restaurantes, consegue satisfazer uma necessidade há muito identificada pela McDonald's e por estes consumidores.
MCDONALDS	A.MC3	A integração de frutas e vegetais demonstra a preocupação com a oferta de uma variedade que permita a adultos e crianças escolherem o que preferirem, através de diversas alternativas na combinação dos menus:
MCDONALDS	A.MC4	Outro pilar estratégico que temos trabalhado de forma concertada é a . Com a ajuda dos nossos fornecedores, da nossa oferta. nossos produtos reformulação dos temos trabalhado na redução de sal, gordura e açúcares São já vários os exemplos da melhoria do perfil nutricional cos nossos produtos: • Em relação às batatas, reduzimos em 50% o teor de sal adicionado por embalagem – de 5g sal/cesto transitámos para 2,5g sal/cesto, o que traduz um valor atual de 0,4g sal por 100g de batatas. • No caso das sopas, reduzimos anualmente e de forma gradual, 20% do teor de sal. Assim de um total de 0,5g sal por 100g de sopa, alterámos as receitas para 0,2g sal por 100g de sopa. • Nos últimos anos reduzimos, em média, 10% de sal em alguns produtos, como é o caso do Big Mac, o Hambúrguer e o Cheeseburguer, entre outros. Fizemo-lo de uma forma responsável, nunca substituindo o sal por outros aditivos ou ingredientes artificiais. • O pão conta, atualmente, com valores de sal inferiores a 1g sal por 100g de produto. • Utilizamos óleo 100% vegetal (óleos de girassol e colza) com teores máximos de ácidos gordos trans de 1,5% e ácidos gordos saturados de 12%. Orgulhamo-nos de oferecer às famílias um excelente sabor e produtos de qualidade que podem fazer parte de uma alimentação equilibrada a par de uma experiência divertida, que todos podem apreciar.
MCDONALDS	A.MC5	O nosso pão tem valores de sal inferiores a 1g sal por 100g de produto e reduzimos em 50% o teor de sal adicionado por embalagem de batatas, o que se traduz num valor atual de 0,4g sal por 100g de batatas. Um caminho nunca terminado. E esta é uma preocupação que vem de trás. Desde 2013, temos vindo a desenvolver a nível global, em conjunto com a organização sem fins lucrativos Alliance for a Healthier Generation (Healthier Generation), um plano estratégico para incentivar a disponibilização de fruta, vegetais, água e produtos com baixo teor de gordura.
MCDONALDS	A.MC6	Disponibilizar refeições equilibradas • Até final de 2022 assegurar que, pelo menos, 50% das refeições Happy Meal cumprem o seguinte requisito nutricional: - Cada refeição deve ser igual ou inferior a 600 calorias; no máximo não deve conter um valor de sódio superior a 650 mg, nem deverá conter um valor superior a 10% de calorias provenientes de açúcares adicionados. O desempenho da McDonald's Portugal • Entre 2005 e 2018, no Happy Meal já conseguimos reduzir o sal em 36%, a gordura saturada do óleo 100% vegetal em 40%, e os ácidos gordos trans em 70%.
MCDONALDS	A.MC7	Simplificar ingredientes • À medida que os gostos e as preferências dos consumidores evoluem, simplificar os ingredientes, removendo e reduzindo sabores e conservantes artificiais. O desempenho da McDonald's Portugal • 57% dos menus Happy Meal disponibilizados pela McDonald's cumprem as premissas destes objetivos. Aumentar o consumo de fruta, vegetais e água no Happy Meal • Apostar na promoção do consumo de fruta, através da implementação de diversas iniciativas, em parceria com entidades especialistas. O desempenho da McDonald's Portugal • Promovemos o consumo de fruta e vegetais, através da implementação de diversas iniciativas. Por exemplo, através de parcerias com a Associação Portuguesa de Nutrição e com a Nutri Ventures, mediante campanhas pedagógicas que decorreram em todos os restaurantes nacionais.
MCDONALDS	A.MC8	Ser transparente na disponibilização de informação nutricional • Disponibilizar informação nutricional dos produtos, de modo a promover a literacia nutricional. O desempenho da McDonald's Portugal • Disponibilizamos informação nutricional sobre os produtos desde 2006, sendo pioneiros no setor da restauração.. Comunicação responsável: Ao nível da publicidade dirigida a crianças • Garantir o cumprimento do código de compromisso da marca que autorregula a sua atuação ao nível da comunicação e marketing para crianças. O desempenho da McDonald's Portugal • Somos signatários do "Código de Autorregulação em matéria de comunicação comercial de Alimentos e Bebidas dirigida a Crianças", bem como do "EU Pledge", e apenas comunicamos combinações de Happy Meal que cumpram com as recomendações da Organização Mundial da Saúde (OMS): abaixo de 533 calorias e que incluam vegetais, água e fruta.
MCDONALDS	A.MC9	a nossa comida compromissos Happy Meal Disponibilizar refeições equilibradas, assegurando que até 2022, pelo menos 50% das refeições cumpram o "novo critério global nutricional para o Happy Meal". Simplificar ingredientes, removendo e reduzindo sabores e conservantes artificiais. Aumentar o consumo de fruta, vegetais e água no Happy Meal, através da implementação de diversas iniciativas, em parceria com entidades especialistas. Ser transparente na disponibilização da informação nutricional, de modo a promover a literacia nutricional. Comunicar de forma responsável, garantindo o cumprimento do código de compromisso da marca que autorregula a sua atuação ao nível da comunicação e marketing para crianças.
MCDONALDS	A.MC10	Fundação Infantil Ronald McDonald Os projetos da Fundação continuam a crescer, para continuarmos a manter os pais ao lado dos filhos, durante o tratamento hospitalar.
MCDONALDS	A.MC11	Campanha McSorriso. Entre 17 e 19 de novembro, a Campanha McSorriso está de volta a todos os restaurantes McDonald's em Portugal, em prol da Fundação Infantil Ronald McDonald e dos seus projetos , contando com o apoio

		de todos os clientes que podem ajudar através da compra de um McMenu. Durante este fim-de-semana, por cada McMenu ou McMenu Grande vendidos, todos os restaurantes McDonald's em Portugal contribuem com 0,25€ para a Fundação Infantil Ronald McDonald e os seus projetos. Mais do que uma "Casa longe de Casa" , a Fundação Infantil Ronald McDonald e, nomeadamente, as Casas e Espaço Ronald McDonald, permitem o apoio e o conforto das famílias, nos momentos em que as suas crianças, em tratamento hospitalar, mais precisam de as ter por perto.
MCDONALDS	A.MC12	Ajudar mais e ao longo de todo o ano. Para poder acolher mais famílias, a Fundação Infantil Ronald com a criação McDonald está a ampliar a sua rede de apoio Hospital Professor Doutor Fernando Fonseca de casa", já passaram Os consumidores podem também contribuir para Fundação emqualquer altura do ano em todos os restaurantes da McDonald's . Basta, para isso, aceder aos mais de 6000 famílias. apoiar a Quiosques Digitais em qualquer restaurante para que de forma voluntária, arredondem o valor final do pedido ou indiquem outro montante para reverter a favor desta causa. de mais projetos. É por isso que, em 2023, vai abrir as portas do Espaço Familiar Ronald McDonald, no , em Lisboa. Por estas "casas longe.
MCDONALDS	A.MC13	Estas casas pretendem ser uma "casa longe de casa" E existem mais de 4000 famílias que sabem bem o significado destas Casas Ronald McDonald e do Espaço Familiar Ronald McDonald. Todas elas acolhidas em espaços destinados aos familiares das crianças, que se deslocam da sua residência habitual para receberem tratamento no Centro Hospitalar Lisboa Central (CHLC), Hospital de Santa Maria em Lisboa ou no Centro Hospitalar S. João e/ou no IPO do Porto. gratuitamente As Casas Ronald McDonald's de Portugal, assim como o Espaço Familiar Ronald McDonald, estão integrados na rede mundial da em Ronald McDonald House Charities , espalhadas pelos cinco continentes mais de 65 países e regiões mais de . As 377 Casas Ronald McDonald e os mais de 263 Espaços Familiares Ronald McDonald acolhem anualmente mais de 3,5 milhões de crianças e suas famílias em tratamento em 90% dos hospitais pediátricos.
MCDONALDS	A.MC14	Usando a nossa continuar a investir e escala e responsabilidade contribuir para impactar , queremos positivamente a sociedade , procurando fazer a diferença em todas as comunidades locais onde estamos integrados. Cada franquiado McDonald's apadrinha, pelo menos, uma instituição local, na comunidade onde está inserido, numa verdadeira parceria de longo prazo. Contamos por isso ao longo destes anos em Portugal, com centenas de instituições apoiadas. Conscientes da forma como integramos a nossa herança de cultura global num saber fazer local, estamos confiantes no nosso compromisso em fazer de cada restaurante e de cada comunidade a nossa casa, porque cada comunidade é família McDonald's.
MCDONALDS	A.MC15	Doámos já mais de 140.000 refeições Desde o início da pandemia, em conjunto com os nossos Franquiados temos vindo a desenvolver iniciativas de apoio à comunidade. Em março e abril de 2020 oferecemos aos profissionais de saúde e equipas hospitalares que mais de 140 mil refeições se mantiveram, como até aqui, na linha da frente.
MCDONALDS	A.MC16	Em fevereiro e março de 2021, continuámos a apoiar os nossos Heróis na linha da frente do combate ao COVID-19, a Hospitais e outras Instituições como PSP, de 14 mil refeições GNR, Bombeiros, SNS 24, entre outras. Ainda, desde março de 2020 que doamos regularmente alimentos ao , para apoiar as famílias que se encontram a passar por Banco Alimentar Contra a Fome maiores dificuldades. Até agora já doámos mais de . de alimentos Estamos a desenvolver outras ações para, nesta fase de pandemia, apoiar quem mais precisa, de acordo com o panorama vivido e as necessidades sentidas em cada localidade do nosso país. 13 toneladas doando mais
MCDONALDS	A.MC17	A pandemia foi desafiante a todos os níveis, e não deixou ninguém de fora na hora de criar novos paradigmas. Famílias que outrora tinham uma vida estável, viram-se confrontadas com dificuldades até então desconhecidas, nomeadamente no que toca às suas refeições. Como reflexo, os Bancos Alimentares viram o número de beneficiários aumentar em grande escala. Consciente deste cenário, a McDonald's uniu-se à Cáritas Portuguesa juntas e decidiram criar uma ação solidária , para proporcionar bons momentos a algumas das famílias portuguesas em situação mais frágil.
MCDONALDS	A.MC18	Para assinalar o Dia Internacional da Família, celebrado a 15 de maio, a McDonald's juntouse à Cáritas Portuguesa e doou mais de 38.000 refeições a famílias apoiadas pelo programa de resposta à Covid-19 da Cáritas Portuguesa , que também apoia as famílias mais afetadas pela crise, abrangidas por programas de apoio da instituição. Desta forma, e com a missão de criar Bons Momentos em Família , abrimos as portas da nossa casa para receber cada uma destas famílias, fazendo aquilo que um deve fazer: mostrar-se disponível para ajudar sempre que necessário e bom vizinho alimentar a esperança num amanhã diferente, para melhor. Esta ação decorre de norte a sul do país e abrange mais de 3000 famílias , compostas por diversos agregados.
MCDONALDS	A.MC19	A parceria entre a McDonald's e a Casa dos Rapazes do Barreiro remonta a 2005, e desde então já integrou mais 20 utentes em estágio no restaurante do Barreiro. de O facto de terem um espaço que possam chamar de lar e um local de trabalho onde se sentem úteis, tem sido fundamental no desenvolvimento psicossocial destes jovens. O balanço destes estágios é tremendamente positivo e sai reforçado pelo testemunho do Dr. Miguel Curato, psicólogo da instituição: " todos adoraram estagiar no vosso restaurante", sendo que em alguns casos houve a formalização de um vínculo profissional de largos anos. O Dr. Miguel Curato prossegue, partilhando que o nosso restaurante " é um parceiro na busca da criação das melhores condições de vida para estes jovens, vindos de situações mais desfavoráveis.
MCDONALDS	A.MC20	Colaboramos na , ajudando a criar um ambiente acolhimento estruturado e estruturante, dando aos jovens a possibilidade de criar laços com a sua Casa. A Associação Novo Futuro gere os destinos da Casa Pinheiro e da Casa Lilás, cujas estruturas e equipamentos sofreram com o desgaste do tempo. Motivados em combater esse desgaste e dar mais vida a estas casas ; focados em criar um ambiente mais bonito, mais harmonioso e que, no final do dia, se assemelhasse ao , mobilizámos uma conforto do LAR Equipa de Voluntários para auxiliar nesta transformação .
MCDONALDS	A.MC21	Como a McDonald's distribuiu 2 milhões de livros
MCDONALDS	A.MC22	Centro de Recursos da Casa Pia de Lisboa Em 2005, demos início a uma parceria com o Centro de Recursos da Casa Pia de Lisboa, que visa pôr em prática este objetivo, . facilitando a integração de colaboradores surdos nas nossas equipas Esta parceria compreende um exigente trabalho prévio de preparação e de articulação interna e conta com o apoio de técnicos especializados, que fazem o acompanhamento desde o recrutamento à entrevista e posterior integração. O envolvimento das nossas equipas também é fundamental, na medida em que prestam formação contínua a estes colaboradores, ajudando-os na construção de uma carreira sólida. Por seu lado, o Centro de Recursos da Casa Pia de Lisboa desenvolve ações de sensibilização junto das nossas equipas, oferecendo aulas de língua gestual portuguesa como forma de facilitar a integração dos novos colegas.
MCDONALDS	A.MC23	O apoio da McDonald's Criada com o intuito de enriquecer as rotinas e desenvolver os jovens residentes na casa, a parceria entre a McDonald's e o Instituto dos Ferroviários tem ajudado a promover a melhoria de competências pessoais e sociais, encaminhando alguns jovens para os processos de seleção dos restaurantes da marca localizados na região; apoiando a sua inserção no mercado de trabalho , através da primeira oportunidade em contexto profissional; bem como doando materiais para a realização de atividades na Casa e oferecendo refeições a alguns dos residentes. Até ao dia de hoje, são vários casos de sucesso ; jovens que ainda se mantêm na instituição e outros que após concluírem os seus

		estudos, seguiram para outras áreas profissionais, com particular sucesso, inclusivamente na McDonald's. Em cada um destes casos, e independentemente dos trajetos escolhidos, a McDonald's revelou-se um apoio fundamental , tendo cimentado objetivos de vida concretos e ajudado a melhorar o bem-estar de cada um destes jovens. McDonald's, aqui para fazer bem.
MCDONALDS	A.MC24	É nacional, é McDonald's Acreditamos que o bem maior se faz pela proximidade entre os produtores e os nossos consumidores.
MCDONALDS	A.MC25	A McDonald's tem evoluído no sentido de acompanhar as tendências da sociedade e, por isso, temos vindo a alargar permanentemente a variedade dos nossos menus. Esta oferta diversificada e equilibrada de produtos, que inclui carne (de vaca e frango), peixe, uma opção vegetariana à base de vegetais e quinoa, fruta, sopa, vegetais e cereais, engloba produtos de todos os grupos alimentares para diferentes alturas do dia (pequeno-almoço, almoço, jantar ou lanche). Para além disso, destacamos a oferta de produtos aptos para celíacos , que se encontram disponíveis em todos os nossos restaurantes, consegue satisfazer uma necessidade há muito identificada pela McDonald's e por estes consumidores.
MCDONALDS	A.MC26	A integração de frutas e vegetais demonstra a preocupação com a oferta de uma variedade que permita a adultos e crianças escolherem o que preferirem, através de diversas alternativas na combinação dos menus:
MCDONALDS	A.MC27	A transparência é algo que nos define, pelo que disponibilizamos informação nutricional sobre os nossos produtos desde 2006, tendo sido pioneiros no setor da restauração. E fizemo-lo de forma inovadora, através de uma metodologia de GDA (Guideline Daily Amount, ou VDR/Valor Diário de Referência em português) que permite aos consumidores saberem, exatamente, qual a percentagem de energia, proteína, gorduras, hidratos de carbono e sal que ingeriram, em determinado momento. Hoje em dia, os nossos consumidores podem encontrar informação nutricional dos nossos produtos no website e app da marca, bem como nas embalagens e toalhetes no restaurante, de modo a promover a literacia nutricional.
MCDONALDS	A.MC28	Disponibilizamos, ainda, , website e app, para que os consumidores possam fazer escolhas pagamento Outro pilar estratégico que temos trabalhado de forma concertada é a . Com a ajuda dos nossos fornecedores, da nossa oferta. nossos produtos reformulação dos temos trabalhado na redução de sal, gordura e açúcares São já vários os exemplos da melhoria do perfil nutricional cos nossos produtos: • Em relação às batatas, reduzimos em 50% o teor de sal adicionado por embalagem – de 5g sal/cesto transitámos para 2,5g sal/cesto, o que traduz um valor atual de 0,4g sal por 100g de batatas. • No caso das sopas, reduzimos anualmente e de forma gradual, 20% do teor de sal. Assim de um total de 0,5g sal por 100g de sopa, alterámos as receitas para 0,2g sal por 100g de sopa. • Nos últimos anos reduzimos, em média, 10% de sal em alguns produtos, como é o caso do Big Mac, o Hambúrguer e o Cheeseburguer, entre outros. Fizemo-lo de uma forma responsável, nunca substituindo o sal por outros aditivos ou ingredientes artificiais. • O pão conta, atualmente, com valores de sal inferiores a 1g sal por 100g de produto. • Utilizamos óleo 100% vegetal (óleos de girassol e colza) com teores máximos de ácidos gordos trans de 1,5% e ácidos gordos saturados de 12%. Orgulhamo-nos de oferecer às famílias um excelente sabor e produtos de qualidade que podem fazer parte de uma alimentação equilibrada a par de uma experiência divertida, que todos podem apreciar.
MCDONALDS	A.MC29	Em 2020 lançámos o Programa de Bolsas de Estudo UP Umprograma nacional que atribui 200 bolsas de estudo por ano a colaboradores dos restaurantes McDonald's. Este programa existe a nível nacional desde 2020 com o objetivo de incentivar jovens colaboradores a ingressar e/ou concluir o Ensino Superior, na área que mais ambicionam. Em 2023, com o arranque da 4ª edição do Programa, o valor atribuído por Bolsa foi duplicado para , reforçando o apoio da McDonald's e 1.000€ por ano letivo Franquiados ao desenvolvimento das suas pessoas. No total, este programa já contribuiu para a formação superior de cerca de 800 jovens.
MCDONALDS	A.MC30	Projecto Upcycling . Saber mais Demos uma nova vida às fardas antigas e transformámo-las em sacos
MCDONALDS	A.MC31	Embalagens e Redução de Resíduos Estamos comprometidos em garantir 100% de embalagens provenientes de fontes renováveis, recicladas ou certificadas, até 2025.
MCDONALDS	A.MC32	Reciclagem e Economia Circular Estamos comprometidos em assegurar a reciclagem das embalagens McDonald's em 100% dos restaurantes nacionais, até 2025.
MCDONALDS	A.MC33	Redução de Emissões Estamos comprometidos em reduzir em 36% as emissões de gases de efeito de estufa em restaurantes e escritórios, até 2030.
MCDONALDS	A.MC34	Pela responsabilidade que temos, expressamos também o nosso contributo para o Desenvolvimento Sustentável e o alinhamento com a Agenda 2030. São vários os Objetivos de Desenvolvimento Sustentável para os quais contribuímos. Contudo, distinguimos estes quatro – Produção e Consumos Sustentáveis, Trabalho Digno e Crescimento Económico, Ação Climática e Redução das desigualdades - como sendo os eixos que impactamos de forma mais relevante e nos quais queremos constantemente melhorar o nosso desempenho. Este é o nosso compromisso.
MCDONALDS	A.MC35	100% segurança, colaboradores 100% formados Todos os nossos colaboradores têm uma formação intensa que inclui diferentes etapas teórico-práticas no restaurante, às quais acresce sempre um curso de manipulação de alimentos obrigatório, ministrado no primeiro dia de trabalho. Este curso contempla todas as boas práticas de manipulação, nomeadamente no que diz respeito à higiene pessoal e limpeza das instalações, equipamentos e utensílios, contaminações química, física e microbiológicas e os procedimentos para evitar contaminações cruzadas.
MCDONALDS	A.MC36	Impactamos: a variedade e melhoria da qualidade nutricional diferenciam-nos A diversidade da oferta hoje vai desde a disponibilização de hambúrgueres 100% carne de vaca, produtos de frango, peixe e uma opção vegetariana à base de vegetais e quinoa, até às sopas, saladas, frutas, gelados, pastelaria e pequeno-almoço. Este caminho foi iniciado em 1999, com o lançamento das saladas shake. Desde então, não parámos o trabalho de diversificação e melhoria contínua, procurando oferecer aos nossos clientes um conjunto de opções variadas do ponto de vista da sua composição nutricional.
MCDONALDS	A.MC37	O nosso pão tem valores de sal inferiores a 1g sal por 100g de produto e reduzimos em 50% o teor de sal adicionado por embalagem de batatas, o que se traduz num valor atual de 0,4g sal por 100g de batatas. Um caminho nunca terminado. E esta é uma preocupação que vem de trás. Desde 2013, temos vindo a desenvolver a nível global, em conjunto com a organização sem fins lucrativos Alliance for a Healthier Generation (Healthier Generation), um plano estratégico para incentivar a disponibilização de fruta, vegetais, água e produtos com baixo teor de gordura.
MCDONALDS	A.MC38	Estamos comprometidos com o melhor para as crianças. Porque a transparência é algo que define a McDonald's, temos procurado disponibilizar toda a informação nutricional relativa aos nossos produtos. Para isso, utilizamos uma metodologia que permite aos consumidores saberem a percentagem de energia, proteína, gorduras, hidratos de carbono e sal que ingerem, em determinado momento. Desta forma, cada cliente pode escolher a sua refeição com o valor nutricional mais adequado ao seu perfil e ao seu estilo de vida.
MCDONALDS	A.MC39	Investimos na procura de embalagens mais sustentáveis, na redução de resíduos e na promoção da reciclagem.

MCDONALDS	A.MC40	"O nosso compromisso em oferecer refeições de qualidade com um sabor único, proporcionando a "melhor experiência de restauração de serviço rápido", sustenta a nossa estratégia de procurar, constantemente, melhorar e inovar a nossa oferta de acordo com as expetativas dos nossos clientes. Queremos ser uma marca admirada… o que significa que queremos que os nossos clientes se sintam bem sempre que nos visitam e, para tal, têm de se sentir bem em relação à comida que servimos, mas também à nossa empresa e ao impacto que a nossa atividade tem nas comunidades onde estamos. E mantemo-nos fiéis à nossa convicção de que somos um dos restaurantes favoritos das famílias e que, por isso, devemos retribuir à comunidade parte do que a mesma nos dá, para ter sucesso a longo prazo. É uma convicção que consagra os valores do nosso fundador, Ray Kroc, e a forma como a McDonald's aguarda, com expetativa, os próximos anos." o futuro Inês Lima, Diretora-Geral da McDonald's Portugal
MCDONALDS	A.MC41	Sabia que... em 2006, a McDonald's foi pioneira no setor da restauração, ao facultar informação nutricional dos seus produtos, de modo a permitir uma tomada de decisão mais informada pelos consumidores?
MCDONALDS	A.MC42	Na McDonald's, queremos fazer-te chegar as nossas práticas de sustentabilidade ambiental. Como tal, assumimos esse compromisso através de um projeto de Upcycling , que garante uma nova vida às fardas usadas pelos colaboradores.
MCDONALDS	A.MC43	Estamos comprometidos em garantir que 100% das embalagens provenientes de fontes renovaveis ou certificadas até 2025.
MCDONALDS	A.MC44	Estamos comprometidos em reduzir em 36% as emissões de gases de efeito de estufa em Restaurantes e escritórios e em 31% a intensidade de emissões em toda a cadeia de distribuição, até 2030.
MCDONALDS	A.MC45	A mobilidade verde é outra aposta da McDonald's Portugal para reduzir emissões para a atmosfera. Desde 2017, temos vindo a instalar pontos de carregamento elétrico em alguns restaurantes McDonald's! Com a criação desta rede de carregadores de veículos elétricos e híbridos em vários pontos do país, a McDonald's pretende promover esta forma de mobilidade junto da comunidade, encorajando e facilitando a adoção de comportamentos mais sustentáveis . A disponibilização deste novo serviço irá também proporcionar uma experiência mais simples, completa e rápida na visita a um destes restaurantes McDrive, em Portugal.

MERCADONA

Website	Code	CPA EXAMPLES
MERCADONA	A.M1	Em 2023, a Mercadona continuou a reforçar os seus vínculos com as associações de consumidores, consciente da relevância da tarefa que estas realizam e do papel de interlocução que desempenham em representação dos "Chefes".
MERCADONA	A.M2	Em Portugal, tem colaborado com as associações de consumidores, DECO e DECO PROTeste, e com uma associação de intolerâncias e alergias, a Associação Portuguesa de Celíacos (APC), ao mesmo tempo que tem continuado a reforçar a sua relação com os consumidores em Bruxelas, neste caso através da colaboração com uma associação de consumidores e quatro entidades institucionai
MERCADONA	A.M3	Mercadona adere à iniciativa "Portugal Sou Eu" e marca presença na Feira Nacional de Agricultura
MERCADONA	A.M4	Em 2023, a Mercadona reuniu com associações de consumidores, associações de intolerantes e alérgicos e Administrações públicas para lhes explicar a sua Estratégia Saudável
MERCADONA	A.M5	Sob o conceito de "a sua cesta equilibrada" tem mantido, através da sua página web e dos diversos canais que tem nas redes sociais, uma comunicação ativa com os clientes, através da divulgação de receitas simples que podem incorporar no seu dia a dia, propostas de menus semanais, recomendação de produtos concretos ou, ainda, partilhando dicas culinárias e alimentares, consciente do quão importante é saber o que precisamos de comer, como podemos cozinhá-lo, em que quantidade e com que frequência.
MERCADONA	A.M6	Dentro da sua estratégia Saudável, a Mercadona e os fornecedores com que colabora têm continuado a melhorar os ingredientes e a reduzir ou substituir aqueles que não acrescentam valor. É o caso do fiambre do fornecedor Casa Tarradellas com 90 % de carne, ou dos batidos de fruta e leite Caribe, fabricados pelo fornecedor García Carrión, que incorporam maior quantidade de fruta e também reduziram 1,4 gramas de açúcar adicionado. Do mesmo modo, foram acrescentadas mais referências de gelados com formato mini, fabricados pelo especialista Helados Estiu, com o objetivo de definir um tamanho de porção adequado à necessidade dos clientes. Também foram introduzidos novos produtos adequados à preparação em eletrodomésticos como a fritadeira de ar quente, por exemplo, os bolinhos de bacalhau do fornecedor Audens Foods, à venda em Espanha, ou os douradinhos de pescada sem glúten e lulas panadas, ambos fabricados pela Congalsa. Todo este esforço permitiu-lhe contar com um sortido variado, eficiente e de qualidade, que permite ao "Chefe" dispor de uma ampla gama de produtos, tanto frescos como não perecíveis. Além disso, graças a estes esforços, reforçou a sua relação com os clientes, conhecendo melhor as suas necessidades para, desta forma, poder oferecer-lhes as melhores opções para uma dieta saudável.
MERCADONA	A.M7	Doações a bancos alimentares, cantinas sociais e outras entidades. - Colaboração em campanhas de captação de recursos a favor de entidades sociais reconhecidas, disponibilizando aos "Chefes" a possibilidade de realizar doações em caixa. - Cartão Sociedade. - Doações a bancos alimentares, cantinas sociais e outras entidades. - Cartão Sociedade. - Gestão do stock nas lojas para reduzir o desperdício alimentar.
MERCADONA	A.M8	A Mercadona é signatária do Pacto Mundial da Organização das Nações Unidas desde 2011 e trabalha para contribuir para os Objetivos de Desenvolvimento Sustentável (ODS) que têm impacto na sua atividade. No caso da pesca, e consciente de como as alterações climáticas afetam a biodiversidade e os oceanos, promove há vários anos a sustentabilidade tanto no âmbito da pesca extrativa como no da aquicultura, o que se traduz numa colaboração estreita e direta com os fornecedores e interfornecedores especia-
MERCADONA	A.M9	Desde 2011, a Mercadona é signatária, em Espanha, do Pacto Mundial das Nações Unidas, fórum que conta com a participação de 24.257 entidades de 167 países diferentes. Com a sua adesão, a empresa, que é reconhecida como Sócio Prescritor Bronze, compromete-se a defender os valores fundamentais em matéria de direitos humanos, normas laborais e luta contra a corrupção. Este fórum também conta com 62 redes locais, entre as quais a do Pacto Mundial da ONU em Espanha, apoiadas por 1.673 entidades. Durante estes doze meses, a Mercadona, ao abrigo do Pacto Mundial das Nações Unidas, continuou a trabalhar também para avançar como empresa na procura de soluções e respostas às necessidades estabelecidas pelos Objetivos de Desenvolvimento Sustentável (ODS)
MERCADONA	A.M10	A Mercadona continuou a consolidar o seu compromisso com os grupos mais vulneráveis através de diferentes iniciativas solidárias com um importante impacto social, como demonstra a inclusão, em 2023, de 215 novas entidades sociais com as quais a empresa começou a colaborar.
MERCADONA	A.M11	Mercadona participa na campanha do Banco Alimentar Contra a Fome
MERCADONA	A.M12	Mercadona participa na campanha do Banco Solidário Animal
MERCADONA	A.M13	Mercadona doa 600 toneladas de alimentos no primeiro semestre de 2023

MERCADONA	A.M14	Mercadona participa na campanha solidária da Cruz Vermelha Portuguesa
MERCADONA	A.M15	Mercadona doa 1.900 toneladas de bens de primeira necessidade em 2022
MERCADONA	A.M16	Comité de direção da Mercadona aprova aumento de salários dos colaboradores de acordo com a inWação
MERCADONA	A.M17	Mercadona reduziu a sua pegada de carbono em 38%
MERCADONA	A.M18	Mercadona aumenta 11% salário de entrada em Portugal a partir de janeiro
MERCADONA	A.M19	Comité de direção da Mercadona aprova aumento de salários dos colaboradores de acordo com a inWação
MERCADONA	A.M20	Mercadona continua a apostar na produção nacional e compra 500 milhões de euros a 900 fornecedores portugueses
MERCADONA	A.M21	Mercadona e Pasto Alentejano juntos na produção de borrego nacional
MERCADONA	A.M22	O Comité de Direção da Mercadona deTniu a subida de 2,7% no salário da equipa
MERCADONA	A.M23	Mercadona completa um ano da sua Estratégia 6.25 para a redução do plástico
MERCADONA	A.M24	AMI e Mercadona assinam acordo de colaboração
MERCADONA	A.M25	Cruz Vermelha Portuguesa e Mercadona assinam acordo de colaboração
MERCADONA	A.M26	Mercadona reduz o consumo energético em 39,7 milhões de kWh em 2019 e 2020
MERCADONA	A.M27	Cáritas Portuguesa e Mercadona assinam acordo de colaboração
MERCADONA	A.M28	Mercadona e CAP assinam protocolo único de colaboração
MERCADONA	A.M29	A Mercadona incorpora plástico reciclado para melhorar as embalagens das suas pizzas refrigeradas
MERCADONA	A.M30	Mercadona aposta no morango nacional
MERCADONA	A.M31	Mercadona reforça doação de produtos de primeira necessidade ao Banco Alimentar Contra a Fome de Aveiro
MERCADONA	A.M32	Mercadona doa mais de 17.000 toneladas de alimentos em 2020 a instituições sociais
MERCADONA	A.M33	Mercadona assina “Pacto Contra a Violência” e mantém compromisso pela igualdade
MERCADONA	A.M34	Mercadona assina acordo com Banco Alimentar de Viana do Castelo
MERCADONA	A.M35	Mercadona realiza doação especial de Natal com 80.000 quilos de bens alimentares
MERCADONA	A.M36	Mercadona acelera a sua estratégia para reduzir o plástico com investimento de mais de 140 milhões de euros
MERCADONA	A.M37	Colaborações A Mercadona trabalha em colaboração com numerosas organizações nacionais e internacionais para partilhar boas práticas, aprender uns com os outros e conseguir avanços na sustentabilidade, tendo sempre em conta a necessária visão da cadeia.
MERCADONA	A.M38	Para proporcionar aos seus “Chefes” a possibilidade de elaborar uma dieta equilibrada, além da variedade do seu sortido, dos conselhos, das receitas e da própria prescrição dos colaboradores, a empresa introduziu melhorias na rotulagem que contribuem para esse mesmo fim. Assim, por exemplo, durante estes meses reformulou a rotulagem de inúmeros produtos, aplicando critérios que facilitam a sua leitura, como é o caso do aumento do tamanho da letra ou a decisão de uniformizar as tabelas nutricionais, facilitando aos “Chefes” a escolha do produto que melhor se adequa às suas necessidades. Do mesmo modo, continuou a trabalhar na melhoria dos elementos que informam ou formam os clientes sobre cada produto, não só dos ingredientes ou da informação nutricional, como também, por exemplo, de outros como a forma de preparação ou o modo de conservação.
MERCADONA	A.M39	Todos estes movimentos, que facilitam as decisões para melhorar os hábitos alimentares dos “Chefes” da Mercadona, enquadram-se também na sua política de transparência total que, há alguns anos, promove nos seus packagings. Uma aposta numa rotulagem transparente que, no caso dos produtos elaborados sob a égide
MERCADONA	A.M40	(....)as suas marcas próprias (Hacendado, Bosque Verde, Deliplus e Compy), conta com um método próprio, de acordo com o qual, além de identificar o fornecedor fabricante, é oferecida ao “Chefe” na rotulagem toda a informação relevante sobre cada produto, uma informação objetiva e de fácil acesso, para que cada cliente possa escolher, conforme os seus hábitos, a melhor forma de obter a sua cesta equilibrada(...)
MERCADONA	A.M41	Há vários anos que a Mercadona mantém um compromisso responsável com os “Chefes” que sofrem de intolerâncias ou alergias alimentares, e trabalha para incluir nas prateleiras produtos adequados para as suas dietas que, além disso, sejam facilmente identificáveis através da rotulagem, aspeto que foi melhorado com o aumento da letra. Desta forma, e como exemplo, em 2023 continuou a aumentar o sortido sem glúten, tendo já um total de 1.533 produtos livres desta proteína. Neste sentido, foram acrescentadas referências como os douradinhos de pescada sem glúten do fornecedor Congalsa. Ao mesmo tempo, mantém relações em Espanha e Portugal com 29 associações de intolerantes e alérgicos com as quais, com as quais, através de um diálogo constante, realiza ações para ajudar a sociedade a conhecer as diferentes necessidades alimentares dos diferentes grupos. Marcas que a Mercadona desenvolve desde 1996, cujos fabricantes estão claramente identificados na rotulagem A empresa continuou também a trabalhar para satisfazer as necessidades específicas de nutrição ou alimentação de vários grupos de “Chefes”, como as pessoas que optam por uma dieta vegana, para as quais atualmente o sortido conta com novas referências, como a bebida de aveia sabor chocolate, elaborada pelo fornecedor Liquats Vegetals, ou a bebida de aveia 0 %, fabricada pela Frías e que, além disso, não tem açúcares nem edulcorantes.

NESTLÉ

Website	Code	CPA EXAMPLES
NESTLÉ	A.N1	ALDI, AUCHAN, DANONE, DECO PROTESTE e NESTLÉ unidos na defesa do rótulo nutricional NutriScore Página de Facebook “Mais Nutri-Score“ para informar os consumidores portugueses Voltar aos Comunicados de Imprensa ALDI, AUCHAN, DANONE, DECO PROTESTE e NESTLÉ uniram-se em prol da alimentação saudável e do apoio ao Nutri-Score. Para isso, criaram a página de Facebook “Mais Nutri-Score”, cujo objetivo é divulgar o Nutri-Score, sistema de informação nutricional que facilita a comparação de produtos alimentares, dentro da mesma categoria, contribuindo dessa forma para melhores escolhas alimentares. As 5 entidades estão empenhadas em que Portugal adote também o Nutri-Score nas embalagens dos produtos tal como já acontece em 7 outros países da Europa. O rótulo nutricional consiste num código de cores e de letras, colocado na frente das embalagens, que ajuda os consumidores a fazerem escolhas alimentares mais saudáveis, baseadas nos níveis de classificação que vão de A - Verde Escuro a E - Vermelho. A ALDI Portugal iniciou o processo de aplicação do Nutri-Score nas suas marcas próprias em abril de 2021. O Nutri-Score já foi introduzido em outros países onde o Grupo ALDI Nord está presente. Em Portugal, a introdução será gradual e terá início em marcas próprias de categorias relevantes: All Seasons, Alfacinha, Golden Bridge, King's Crown e Rio d'Oro. é o nome da página na qual a ALDI disponibiliza aos seus clientes “O Nutri-Score explicado de forma simples” as informações mais importantes sobre o Nutri-Score e sobre o processo de implementação do mesmo. Por seu turno, o folheto da campanha nas lojas AUCHAN, inserida na Feira de Vida Saudável (que decorreu em maio), dedicou 2 páginas ao tema Nutri-Score com visibilidade para produtos com este sistema de informação nutricional. Foi,

		igualmente, distribuído aos clientes que efetuaram compras online e nas parafarmácias da AUCHAN, um folheto explicativo do sistema Nutri-Score e de divulgação da campanha da DECO PROTESTE. Quanto à DANONE Portugal, que deu início à colocação do Nutri-Score nas embalagens dos seus produtos no início de 2020, tem na atualidade o Nutri-Score implementado em praticamente todo o seu portefólio, onde se destacam marcas como Activia, Actimel, Danacol, Danone, Danonino e YoPRO. A DECO PROTESTE lançou uma carta aberta a favor da adoção do Nutri-Score como esquema de informação nutricional oficial em Portugal: Nutri-Score: exija o rótulo que fala para si ! A NESTLÉ Portugal tem vindo a implementar progressivamente o sistema Nutri-Score nos seus produtos desde o início de 2020. De momento, já existe em produtos de todas as suas categorias e marcas abrangidas, como FITNESS, ESTRELITAS, CHOCAPIC, CHEEERIOS, NAT, NESTUM, NESQUIK, NESCAFÉ, GARDEN GOURMET e SMARTIES. Nutri- A Nestlé é ainda a primeira empresa em Portugal a disponibilizar a informação nutricional nos rótulos dos seus produtos - já disponível nas embalagens de NESTUM Mel Score em Braille e de NESTUM Chocolate. Nos meses de junho e julho, promoveu também uma campanha digital, alinhada com outros mercados europeus onde a Nestlé está presente e que já adotaram o Nutri-Score nos rótulos dos seus produtos.
NESTLÉ	A.N2	NESQUIK® 0% é parceiro da Associação Portuguesa de Nutrição
NESTLÉ	A.N3	Nestlé Professional e AHRESP assinam protocolo de colaboração
NESTLÉ	A.N4	Nestlé Portugal e NOVA Medical School voltam a estar juntas na formação dos futuros nutricionistas II Edição do Projeto "Cereals for All" Voltar aos Comunicados de Imprensa Depois do sucesso da primeira edição de "Cereals for All – Desenvolver o Futuro dos Cereais", a Nestlé volta a desafiar os alunos finalistas da Licenciatura em Ciências da Nutrição da NOVA Medical School a envolverem-se no desenvolvimento de produtos alimentares inovadores. Esta colaboração pioneira entre a Indústria Alimentar e a Academia visa promover a imersão dos alunos no processo de desenvolvimento de produtos à base de cereais, saudáveis e sustentáveis, nutricionalmente adaptados ao seu público-alvo. Decorreu, hoje, o evento de apresentação e de assinatura protocolar entre a Nestlé Portugal e a NOVA Medical School que deu início à segunda edição do Projeto "Cereals for All – Desenvolver o Futuro dos Cereais" – uma parceria inovadora entre a indústria alimentar e o meio académico. O projeto faz parte da unidade curricular de estágio e envolve todos os alunos finalistas da Licenciatura em Ciências da Nutrição, no processo de desenvolvimento de produtos alimentares. Durante a apresentação, foi feito o balanço da primeira edição, com a participação e testemunho de um aluno como porta-voz da turma anterior. O evento teve lugar no edifício da NOVA Medical School, em Lisboa, e terminou com a assinatura do protocolo por ambas as entidades. Nesta segunda edição, a parceria entre a Nestlé Portugal e a NOVA Medical School pretende continuar a proporcionar aos alunos o contacto com o processo de desenvolvimento de novos produtos alimentares, saudáveis e sustentáveis, à base de cereais. O Projeto inclui várias etapas, a começar pelo próprio evento de lançamento da II Edição, seguindo-se uma visita à Fábrica da Nestlé em Avanca, com os alunos participantes. Durante o seu trabalho os alunos serão inspirados em duas power sessions de partilha de conhecimento das várias etapas de um processo de desenvolvimento de um produto alimentar. Ao longo do Projeto, os alunos têm a oportunidade de aliar os conhecimentos adquiridos durante os seus estudos ao processo criativo e tecnológico de inovação em produtos alimentares, através da mentoria dada pelas equipas técnicas da Nestlé Portugal. "Estamos entusiasmados por anunciar o lançamento da segunda edição do projeto 'Cereals for All'. O sucesso do projeto do ano passado foi evidente e ficou patente no interesse e na dedicação dos estudantes que nos apresentaram conceitos inovadores e 'out of the box' ao longo do processo de desenvolvimento dos produtos", afirma Ana Leonor Perdigão, Nutrition, Health and Wellness Manager da Nestlé Portugal. "Esta parceria única entre a Nestlé e a NOVA Medical School continua a alargar as fronteiras da inovação na indústria alimentar, e esperamos ansiosamente ver os novos produtos e ideias que sairão desta segunda edição.", concluiu. Por seu lado, Conceição Calhau, responsável pela Licenciatura em Ciências da Nutrição, da NOVA Medical School, afirma: "A parceria com a Nestlé permite à NOVA Medical School contribuir com a sua experiência em nutrição para o desenvolvimento de produtos mais saudáveis e inovadores, enquanto apresenta uma oportunidade de aprendizagem única aos seus alunos de Ciências da Nutrição, podendo aplicar a sua experiência teórica num ambiente prático de competências adquiridas durante os 4 anos de curso e aquisição de competências nas áreas do negócio, empreendedorismo, gestão de prioridades e pressão empresarial, muito importante para preparação para o mercado de trabalho de hoje. Estamos entusiasmados para ver os resultados deste segundo ano." O Projeto "Cereals for All" é único em Portugal e, em 2022, reuniu 14 alunos finalistas da Licenciatura em Ciências da Nutrição e várias equipas de desenvolvimento de produtos da Nestlé, com o objetivo de reforçar as competências dos alunos na inovação de produtos alimentares saudáveis e sustentáveis, à base de cereais, nutricionalmente adaptados a diferentes perfis de consumidores. A primeira edição do programa resultou em cinco projetos nas áreas de Saúde Infantil, Conveniência, Saúde Mental, Saúde da Mulher e Envelhecimento Ativo.
NESTLÉ	A.N5	Nestlé Portugal e NOVA Medical School voltam a estar juntas na formação dos futuros nutricionistas
NESTLÉ	A.N6	A Nestlé e a Nova SBE lançaram em 2022 a 4ª edição do programa de Open Innovation START and CO. É tempo de conhecermos os três projetos finalistas: 10pple, Little One e Shoppermotion foram as startups selecionadas para colaborar com a Nestlé, em Portugal e em Espanha.
NESTLÉ	A.N7	A Nestlé Health Science organizou, em maio de 2022, o CANuG - Curso Anual de Nutrição em Geriatria. O curso foi realizado em parceria com a Associação Portuguesa de Nutrição Entérica e Parantérica (APNEP), a Sociedade Portuguesa de Medicina Interna (SPMI) e o Núcleo de Estudos de Nutrição Clínica (NENC) da SPMI. A iniciativa contou com 130 especialistas, entre eles Médicos de Medicina Interna, Médicos de Medicina Geral e Familiar, Jovens Recém Especialistas e Nutricionistas. O principal objetivo foi a partilha de experiências profissionais e contextos hospitalares distintos a nível nacional em temas como a identificação de doentes desnutridos, a abordagem ao rastreio nutricional ou a intervenção nutricional do doente.
NESTLÉ	A.N8	Nestlé regressa com os estágios de verão para estudantes universitários
NESTLÉ	A.N10	O Programa em Portugal O Programa Nestlé por Crianças mais Saudáveis iniciou-se em 1999, em parceria com a Direção-Geral da Educação do Ministério da Educação e ao longo deste tempo tem chegado a milhões de crianças em todo o país. Este programa implementa-se através das escolas mas também com ações diretas às famílias e à comunidade. O Programa Nestlé por Crianças mais Saudáveis tem como foco promover uma alimentação mais saudável e sustentável como parte de um estilo de vida promotor de saúde. O Programa procura dinamizar ações práticas e lúdicas, mantendo um caráter didático, formativo e informativo. As suas iniciativas são estruturadas num clima de respeito pela diversidade, sustentabilidade e equidade, para uma cidadania participada, responsável e inclusiva
NESTLÉ	A.N11	Alguns dias depois, a 18 de outubro decorreu a Fitness Pink Ribbon Talk, uma conversa intimista sobre o tema, em parceria com a Rádio Renascença, que juntou a atriz Carla Andrino, a Presidente do iMM Maria Mota e a atleta Patrícia Mamona. "Esta campanha pretende reforçar a ideia de que isto se trata de uma luta comum, de todas as mulheres.

		Queremos ajudar a combater, investigar e, acima de tudo, alertar para a importância do diagnóstico precoce do cancro da mama", acrescentou ainda Maria João Coentro.
NESTLÉ	A.N12	"CREATING THE FUTURE OF CEREALS" Nestlé e NOVA Medical School unidas para desenvolver produtos inovadores
NESTLÉ	A.N13	Juntar conhecimento científico na área da indústria alimentar aos desafios das empresas é um dos segredos para o sucesso. O projeto "Cereals for All – Creating the Future of Cereals" é um dos exemplos de uma iniciativa que juntou a Nestlé à NOVA Medical School com vista a desenvolver produtos inovadores na área da saúde e sustentabilidade.
NESTLÉ	A.N14	Cereals for All – Creating the Future of Cereals" é um projeto pioneiro em Portugal e juntou 14 alunos finalistas da licenciatura em Ciências da Nutrição da Nova Medical School (NMS) a diversas equipas de desenvolvimento de produto da Nestlé. Ao longo de três meses, entre março e maio de 2022, trabalharam em conjunto, no desenvolvimento de produtos à base de cereais, saudáveis e sustentáveis desde a sua origem. Desta sinergia resultaram cinco projetos nas áreas de Saúde Infantil, Conveniência, Saúde Mental, Saúde da Mulher e Envelhecimento Ativo.
NESTLÉ	A.N15	5 PROJETOS Desta sinergia resultaram cinco projetos nas áreas de Saúde Infantil, Conveniência, Saúde Mental, Saúde da Mulher e Envelhecimento Ativo.
NESTLÉ	A.N16	Esta parceria entre a Nestlé Portugal e a NOVA Medical School tem por base um protocolo de colaboração com vista à inovação em produtos alimentares que se distingam pelo seu caráter healthy and sustainable by design e que sejam nutricionalmente adequados para a população-alvo à qual se destinam. A inovação é a palavra chave nesta iniciativa, como refere Andreia Vaz, Innovation Beyond the Core & Digital Transformation Iberian Director na Nestlé. "O encontro entre a NOVA Medical School e a Nestlé traz uma possibilidade transformadora na criação de novos produtos na área dos cereais, um segmento muito importante para o nosso negócio". E continua: "Com esta parceria, pretendemos trazer uma perspetiva fresca para a criação de novos conceitos, pretendemos envolver a geração de nutricionistas do futuro neste processo e dar-lhes poder. Mas queremos também aprender sobre o conhecimento científico mais recente na criação de produtos à base de cereais, permitindo que sejam reconhecidos pela comunidade científica como saudáveis e sustentáveis desde a origem", conclui.
NESTLÉ	A.N17	E claro que também a Academia tira frutos de uma parceria deste género. "É muito importante e vai ao encontro da estratégia da NOVA Medical School que aposta num ensino baseado na aquisição de competências práticas, no raciocínio clínico e na evidência científica, colocando, sempre, o conhecimento ao serviço da comunidade", refere Helena Canhão, Diretora da NOVA Medical School.
NESTLÉ	A.N18	PARCERIA ENTRE NESTLÉ E FACULDADE DE CIÊNCIAS E TECNOLOGIA Investigar e inovar em sustentabilidade
NESTLÉ	A.N19	Nestlé e NOVA Medical School juntas no desenvolvimento de produtos alimentares inovadores
NESTLÉ	A.N20	Nestlé lança em Portugal os primeiros produtos com NutriScore Novo sistema de rotulagem ajuda nas escolhas mais saudáveis Voltar aos Comunicados de Imprensa A Nestlé está a iniciar em Portugal a implementação do Nutri-Score nas embalagens dos seus produtos. Este novo rótulo nutricional consiste num código de cores e de letras, colocado na frente das embalagens, que ajuda os consumidores a fazerem escolhas alimentares mais saudáveis, baseadas nos níveis de classificação que vão de A-Verde: o mais saudável, a EVermelho: o menos saudável. As primeiras embalagens de produtos Nestlé a incluírem este novo sistema de informação no nosso país, e que já estão no mercado, são os cereais de pequeno-almoço FITNESS Original e NESQUIK Alphabet, ambos classificados com A-Verde. A estes seguir-se-á a gama de produtos flexitarianos Garden Gourmet. Este sistema, já implementado em diversos países europeus e que agora é também adotado pela Nestlé em Portugal, é considerado como o mais eficiente na informação rápida ao consumidor sobre as características e os benefícios de um determinado produto. Em vários Estados-Membro da União Europeia (EU), grupos de consumidores, legisladores, académicos, empresas de alimentação e retalhistas estão a pedir que o rótulo nutricional Nutri-Score se torne obrigatório na UE. "Dar aos consumidores as melhores ferramentas de informação para poderem fazer escolhas mais acertadas para si e para as suas famílias é um dos compromissos da Nestlé. O Nutri-Score oferece agora uma solução clara que ajuda o consumidor a decidir mais rápido, Para além disso, o Nutri-Score motiva as empresas de alimentos e bebidas a acelerar o desenvolvimento de produtos nutricionalmente mais equilibrados. O Nutri-Score não deve apenas ajudar a Nestlé a elevar a fasquia; convidamos toda a indústria a juntar-se a nós.", afirma Paolo Fagnoni, Diretorgeral da Nestlé Portugal. Com um em cada dois adultos europeus com sobrepeso ou obesidade - aumentando a probabilidade de doenças cardíacas, diabetes e cancro - é claramente necessária uma ação mais forte para ajudar a conter esta crise de saúde pública. A rotulagem nutricional foi identificada pelos governos nacionais e por especialistas internacionais em saúde como uma das principais ferramentas políticas que podem ser usadas para apoiar os consumidores a fazer escolhas alimentares saudáveis e informadas num piscar de olhos. Pesquisas realizadas em vários países mostram que o Nutri-Score é atualmente o esquema com melhor desempenho, na medida em que ajuda os consumidores a compararem a qualidade nutricional dos alimentos numa gama de produtos e a fazer escolhas mais saudáveis no supermercado.
NESTLÉ	A.N21	De forma a oferecer aos nossos consumidores a melhor orientação possível nas embalagens de produtos alimentares e indo ao encontro do nosso propósito de ajudar as pessoas a levar vidas mais saudáveis, já desde junho de 2019 que defendemos o uso do Nutri-Score em toda a Europa como sistema interpretativo de informação nutricional de produtos alimentares e bebidas. Em Portugal, segundo o estudo "Atitudes dos consumidores portugueses face à rotulagem alimentar" (IPAM, FCNAUP), 59% dos portugueses afirma ler regularmente os rótulos dos alimentos, mas 40% admite não compreender a informação aí constante para fazer escolhas mais saudáveis. A Nestlé apoia a implementação obrigatória do Nutri-Score na Europa: Atualmente, o Nutri-Score só é aplicado a título voluntário. Mas queremos mudar isso. Estamos empenhados em tornar o Nutri-Score no sistema de avaliação nutricional padrão da União Europeia e obrigatório na frente das embalagens. Para isso juntámo-nos a mais de 30 investigadores, deputados e organizações de defesa dos consumidores, assim como outras empresas, como a DANONE, a AUCHAN e o LIDL. Numa à comissária da União Europeia, Stella Kyriakides. carta redigida em conjunto , lançámos o nosso desafio a 28 de abril de 2020
NESTLÉ	A.N22	ALIMENTAR O CONHECIMENTO É SAUDÁVEL. Início Bem-Estar Nutrição SEGUE AS NOSSAS DICAS Escolhe a categoria que mais te interessa e aprofunda o teu conhecimento sobre vários temas. NUTRIÇÃO FLEXITARIANISMO. O QUE É? Segundo a Organização Mundial da Saúde (OMS), o flexitarianismo pode ser definido como um padrão alimentar de base vegetal (plant-based) que inclui igualmente alimentos de origem animal.
NESTLÉ	A.N23	Como podemos contribuir para um mundo mais sustentável através de pequenas alterações na nossa alimentação? Reunimos oito dicas práticas inspiradas por várias fontes para o ajudar nesta jornada: 1 1. Ingerir as porções diárias de fruta e hortícolas recomendadas. 2. Aumentar o consumo diário de leguminosas e dar preferência aos cereais integrais. Já experimentou os cereais integrais Nutri-Score A! NESTLÉ FITNESS Original ? A melhor parte é que são 3. Optar por produtos da época e de maior proximidade. 4. Incluir mais alimentos e bebidas vegetais na alimentação diária. Faça

		o teste com os seus amigos! Experimente um churrasco com o nosso de origem vegetal de diferença? Sensational Burger GARDEN GOURMET , com Nutri-Score A. Será que alguém dá pela 5. Opte por peixe e marisco de origem sustentável ou alternativas vegetais a estes alimentos. Já ouviu falar no VUNA ? Sim, leu bem! É uma das nossas mais recentes inovações, com Nutri-Score B, capaz de dar aquele toque delicioso à sua salada, wrap ou poke bowl. E está disponível nos supermercados em Portugal! 6. Evite desperdiçar comida. Faça a sua lista de compras consoante o planeamento semanal de refeições e use a criatividade na cozinha para aproveitar sobras. 7. Procure embalagens recicláveis, evite embalagens desnecessárias e recicle-as nos contentores adequados. 8. Aumente gradualmente o número de refeições semanais à base de proteína vegetal. Temos produtos e receitas incríveis que lhe vão servir de ponto de partida:
NESTLÉ	A.N24	Programa de educação alimentar da Nestlé leva às escolas a temática do desperdício alimentar
NESTLÉ	A.N25	Ano letivo 2023/2024 Este ano letivo continuamos a promover estilos de vida saudáveis e sustentáveis em conjunto com as escolas e famílias portuguesas. Ao longo deste ano letivo, partilhamos materiais e vídeos pedagógicos sobre as 3 grandes temáticas deste ano: Biodiversidade, Desperdício Alimentar e Sazonalidade. Adicionalmente ao Ministério da Educação, parceiro do programa desde 1999, este ano letivo juntam-se a nós a Quinta pedagógica dos Olivais e o Movimento Unidos Contra o Desperdício.
NESTLÉ	A.N26	Lançámos também o concurso escolar "Receitas com os Pés assentes na Terra", onde desafiamos cada turma a criar um livro de receitas saudáveis e sustentáveis, tendo como principal foco a redução do desperdício alimentar, a sazonalidade e a tradição
NESTLÉ	A.N27	A nossa ambição é ajudar 50 milhões de crianças a desenvolver uma vida mais saudável até 2030. A nutrição é a chave para assegurar o desenvolvimento saudável das crianças. Contudo, como sempre, a teoria é bem diferente da prática. Encorajar as crianças a comer mais vegetais e frutas, assegurando que as suas refeições sejam tão nutritivas quanto possível, e incorporando diferentes fontes de proteína – incluindo opções de origem vegetal – pode, por vezes, ser um desafio para os pais e cuidadores. A iniciativa Nestlé por Crianças mais Saudáveis reúne todos os esforços para apoiar os pais e cuidadores na "jornada" de criar crianças saudáveis. Em todo o mundo, a Nestlé está a lançar mais alimentos e bebidas nutritivos para dar resposta às necessidades nutricionais das crianças, enquanto promove a educação alimentar através de programas escolares, bem como dicas práticas para o dia a dia e receitas saudáveis para os pais. Envolver as crianças na preparação das refeições leva-as a adotar hábitos alimentares mais saudáveis – em suma, a qualidade da sua alimentação é superior, tornando-as mais aptas a comer frutas e vegetais. É por isso que incentivamos e advogamos a partilha do momento da preparação e confeção das refeições, pelos pais e crianças.
NESTLÉ	A.N28	O Programa em Portugal O Programa Nestlé por Crianças mais Saudáveis iniciou-se em 1999, em parceria com a Direção-Geral da Educação do Ministério da Educação e ao longo deste tempo tem chegado a milhões de crianças em todo o país. Este programa implementa-se através das escolas mas também com ações diretas às famílias e à comunidade. O Programa Nestlé por Crianças mais Saudáveis tem como foco promover uma alimentação mais saudável e sustentável como parte de um estilo de vida promotor de saúde. O Programa procura dinamizar ações práticas e lúdicas, mantendo um caráter didático, formativo e informativo. As suas iniciativas são estruturadas num clima de respeito pela diversidade, sustentabilidade e equidade, para uma cidadania participada, responsável e inclusiva
NESTLÉ	A.N29	Nutrição, saúde e bem-estar Guiados pelo nosso compromisso com a Nutrição, Saúde e Bem-estar, o nosso objetivo é fornecer as escolhas mais saborosas e saudáveis em todas a nossas categorias de produto. Trabalhamos para aumentar o valor nutricional dos nossos produtos. Esforçamo-nos por melhorar a saúde geral e a acessibilidade do nosso portfólio. As nossas capacidades de inovação estabelecidas desde há longo tempo ajudam-nos a inovar rápida e agilmente. Estamos comprometidos em fornecer informação nutricional transparente e em encorajar estilos de vida saudáveis. Expressamo-lo por meio da nossa assinatura "Somos a Nestlé, the Good food, Good life company".
NESTLÉ	A.N30	Como a Nestlé melhora a saúde das crianças Para combater os problemas de saúde pública globais e satisfazer as preferências dos consumidores, estamos a lançar alimentos e bebidas mais nutritivos ao simplificar as listas de ingredientes e remover corantes artificiais. Usamos enriquecimento de micronutrientes para ajudar a combater os desafios de saúde. Também estamos a reduzir ainda mais nos açúcares, sal e gorduras saturadas, e a aumentar a quantidade de legumes e cereais integrais nos nossos produtos.
NESTLÉ	A.N31	A nutrição é a chave para assegurar o desenvolvimento saudável das crianças. Contudo, como sempre, a teoria é bem diferente da prática. Encorajar as crianças a comer mais vegetais e frutas, assegurando que as suas refeições sejam tão nutritivas quanto possível, e incorporando diferentes fontes de proteína – incluindo opções de origem vegetal – pode, por vezes, ser um desafio para os pais e cuidadores. A iniciativa Nestlé por Crianças mais Saudáveis reúne todos os esforços para apoiar os pais e cuidadores na "jornada" de criar crianças saudáveis. Em todo o mundo, a Nestlé está a lançar mais alimentos e bebidas nutritivos para dar resposta às necessidades nutricionais das crianças, enquanto promove a educação alimentar através de programas escolares, bem como dicas práticas para o dia a dia e receitas saudáveis para os pais. Envolver as crianças na preparação das refeições leva-as a adotar hábitos alimentares mais saudáveis – em suma, a qualidade da sua alimentação é superior, tornando-as mais aptas a comer frutas e vegetais. É por isso que incentivamos e advogamos a partilha do momento da preparação e confeção das refeições, pelos pais e crianças.
NESTLÉ	A.N32	Chegou a nova gama de cereais FITNESS® com 0% açúcares adicionados
NESTLÉ	A.N33	Sabor a chocolate, 0% açúcares adicionados: NESTUM traz inovação ao mercado
NESTLÉ	A.N34	CHOCAPIC renova compromisso nutricional e atinge Nutri-Score A
NESTLÉ	A.N35	VITAL PROTEINS desenvolve receitas para tornar o verão dos portugueses mais saudável e delicioso
NESTLÉ	A.N36	A Nestlé Health Science organizou, em maio de 2022, o CANuG - Curso Anual de Nutrição em Geriatria. O curso foi realizado em parceria com a Associação Portuguesa de Nutrição Entérica e Parantérica (APNEP), a Sociedade Portuguesa de Medicina Interna (SPMI) e o Núcleo de Estudos de Nutrição Clínica (NENC) da SPMI. A iniciativa contou com 130 especialistas, entre eles Médicos de Medicina Interna, Médicos de Medicina Geral e Familiar, Jovens Recém Especialistas e Nutricionistas. O principal objetivo foi a partilha de experiências profissionais e contextos hospitalares distintos a nível nacional em temas como a identificação de doentes desnutridos, a abordagem ao rastreio nutricional ou a intervenção nutricional do doente.
NESTLÉ	A.N37	Plantação de 10 mil árvores Ação de reflorestação da Nestlé Portugal no Pinhal de Leiria
NESTLÉ	A.N38	Através do Nestlé Cocoa Plan, estamos a construir uma cadeia de abastecimento mais responsável para a indústria do cacau e assumimos o compromisso de adquirir 100% do nosso cacau através deste plano, até 2025.
NESTLÉ	A.N39	APOIO ÀS COMUNIDADES Ajudámos no combate à Covid-19 e na integração de refugiados Apoiar quem mais precisa. Em contexto de guerra. Em contexto de crise. Em contexto de pandemia. Assim foi, e assim continuará a ser.

		Esta é uma das missões da Nestlé. E é nesta relação de proximidade com organizações como o Banco Alimentar contra a Fome ou a Cruz Vermelha Portuguesa, ou hospitais por todo o país que assenta este auxílio.
NESTLÉ	A.N40	O QUE É O PROJETO "MAIS FELIZ?" É um projeto que apoia refugiados, famílias e crianças, idosos e sem-abrigo. Trata-se de uma ajuda com o pagamento de bens de primeira necessidade como água, luz, gás, adiantamentos no pagamento de rendas da casa, idas ao dentista, aquisição de cadeiras de rodas, entre outros bens fundamentais
NESTLÉ	A.N41	O início de 2022 ficou marcado pela guerra na Ucrânia. Milhares de refugiados procuraram Portugal e a Nestlé mais uma vez, em parceria com a Cruz Vermelha Portuguesa, doou mais de 100 mil produtos alimentares e 27.000 euros que permitiram equipar, na íntegra, o recém criado Centro de Acolhimento Temporário de Almada. Este é apenas um dos exemplos de uma relação duradoura que tem tido os melhores resultados. Fomos saber mais sobre este apoio com Ana Jorge, presidente da Cruz Vermelha Portuguesa.
NESTLÉ	A.N42	FAZER UM CACAU MELHOR COMEÇA COM A MELHORIA DA VIDA DAS PESSOAS QUE O CULTIVAM
NESTLÉ	A.N43	"PROGRAMA PRO_MOV" À procura de um novo rumo, apostando na requalificação
NESTLÉ	A.N44	Em Portugal, o objetivo é lançar 30 cursos e abrir 600 turmas, em diversas áreas de atividade e regiões do país, requalificando 20 mil portugueses até 2025.
NESTLÉ	A.N45	FITNESS PINK RIBBON 10 anos a apoiar a luta contra o cancro da mama O diagnóstico precoce é fundamental para combater aquele que é o segundo cancro mais mortal entre as mulheres portuguesas. E o mês de outubro de 2022 ficou mais uma vez marcado pela prevenção contra esta doença, com uma série de iniciativas da marca FITNESS, nomeadamente através da campanha Pink Ribbon
NESTLÉ	A.N46	Dá o primeiro passo contra o cancro da mama". Este foi o mote para a campanha lançada pelos cereais FITNESS. Pelo décimo ano consecutivo, a marca associou-se à luta contra o cancro da mama, apoiando a ciência através do iMM-Laço Hub, um projeto dedicado à investigação do cancro da mama, e alertando para a importância do diagnóstico precoce.
NESTLÉ	A.N47	Relação próxima com os agricultores NESCAFÉ trabalha em conjunto com os cafeicultores para testar, estudar e avaliar a eficácia de várias práticas agrícolas regenerativas, com foco em sete origens principais, de onde a marca obtém 90% do seu café: Brasil, Vietname, México, Colômbia, Costa do Marfim, Indonésia e Honduras.
NESTLÉ	A.N48	"Fomos o primeiro mercado do mundo a ter o seu próprio sistema de reciclagem, com o qual fizemos várias atividades de criação de valor para as Comunidades." MARIA TERESA MENDES Business Executive Officer Coffe Portugal
NESTLÉ	A.N49	Em 2023, encerrámos as comemorações do nosso 100º aniversário, através de uma ação de reflorestação no Pinhal de Leiria. Com a plantação de 10.000 pinheiros na zona da Lagoa da Ervedeira cumprimos com o compromisso que assumimos no âmbito do projeto de sustentabilidade "Nestlé 100 Fronteiras".
NESTLÉ	A.N50	Há mais de uma década, lançámos uma iniciativa global para a sustentabilidade do café chamada Nescafé Plan. Como parte deste plano, trabalhamos para reduzir o impacte ambiental do café, desde a produção até às nossas fábricas.
NESTLÉ	A.N51	Para fazer chocolate de qualidade, precisamos de cacau de qualidade. Mas cultivar cacau apresenta inúmeros desafios, como a desflorestação ou as condições de vida nos locais onde o cacau é produzido. É por isso que, desde 2009, a Nestlé tem vindo a apoiar as comunidades produtoras de cacau, através do programa Nestlé Cocoa Plan, tendo assumido o compromisso de, até 2025, todo o cacau utilizado na produção dos seus chocolates ser 100% obtido de forma sustentável.
NESTLÉ	A.N52	"Income Accelerator Program" O programa inovador para combater o trabalho infantil nas plantações de cacau
NESTLÉ	A.N53	Temos três ambições para nos ajudar a atingir o objetivo de desenvolver o poder da alimentação para melhorar a qualidade de vida de todos, hoje e para as gerações futuras. A nossa ambição para 2030 é ajudar 50 milhões de crianças a terem uma vida mais saudável. Como estamos a ajudar as crianças A nossa ambição para 2030 é melhorar as condições de vida de 30 milhões de pessoas nas comunidades onde operamos. Como estamos a melhorar as condições de vida A nossa ambição para 2030 é atingir zero impacte ambiental nas nossas operações.
NESTLÉ	A.N54	Como a Nestlé melhora a saúde das crianças Para combater os problemas de saúde pública globais e satisfazer as preferências dos consumidores, estamos a lançar alimentos e bebidas mais nutritivos ao simplificar as listas de ingredientes e remover corantes artificiais. Usamos enriquecimento de micronutrientes para ajudar a combater os desafios de saúde. Também estamos a reduzir ainda mais nos açúcares, sal e gorduras saturadas, e a aumentar a quantidade de legumes e cereais integrais nos nossos produtos.
NESTLÉ	A.N55	A nossa ambição é ajudar 50 milhões de crianças a desenvolver uma vida mais saudável até 2030. A nutrição é a chave para assegurar o desenvolvimento saudável das crianças. Contudo, como sempre, a teoria é bem diferente da prática. Encorajar as crianças a comer mais vegetais e frutas, assegurando que as suas refeições sejam tão nutritivas quanto possível, e incorporando diferentes fontes de proteína – incluindo opções de origem vegetal – pode, por vezes, ser um desafio para os pais e cuidadores. A iniciativa Nestlé por Crianças mais Saudáveis reúne todos os esforços para apoiar os pais e cuidadores na "jornada" de criar crianças saudáveis. Em todo o mundo, a Nestlé está a lançar mais alimentos e bebidas nutritivos para dar resposta às necessidades nutricionais das crianças, enquanto promove a educação alimentar através de programas escolares, bem como dicas práticas para o dia a dia e receitas saudáveis para os pais. Envolver as crianças na preparação das refeições leva-as a adotar hábitos alimentares mais saudáveis – em suma, a qualidade da sua alimentação é superior, tornando-as mais aptas a comer frutas e vegetais. É por isso que incentivamos e advogamos a partilha do momento da preparação e confeção das refeições, pelos pais e crianças.
NESTLÉ	A.N56	#ThePowerOfYOUth O desemprego jovem é um problema grave em todo o mundo e um grande fator para a pobreza global, além de acarretar repercussões mais abrangentes. Os jovens estão cada vez mais a sair dos meios rurais para procurar trabalho em meios urbanos, criando um futuro incerto para as aldeias que deixam para trás. Com o aumento da procura de alimentos em todo o mundo, a agricultura tem de ser vista como uma carreira financeiramente viável para os jovens.
NESTLÉ	A.N57	Porquê a iniciativa Nestlé needs YOUth? Acreditamos que as comunidades não têm como prosperar se não criarem um futuro promissor para as gerações mais novas. É por isso que estamos decididos a ajudar os jovens a desenvolver as suas competências para que possam encontrar empregos enriquecedores ou criar os seus próprios negócios, de forma a ajudar a criar comunidades que prosperam e apoiam os . de Desenvolvimento Sustentável das Nações Unidas (ODS) Criada em 2013, a iniciativa Nestlé needs YOUth ajuda a dotar os jovens das competências de que precisam para prosperar nos locais de trabalho do futuro.
NESTLÉ	A.N58	Nos 10 anos da iniciativa Nestlé needs YOUth, a Nestlé Portugal deu oportunidade a 772 jovens de ingressarem no mercado de trabalho, através de estágios, e contratou mais de 2600 jovens até 30 anos.
NESTLÉ	A.N59	Para o Planeta A nossa ambição para 2030 é atingir zero impacte ambiental nas nossas operações.
NESTLÉ	A.N60	Nestlé lança em Portugal os primeiros produtos com NutriScore Novo sistema de rotulagem ajuda nas escolhas mais saudáveis Voltar aos Comunicados de Imprensa A Nestlé está a iniciar em Portugal a implementação do Nutri-Score

		nas embalagens dos seus produtos. Este novo rótulo nutricional consiste num código de cores e de letras, colocado na frente das embalagens, que ajuda os consumidores a fazerem escolhas alimentares mais saudáveis, baseadas nos níveis de classificação que vão de A-Verde: o mais saudável, a EVermelho: o menos saudável. As primeiras embalagens de produtos Nestlé a incluírem este novo sistema de informação no nosso país, e que já estão no mercado, são os cereais de pequeno-almoço FITNESS Original e NESQUIK Alphabet, ambos classificados com A-Verde. A estes seguir-se-á a gama de produtos flexitarianos Garden Gourmet. Este sistema, já implementado em diversos países europeus e que agora é também adotado pela Nestlé em Portugal, é considerado como o mais eficiente na informação rápida ao consumidor sobre as características e os benefícios de um determinado produto. Em vários Estados-Membro da União Europeia (EU), grupos de consumidores, legisladores, académicos, empresas de alimentação e retalhistas estão a pedir que o rótulo nutricional Nutri-Score se torne obrigatório na UE. “Dar aos consumidores as melhores ferramentas de informação para poderem fazer escolhas mais acertadas para si e para as suas famílias é um dos compromissos da Nestlé. O Nutri-Score oferece agora uma solução clara que ajuda o consumidor a decidir mais rápido, Para além disso, o Nutri-Score motiva as empresas de alimentos e bebidas a acelerar o desenvolvimento de produtos nutricionalmente mais equilibrados. O Nutri-Score não deve apenas ajudar a Nestlé a elevar a fasquia; convidamos toda a indústria a juntar-se a nós.”, afirma Paolo Fagnoni, Diretorgeral da Nestlé Portugal. Com um em cada dois adultos europeus com sobrepeso ou obesidade - aumentando a probabilidade de doenças cardíacas, diabetes e cancro - é claramente necessária uma ação mais forte para ajudar a conter esta crise de saúde pública. A rotulagem nutricional foi identificada pelos governos nacionais e por especialistas internacionais em saúde como uma das principais ferramentas políticas que podem ser usadas para apoiar os consumidores a fazer escolhas alimentares saudáveis e informadas num piscar de olhos. Pesquisas realizadas em vários países mostram que o Nutri-Score é atualmente o esquema com melhor desempenho, na medida em que ajuda os consumidores a compararem a qualidade nutricional dos alimentos numa gama de produtos e a fazer escolhas mais saudáveis no supermercado.
NESTLÉ	A.N61	Em Portugal, a Nestlé conta com mais de 900 fornecedores locais das mais variadas áreas de atividade, sendo esta também uma forma de criar e partilhar valor com a sociedade portuguesa, de apoiar a resiliência do tecido económico nacional e das comunidades onde as empresas estão inseridas. Em 2022, a Nestlé comprou em Portugal 53% das suas necessidades com um investimento total de 140 milhões de euros.
NESTLÉ	A.N62	Nestlé Portugal planta 10 mil árvores no Pinhal de Leiria
NESTLÉ	A.N63	Nestlé Baby&me lança nova temporada de “Pais Sem Livro de Instruções”, o seu podcast sobre parentalidade, com Helena Coelho como anfitriã e Pedro Teixeira como primeiro convidado
NESTLÉ	A.N64	Nespresso e Café Joyeux juntos a promover a inclusão no trabalho em Portugal
NESTLÉ	A.N65	Nestlé Portugal recorre a combustível de origem renovável para camiões de logística
NESTLÉ	A.N66	Os cereais FITNESS apoiam a luta contra o cancro da mama há 11 anos
NESTLÉ	A.N67	Programa de educação alimentar da Nestlé leva às escolas a temática do desperdício alimentar
NESTLÉ	A.N68	Parceria entre Nestlé e Too Good To Go vende mais de 23.500 Surprise Bags em três anos
NESTLÉ	A.N69	Surfistas Marta Paço, João Kopke e Teresa Abraços juntam-se numa talk sobre os benefícios do surf para a saúde mental
NESTLÉ	A.N70	BUONDI® promove surf como terapia para a saúde mental
NESTLÉ	A.N71	João Francisco Lima, José Mata e João Kopke juntam-se numa talk sobre os benefícios do surf para a saúde mental§
NESTLÉ	A.N72	NESTUM soma mil colmeias oferecidas aos apicultores portugueses
NESTLÉ	A.N73	Nestlé Portugal conseguiu reduzir 47% de emissões de gases com efeito de estufa na última década
NESTLÉ	A.N74	“Nestlé 100 Fronteiras” desafia colaboradores a implementarem ações de sustentabilidade por todo o país
NESTLÉ	A.N75	Nespresso doa mais 78 toneladas de arroz ao Banco Alimentar através do projeto ‘Reciclar é Alimentar’
NESTLÉ	A.N76	Patrícia Mamona associa-se aos cereais FITNESS na luta contra o cancro da mama num grande laço humano
NESTLÉ	A.N77	Parceria entre Nestlé e Universidade NOVA é alargada à Faculdade de Ciências e Tecnologia
NESTLÉ	A.N78	João Manzarra visita plantações de NESCAFÉ® na Colômbia
NESTLÉ	A.N79	ROCK IN RIO e BUONDI: Evento gerou mais de 150 quilos de borras de café para aplicar no jardim do Parque da Bela Vista
NESTLÉ	A.N80	VITAL PROTEINS junta caras conhecidas para celebrar o Dia Mundial do Yoga
NESTLÉ	A.N81	Três startups vão inovar com a Nestlé em projetos que prometem ajudar consumidores a terem hábitos mais saudáveis
NESTLÉ	A.N82	Nestlé promove práticas sustentáveis junto de crianças através dos “Heróis da Reciclagem”
NESTLÉ	A.N83	Movimento Juntos Pelas Abelhas reúne caras conhecidas
NESTLÉ	A.N84	Apoio da Nestlé aos refugiados permite abertura de novo Centro de Acolhimento da Cruz Vermelha Portuguesa
NESTLÉ	A.N85	NESTUM® oferece mais 300 colmeias e homenageia apicultores nas suas embalagens
NESTLÉ	A.N86	NESTUM® volta a apoiar a apicultura nacional com oferta de mais 300 colmeias
NESTLÉ	A.N87	Nestlé e Nova SBE lançam a 4ª edição do programa de Open Innovation START and CO. 2022
NESTLÉ	A.N88	Nestlé doou mais de 600 mil produtos em 2021
NESTLÉ	A.N89	Arroz doado pela Nespresso ao Banco Alimentar tem nova embalagem 100% reciclável
NESTLÉ	A.N90	Como podemos contribuir para um mundo mais sustentável através de pequenas alterações na nossa alimentação? Reunimos oito dicas práticas inspiradas por várias fontes para o ajudar nesta jornada: 1 1. Ingerir as porções diárias de fruta e hortícolas recomendadas. 2. Aumentar o consumo diário de leguminosas e dar preferência aos cereais integrais. Já experimentou os cereais integrais Nutri-Score A! NESTLÉ FITNESS Original ? A melhor parte é que são 3. Optar por produtos da época e de maior proximidade. 4. Incluir mais alimentos e bebidas vegetais na alimentação diária. Faça o teste com os seus amigos! Experimente um churrasco com o nosso de origem vegetal de diferença? Sensational Burger GARDEN GOURMET , com Nutri-Score A. Será que alguém dá pela 5. Opte por peixe e marisco de origem sustentável ou alternativas vegetais a estes alimentos. Já ouviu falar no VUNA ? Sim, leu bem! É uma das nossas mais recentes inovações, com Nutri-Score B, capaz de dar aquele toque delicioso à sua salada, wrap ou poke bowl. E está disponível nos supermercados em Portugal! 6. Evite desperdiçar comida. Faça a sua lista de compras consoante o planeamento semanal de refeições e use a criatividade na cozinha para aproveitar sobras. 7. Procure embalagens recicláveis, evite embalagens desnecessárias e recicle-as nos contentores adequados. 8. Aumente gradualmente o número de refeições semanais à base de proteína vegetal. Temos produtos e receitas incríveis que lhe vão servir de ponto de partida:
NESTLÉ	A.N91	NESTLÉ APOIA STARTUPS PRONTAS A INOVAR 31 projetos apoiados em quatro anos
APN	A.N92	A APN apresenta a possibilidade de atribuição de um selo a produtos e serviços que preencham os requisitos de parceria com a mesma: Nesquik o% açúcares adicionados
APN	A.N93	Patrocínio Congresso Alimentação e Nutrição 2022

APN	A.N94	Patrocínio Congresso Alimentação e Nutrição 2023
NMS	A.N95	NMS e Nestlé juntas no desenvolvimento de produtos alimentares inovadores Parceria pioneira entre a Indústria Alimentar e a Academia 31-Mai-2022 A Nestlé e os alunos finalistas da licenciatura em Ciências da Nutrição da NOVA Medical School (NMS) trabalharam em conjunto, nos últimos três meses, no desenvolvimento de produtos à base de cereais, saudáveis e sustentáveis desde a sua origem, no âmbito projeto "Cereals for All – Creating the Future of Cereals". Esta é uma parceria inédita em Portugal, entre uma empresa da indústria alimentar e a Academia, que resulta de um protocolo de colaboração entre a Nestlé Portugal e a NOVA Medical School com vista à inovação em produtos alimentares que se distingam pelo seu caráter "healthy and sustainable by design" e que sejam nutricionalmente adequados para a população-alvo à qual se destinam. Deste trabalho, apresentado no dia 30 de maio a júri, em pitch final, resultaram cinco projetos nas áreas de Saúde Infantil, Conveniência, Saúde Mental, Saúde da Mulher e Envelhecimento Ativo que passarão agora às respetivas fases de teste e prototipagem. Ao longo do processo de desenvolvimento os alunos tiveram a oportunidade de aplicar os conhecimentos adquiridos durante o curso no processo criativo de inovação de um novo produto alimentar à base de cereais, sempre acompanhados pela direção técnica das equipas de Inovação e Marketing da Nestlé Portugal e com foco nas necessidades reais do mercado, utilizando o know-how da Nestlé em produção alimentar. "O encontro entre a NOVA Medical School e a Nestlé traz uma possibilidade transformadora na criação de novos produtos na área dos cereais, um segmento muito importante para o nosso negócio. Com esta parceria, pretendemos trazer uma perspetiva fresca para a criação de novos conceitos, pretendemos envolver a geração de nutricionistas do futuro neste processo e darlhes poder. Mas queremos também aprender sobre o conhecimento científico mais recente na criação de produtos à base de cereais, permitindo que sejam reconhecidos pela comunidade científica como "saudáveis e sustentáveis desde a origem". Esta visão é possível com esta inédita parceria com a NOVA Medical School e a Nestlé. Estamos muito satisfeitos com o trabalho realizado até nesta fase, não são só com a abertura, mas também com a adesão dos alunos." Afirma Andreia Vaz, Diretora Ibérica de Nestlé Innovation Beyond the Core. Por seu lado, Helena Canhão, Diretora da NOVA Medical School, sustenta que "esta parceria é muito importante e vai ao encontro da estratégia da NOVA Medical School que aposta num ensino baseado na aquisição de competências práticas, no raciocínio clínico e na evidência científica, colocando, sempre, o conhecimento ao serviço da comunidade". Ao longo dos três meses de trabalho conjunto, os alunos da Licenciatura em Ciências da Nutrição da NMS desenvolveram um trabalho imersivo com a Nestlé, tendo visitado uma das suas principais fábricas em Portugal, a Fábrica de Avanca, que produz, entre outras, marcas icónicas como o CERELAC, o NESTUM, o CHOCAPIC e MOKAMBO. Foram ainda realizadas Power Sessions, para que os alunos aprendessem sobre o processo de desenvolvimento de novos produtos, e sessões de mentoria com os vários especialistas da Nestlé.
NMS	A.N96	(…) o evento de apresentação e de assinatura protocolar entre a Nestlé Portugal e a NOVA Medical School que deu início à segunda edição do Projeto "Cereals for All – Desenvolver o Futuro dos Cereais" – uma parceria inovadora entre a indústria alimentar e o meio académico. O projeto faz parte da unidade curricular de estágio e envolve todos os alunos finalistas da Licenciatura em Ciências da Nutrição, no processo de desenvolvimento de produtos alimentares. Durante a apresentação, foi feito o balanço da primeira edição, com a participação e testemunho de um aluno como porta-voz da turma anterior. O evento teve lugar no edifício da NOVA Medical School, em Lisboa, e terminou com a assinatura do protocolo por ambas as entidades. Nesta segunda edição, a parceria entre a Nestlé Portugal e a NOVA Medical School pretende continuar a proporcionar aos alunos o contacto com o processo de desenvolvimento de novos produtos alimentares, saudáveis e sustentáveis, à base de cereais. O Projeto inclui várias etapas, a começar pelo próprio evento de lançamento da II Edição, seguindo-se uma visita à Fábrica da Nestlé em Avanca, com os alunos participantes. Durante o seu trabalho os alunos serão inspirados em duas power sessions de partilha de conhecimento das várias etapas de um processo de desenvolvimento de um produto alimentar. Ao longo do Projeto, os alunos têm a oportunidade de aliar os conhecimentos adquiridos durante os seus estudos ao processo criativo e tecnológico de inovação em produtos alimentares, através da mentoria dada pelas equipas técnicas da Nestlé Portugal. "Estamos entusiasmados por anunciar o lançamento da segunda edição do projeto 'Cereals for All'. O sucesso do projeto do ano passado foi evidente e ficou patente no interesse e na dedicação dos estudantes que nos apresentaram conceitos inovadores e 'out of the box' ao longo do processo de desenvolvimento dos produtos", afirma Ana Leonor Perdigão, Nutrition, Health and Wellness Manager da Nestlé Portugal. "Esta parceria única entre a Nestlé e a NOVA Medical School continua a alargar as fronteiras da inovação na indústria alimentar, e esperamos ansiosamente ver os novos produtos e ideias que sairão desta segunda edição.", concluiu. Por seu lado, Conceição Calhau, responsável pela Licenciatura em Ciências da Nutrição, da NOVA Medical School, afirma: "A parceria com a Nestlé permite à NOVA Medical School contribuir com a sua experiência em nutrição para o desenvolvimento de produtos mais saudáveis e inovadores, enquanto apresenta uma oportunidade de aprendizagem única aos seus alunos de Ciências da Nutrição, podendo aplicar a sua experiência teórica num ambiente prático de competências adquiridas durante os 4 anos de curso e aquisição de competências nas áreas do negócio, empreendedorismo, gestão de prioridades e pressão empresarial, muito importante para preparação para o mercado de trabalho de hoje. Estamos entusiasmados para ver os resultados deste segundo ano." O Projeto "Cereals for All" é único em Portugal e, em 2022, reuniu 14 alunos finalistas da Licenciatura em Ciências da Nutrição e várias equipas de desenvolvimento de produtos da Nestlé, com o objetivo de reforçar as competências dos alunos na inovação de produtos alimentares saudáveis e sustentáveis, à base de cereais, nutricionalmente adaptados a diferentes perfis de consumidores. A primeira edição do programa resultou em cinco projetos nas áreas de Saúde Infantil, Conveniência, Saúde Mental, Saúde da Mulher e Envelhecimento Ativo
NMS	A.N97	Nestlé, patrocinador do Open Day 2018, 2019
NMS	A.N98	Prémio NESTLÉ em Projeto de Investigação 2022: Ao abrigo da parceria entre a NMS e a NESTLÉ
NMS	A.N99	Prémio NESTLÉ em Projeto de Investigação 2023: Ao abrigo da parceria entre a NMS e a NESTLÉ
FMUL	A.N100	Neste sentido, nos dias 14 e 15 de Setembro de 2023, o Laboratório de Nutrição da Faculdade de Medicina da Universidade de Lisboa (FMUL) em parceria com a Sociedade Portuguesa de Medicina do Estilo de Vida, organizou a primeira edição do Curso Estilo de Vida Saudável nos Primeiros 1000 Dias de Vida. Este curso contou com mais de 125 participantes das mais diversas áreas da saúde, nomeadamente médicos de medicina geral e familiar, pediatras, nutricionistas, enfermeiros, psicólogos, fisioterapeutas, fisiologistas do exercício, professores e estudantes de cursos na área da saúde. Por fim, adotámos uma política de sustentabilidade ao longo de todo o curso. Não houve oferta de brindes, tão comuns nos congressos da área da saúde, e pedimos a cada participante para trazer a sua garrafa de água, caderno e caneta. Procurámos ainda oferecer produtos da época em embalagens recicladas e recicláveis nos coffee breaks e nos almoços. Agradecemos por isso, o apoio da Nestlé Nutrition Institute, The Revolution Food, Vale da Rosa e EPAL que contribuíram para nos ajudar a cumprir esta missão.

ESB	A.ESB1	A Escola Superior de Biotecnologia desenvolve intercâmbios com múltiplas instituições dentro e fora de Portugal, incluindo centros de investigação, universidades, empresas e unidades de saúde: Modelo Continente Hipermercados, Bimbo, Danone Portugal, Imperial Produtos Alimentares, Nestlé Portugal, Primor Charcutaria Prima, Sonae, Cerealis, Vitacress Portugal
ESB	A.ESB4	A Semana Aberta é uma iniciativa da Escola Superior de Biotecnologia que se realiza desde 1992 e se repete todos os anos na primavera. Em 2024 a 32ª edição decorre nos dias 10, 11 e 12 de abril e está disponível sem custos para as escolas secundárias – todos os alunos dos cursos de ciências e tecnologias são bem vindos. As inscrições devem decorrer preferencialmente até 22 de março de 2024. Patrocinadores: Cerealis, Nestlé, Sovena

NOVARROZ

Website	Code	CPA EXAMPLES
NOVARROZ	A.NO1	NUTRIÇÃO Veja os conselhos e dicas que temos para melhorar a sua alimentação e a sua vida! Reduza o sal e ganhe mais saúde! São as calorias que nos movem? O que é o glúten, onde está e quando não deve ser consumido? O ferro e os seus alimentos potenciadores/inibidores Hidratação no Verão No verão o que devo levar no piquenique? Água: tão básica, tão indispensável Boas práticas para um coração mais saudável Páscoa mais leve 10 benefícios do arroz selvagem para saúde A (des)valorização das leguminosas Alimentação e saúde Arroz e legumes = proteína de qualidade O arroz e a saúde Porquê cereais integrais? Como organizar os alimentos no nosso frigorífico? Alimentação infantil saudável O arroz, alimento universal Quadra natalícia: tem muita comida de sobra e não quer desperdiçar? Alimentação vegetariana e macrobiótica Como manter os hábitos saudáveis no inverno Proteína: quantidade e qualidade de mãos dadas Será que sabemos mesmo distinguir carnes vermelhas e carnes brancas Como a proteína nos pode ajudar e como incluí-la na dieta?
NOVARROZ	A.NO2	Segundo a Organização Mundial da Saúde (OMS) a maioria das pessoas consome muito sal, em média de 9 a 12 gramas, sendo o dobro da ingestão máxima recomendada. Estima-se que 2,5 milhões de mortes poderiam ser evitadas a cada ano se o consumo global de sal fosse reduzido ao nível recomendado. Os Estados membros da OMS decidiram reduzir em 30% o consumo de sal da população mundial em 2025, de forma a melhorar o estado de saúde da mesma. O sal ou sal de cozinha é uma substância cristalina quimicamente designada por cloreto de sódio e composta por dois minerais, o sódio e o cloro, sendo que este último confere o sabor salgado. Embora, estes minerais sejam essenciais para um bom funcionamento do nosso organismo, o seu consumo excessivo trás inúmeras consequências, principalmente o sódio, daí ser essencial o seu uso e consumo com parcimónia. Em muitos países, 80% do sal ingerido provém de alimentos processados. Muitas vezes associa-se alimentos salgados a alimentos com alto teor de sal, mas isso não implica que alguns alimentos que aparentemente não parecem salgados não contenham um alto teor de sal, podem sim conter alguns ingredientes que podem mascarar o seu sabor, como é o caso dos açúcares. Assim, a leitura dos rótulos é sempre importante e nunca deve ser descartada. As consequências do consumo excessivo de sal (Direção Geral da Saúde): Aumenta o risco do aparecimento de determinados tipos de cancro (ex.: estômago) Aumenta o risco de aparecimento de hipertensão arterial Risco aumentado de doenças cardiovasculares Sobrecarga do funcionamento do rim (há um maior esforço feito pelo rim para excretar o excesso de sódio) Maior retenção de líquidos pelo organismo, o que implica aumento do peso e contribui para o aparecimento de celulite A quantidade de sal: De acordo com a OMS, o ideal é não exceder os 5g de sal por dia para um adulto (aproximadamente 1 colher de chá rasa) e 3g diárias para as crianças, sendo que 5g de sal corresponde a 2g de sódio. As várias designações de sódio presentes nos rótulos dos alimentos: Teor de sal, sódio, NaCl (cloreto de sódio), Na (símbolo químico do sódio), glutamato monossódico, bicarbonato de sódio, bissulfato de sódio, fosfato dissódico, hidróxido de sódio e propionato de sódio. É importante ler os rótulos dos alimentos para saber o que está a comprar. Evite os alimentos que contenham mais que 5% da dose diária recomendada (DDR) de sódio ou com mais de 1,5g de sal por 100g, o correspondente a 0,6g de sódio. No caso das bebidas, evitar as que contenham mais de 0,75g de sal ou 0,3g de sódio por 100mL. Os produtos/alimentos mais ricos em sal: Carnes processadas – enchidos, fumados, salsichas, hambúrguer,.. Bacalhau e algas marinhas – importante que sejam bem demolhados Algumas conservas – milho, ervilhas, feijão, grão-de-bico, atum, sardinha, cavala,.. Refeições précozinhadas e sopas instantâneas Rissóis, croquetes, chamuças, bolinhos de bacalhau, panados, empadas e folhados Molhos embalados e caldos concentrados Batatas fritas de pacote e aperitivos salgados Frutos oleaginosos com sal Determinadas bolachas e cereais de pequeno-almoço Margarinas e manteigas com sal, e queijos curados Azeitonas Refrigerantes – o sódio pode ser adicionado a sumos como conservante Águas minerais gaseificadas Dicas para reduzir o consumo diário de sal: Ler os rótulos dos alimentos que compra, procurando sempre os que apresentam menor teor de sal No caso de o produto não conter informação nutricional no rótulo, leia a lista dos ingredientes tendo em atenção se existem ou não algumas das várias designações de sódio, que acrescentam aos alimentos quantidades significativas de sódio Reduzir gradualmente a quantidade de sal adicionada durante a confeção dos alimentos Todo o consumo de sal deve ser iodado, ou seja, “enriquecido” com iodo (composto essencial para o desenvolvimento saudável do cérebro do feto e do filho jovem e para otimizar as funções mentais em geral) Não levar o saleiro para a mesa, de forma a evitar a adição de sal fino aos pratos já cozinhados Substituir o sal usado na confeção dos alimentos por ervas aromáticas, especiarias, vinho ou sumo de limão Marinar a carne, o peixe, o tofu, o seitan ou o tempeh em vinhas de alhos ou com outros temperos sem sal antes de os confecionar, assim o sabor e aroma dos temperos adicionados ficarão mais intensos e mais saborosos No restaurante pode pedir para preparar o seu prato com menos sal e no caso de serem pratos com molhos peça que sejam servidos em separado Consumir frutas e vegetais, estes contêm potássio, mineral que ajuda a baixar a pressão arterial Por: Bernardete Carvalho, Nutricionista 0411N Data: 26/02/2018
NOVARROZ	A.NO3	Uma embalagem de arroz 100% em papel? Existe e é portuguesa
APN	A.NO4	Patrocínio Congresso Alimentação e Nutrição 2022
FMUL	A.FMUL1	O TASTY já conta com a participação entusiasta de 12 doentes que, de forma aleatória, foram alocados ao grupo de controlo ou ao grupo de intervenção. Os doentes que constituem o grupo de intervenção são convidados a seguir um padrão alimentar tipicamente mediterrânico enriquecido em alimentos fermentados, durante 12 semanas, sob acompanhamento por nutricionistas. A intervenção nutricional que implementamos no TASTY vai muito além da entrega de um plano alimentar. Passa também por promover a literacia nesta área e capacitar os doentes para melhores escolhas alimentares e melhores técnicas culinárias. Contamos com a ajuda de vários parceiros para pôr em prática este plano ambicioso, nomeadamente o Pingo Doce, que entrega aos doentes semanalmente cabazes de alimentos selecionados por nós, com a Sovena, que oferece azeite virgem extra Oliveira da Serra (não pode faltar na Dieta Mediterrânica!), com a Nestlé, que oferece os leites fermentados Kefir, e com diversas empresas da indústria das

		conservas (Bom Petisco, Minerva, Esip e a Cofisa) que oferecem conservas de peixe gordo (sardinha e cavala em azeite virgem extra ou em tomate).
FCNAUP	A.FCNAUP2	A FCNAUP promove visitas de estudo a instituições ou empresas em contexto de aprendizagem, criando momentos de partilha de experiências e necessidades profissionais: Novarroz, Cozinha Central do Pingo Doce

NUTRIENVESTE

Website	Code	CPA EXAMPLES
NUTRIENVESTE SOVENA	A.NU1	A nossa aposta na inovação tem reflexo nos nossos parceiros: o IBET, Instituto de Biologia Experimental e Tecnológica, é a maior organização sem fins lucrativos na área da investigação biotecnológica em Portugal. O seu papel é essencial para nos ajudar a assegurar o desenvolvimento de projetos na área agroindustrial à escala piloto e para a transferência de tecnologias e processos para a indústria. Trabalhamos juntos na área da qualidade nutricional e sensorial, na estabilidade, genuinidade e funcionalidade dos alimentos e na segurança alimentar. Trabalhamos juntos para nos diferenciarmos, inovarmos e certificarmos internacionalmente os nossos produtos e processos
NUTRIENVESTE SOVENA	A.NU2	A AESE é a primeira Business School em Portugal e foi fundada em 1980, pela Associação de Estudos Superiores de Empresa que a constituiu à imagem do IESE Business School (a escola de pósgraduação em gestão da Universidade de Navarra) para dar aos executivos as ferramentas e técnicas de gestão, bem como os valores centrados na ética e no humanismo, que permitem o seu contínuo aperfeiçoamento pessoal e profissional. Ao longo dos anos, temos vindo a desenvolver e a consolidar uma relação de parceria com a AESE neste domínio da formação executiva, possibilitando a muitos dos nossos quadros o reforço de competências de direção e gestão de empresas, de liderança, entre outras competências críticas para o negócio. A formação tem sido assegurada pela participação em programas de Executive MBA ou em Programas Executivos como o PADE, o PDE ou o GAIN.
NUTRIENVESTE SOVENA	A.NU3	BCSD Portugal A SOVENA aderiu ao Business Council for Sustainable Development (BCSD) Portugal, uma associação sem fins lucrativos que agrega e representa mais de 130 empresas de referência em Portugal, que se comprometem ativamente trabalhando em conjunto para acelerar a transição para um mundo mais sustentável.
NUTRIENVESTE SOVENA	A.NU4	Global Commitment A Sovena aderiu ao "The New Plastics Economy Global Commitment", dirigido pela Ellen Macarthur Foundation, que une empresas, governos e outras organizações, numa visão comum e compromisso de redução e uso mais racional do plástico, reduzindo seu impacto no planeta. Nossa meta é uma redução de 3.500 toneladas de plástico virgem até 2025 (31%), através do aumento de PET reciclado (28%) e de uma redução efetiva de plástico (3%). Acompanhe nosso progresso aqui Continuaremos a traçar novos caminhos na nossa história, para construir futuros mais sustentáveis.
NUTRIENVESTE SOVENA	A.NU5	Unidos contra o desperdício A Sovena aderiou através das marcas Fula e Oliveira da Serra Unidos à iniciativa Unidos Contra o Desperdício, plataforma coliderada em Portugal pelo Banco Alimentar.
NUTRIENVESTE SOVENA	A.NU6	O Pacto para Gestão da Água é uma iniciativa que tem como propósito colocar o tema da ÁGUA no centro da agenda nacional Portuguesa. As entidades parceiras desta iniciativa assumem a sua responsabilidade em adotar medidas mais sustentáveis de forma a contribuir para uma gestão mais eficiente da água.
NUTRIENVESTE SOVENA	A.NU7	Too Good To Go | Luta contra o desperdício Reforçando a sua estratégia na área da sustentabilidade, a marca Oliveira da Serra assinou uma parceria com a Too Good To Go, plataforma que disponibiliza produtos e refeições que seriam potencialmente desperdiçados e os vende abaixo do preço normal em formato Magic Boxes. Contribuir activamente com os nossos produtos para a economia circular é uma decisão que demonstra a nossa forte aposta no desenvolvimento de um consumo mais sustentável. É, sem dúvida, neste tipo de iniciativas que pretendemos participar, unindo esforços para o principal objectivo: consciencializar e promover o desperdício zero.
NUTRIENVESTE SOVENA	A.NU8	Na Sovena, o nosso compromisso com a excelência estende-se para além das nossas marcas. Somos uma família de empresas unidas com uma visão comum de inovação e responsabilidade socioambiental. Assim, como as nossas marcas icónicas que se tornaram parte integrante das cozinhas de milhões de pessoas, as empresas do Grupo Sovena também têm deixado a sua marca nos setores em que atuam, partilhando firmemente o nosso compromisso com a qualidade e a preservação do planeta. As empresas do grupo estão empenhadas em satisfazer as necessidades em constante evolução dos consumidores, contribuindo para um mundo mais saudável e sustentável. Mantemo-nos atentos às tendências globais e às preferências locais, permitindo-nos expandir continuamente a nossa oferta e manter a nossa relevância.
NUTRIENVESTE SOVENA	A.NU9	Ecovadis Rat ing Em 2022, a Sovena recebeu a medalha de bronze da Ecovadis, uma plataforma colaborativa que permite às empresas monitorizar o seu desempenho no desenvolvimento sustentável. Este prémio é um reconhecimento do nosso trabalho nas áreas do ambiente, trabalho e direitos humanos, ética e compras sustentáveis. Na Sovena, queremos criar, continuamente, valor para as Pessoas e o Planeta. Só indo ao encontro das suas necessidades atuais e futuras nos permitirá, e aos nossos produtos, fazer a diferença. Vamos continuar a alimentar o futuro!
NUTRIENVESTE SOVENA	A.NU10	Act4nature | Proteção da biodiversidade Estudos científicos recentes alertam que o planeta está a perder biodiversidade a um ritmo sem precedentes, aproximando-se rapidamente de um ponto sem retorno. De acordo com estimativas do World Economic Forum, em 2020, mais de metade do valor económico mundial gerado é moderada ou altamente dependente da natureza, estando assim exposto à perda da natureza, o que se traduz em enormes riscos para as empresas e para as suas cadeias de valor. Com a iniciativa act4nature Portugal, o BCSD Portugal continua a mobilizar as empresas para atuarem em prol da natureza, através da concretização de ações de proteção, promoção e restauro da biodiversidade e dos serviços dos ecossistemas que contribuam para parar e reverter a sua perda até 2030.
NUTRIENVESTE SOVENA	A.NU11	No âmbito da Responsabilidade Social Corporativa, algumas iniciativas que nos inspiram e nas quais procuramos atuar, vão desde temas como: Impacto Ambiental e nos Recursos Naturais Segurança Alimentar Investimento no Capital Humano Melhoria das Condições de Trabalho e Sociais dos nossos Colaboradores Educação Nutricional nas Comunidades Envolventes Apoio a Instituições de Solidariedade Social
NUTRIENVESTE SOVENA	A.NU12	A S o ve n a Gro u p e a Nu t r i fa rms S A re c e b e ram o Prém i o C o n s ume r We l l - Being 16 de Fevereiro de 2023 | Sovena Group A Sovena Group e a Nutrifarms SA receberam o Prémio Consumer Well-Being atribuído pelo CORE (Center for Consumer Well-being & Retail Innovation) da Católica Lisbon School of Business and Economics.
NUTRIENVESTE SOVENA	A.NU13	Crescer com um propósito é alimentar o futuro. A nossa estratégia FEEDING FUTURES FOR A PLANET THAT PROSPERS AND FOR PEOPLE THAT THRIVE assenta no nosso propósito e impulsiona a Sovena para uma melhoria constante. Promover uma alimentação cada vez mais diversificada e sustentável através da qualidade e inovação dos nossos produtos, da utilização de uma agricultura que proteja a natureza e de uma transformação eficiente e circular. Promovemos uma equipa de funcionários empenhados e conectados, uma comunidade e consumidores informados acerca de dietas diversas. A nossa mais recente avaliação de materialidade deu lugar à definição da nossa estratégia, estruturada em cinco compromissos e com dois potenciadores fundamentais que garantem o nosso sucesso: os mais

		elevados padrões de QUALIDADE e SEGURANÇA ALIMENTAR e uma forte cultura de INOVAÇÃO e DESENVOLVIMENTO.
NUTRIENVESTE SOVENA	A.NU14	Sovena investe 50 milhões de euros ao longo deste ano 25 de Julho de 2023 | JN Apesar de querer diversificar o negócio, o grupo continua a estar "muito focado e baseado nas gorduras alimentares", diz o seu CEO, Jorge de Melo. A empresa deu um aumento salarial médio de 5,5%.
FMUL	A.FMUL1	O TASTY já conta com a participação entusiasta de 12 doentes que, de forma aleatória, foram alocados ao grupo de controlo ou ao grupo de intervenção. Os doentes que constituem o grupo de intervenção são convidados a seguir um padrão alimentar tipicamente mediterrânico enriquecido em alimentos fermentados, durante 12 semanas, sob acompanhamento por nutricionistas. A intervenção nutricional que implementamos no TASTY vai muito além da entrega de um plano alimentar. Passa também por promover a literacia nesta área e capacitar os doentes para melhores escolhas alimentares e melhores técnicas culinárias. Contamos com a ajuda de vários parceiros para pôr em prática este plano ambicioso, nomeadamente o Pingo Doce, que entrega aos doentes semanalmente cabazes de alimentos selecionados por nós, com a Sovena, que oferece azeite virgem extra Oliveira da Serra (não pode faltar na Dieta Mediterrânica!), com a Nestlé, que oferece os leites fermentados Kefir, e com diversas empresas da indústria das conservas (Bom Petisco, Minerva, Esip e a Cofisa) que oferecem conservas de peixe gordo (sardinha e cavala em azeite virgem extra ou em tomate).
ESB	A.ESB4	A Semana Aberta é uma iniciativa da Escola Superior de Biotecnologia que se realiza desde 1992 e se repete todos os anos na primavera. Em 2024 a 32ª edição decorre nos dias 10, 11 e 12 de abril e está disponível sem custos para as escolas secundárias – todos os alunos dos cursos de ciências e tecnologias são bem vindos. As inscrições devem decorrer preferencialmente até 22 de março de 2024. Patrocinadores: Cerealis, Nestlé, Sovena

PEPSICO

Website	Code	CPA EXAMPLES
PEPSICO	A.P1	Além disso, há mais de 25 anos que trabalha com a SUMOL+COMPAL, responsável pelo engarrafamento e distribuição das nossas bebidas refrescantes por todo o território português. Graças a este acordo de franchising, ajuda a criar mais de 150 postos de emprego indireto.
PEPSICO	A.P2	MARKETING RESPONSÁVEL Enquanto empresa global de alimentos e bebidas de confiança, temos a responsabilidade importante de comercializar os nossos produtos de forma rigorosa e transparente a todos os consumidores~ Espera-se que represente sempre os nossos produtos de forma honesta, responsável e em conformidade com todas as leis e regulamentos respetivos nos países onde operamos~
PEPSICO	A.P3	Se o seu trabalho envolver marketing, nunca deve: EXAGERAR OU REPRESENTAR MAL AS QUALIDADES OU EMBALAMENTO DOS NOSSOS PRODUTOS USAR DECLARAÇÕES ENGANOSAS OU FALSAS NA PUBLICIDADE OU RÓTULOS FAZER REIVINDICAÇÕES, INCLUINDO REIVINDICAÇÕES SOBRE EFEITOS À SAÚDE, OS NOSSOS PRODUTOS OU INGREDIENTES, SEM AS EVIDÊNCIAS ADEQUADAS E AUTORIZAÇÕES LEGAIS APROPRIADAS Certifique-se de que segue todos os processos de revisão e aprovação locais para conteúdo orientado para o consumidor~ Tem de ter especial cuidado no desenvolvimento de anúncios e na avaliação da programação que transmite mensagens para crianças com menos de 13 anos
PEPSICO	A.P4	Na PepsiCo, acreditamos que um sistema alimentar mais sustentável deve ir ao encontro das necessidades humanas, tanto para nutrição quanto para prazer. Trabalhamos continuamente para introduzir produtos compostos por mais ingredientes nutritivos necessários aos nossos consumidores. E continuamos a reformular as receitas existentes em todo o nosso portefólio para reduzir os açúcares adicionados, sódio e gorduras saturadas, para que os consumidores possam continuar a desfrutar das nossas marcas como parte de uma dieta equilibrada.
PEPSICO	A.P5	ESCOLHAS MELHORADAS NO NOSSO PORTEFÓLIO Há mais de 50 anos que a nossa predisposição para nos adaptarmo-nos permitiu transformar-nos de uma empresa que produzia apenas batatas e refrigerantes num líder global que oferece a ampla variedade de alimentos e bebidas mais agradáveis do mundo. Continuamos a expandir o nosso portefólio para oferecer mais opções para todos os gostos, estilos de vida e ocasiões.
PEPSICO	A.P6	O Nosso Objetivo: reduzir açúcares adicionados, sódio e gorduras saturadas. Reduzir Açúcares Adicionados: ≥ 67% do portefólio de bebidas terá ≤ 100 calorias de açúcares adicionados por 12oz. até 2025 Reduzir o Sódio: ≥ 75% do portefólio de alimentos não excederá as 1,3 miligramas de sódio por caloria até 2025 Reduzir as Gorduraa Saturadas: ≥ 75% do portefólio de alimentos não excederá as 1,1 gramas de gordura saturada por 100 calorias até 2025
PEPSICO	A.P7	O novo projecto que irá transformar os resíduos orgânicos da fábrica do Carregado em biogás implicará um investimento de 7.5 milhões de euros.
PEPSICO	A.P8	STEM TALENT GIRL Inspirar, educar e capacitar a próxima geração de mulheres líderes. Colaboramos com toda a nossa paixão com o programa STEM Talent Girl, pioneiro e cujo objetivo é aumentar o talento e promover a vocação para carreiras científicas e tecnológicas entre os alunos entre o 9º e o 12º ano de escolaridade. Graças a essa iniciativa, os alunos puderam conhecer em primeira pessoa como é a jornada de trabalho na fábrica de snacks da PepsiCo, com os seus mentores, todos profissionais qualificados e formados em carreiras STEM. Várias sessões de acompanhamento e mentoria foram realizadas ao longo do ano, com o objetivo de incutir a curiosidade para que não hesitem em desenvolver a sua vocação.
PEPSICO	A.P9	DONATIVOS PARA A WORLDREADER Tendo em conta o Dia Internacional da Mulher, contribuímos, juntamente com nossa equipa em escritórios e fábricas, com uma doação para a ONG Worldreader, uma fundação que visa trazer a leitura para todos, especialmente aqueles que estão numa situação de desigualdade, sendo as mulheres um dos grupos mais afetados, ultrapassando 2/3 da população total de analfabetos.
PEPSICO	A.P10	COMITÉ DE DIVERSIDADE A PepsiCo está comprometida em fornecer às pessoas com desigualdade de tratamento e oportunidades. Há oito anos, formámos um Comité de Diversidade na Europa, com o objetivo de partilhar atividades em diferentes mercados. A novidade deste 2019 é o posicionamento da Diversidade como uma prioridade, graças à criação de um novo Comité a nível local, e que desenvolvemos em colaboração com a Fundação Adecco. Mais especificamente, nos próximos anos vamos concentrarmo-nos em três grupos de pessoas com maior risco de exclusão devido a deficiência, sexo ou idade.
PEPSICO	A.P11	GRUPO EQUAL Em 28 de junho, são celebrados os distúrbios de Stonewall, que foi o ponto de virada dos movimentos de libertação do coletivo LGTBI. Desde então, a Pride é um dia em que comemoramos a luta por direitos e oportunidades iguais e na qual celebramos a diversidade. Esta é a primeira iniciativa que a nossa equipa EQUAL lançou, um grupo formado por colaboradores e tem como principal objetivo apoiar o coletivo LGTBI, garantindo um ambiente de trabalho agradável e inclusivo. Na nossa primeira edição do Pride organizamos uma série de palestras para oferecer

		informação, contexto e conscientização sobre o coletivo LGTBI com o objetivo de derrubar barreiras e preconceitos através do conhecimento e da empatia.
PEPSICO	A.P12	A estratégia de impacto hídrico da PepsiCo concentra-se na melhoria da gestão da água nos nossos negócios e nas regiões onde atuamos. O nosso objetivo final é apoiar a longo prazo a segurança da água sustentável para todos os seus utilizadores, especialmente em áreas de elevado risco de escassez de água.
PEPSICO	A.P13	A empresa participa neste movimento contra o desperdício alimentar pelo segundo ano consecutivo.

PRIMOR

Website	Code	CPA EXAMPLES
PRIMOR	A.PR1	Projetos de Investigação e Desenvolvimento A construção dos pilares da inovação também está sustentada na co-promoção de projetos de Investigação & Desenvolvimento, em parceria com as Entidades do Sistema Científico e Tecnológico, veículos para o novo conhecimento e reforço das equipas técnicas do Grupo Primor, através do acolhimento e integração de alunos/investigadores.
PRIMOR	A.PR2	Descrição: A Agenda VIIAFOOD, projeto apoiado ao abrigo do PRR (www.recuperarportugal.gov.pt), visa promover a transformação estrutural do setor Agroalimentar em linha com o propósito estabelecido no Pacto para a Competitividade e Internacionalização deste setor nacional. Para o efeito, a Agenda VIIAFOOD pretende criar uma plataforma nacional para o desenvolvimento e industrialização de produtos, processos e serviços inovadores, materializando-se num plano estratégico concertado, com medidas e ações conjuntas orientadas aos objetivos transversais do Setor. Com efeito, a Agenda VIIAFOOD pretende reforçar a colaboração entre as empresas da indústria agroalimentar, empresas de outras indústrias relacionadas e entidades de Investigação & Desenvolvimento para alavancar a criação de sinergias e a transferência de conhecimento e tecnologias de modo a (re)industrializar do Setor Agroalimentar Português.
PRIMOR	A.PR3	Designação do Projeto | cLabel+: Alimentos inovadores “clean label” naturais, nutritivos e orientados para o consumidor Número do Projeto | 46080 Tipologia da Operação | Sistema de Incentivos à Investigação e Desenvolvimento Tecnológico (SI I&DT) – Programas Mobiliadores Códigos da Operação | POCI-01-0247-FEDER-046080 | LISBOA-01-0247-FEDER-046080 Objetivo temático | OT 1 – Reforçar a investigação, o desenvolvimento tecnológico e a inovação Região de Intervenção | Norte, Centro, Lisboa, Alentejo Promotor líder | SUMOL+COMPAL MARCAS, S.A. Copromotores: PORTUGALFOODS – ASSOCIAÇÃO INTEGRALAR – INTERVENÇÃO DE EXCELÊNCIA NO SECTOR AGRO-ALIMENTAR; UM – UNIVERSIDADE DO MINHO; INIAV – INSTITUTO NACIONAL DE INVESTIGAÇÃO AGRÁRIA E VETERINÁRIA, I.P.; ISA – INSTITUTO SUPERIOR DE AGRONOMIA; INL – LABORATÓRIO IBÉRICO INTERNACIONAL DE NANOTECNOLOGIA (LIN); UNIVERSIDADE DE AVEIRO; SENSE TEST – SOCIEDADE DE ESTUDOS DE ANÁLISE SENSORIAL A PRODUTOS ALIMENTARES LDA; MENDES GONÇALVES, S.A.; UNIVERSIDADE DE COIMBRA; WEDOTECH – COMPANHIA DE IDEIAS E DE TECNOLOGIAS LDA; VIEIRA DE CASTRO PRODUTOS ALIMENTARES, S.A.; REQUIMTE – REDE DE QUIMICA E DE TECNOLOGIA – ASSOCIAÇÃO; UTAD – UNIVERSIDADE DE TRAS OS MONTES E ALTO DOURO; UNIVERSIDADE DO PORTO; FRULACT – INDÚSTRIA AGRO-ALIMENTAR, S.A.; UNIVERSIDADE CATÓLICA PORTUGUESA; UNIVERSIDADE NOVA DE LISBOA; PRIMOR CHARCUTARIA – PRIMA, S.A.; SPI – SOCIEDADE PORTUGUESA DE INOVAÇÃO – CONSULTADORIA EMPRESARIAL E FOMENTO DA INOVAÇÃO S.A. Data de aprovação | 23.11.2020 Data de início | 01.06.2020 Data de conclusão | 31.05.2023 Investimento total elegível | 4.641.020,43 euros Apoio financeiro da União Europeia através do FEDER | 3.106.642,43 euros Programa financiador | Programa Operacional Competitividade e Internacionalização; Programa Operacional Regional de Lisboa Breve Descrição do Projeto | O projeto cLabel+ tem como principal objetivo responder aos desafios que o consumidor coloca à indústria alimentar e pretende desenvolver processos e produtos que sejam entendidos pelo consumidor como consistentes com produtos com ingredientes naturais, com limitada utilização de um número elevado de aditivos, e que permitam, pelas suas propriedades físicas, químicas e nutricionais, proporcionar a experiência esperada, mantendo a segurança alimentar. Para o efeito, o projeto cLabel+ integra um conjunto de projetos parciais (PPS) complementares que se integram na realização do objetivo global, nomeadamente: Valorização nutricional de produtos através dos macronutrientes e das condições de processamento (A.PPS1); Soluções inovadoras e “clean label” para a redução do açúcar e modelação do poder adoçante em alimentos (A.PPS2); Estabilização e conservação “clean label” de molhos, produtos cárneos e confeitaria (A.PPS3); Novas metodologias para a integração e otimização da resposta do consumidor (A.PPS4); Gestão do projeto, disseminação e valorização de resultados (A.PPS5).
PRIMOR	A.PR4	Designação do projeto | MOBFOOD – Mobilização de Conhecimento Científico e Tecnológico em Resposta aos Desafios do Mercado Agroalimentar Código do projeto | 24524 Tipologia da operação | Sistema de Incentivos à Investigação e Desenvolvimento Tecnológico – Programas Mobilizadores Código da operação | POCI-01-0247-FEDER-024524 Objetivo temático | OT 1 – Reforçar a investigação, o desenvolvimento tecnológico e a inovação Região de intervenção | Norte, Centro, Lisboa, Alentejo Promotor líder | PRIMOR CHARCUTARIA – PRIMA, S.A. Copromotores: Fábrica de Conservas “A Poveira”, S.A. A.BLC3 Evolution, Lda. Cerealis Produtos Alimentares S.A. FoodinTech Lda. ICM – Indústrias de Carnes do Minho, S.A. I.T.S. – Indústria Transformadora de Subprodutos S.A. Olano Logistica de Frio, Lda. Sense Test – Sociedade de Estudos de Análise Sensorial a Produtos Alimentares, Lda. Univeg Logistics Portugal – Logística e Transportes S.A. Vizelpas – Comércio de Artigos Plásticos, Lda. CIIMAR – Centro Interdisciplinar de Investigação Marinha e Ambiental Faculdade de Ciências da Universidade do Porto Instituto de Biologia Experimental e Tecnológica Instituto Nacional de Saúde Doutor Ricardo Jorge, I.P. Instituto Politécnico de Coimbra Instituto Politécnico de Leiria Instituto Politécnico de Viseu Instituto Superior de Agronomia Pólo de Inovação em Engenharia de Polímeros Universidade de Aveiro Universidade do Minho NOVA Medical School | Faculdade de Ciências Médicas da Universidade Nova de Lisboa Fromageries Bel Portugal Matadouro Central de Entre Douro e Minho S.A. Decorgel – Produtos Alimentares S.A. Frulact – Indústria Agro Alimentar S.A. INOVA+ – Innovation Services, S.A. Mendes Gonçalves, S.A. Sebol – Comércio e Indústria de Sebo S.A. Sonae Center Serviços II, S.A. Vitacress Portugal, S.A. CATAA – Associação Centro de Apoio Tecnológico Agroalimentar Universidade Católica Portuguesa Faculdade de Engenharia da Universidade do Porto Laboratório Ibérico Internacional de Nanotecnologia Instituto Politécnico de Bragança Instituto Politécnico de Castelo Branco Instituto Politécnico de Santarém Instituto Politécnico de Viana do Castelo Instituto Superior Técnico INTEGRALAR – Intervenção de Excelência no sector Agro-Alimentar Universidade de Coimbra Faculdade de Ciências e Tecnologia da Universidade Nova de Lisboa Universidade de Trás-os-Montes e Alto Douro Data de aprovação | 24.07.2017 Data de início | 01.12.2017 Data de conclusão | 31.05.2021 Custo total elegível | 7.021.739,21 € Apoio financeiro da União Europeia |

		4.547.393,15 € Programa financiador | Programa Operacional Competitividade e Internacionalização (POCI), Programa Operacional Lisboa (PO Lisboa) Breve descrição do projecto | O projeto MOBFOOD tem como objetivo responder aos desafios relacionados com a promoção de uma indústria alimentar nacional mais competitiva. Passará pela promoção de novas estratégias de crescimento baseadas no reforço da capacidade tecnológica, de inovação e de I&D orientadas à obtenção de novos produtos, serviços, processos ou tecnologias, atuando ao longo de toda a cadeia de valor e reforçando a colaboração entre o setor empresarial e não empresarial. Pretende-se que o setor alimentar seja sustentável, totalmente integrado, interligado, transparente, resiliente, seguro, eficiente na utilização dos recursos e centrado no consumidor. Pretende-se alcançar estes objetivos através da implementação de soluções em três pilares principais: "Segurança Alimentar e Sustentabilidade", "Alimentação para a Saúde e Bem-estar" e "Alimentos Seguros e Qualidade", materializando-se na investigação e desenvolvimento de novos processos, produtos ou serviços. Estrutura do projeto | PPS 2. Resíduos e Utilização Eficiente de Recursos | PPS 3. Embalagens sustentáveis com propriedades ativas para aplicações alimentares |PPS 4. Nutrição, Saúde e Bem-Estar | PPS 5. Qualidade e Segurança Alimentar |PPS 6. Autenticidade e rastreabilidade de produtos frutícolas DOP (Pera Rocha do Oeste) e IGP (Maçã de Alcobaça var. Golden Delicious) e queijo DOP (Serra da Estrela) | PPS 7. Logística – Cadeia Logística Agroalimentar Sustentável Colaborativa | PPS 8. Consumidor – Novas Tecnologias de Avaliação | PPS 9. Coordenação de Projeto, Promoção, Disseminação e Exploração de Resultados
PRIMOR	A.PR5	Academia Formar pessoas, vencer no Futuro. O Grupo Primor privilegia o contacto permanente com o mundo académico ou cientíDco-tecnológico, partilhando continuamente experiências e conhecimento, participando ou promovendo projetos de investigação, ou através do acolhimento de universidades e alunos nas suas unidades produtivas. A própria integração de investigadores nos seus quadros tem sido um pilar de desenvolvimento de conhecimento dentro do Grupo Primor.
PRIMOR	A.PR6	A 1ª Gama de produtos de Charcutaria Clean Label Na Primor, estamos atentos às tendências dos consumidores, nomeadamente às suas preocupações com a saúde e bem-estar e atenção prestada à composição dos produtos que consomem. A procura por soluções mais simples, naturais e sem aditivos artificiais veio para ficar! Estas são as razões que nos levaram ao desenvolvimento da nossa primeira linha CLEAN LABEL, um conjunto de produtos mais equilibrados e próximos da sua verdadeira essência. Experimente todo o sabor e prazer da melhor experiência de charcutaria em estado Puro
PRIMOR	A.PR7	A Primor-Charcutaria é eleita novamente Nº1 na "Escolha do Consumidor" em 2022
PRIMOR	A.PR8	O bem-estar animal é uma das principais preocupações do Grupo Primor, defendendo o respeito pelas cinco liberdades dos animais em todas as fases da sua vida: o animal deve estar livre de fome e sede, livre de desconforto, livre de dor, lesões ou doença, livre para expressar os seus comportamentos normais e livre de medo e aFição.
PRIMOR	A.PR9	O Grupo Primor compromete-se a valorizar e a desenvolver uma organização sustentável a longo prazo de forma a adicionar valor a clientes, consumidores, parceiros e acionistas. O modo como o Grupo tem vindo a evoluir, aprendendo com o passado e de olhos postos no futuro, reFete esta aspiração, orientadora de todas as suas atividades.
PRIMOR	A.PR10	Patrocínio Congresso Alimentação e Nutrição 2023
ESB	A.ESB1	A Escola Superior de Biotecnologia desenvolve intercâmbios com múltiplas instituições dentro e fora de Portugal, incluindo centros de investigação, universidades, empresas e unidades de saúde: Modelo Continente Hipermercados, Bimbo, Danone Portugal, Imperial Produtos Alimentares, Nestlé Portugal, Primor Charcutaria Prima, Sonae, Cerealis, Vitacress Portugal

SONAE

Website	Code	CPA EXAMPLES
SONAE	A.S1	A Missão Continente junta-se à Fundação Portuguesa de Cardiologia no evento "Maio, Mês do Coração", para sensibilizar a população para a importância de um estilo de vida saudável, sobretudo através da alimentação. O webinar é de acesso livre e gratuito e irá decorrer dia 27 de maio, a partir das 18h na Plataforma Doenças Cardiovasculares, em maionocoracao.pt.
SONAE	A.S2	Presentes à Mesa. Missão Continente. PARCEIROS E APOIOS INSTITUCIONAIS A campanha tem o apoio da APN – Associação Portuguesa de Nutrição e da ENSP - Escola Nacional de Saúde Pública, e conta ainda com parceiros como a TVI e a Federação Portuguesa de Futebol.
SONAE	A.S3	O Programa Educativo "Escola Missão Continente", criado em 2016, pretende impactar os alunos do 1º ciclo do Ensino Básico (CEB), professores, pais e toda a comunidade escolar, sensibilizando os mais novos para uma alimentação e estilos de vida saudáveis e educação para o consumo consciente e sustentável. Ao longo do ano letivo proporciona conteúdos, atividades educativas, desafios (com prémios associados), materiais lúdicos pedagógicos e visitas no terreno. A Escola Missão Continente tem como parceiros institucionais a Direção Geral da Saúde, o CEIDSS – Centro de Estudos e Investigação em Dinâmicas Sociais e Saúde, o Centro Hospitalar Universitário de São João (CHUSJ), o Instituto de Ciências Biomédicas Abel Salazar da Universidade do Porto e a Federação Portuguesa de Futebol (FPF) e ainda o apoio institucional da Direção-Geral da Educação.
SONAE	A.S4	Promovido pela Missão Continente, em parceria com o Instituto de Ciências Sociais da Universidade de Lisboa, o III Grande Inquérito Nacional de Sustentabilidade dá continuidade aos dois inquéritos anteriores. Nesta edição, com um foco menos generalizado, dá-se especial atenção aos estilos de vida e transições sócio-ecológicas, cívicas e económicas no contexto pós-pandémico.
SONAE	A.S5	O Presidente da República Portuguesa, Marcelo Rebelo Sousa, deixou uma mensagem de reconhecimento da importância do III Grande Inquérito Nacional de Sustentabilidade para visão sobre o presente e futuro da sustentabilidade em Portugal.
SONAE	A.S6	Ao longo de 4 edições, a Conferência Portugal Saudável juntou figuras relevantes da academia, da investigação, da política, da saúde, do retalho e da comunicação, para, em conjunto, enriquecerem o debate público sobre sistemas de alimentação sustentáveis capazes de conciliar os diferentes setores
SONAE	A.S7	O Conselho Estratégico Missão Continente é composto por representantes de várias entidades, públicas e privadas, e tem por propósito o levantamento e debate de propostas de intervenção social que contribuam, efetivamente, para melhorar a vida das pessoas e comunidades. Com base no contexto social e económico do país, todos os anos são atualizadas estratégias e iniciativas que ajudam a fortalecer a resposta solidária da Missão Continente nas diversas áreas onde atua.
SONAE	A.S8	A Missão Continente colabora com empresas de diferentes setores, entidades governamentais, ONGs, associações e universidades em torno de uma visão comum – uma economia circular para os plásticos em Portugal. O Pacto Português para os Plásticos tem como missão preservar o seu valor económico e social e garantir que os plásticos nunca terminam

		na natureza. "Vamos Reinventar o Plástico" é o mote da campanha do Pacto Português para os Plásticos, que pretende sensibilizar os consumidores portugueses para uma utilização responsável do plástico. O objetivo é mobilizar a sociedade no processo de transição para uma economia circular dos plásticos em Portugal. Depois do envolvimento de empresas de diferentes setores, entidades governamentais, ONGs, associações e universidades, chegou o momento de envolver também os cidadãos na missão de solucionar os problemas associados ao plástico. O Continente, além de membro signatário do pacto, foi um dos grandes impulsionadores da constituição do mesmo, na sequência da sua adesão ao Global Commitment - New Plastics Economy da Fundação Ellen MacArthur. Foi o primeiro retalhista português a assinar este pacto.
SONAE	A.S9	Conselho Estratégico Parceiros institucionais • Agência 42 Lisboa • Agência Nacional para a Qualificação e o Ensino Profissional • Agência para a Competitividade e Inovação • Associação Portuguesa de Empresas de Distribuição • Associação Portuguesa de Nutrição • Comissão para a Cidadania e Igualdade de Género • Cruz Vermelha Portuguesa • Direção-Geral da Educação • Escola Nacional de Saúde Pública • • Federação Portuguesa de Futebol Federação Portuguesa dos Bancos Alimentares Contra a Fome • • Instituto de Ciências Sociais Instituto Superior de Agronomia da Universidade de Lisboa • Portugal Inovação Social • Quercus • Refood • RUTIS – Rede de Universidades Seniores • Sociedade Portuguesa de Pediatria • Casa do Impacto • Centro de Estudos e Investigação em Dinâmicas Sociais e Saúde • Centro Materno Infantil do Norte • Confederação Nacional das Instituições de Solidariedade • • • • • Direção Geral de Alimentação e Veterinária Direção Geral da Saúde Entrajuda European Venture Philanthropy Association Federação das Indústrias Portuguesas Agroalimentares • GRACE - Empresas Responsáveis • Hospital de São João do Porto • • Instituto Ciências Biomédicas Abel Salazar Ordem dos Enfermeiros • Programa Nacional para a Promoção da Alimentação Saudável • União das Misericórdias Portuguesas • Unidades de Saúde Familiar – Associação Nacional
SONAE	A.S10	Composto por 18 personalidades especialistas e influentes em diversos setores, o Conselho Estratégico da Missão Continente tem como objetivo acompanhar e fomentar o debate sobre temas relacionados com a sua área de atuação. Permitindo, desta forma, a consistência no trabalho anual entre a Missão Continente e os parceiros sociais. Conselho Estratégico Filipa Henrique de Jesus, Presidente da Agência Nacional para a Qualificação e o Ensino Profissional Gonçalo Lobo Xavier, Presidente da Associação Portuguesa de Empresas de Distribuição Célia Craveiro, Presidente da Associação Portuguesa de Nutrição Sandra Ribeiro, Presidente da Comissão para a Cidadania e a Igualdade de Género José Vitor Pedroso, Diretor-Geral da Educação Sónia Dias, Diretora da Escola Nacional de Saúde Pública Isabel Jonet, Presidente da Federação Portuguesa dos Bancos Alimentares Contra a Fome Fernando Gomes, Presidente da Federação Portuguesa de Futebol Karin Wall, Diretora do Instituto de Ciências Sociais António Guerreiro de Brito, Presidente do Instituto Superior de Agronomia Filipe Almeida, Presidente da Estrutura Missão Portugal Inovação Social Alexandra Azevedo, Presidente da Quercus Hunter Halder, Presidente da Re-food Luís Jacob, Presidente da Rede de Universidades Seniores Inês Azevedo, Presidente da Sociedade Portuguesa de Pediatria Ana Jorge, Presidente da Cruz Vermelha Portuguesa Francisco Sá, Presidente do IAPMEI Pedro Santa Clara, Diretor da 42 Lisboa
SONAE	A.S11	Para o bem de todos, o melhor de cada um A campanha Todos por Todos, em parceria com a TVI, Federação Portuguesa de Futebol e LAPS Foundation, teve como missão ajudar na resposta social nacional em pleno período de pandemia, altura em que os pedidos de ajuda às instituições de apoio social aumentaram e o sentido de entreajuda foi fundamental.
SONAE	A.S12	Ao longo do ano, a Missão Continente associou-se diversas entidades e iniciativas para estar ao lado de quem mais precisa. Foi parceira alimentar na campanha "Unidos por Portugal" da Federação Portuguesa de Futebol e da SIC Esperança deu apoio a 334 instituições sociais. Em associação à Secretaria de Estado para a Cidadania e Igualdade, foi desenvolvida a campanha de prevenção contra violência doméstica e tráfico de seres humanos. Cerca de 50 estruturas de resposta e acolhimento para vítimas de violência doméstica foram apoiadas pela Missão Continente com doação de bens alimentares e higiene pessoal.
SONAE	A.S13	A Dieta Mediterrânica, considerada um padrão de referência alimentar completo e equilibrado, é um dos pontos em destaque, ao qual se junta a redução de sal, açúcar e gorduras: aspetos fundamentais para a promoção da saúde. Convidados/Oradores Ana Alves, diretora comercial de Marcas Próprias Continente Mayumi Delgado, nutricionista e responsável pela equipa de Nutrição Continente Luís Negrão, médico de Saúde Pública e assessor médico da Fundação Portuguesa de Cardiologia Para assistir a este webinar basta clicar aqui.
SONAE	A.S14	Promover a alimentação e estilo de vida saudáveis Mudar mentalidades Continuamos no caminho da promoção da alimentação e do estilo de vida saudáveis, por isso, encontrámos alternativas para chegar a mensagem às nossas crianças em idade escolar, mesmo quando a pandemia as manteve em casa. E porque sabemos o quanto é importante a redução dos açucares e do sal na alimentação, estamos a diminuir a utilização destes ingredientes nos nossos produtos de marca própria.
SONAE	A.S15	Prevenir a obesidade infantil Para medir a ação do Programa nas crianças que nele participam, a "Escola Missão Continente" vai avaliar, pela primeira vez, o impacto deste programa educativo para uma alimentação saudável, em 35 escolas do 1º CEB, durante 4 anos consecutivos (2021-2025). É o primeiro estudo num programa de nutrição e saúde comunitária, com esta dimensão, em Portugal. O estudo científico de medição de impacto acompanhará crianças do 1º ciclo do Ensino Básico (CEB), inscritas no programa, abordando a temática geral "Dieta Mediterrânica - uma opção saudável, sustentável e consciente", onde a cada ano se aplicará um programa específico com conteúdos integrados, ajustados às idades das crianças. Todas as turmas inscritas no Programa vão abordar 4 temas principais ao longo dos 4 anos de acompanhamento.
SONAE	A.S16	Trata-se de um dos primeiros estudos envolvendo um programa de nutrição e saúde comunitária com a dimensão nacional que incluirá 40 turmas de 35 escolas do 1º CEB, representando os 18 Distritos de Portugal Continental incluindo ainda 2 da Madeira e dos Açores. Este estudo pretende ainda contrariar o provável impacto da pandemia por COVID-19 no progresso da obesidade infantil em Portugal, onde 1 em cada 3 crianças apresenta excesso de peso. Ana Rito, Presidente da Direção do Centro de Estudos e Investigação em Dinâmicas Sociais e Saúde (CEIDSS) e Investigadora do Instituto Nacional de Saúde Doutor Ricardo Jorge (INSA)
SONAE	A.S17	Este estudo aprofunda temáticas como a produção e consumo, a literacia funcional (alimentar, digital, sanitária, etc.), o desperdício e destino dos resíduos urbanos, a participação cívica, as perspectivas de futuro, e a vulnerabilidade e resiliência dos portugueses face a riscos globais. Foi realizado através de um questionário, baseado num inquérito estruturado de acordo com uma amostragem de 1.520 cidadãos residentes em Portugal Continental, com idade superior a 18 anos.
SONAE	A.S18	A criação de conhecimento e a sensibilização para atitudes conscientes que permitam construir, em conjunto, um sistema alimentar e uma sociedade mais saudável e sustentável são os pilares desta iniciativa desenvolvida pela Missão Continente. Esta conferência é aberta a todos e totalmente gratuita, pois consideramos que o acesso e partilha de

		informação, a interação dos diferentes setores, assim como a literacia alimentar podem resolver problemas como a fome, a má nutrição e o desperdício alimentar.
SONAE	A.S19	nutricionista online O Nutricionista Online é uma funcionalidade onde pode esclarecer as suas dúvidas sobre alimentação ou sobre os impactos na saúde daquilo que come. Afinal, só queremos estar mais perto de si e cumprir a nossa missão de o ajudar a viver com mais qualidade de vida. Por trás de um grande projeto, há sempre grandes pessoas. Está na altura de as conhecer.
SONAE	A.S20	Missão Geração Saudável A Missão Geração Saudável tem como objetivo estimular as gerações mais jovens a terem comportamentos e hábitos que promovam vidas mais saudáveis, através de 3 temas principais: Alimentação Saudável Estilo de Vida Ativo Consumo Consciente
SONAE	A.S21	Há mais de uma década que o Continente foi pioneiro em Portugal ao adotar este sistema de informação nutricional nos seus produtos de marca própria. A Missão Continente revolucionou, assim, a leitura dos produtos. Indo ao encontro de um consumidor cada vez mais atento e exigente, o Semáforo Nutricional representa hoje, para muitos dos clientes, um critério decisivo no momento da compra.
SONAE	A.S22	Como interpretar o rótulo dos produtos marca Continente? No nosso Semáforo Nutricional, os círculos vermelhos, amarelos ou verdes estão localizados em evidência, na frente das embalagens. As percentagens no interior dos círculos correspondem à proporção do Valor Diário de Referência (VDR) que o produto contém de um determinado nutriente. O VDR é um guia que ajuda o consumidor a ingerir diariamente a quantidade de nutrientes e calorias adequadas. As percentagens do VDR dizem-nos qual o contributo da dose do alimento para a satisfação das nossas necessidades diárias (em termos de calorias e dos principais nutrientes).
SONAE	A.S23	Nutrição Saiba mais sobre os elementos nutritivos de cada ingrediente ou alimento.
SONAE	A.S24	Saiba mais sobre nutrição Fique a par sobre curiosidades, o que pode fazer para melhorar e o que deve evitar
SONAE	A.S25	Saiba como a literacia alimentar é importante para a promoção da alimentação saudável e aumento da qualidade de vida! Dicas De Nutrição Alimentação Saudável Atualizado a 26/01/2022 Escolhas Saudáveis Continente Magazine Vitor de Sá Martins Nutricionista Célula 0997N Com o objetivo de capacitar e aumentar a literacia alimentar, a Diaverum, em parceria com o Continente e outros parceiros, preparou um livro de saborosas receitas adaptadas especialmente para pessoas com doença renal crónica em hemodiálise, mas que poderão ser partilhadas pelo resto da família e dos amigos. No livro "Bons Momentos - Receitas Saborosas para Todos, Especialmente para Pessoas em Diálise" , receitas tradicionais, como o pastel de nata , a açorda de bacalhau , as pataniscas e as rabanadas /fatias douradas, foram ajustadas de acordo com as indicações nutricionais necessárias, para que o prazer, a tradição e a partilha se mantenham e esta doença não seja uma razão para não ter uma vida plena e com qualidade. Este livro está disponível para todas pessoas que tratamos e através do site www.diaverum.pt . Deixamos-lhe aqui uma das receitas que pode encontrar ao longo das suas páginas.
SONAE	A.S26	O que comer antes e depois do treino? Por À Roda da Alimentação
SONAE	A.S27	Alimentação na gravidez Por À Roda da Alimentação
SONAE	A.S28	O movimento À Roda da Alimentação é uma iniciativa do Continente e dos seus especialistas, que nasceu da vontade de inspirar uma mudança de hábitos e de estilos de vida, para ajudar a melhorar a vida de todos os portugueses. Por isso, não vamos parar. Vamos continuar a espalhar este movimento de mudança, partilhando tudo o que já aprendemos e todas as novas descobertas que estão para vir: receitas e hábitos alimentares saudáveis, dicas para reduzir o desperdício e soluções para tornar a sua vida mais ativa e mais sustentável. Para além disso, vamos continuar a trazer-lhe histórias inspiradoras através de novos participantes e especialistas que trabalham na promoção da vida saudável, para que conheça novas perspetivas, experiências e saberes. No fundo, tudo o que precisa de saber para dar uma volta positiva à sua vida.
SONAE	A.S29	Sabia que no À Roda da Alimentação contamos com uma equipa de nutricionistas que assegura o rigor dos nossos conteúdos? Está na altura de os conhecer, saber quem são e o que fazem. Tudo por uma vida mais saudável.
SONAE	A.S30	A Importância do nutricionista À Roda da alimentação está a transformar mentalidades. Este movimento já alterou e inspirou muitos hábitos alimentares dos portugueses graças aos artigos, às receitas e às dicas que publica sobre alimentação saudável e questões de saúde. O que talvez não saiba é que há uma excelente equipa de nutricionistas que trabalha na sombra para validar o rigor dos nossos conteúdos. É isso que nos distingue. Quando o tema é alimentação, é bom ter acesso a informação credível e validada. E esta equipa, além de ensinar a comer e melhorar o nosso estado de saúde, disponibiliza ferramentas necessárias para estarmos mais informados em relação às nossas escolhas alimentares, tornandoas mais conscientes e responsáveis. É o caso do Nutricionista Online, especificamente dedicada a esclarecer dúvidas ou a responder a qualquer questão sobre a alimentação e o seu impacto na saúde.
SONAE	A.S31	Nutricionista online: Últimas perguntas Papas de aveia engordam? Como baixar o percentual de massa gorda? Qual a alimentação recomendada para doentes com asma? A moda do sem glúten aplica-se a quem? Como saber se somos intolerantes ao glúten? Os cereais de pequeno almoço engordam? Como reduzir o consumo de sal sem perder o sabor dos alimentos? Perguntas populares Há gorduras boas e más? Que gorduras devo incluir na alimentação e que cuidados devo ter? Como reduzir calorias na alimentação? Que cuidados devo ter? Que tipos de açúcar existem? Que cuidados devo ter? É verdade que comer à noite engorda? O que se deve comer ao jantar? Não jantar faz mal? Quais as principais diferenças na alimentação de um adulto, de um idoso ou de uma criança? Que cuidados com a alimentação no desporto existem?
SONAE	A.S32	Promover uma Alimentação Saudável Comer brócolos nunca foi tão fácil e divertido! No Programa Escola Missão Continente, procuramos promover uma alimentação saudável junto dos mais novos através de iniciativas didáticas e educativas. Ao longo do Programa, abordamos temáticas como: a importância do consumo regular de frutas e legumes, a necessidade de reduzir a ingestão de sal e açúcares, entre outros. Desta forma, pretendemos desenvolver bons hábitos alimentares com escolhas diárias que contribuam para uma vida saudável. Uma alimentação variada e equilibrada é fundamental para a criação de gerações mais saudáveis e felizes
SONAE	A.S33	Reduções de açúcar e sal nos produtos de Marca Própria Cortámos 1.185 toneladas de açúcar e sal nos nossos produtos O Continente tem vindo a desenvolver planos de otimização nutricional em mais de 350 produtos de marca própria, tendo em vista a redução do açúçar, sal e gorduras, contribuindo desta forma para a saúde de todos. As reduções incidem sobre produtos de marca própria, com destaque para iogurtes, cereais, bolachas, refrigerantes, batatas fritas, polpas de tomate e sopas de take away, contribuindo desta forma para melhorar a saúde e estilo de vida dos portugueses. Entre outros exemplos, as leguminosas em conserva garantem baixo teor de sal e as polpas de tomate não têm qualquer sal adicionado. A marca eliminou ainda o óleo de palma a 80 dos seus produtos.

SONAE	A.S34	Reduções anuais de Açúcar, Sal e Gorduras nos produtos de marca Continente -120 ton -800 ton redução de açúcares redução de sal -385 ton redução de gorduras saturadas Até ao final de 2022, estima-se uma redução de sal nas sopas Continente de cerca de 4 toneladas, uma redução de 12% do teor de sal em batatas fritas e snacks e uma redução de 10% do teor de açúcar em iogurtes e leites com chocolate. Para Mayumi Delgado, responsável pela equipa de Nutrição do Continente, "são reduções importantes que o Continente faz em prol da saúde dos portugueses, ajudando à prevenção da obesidade, mas também das doenças associadas, como doenças cardiovasculares, diabetes tipo 2, e muitas outras. Dois em cada três portugueses têm excesso de peso ou obesidade e o problema agravou-se com a pandemia. Nesse sentido, a equipa de nutrição do Continente tem acelerado a otimização nutricional para ajudar a inverter esta situação". Paralelamente, o Continente aposta no Semáforo Nutricional na frente das embalagens, para que o consumidor tenha acesso a informação simplificada sobre os produtos, principalmente sobre os nutrientes que, em excesso, podem ser um problema grave para a saúde. Através de um código de cores, o semáforo mostra a concentração de açúcar, sal, gorduras e gorduras saturadas (verde – baixa, amarelo –moderada e vermelho – elevada). Uma dica essencial para escolhas equilibradas é optar mais vezes por mais verdes ou amarelos e menos por 'sinais vermelhos'. As reduções são graduais para não impactar o sabor dos produtos, que garantimos com provas sensoriais junto dos consumidores, que testam e avaliam os novos produtos Mayumi Delgado, Responsável pela equipa de Nutrição do Continente
SONAE	A.S35	Há mais de 10 anos, a criação do Semáforo Nutricional nos produtos Continente abriu caminho a uma escolha mais transparente e informada dos nossos clientes. Com uma preocupação crescente pelos bons hábitos alimentares, o consumidor torna-se mais exigente nas suas escolhas. Reduzir no sal, no açúcar ou nas gorduras, adotar hábitos alimentares saudáveis e reorientar o sistema alimentar a partir da disponibilização de alimentos mais equilibrados a preços acessíveis, é algo que continuamos a considerar urgente.
SONAE	A.S36	À Roda da Alimentação Com Catarina Furtado Série 1 48 Episódios
SONAE	A.S37	A Missão Continente tem como compromisso a Sustentabilidade, contribuindo positivamente para as comunidades em que se insere e atuando em três eixos: Alimentação, Pessoas e Planeta.
SONAE	A.S38	Ao longo dos últimos anos, seja através das suas próprias iniciativas, seja através da participação em campanhas e projetos externos, a Missão Continente tem alinhado a sua atuação com a agenda do Desenvolvimento Sustentável. Procuramos desenvolver um futuro mais justo, feliz e sustentável, assente em três grandes eixos de atuação – Alimentação, Pessoas e Planeta.
SONAE	A.S39	O Impacto da Missão Continente por áreas de atuação A Missão Continente mantém-se empenhada na prossecução dos seus objetivos em prol de uma sociedade mais equilibrada e um futuro sustentável. Inclusão Social Saúde 12.492 beneficiários. 4.020 consultas prestadas. 2.499 visitas ao domicílio. 26 voluntários envolvidos. 3 viaturas adquiridas. 6.210 beneficiários. 25.676 de visitas ao domiciliário. 700 voluntário envolvidos. Educação e Sensibilização 70.704 alunos beneficiários. 655 escolas. 5.110 professores. 6.789 pessoas sensibilizadas. Os grandes números de 2022 + de 70.000 alunos envolvidos no programa Escola Missão Continente no ano lectivo 2021/22 (+74% face a 2020/21) 1.200.000€ doados a 11 instituições que combatem a solidão e isolamento social + de 320.000€ doados a 5 instituições no âmbito da campanha Sacos Solidários (Liga Portuguesa Contra o Cancro; Fundação do Gil; Ajudar Moçambique; CASA e APAV). 1.600.000€ doados ao Fundo da Cruz Vermelha Portuguesa, criado para apoiar os refugiados provenientes da Ucrânia. 29.800.000€ doados a 1.076 instituições, em excedentes alimentares das lojas e entrepostos. + de 2.350.000 angariados em vales solidários para campanhas Banco Alimentar Contra a Fome, Animalife e Cruz Vermelha Portuguesa
SONAE	A.S40	Missão em números Em 2021 continuámos a agilizar grandes iniciativas e a pensar em novas formas de ajudar as comunidades. Chegámos aos mais vulneráveis "Luzes com Presença" 1.200.000 € Apoio Covid-19 angariados para apoiar 11 instituições que combatem a solidão e o isolamento social 30.000 50 ton bens alimentares, de cuidado e higiene pessoal e refeições doados a utentes e profissionais de saúde de bens alimentares doados à REA para apoiar 450 mil pessoas em situação de vulnerabilidade Realizámos recolhas de bens e campanhas de vales Cruz Vermelha Portuguesa Banco Alimentar Contra a Fome 501.778 € em vales angariados 385.050 € em vales angariados 464 ton de bens alimentares angariados A MISSÃO EM NÚMEROS Animalife - Banco Solidário Animal 253.696 € em vales angariados 209 ton de bens alimentares angariados
SONAE	A.S41	Doámos excedentes alimentares das nossas lojas 19,2 M€ em excedentes alimentares doados 1.448 instituições sociais e bem-estar animal apoiadas Apoiámos 284 iniciativas locais com mais de 385.000€ Lançámos os Heróis Missão Continente, com 2.177 pessoas registadas e 13 missões para descobrir.
SONAE	A.S42	Na Missão Continente prosseguimos empenhados em prol de uma sociedade mais equilibrada e de um futuro sustentável. Incentivar uma alimentação e estilo de vida saudáveis, consciencializando para o consumo responsável Agir em conjunto com as comunidades, ajudando-as a crescer, serem mais sustentáveis e responder aos desafios sociais e ambientais Promover a economia circular e as melhores práticas ambientais, reduzindo o desperdício alimentar e incentivando o uso responsável do plástico Investir em projetos de impacto social que transformem e melhorem a vida de pessoas em contexto de vulnerabilidade
SONAE	A.S43	Sensibilizar para o consumo consciente Menos desperdício Anualmente, milhões de toneladas de alimentos são desperdiçados globalmente, com implicações graves, quer a nível social como ambiental. Cientes da importância de reduzir o desperdício alimentar, temos vindo a desenvolver, ano após ano, mecanismos e ações concretas que visam promover o consumo consciente, com ganhos claros quer para os consumidores, como para os nossos produtores nacionais.
SONAE	A.S44	Redução do Desperdício Alimentar Unidos contra o desperdício "Perda de alimentos" é a diminuição da quantidade e da qualidade dos produtos agrícolas, silvícolas e da pesca destinados ao consumo humano e que, em última análise, não são consumidos pelas pessoas. As perdas de alimentos ocorrem ao longo da cadeia de abastecimento, da produção à colheita e ao manuseamento pós-colheita, do armazenamento à transformação e durante o transporte. Fonte: Food and Agriculture Organization of the United Nations. • Estima-se que 1/3 de todos os alimentos produzidos globalmente se perdem ou vão para o lixo… aproximadamente 1,6 mil milhões de toneladas por ano. • O que significa que são desperdiçadas 51 toneladas de comida por segundo. • Cerca de 870 milhões de pessoas em todo o mundo estão subnutridas. • Representa 8% das emissões globais de gases de efeito estufa (GEE). • Estima-se que a perda anual de alimentos e resíduos atingirá 2,1 mil milhões de toneladas no valor de 1,5 triliões de dólares. Mobilizados contra o desperdício alimentar e atendendo à posição que ocupamos na cadeia de valor, temos vindo a dinamizar parcerias na cadeia de abastecimento que permitem desenvolver importantes projetos de inovação e circularidade e de combate ao desperdício gerado. Ao nível das nossas operações, temos vindo a desenvolver um conjunto de mecanismos que nos permitem acelerar o escoamento de produtos e do programa de doação de excedentes alimentares junto da Comunidade e dos nossos Colaboradores. Estas iniciativas permitiram evitar mais de 37 M€ de desperdício em 2021

SONAE	A.S45	Caixas de frutas e legumes Zer0% Desperdício Com o objetivo de prevenir a quebra, o Continente desenvolveu caixas de 5 Kg com frutas e legumes que estão perto de ultrapassar o ponto ótimo de consumo. Etiquetas cor-de-rosa Estratégia implementada há mais de 10 anos nas lojas Continente, as etiquetas cor-de-rosa são rótulos inteligentes de depreciação que comunicam uma redução de preço em produtos que se aproximam do fim do prazo de validade. Um sistema que evita o desperdício e oferece, simultaneamente, benefício económico ao cliente. Doação de excedentes alimentares Faz parte do dia a dia das lojas Continente o reaproveitamento e redistribuição de bens alimentares: • Junto de instituições de solidariedade social e associações de apoio a animais; • Junto de colaboradores nas áreas sociais das lojas e entrepostos.
SONAE	A.S46	Dinamização de parcerias na cadeia de valor O Vinagre de Sidra Maçã de Alcobaça Continente surge no âmbito de uma iniciativa do Clube de Produtores Continente, a "Feira do Desperdício". Na produção frutícola são gerados alguns subprodutos, nomeadamente ao nível da produção de maçã, que pelo seu calibre, pelo seu aspeto não são valorizados em fresco. Surgindo, por isso, a necessidade de valorizar esta maçã, que é considerada subproduto, num produto diferenciador e de economia circular.
SONAE	A.S47	O Clube de Produtores Continente (CPC) é uma estrutura de apoio à produção agrícola e agropecuária portuguesa, criado em 1998 para aproximar a MC aos produtores nacionais de norte a sul do país, Açores e Madeira. A sua missão é promover os produtos nacionais de acordo com elevados padrões de qualidade e segurança, apoiando os seus produtores de forma consistente e estruturada. Um trabalho de parceria, com suporte em conhecimento técnico-científico, em que todos ganham.
SONAE	A.S48	Declaração para a Sustentabilidade - Clube de Produtores Continente Para promover a produção e o consumo sustentáveis, e um sistema alimentar que respeita o ambiente, o Clube de Produtores Continente estabeleceu, com os seus produtores membros, uma Declaração para a Sustentabilidade, baseada em 11 princípios e diversas iniciativas. Iniciativas estas, que contemplam todas as áreas do sector agroalimentar e as boas práticas de produção, embalagem, nutrição, etc. que deverão ser atingidas. Esta Declaração para a Sustentabilidade, apresentada aos 251 membros do Clube, está alinha ao 12º objetivo da ONU (Produção e Consumo Sustentáveis), à Estratégia Europeia Farm to Fork (Do Prado ao Prato – Um sistema alimentar justo, saudável e respeitador do ambiente) e ao Roteiro para a Neutralidade Carbónica 2050 (RNC2050) do Ministério do Ambiente e da Transição Energética. A execução das iniciativas previstas nesta declaração terá um prazo de três anos. Num trabalho de parceria, implementação e verificação, o Clube de Produtores Continente estará a apoiar os produtores nacionais, ajudando-os e preparando-os para o cumprimento das metas previstas pela Comissão Europeia. No futuro, o cumprimento deste Acordo para a Sustentabilidade poderá ser critério na decisão de compras.
SONAE	A.S49	Projeto "Searas de Trigo com Biodiversidade – Salvemos a Águia Caçadeira" A Águia-caçadeira é uma das aves em maior declínio da fauna terrestre nacional, muito devido à substituição da cultura cerealífera de trigo e aveia por prados permanentes, cujo corte se faz mais cedo coincidindo com o período de nidificação desta espécie e que pode causar a perda de ovos, crias e, por vezes, adultos também. Com o objetivo de promover a conservação desta espécie, o Clube de Produtores Continente, a Associação Nacional de Produtores de Proteaginosas, Oleaginosas e Cereais (ANPOC) e o Centro de Investigação em Biodiversidade e Recursos Genéticos (CIBIO/BIOPOLIS) da Universidade do Porto, com a colaboração do Instituto da Conservação da Natureza e das Florestas (ICNF), juntaram-se num projeto que pretende valorizar o contributo das searas de trigo nacionais para a promoção de biodiversidade de aves, incluindo a de espécies ameaçadas como a Águiacaçadeira. Os agricultores e proprietários de terrenos estão a ajudar ativamente na identificação das colónias destas aves, enviando ao CIBIO/BIOPOLIS e ao ICNF informações sobre os avistamentos das águias, o número de animais e, sempre que possível, o sexo dos mesmos, além de implementarem voluntariamente medidas de proteção dos ninhos e crias (delimitando o espaço em que estes se encontram, para que não haja atividade de máquinas agrícolas e instalando proteções anti-predadores, por exemplo). Até ao momento já foram acompanhadas 13 ceifas de 26 produtores nacionais para implementar estas medidas. Através desta iniciativa, as cerca de 7 toneladas de farinha utilizadas diariamente nas padarias das lojas Continente, já provêm de searas de trigo da região do Alentejo que são monitorizadas de forma a proteger a biodiversidade e a conservação de aves em extinção. O Clube de Produtores Continente está comprometido com a proteção da fauna e flora do nosso país, com a biodiversidade e a sustentabilidade ambiental, acreditando que este projeto pode ter um impacto muito significativo na sobrevivência e recuperação desta espécie atualmente em vias de desaparecer das planícies alentejanas.
SONAE	A.S50	Resíduo Zero – Frutas e Legumes Nacionais sem Resíduos de Pesticidas O Projeto Resíduo Zero resulta da parceria entre o Clube de Produtores Continente e a Zerya (consultora espanhola) e visa o desenvolvimento de sistemas de produção agrícola personalizados, com enfoque em boas práticas agrícolas e produtos livres de resíduos e com vista à certificação dos produtores. Nestes sistemas é garantida a obtenção de produtos hortofrutícolas livres de resíduos de pesticidas e em que são salvaguardados princípios de uso eficiente de recursos, menor consumo energético, menores emissões e maior controlo sobre aspetos microbiológicos, questão-chave no que respeita à segurança alimentar e integração com o meio ambiente, garantido a sustentabilidade de todo o sistema agrícola. Um sistema que não se cinge a um conjunto de proibições, mas sim a um conhecimento aprofundado da produção e utilização racional de recursos, nomeadamente fitofármacos. O objetivo do projeto é garantir Hortofrutícolas Nacionais Resíduo Zero nas lojas Continente. Neste projeto estão envolvidos 29 produtores de hortofrutícolas membros do Clube de Produtores Continente, que irão aplicar modelos de gestão e produção desenvolvidos pela Zerya e, então, ter certificada a sua produção e produtos como Resíduo Zero. Neste momento estarão afetos ao projeto do Clube de Produtores Continente cerca de 1000 hectares. O Clube de Produtores assume assim um compromisso com a produção vegetal sem resíduos, garantindo boas práticas na origem, e frutas e legumes Continente mais saudáveis. Este compromisso tem sido materializado através do apoio e suporte de custos da formação e da própria certificação dos produtores.
SONAE	A.S51	Juntos por aqueles que mais precisam Somos um, somos todos. Somos aqueles que nos procuram diariamente, as instituições que ajudamos, os animais que salvamos. Somos a mão amiga que abraça no momento de solidão, que acende uma luz de esperança quando tudo parece ter menos brilho. Queremos ser cada vez mais um investidor social, ter uma atuação inovadora, pioneira e interventiva no universo empresarial. Por tal, anualmente, desenvolvemos inúmeras iniciativas de apoio à comunidade, onde aplicamos as melhores práticas e que contam com parceiros e milhares de portugueses, a nível nacional e local. A todos eles, o nosso sincero obrigado.
SONAE	A.S52	Enquanto investidor social, a Missão Continente reforça o seu papel transformador para gerar impacto social positivo e mensurável nos projetos que financia. É um promotor ativo da mudança ampla e duradoura nas comunidades em que está inserido, em prol da criação de valor económico, INICIATIVAS NO ÂMBITO DA SUSTENTABILIDADE · APOIAR A COMUNIDADE social e ambiental sustentável, trabalhando com parceiros como o Portugal Inovação Social, a Casa do Impacto, a EVPA e a Sair da Casca. Em 2021, fomos investidores sociais dos seguintes projetos: •

		PÊLOS DOIS | DTC SOCIAL - Atividades de reabilitação de reclusos com animais e reintegração dos animais em famílias. • SER MULHER | MUNDO A SORRIR - Contribuir para a melhoria física e psicológica de 50 mulheres vítimas de violência doméstica, através de apoio psicossocial e reabilitação oral. • MUM_ON_CV | VIDA NORTE - Projeto de capacitação e promoção da integração profissional de grávidas e recém mães, através da promoção de competências e da concretização dos valores da Inclusão e da Igualdade de Género.
SONAE	A.S53	No Natal de 2021, quisemos inspirar os pequenos gestos, em prol do combate à solidão e ao isolamento social. A maior campanha de sempre da Missão Continente, Luzes com Presença, terminou com um total de 1 milhão e 200 mil euros angariados. O valor reverteu para financiar 11 instituições com atuação nacional e local, com projetos de combate à solidão e ações concretas como apoio emocional especializado por telefone, apoio à inclusão digital, cuidados de saúde complementares, sessões de expressão plástica e musical, animais de companhia, visitas ao domicílio e acompanhamento na realização de tarefas quotidianas, entre outras atividades e formas de ajuda especializada a quem mais precisa.
SONAE	A.S54	Instituições apoiadas: • ACASO - Associação Cultural e Apoio Social de Olhão | Olhão • Aldeias Humanitar | Sernancelhe • Associação Coração Amarelo | Porto de Mós • Associação Mais Proximidade | Lisboa • Centro Social Nossa Sr.ª do Extremo | Vila Pouca de Aguiar • CRESAÇOR | Açores, São Miguel • Cruz Vermelha Portuguesa – Teleassistência | Nacional • DTC Social | Região Norte • Espaço T - Associação para o apoio à integração social e comunitária - Arte ao Domicílio | Porto • SOS Voz Amiga | Nacional • Viver de Afetos - Associação de Desenvolvimento Comunitário Câmara de Lobos Viva | Madeira
SONAE	A.S55	Mas, afinal, o que é os Heróis Missão Continente? Um movimento orgânico onde todos nos transformamos em heróis assumindo missões que nos são próximas em prol de um futuro melhor A iniciativa Heróis Missão Continente pretende mobilizar as pessoas a adotar melhores hábitos, atuar nas suas comunidades e gerar mais participações, criando um movimento natural e orgânico de boas ações. Os Heróis Missão Continente são pessoas comuns, que através de pequenos gestos e praticando boas ações, conseguem gerar impacto positivo na sua vida, na dos outros e no planeta. Com esta iniciativa queremos encorajar os membros de cada comunidade a contribuir, assumindo responsabilidade e uma ação local. Qualquer pessoa pode participar. Basta aceder ao site da Missão Continente, fazer a inscrição e selecionar uma das missões disponíveis, tão simples como cozinhar uma refeição saudável, fazer a separação de lixo em casa ou ajudar um vizinho mais vulnerável. O objetivo é partilhar experiências para inspirar outros a juntarem-se a este grande movimento nacional e, assim, formar uma sociedade mais solidária, preocupada e sustentável.
SONAE	A.S56	Em 2021, a Missão Continente reaproveitou o equivalente a 21,4 milhões de euros de produtos alimentares através da redistribuição dos excedentes das lojas. Cerca de 19,2 milhões de euros em excedentes alimentares foram doados a um conjunto de 1.448 instituições de todo o país. As doações das lojas Continente, que decorrem durante o ano por todo o país, aumentaram 39,1% e chegaram a mais 370 instituições face a 2020.
SONAE	A.S57	A Missão Continente apoia instituições de solidariedade social e associações de bem-estar animal em campanhas locais e nacionais de recolha de bens. Empenhamo-nos em apoiar as campanhas de angariação de bens essenciais levadas a cabo por instituições de solidariedade social, quer de âmbito local como nacional. E nunca nos esquecemos que a generosidade dos nossos clientes tem sido fundamental para melhorar a vida de muitos. Acreditamos que juntos vamos mais longe. Como damos força a esta causa? Disponibilizamos o espaço físico das nossas lojas e contribuímos com apoio logístico e de comunicação, amplificando assim o sucesso dos resultados das instituições.
SONAE	A.S58	Apoios Covid-19 A Missão Continente apoia instituições de solidariedade social e associações de bem-estar animal em campanhas locais e nacionais de recolha de bens. Empenhamo-nos em apoiar as campanhas de angariação de bens essenciais levadas a cabo por instituições de solidariedade social, quer de âmbito local como nacional. E nunca nos esquecemos que a generosidade dos nossos clientes tem sido fundamental para melhorar a vida de muitos. Acreditamos que juntos vamos mais longe. Como damos força a esta causa? Disponibilizamos o espaço físico das nossas lojas e contribuímos com apoio logístico e de comunicação, amplificando assim o sucesso dos resultados das instituições. Unidade Hospital Casa dos Atletas FPF SUCH - Serviço de Utilização Comum dos Hospitais 25.000 unidades de alimentos doados para utentes e equipas de trabalho 200 kg + 50 mantas doados a profissionais de saúde, forças de segurança e doentes Ordem dos Enfermeiros 8.000 unidades de bens alimentares e de cuidado e higiene pessoal
SONAE	A.S59	• Financiamento de 2.700 testes, equivalente a 40 famílias; • Parceria Centro de Testes de Ciências (CT Ciências), Faculdade de Ciências da Univ. Lisboa (Ciências ULisboa), Associação Nacional de Cuidadores Informais (ANCI); • Programa de vigilância epidemiológica, regular e gratuito para Cuidadores informais, Pessoas Cuidadas, e familiares em convivência direta. "Food Trucks Are Safe" do Chef Chakall Doação de alimentos para confeção e distribuição de 8 mil refeições aos profissionais de saúde de nove hospitais. #TODOSJUNTOS • 50 ton de bens alimentares doados pela Missão Continente através da Rede de Emergência Alimentar; • 450 mil pessoas em situação de vulnerabilidade apoiadas. • doados ao "Virar da página" do Rotary Club Braga Norte; • Mais de 10 mil produtos em cabazes doados à Re-food e Farense Futebol Clube; • Mais de 1.700 produtos alimentares e de higiene pessoal doados à Cama Solidária; • Doação de televisões Leds ao Hospital de Cantanhede.
SONAE	A.S60	A Missão Continente desenvolveu um saco de compras solidário para ajudar instituições de apoio social e/ou bem-estar animal. Números alcançados desde o início da iniciativa (agosto 2020) 90.000 € 3 Valor total doado instituições: Associação Portuguesa dos Bombeiros Voluntários, Animalife e Cruz Vermelha Portuguesa
SONAE	A.S61	Bolas de Natal solidárias A campanha a nível nacional desenvolvida pela Missão Continente para apoiar as Aldeiras de Crianças SOS consistiu numa ação de venda de Bolas de Natal solidárias com mensagens inspiradas no "Amor" e na "Família". A adesão a esta campanha reforça a confiança e credibilidade dos portugueses na causa defendida pela organização, possibilitando assim a continuação da sua missão: proporcionar às crianças e jovens os cuidados que necessitam e as oportunidades que tanto merecem.
SONAE	A.S62	Contos solidários Disney A Missão Continente e a LeYa lançaram uma coleção de contos clássicos infantis da Disney com o objetivo de ajudar uma causa. A compra dos "Contos Solidários" é uma forma simples e solidária de ajudar e, simultaneamente, uma hipótese de conhecer algumas das mais belas histórias da Disney. Através destas histórias, as preferidas da maioria das crianças, pretendeu-se criar um ambiente de conforto e inspiração, contribuindo para mais sorrisos nos serviços pediátricos portugueses durante as visitas dos Doutores Palhaços, temporariamente afastados desde 2020 devido à Covid-19, mas presentes através do canal TV ONV. Esta iniciativa gerou um valor total doado de 217.411,50 €, desde novembro de 2019 a dezembro de 2021.
SONAE	A.S63	Combate ao excesso de peso e obesidade Este programa atua na sensibilização e intervenção com um plano de acompanhamento personalizado a colaboradores com excesso de peso, durante um período de 6 meses. 73% perderam peso entre a consulta inicial e a intermédia 70% diminuíram massa gorda ao fim de 90 dias 73% reduziram o perímetro abdominal

SONAE	A.S64	Em 2019, a MC tornou-se no 1º retalhista português a associar-se ao New Plastics Economy Global Commitment, juntando-se a um conjunto de entidades de diferentes setores, que partilham uma visão comum para uma economia circular do plástico. Desde então, a MC reporta anualmente o seu progresso em relação às ambiciosas metas que definiu para as suas embalagens de plástico e com as quais está profundamente comprometida, nomeadamente, assegurar que até 2025 as suas embalagens de marca própria são recicláveis, reutilizáveis ou compostáveis. Este é um caminho feito de muitas, pequenas e grandes, mudanças que, no seu conjunto, atualmente se traduzem atualmente numa taxa de reciclabilidade das nossas embalagens de plástico de 74,7%.
SONAE	A.S65	2021, o café do sistema exclusivo Continente tem uma nova embalagem, mais compacta e de cartão 100% reciclável, permitindo uma poupança de 26 toneladas de plástico por ano.
SONAE	A.S66	O Continente alterou a composição das garrafas de água de marca própria, de 0,33l aos garrafões de 6l, incorporando 25% de plástico reciclado em cada uma, o que resultará na eliminação de 400 toneladas de plástico virgem por ano.
SONAE	A.S67	Ganha o Planeta! Em 2021 participámos em cinco projetos-piloto de sistemas de incentivo ao depósito de embalagens de bebidas, implementando este serviço em 25 lojas Continente, de norte a sul do país, e colaborando com várias entidades – como a APED, APIAM-Probeb, e os municípios de Cascais e Vila Real.
SONAE	A.S68	Plug&Charge Estamos ainda mais elétricos! Em 2020, o Continente inaugurou os primeiros hubs Plug&Charge do país. Este serviço simples e inovador permite aos clientes carregar veículos elétricos com até 200 km de autonomia em 1 hora, enquanto fazem compras na loja. Este projeto tem como objetivo melhorar a eficiência de consumo e a sustentabilidade ambiental, e contribuir para reduzir a pegada carbónica, compromisso assumido na assinatura do Acordo de Paris.
SONAE	A.S69	Continuamos determinados na proteção do ambiente, na minimização dos impactes ambientais negativos das nossas atividades, na promoção de boas práticas quer internamente quer na comunidade e na melhoria do nosso desempenho ambiental. Produção e consumo de eletricidade 70 certificações ambientais a partir de fontes renováveis: 24 GWh = aumento de 105% 6.800 toneladas de emissões A.CO2 evitadas Certificações de sustentabilidade Aumento para 83,6% taxa de valorização dos resíduos aumento de artigos de marca própria com certificações de sustentabilidade INICIATIVAS NO ÂMBITO DA SUSTENTABILIDADE · BOAS PRÁTICAS AMBIENTAIS 63 de acordo com a ISO 14001 (lojas, entrepostos e centro de processamento)
SONAE	A.S70	Excedentes alimentares são todos os produtos que deixaram de ter valor comercial, mas que conservam perfeitas condições de consumo. O Continente assumiu como Missão doar diariamente os seus excedentes, alimentares e não alimentares, e apoiar centenas de instituições de solidariedade social e associações de bem-estar animal.
SONAE	A.S71	Com a sua ajuda, conseguimos contribuir para combater o desamparo em Portugal. Apoiamos cerca de 1000 instituições por todo o país, algumas delas bem perto da sua casa. No Natal de 2023, quem ajudou pode escolher a causa que queria apoiar, entre cerca de 1000 instituições distribuídas pelo país. Juntos, provamos que a ajuda mora (mesmo) ao lado.
SONAE	A.S72	A Missão Continente e a LeYa lançaram uma coleção de contos clássicos infantis da Disney com o objetivo de apoiar uma causa. Atualmente está a decorrer a 3.ª edição dos Contos Solidários para apoiarmos o IPO Porto e Lisboa. O donativo vai permitir investir na requalificação, modernização e melhoria das condições hoteleiras e do percurso do doente e familiares ou cuidadores do IPO Porto e Lisboa.
SONAE	A.S73	Sacos Solidários Missão Continente estão de volta com o objetivo de envolver a comunidade no apoio a 6 causas. Juntos, no ano em que celebramos 20 anos da Missão Continente, vamos angariar donativos para 24 instituições de apoio social.
SONAE	A.S74	Refood A missão da Refood é contribuir para uma sociedade mais sustentável, justa e solidária, através da recuperação de comida em boas condições e distribuição da mesma por pessoas necessitadas. Este é um movimento independente e 100% voluntário, orientado por cidadãos e organizado em comunidades locais.<br>Comunidade Vida e Paz Esta instituição pretende acolher pessoas em situação de sem-abrigo ou situação de vulnerabilidade social, ajudando-as a (re)construir o seu projeto de vida. Assim, todas as noites são distribuídas entre 450 e 500 ceias, através das Equipas Voluntárias de Rua, que pretendem criar uma relação que permita despertar o desejo de abandonar a rua e iniciar um caminho de mudança.<br>Porta Solidária O projeto Porta Solidária apoia famílias em situação de grave carência económica e pessoas em situação de sem-abrigo, providenciando roupa e medicamentos a todos os que se dirigem à "sala de atendimento social". Atualmente, são servidos cerca de 400 jantares todos os dias da semana, que correspondem, muitas vezes, à única refeição que as pessoas têm nesse dia.<br>Cáritas Paroquial da Matriz de Portimão À semelhança de outras Cáritas Paroquiais espalhadas pelo país, esta é uma instituição de referência na zona de Portimão, que oferece apoio alimentar a mais de 800 pessoas carenciadas. Existe também uma loja social e uma barbearia social, a custo zero, para pessoas carenciadas e em situação de sem-abrigo. Espaço T Neste espaço o "t" significa "todos", sendo que a arte é utilizada como instrumento de desenvolvimento de competências artísticas, pessoais, sociais, culturais e formativas. O principal diferenciador desta instituição é a promoção da inclusão de todos, incluindo pessoas com deficiências físicas, imigrantes e pessoas com dificuldades psicossociais. Corações com Coroa Surgiu em 2012 e é uma organização que trabalha na área da Igualdade de Género e dos Direitos Humanos. Tem como missão apoiar, informar e capacitar jovens mulheres, através da atribuição de bolsas de estudo, disponibilização de acompanhamento psicológico gratuito e ações nas escolas sobre a violência no namoro ou a menstruação e a sustentabilidade. Teatro do Vão O Teatro do Vão tem como principal objetivo a promoção da cultura e da criatividade, entendidas como veículos para o desenvolvimento do ser humano. Através de espetáculos de teatro, procura-se potenciar novos universos plurais, inclusivos e menos binários. As peças pretendem ser democráticas e abordam temas como a representatividade e a acessibilidade. Associação Salvador A Associação Salvador tem como missão promover a inclusão das pessoas com deficiência motora na sociedade e melhorar a sua qualidade de vida, potenciando os seus talentos e sensibilizando para a igualdade de oportunidades. Foi fundada em 2003 e tem desenvolvido projetos ambiciosos nas áreas do Conhecimento, Integração e Sensibilização, já tendo ajudando mais de 4 mil pessoas. ALARGAMOS HORIZONTES O Saco Solidário Missão Continente "Alargamos Horizontes" está à venda em todas as lojas Continente, Continente Modelo e Continente Bom Dia (exceto Madeira). Por cada saco vendido, 0,50 € serão doados às Instituições UNICEF, Um Pequeno Gesto, Hope for Fulanis e The Big Hand. UNICEF Presente em países marcados por conflitos e catástrofes, trabalhando para que todas as crianças tenham o direito à sobrevivência, cuidados de saúde, nutrição adequada, acesso a água, proteção e educação. A UNICEF é financiada inteiramente por contribuições voluntárias e por tal, a realização do seu trabalho, só é possível com os donativos que recebe. UM PEQUENO GESTO Fundada em 2004, esta ONG portuguesa acredita que o acesso a educação, nutrição e um ambiente saudável e digno, permite às crianças moçambicanas quebrar o ciclo de pobreza. Promove a melhoria

		das condições de vida de crianças e famílias desfavorecidas através de programas de Apadrinhamento individual, de Apoio à Pobreza, Infraestruturas, Educação e Sustentabilidade. HOPE FOR FULANIS ONG direcionada para o desenvolvimento sustentável em África e no Mundo, tem por finalidade a ajuda humanitária nas mais diversas áreas, como a educação, a nutrição infantil, a saúde, a formação profissional e a captação de água potável. Propõe-se a promover o desenvolvimento humano em todas as suas formas, procurando que as comunidades se tornem autossustentáveis. THE BIG HAND Fundada em 2010, é uma ONG que tem como missão promover o bem-estar das crianças que vivem em condições desfavoráveis, em Moçambique, garantindo o acesso à educação, cuidados de saúde, nutrição, água e saneamento básico. Centrado na criança, constrói escolas, professores e desenvolve programas inclusivos e parcerias locais, que visam preparar as crianças para a vida na comunidade onde vivem. Bom para a Madeira O Saco Solidário Missão Continente “Bom para a Madeira” está à venda nas lojas Continente Modelo da Região Autónoma da Madeira (RAM), sendo exclusivo para esta região. Por cada saco vendido, 0,50€ serão doados às instituições Associação Santana Cidade Solidária (ASCS), Banco Alimentar Madeira, Centro Social e Paroquial de Santo António e à Sociedade Protetora dos Animais Domésticos (SPAD). Associação Santana Cidade Solidária Fundada a partir da necessidade de criar respostas sociais no Concelho de Santana, na Madeira, a ASCS teve o objetivo inicial de construir o Lar de Idosos de Santana. Nos últimos 21 anos, tem fomentado a promoção dos diferentes grupos etários do Concelho, no que concerne aos aspetos sociais, económicos, culturais e desportivos. Banco Alimentar Madeira O Banco Alimentar Madeira é um dos 21 Bancos Alimentares Contra a Fome atualmente em atividade, que procuram lutar contra o desperdício de produtos alimentares, encaminhando-os para distribuição a pessoas carenciadas. Na Madeira, existem 16 voluntários que conseguiram angariar 940 toneladas de produtos alimentares em 2021. Centro Social e Paroquial de Santo António Tem a missão de gerar valor acrescentado ao nível dos recursos humanos, financeiros e materiais, de forma a contribuir para o desenvolvimento de competências geradoras de qualidade de vida para os cidadãos. Entre outras respostas sociais, implementaram um Centro de Dia, um Serviço de Ajuda Domiciliária e uma Casa Abrigo para Vítimas de Violência Doméstica. Conheça o trabalho desta instituição. Sociedade Protetora dos Animais Domésticos Pretende lutar contra o abandono de animais domésticos na Madeira, através da criação de um canil e gatil com capacidade para 144 animais e da prestação de cuidados médico-veterinários a animais em situação de abandono. A SPAD procura aumentar a consciência da população para o abandono animal e promover a adoção responsável. Apoio Animal CUIDAMOS COM CARINHO O Saco Solidário Missão Continente "Cuidamos com Carinho" está à venda em todas as lojas Continente, Continente Modelo e Continente Bom Dia (exceto Madeira). Por cada saco vendido, 0,50 € serão doados às Instituições Projeto de Apoio a Vítimas Indefesas (PRAVI), Liga Portuguesa dos Direitos do Animal, Associação Animais de Rua e Animalife. PRAVI O Projeto de Apoio a Vítimas Indefesas é uma associação sem fins lucrativos e de utilidade pública, com âmbito nacional, que ajuda milhares de animais abandonados ou vítimas de mais tratos, recolhendo-os, tratando-os, esterilizando-os e promovendo a sua adoção responsável. Promove a formação de terapeutas e a educação de cães para Terapias e Atividades Assistidas por Animais, as quais aplica a crianças e jovens em risco e idosos dependentes ajudando-os a ultrapassar problemas do foro físico e psicológico, através dos animais. Saiba mais no site. Liga Portuguesa dos Direitos do Animal Há 42 anos, desde a sua fundação, que tem vindo a mudar a opinião pública em Portugal quanto à proteção dos animais e dos seus diretos. Tem um papel ativo na ligação com vários organismos nacionais e internacionais que interferem diretamente nas leis gerais e específicas para a defesa dos animais. Gere duas Associações Zoófilas com cerca de 200 animais e presta apoio a animais de famílias carenciadas. Saiba mais no site. Associação Animais de Rua Ajuda milhares de animais nas ruas, esterilizando-os para que não se reproduzam, tratando-os quando estão doentes e alimentando-os. Estes animais sem acesso a cuidados de saúde básicos, alimentação suficiente, abrigo ou conforto, são vítimas de fome, doenças e maustratos, condenados a existências curtas e sem qualidade de vida. A Associação procura minorar o sofrimento destes animais e evitar que outros nasçam nestas condições. Promove o convívio pacífico entre os animais e as pessoas, em respeito pelo bem-estar animal. Saiba mais no website. Animalife É uma associação de sensibilização e apoio social e ambiental, de âmbito nacional, sem fins lucrativos, cujo objetivo é diminuir o grave problema do abandono animal e uma das principais causas de abandono em Portugal: a vulnerabilidade económica e social das famílias. Atua em três âmbitos: apoio a associações e grupos de proteção, apoio a famílias em situação de carência económica, acompanhamento de pessoas em situação de sem-abrigo com animais a cargo. Saiba mais no website. Apoio à Infância e Juventude CONFIAMOS NO FUTURO O Saco Solidário Missão Continente "Confiamos no Futuro" está à venda em todas as lojas Continente, Continente Modelo e Continente Bom Dia. Por cada saco vendido, 0,50€ serão doados às Instituições Movimento Transformers, Obra do Frei Gil, CrescerSer e Casa S. Francisco de Assis. Movimento Transformers O Movimento Transformers existe para promover e aumentar a participação cívica e social de jovens nas suas comunidades em Portugal. Garante o seu envolvimento nas comunidades através de três eixos principais: o Voluntariado, o Associativismo e a Consciencialização. Saiba mais sobre o projeto no website. Obra do Frei Gil Assente na promoção dos direitos da criança e jovem e da família, tem respostas sociais de casas de acolhimento residencial, infantário e centro de apoio familiar e de aconselhamento parental. A Obra de Frei Gil tem a sua sede na Praia de Mira no distrito de Coimbra, e estende a sua ação de intervenção também pelos distritos de Aveiro e Porto. Saiba mais no website. CrescerSer A instituição de âmbito nacional tem o objetivo de apoiar crianças e jovens em perigo, privadas do meio familiar e vitimas de violência, promovendo, organizando e dinamizando serviços comunitários de apoio. Existe para dar resposta às situações de abandono, negligência, violência e abuso. A Instituição tem 6 Centros de Acolhimento para crianças dos 0 aos 18/25 anos. Saiba mais no site da instituição. Casa de S. Francisco de Assis Tem a missão de acolher e reintegrar crianças e jovens em perigo, famílias em dificuldade, jovens e adultos portadores de deficiência cognitiva ligeira e moderada. Através de várias respostas sociais de acolhimento, terapêuticas e de capacitação, pretende contribuir para a criação de condições que garantam a adequada satisfação das necessidades físicas, psíquicas, emocionais e sociais. Saiba mais no website.
SONAE	A.S75	Esta iniciativa tem como missão recolher rolhas de cortiça para a reciclagem, promover a cortiça e financiar a rearborização, bem como sensibilizar as pessoas.
SONAE	A.S76	Juntos, conseguimos ajudar Ao comprar um vale Presentes à Mesa, na sua loja Continente, Continente Modelo, Continente Bom Dia e Continente Online, no valor de 1€ ou 5€ contribuiu para apoiar: Rede Emergência Alimentar Instituições sociais locais associadas a cada loja Continente O valor de cada vale foi distribuído, em partes iguais, entre a Rede de Emergência Alimentar e as instituições sociais locais selecionadas por cada loja, numa ótica de envolvimento e compromisso com a comunidade local. O total angariado foi entregue na íntegra à Rede de Emergência Alimentar e para as instituições envolvidas.
SONAE	A.S77	Місія підтримки України* Um dos propósitos da Missão Continente é estar próximo dos que mais precisam, dando apoio imediato, mesmo em situações de calamidade. Por esta razão, o conflito na Ucrânia não passa despercebido. São

		milhares de inocentes em perigo imediato, entre os quais estão sobreviventes de bombardeamentos e refugiados que procuram segurança e paz. *Missão de Apoio à Ucrânia
SONAE	A.S78	Ilumine esta causa Este Natal vamos combater a solidão e o isolamento social Ao comprar um VALE LUZES COM PRESENÇA doou diretamente o seu valor a Instituições que combatem a solidão e o Isolamento Social. Esta contribuição será usada para promover projetos e ações de proximidade/integração, tais como chamadas telefónicas, visitas domiciliárias, apoio psicológico, animação cultural, entre muitas outras formas de ajuda.
SONAE	A.S79	Pelo 13.° ano consecutivo, o Continente recebeu a distinção "Marca de Confiança Ambiente", um reconhecimento que a marca agradece e de que se orgulha.
SONAE	A.S80	Missão Continente pretende contribuir para a redução do desperdício alimentar, sensibilizando para a adoção de hábitos de consumo mais conscientes e sustentáveis.
SONAE	A.S81	Com o objetivo de contribuir para a redução do desperdício alimentar, foram criados vários projetos que atuam desde o processo produtivo, ao consumo final dos produtos, até à criação de produtos de economia circular, que permitiram evitar mais de 37 M€ de desperdício em 2021.
SONAE	A.S82	Unidos contra o Desperdício A Missão Continente associou-se ao movimento cívico nacional 'Unidos Contra o Desperdício'. Este movimento, pretende chamar a atenção para a problemática do desperdício alimentar e no seu manifesto, lê-se que "quer tornar habitual o aproveitamento de excedentes, alertar para perdas e desperdícios, incentivar e facilitar a doação das sobras e promover o consumo responsável".
SONAE	A.S83	Começamos a aproveitar desde o início O Continente está a aproveitar o desperdício gerado na produção das maçãs e peras, transformando-as em snacks de fruta desidratada. Mas não só, também há sumos naturais que levam apenas estas frutas espremidas, sem qualquer adição de açúcar ou água, como o sumo 100% maçãs de Alcobaça. Banana Solteira Bananas soltas é um dos artigos com maior percentagem de desperdício nas nossas lojas. Porquê? Um dos principais motivos é o facto de os consumidores partirem os cachos e deixarem bananas soltas que, por esse motivo, se tornam menos apelativas à compra. Mas nós continuamos a achar as bananas "solteiras" atraentes! Num esforço de reverter esta situação e de sensibilizar os consumidores para o combate ao desperdício alimentar, disponibilizamos estas bananas soltas num único cesto. Será que vai conseguir resistir? Caixas Zer0% Desperdício Com o objetivo de contribuir para a redução do desperdício alimentar, foram criados vários projetos que atuam desde o processo produtivo, ao consumo final dos produtos, até à criação de produtos de economia circular, que permitiram evitar mais de 54M€ de desperdício em 2022. Em 2022, todas as lojas Continente, Continente Modelo e Continente Bom Dia tiveram as Caixas Zer0% Desperdício em vigor, evitando o desperdício de frutas e legumes. Em 2020, as Caixas Zer0% Desperdício estiveram em vigor em 49 lojas Continente. Este ano, chegarão às mais de 300 lojas, evitando o desperdício de cerca de 25 toneladas de frutas e legumes por ano. Etiquetas Cor de Rosa Quer ao mesmo tempo poupar e aproveitar? Esteja atento às nossas Etiquetas Cor de Rosa. Há mais de 10 anos que, nas lojas Continente, estas etiquetas comunicam uma redução de preço em produtos que se aproximam do fim do prazo de validade. Este sistema evita o desperdício e oferece, simultaneamente, benefício económico ao cliente. Uma nova forma de pensar o futuro O modelo de Economia Circular surgiu em oposição ao modelo de Economia Linear, que pressupõe que um produto tem um princípio e um fim. Dar uma nova vida ao que, supostamente, chegou ao fim é também uma forma de combater o desperdício alimentar. A Missão Continente tem criado produtos inovadores, com mais-valias para a Economia Circular. Conheça-os! Doação de excedentes alimentares Há 27 anos que faz parte do dia a dia das lojas Continente o reaproveitamento e redistribuição de bens alimentares: •Junto de instituições de solidariedade social e associações de apoio a animais; •Junto de colaboradores nas áreas sociais das lojas e entrepostos Até à data, já foram doados mais de 29,8 milhões de euros em excedente
SONAE	A.S84	Missão Viver sem Desperdiçar Um livro de receitas e muito mais! Do planeamento das suas refeições às compras, da organização da despensa ao frigorífico bem organizado sem nos esquecermos de dicas de conservação dos alimentos, neste ebook encontra os melhores conselhos para: criar receitas deliciosas e para toda a gente; reduzir o desperdício alimentar aí em casa; fazer as suas compras de forma mais consciente. O ambiente e a sua carteira agradecem.
SONAE	A.S85	EVITAR O DESPERDÍCIO dicas úteis para o dia a dia ALIMENTAÇÃO E AMBIENTE EM EQUÍLIBRIO ARMAZENE E CONSERVE BEM PARA PERDER MENOS 5 15 FORA DE VALIDADE, CONSUMIR OU NÃO? FESTEJE E APROVEITE TUDO 20 24 SEM DESPERDÍCIO AO LONGO DE TODA A CADEIA RECEITAS E DICAS
SONAE	A.S86	As Pastas 100% Caju e Mix Frutos Secos Continente são produtos que promovem a alimentação saudável e o acesso à nutrição básica das crianças que vivem na GuinéBissau. Uma iniciativa apoiada pela Missão Continente em parceria com a Hope for Fulanis.
SONAE	A.S87	Promover o acesso à nutrição básica Ao comprar a Pasta 100% Caju ou Mix Frutos Secos Continente numa das lojas Continente, uma parte do valor reverte para o centro de prevenção à desnutrição infantil, que garante acesso a refeições gratuitas de centenas de crianças guineenses, contribuindo como incentivo para a sua escolaridade e para o seu desenvolvimento físico, cognitivo e emocional.
SONAE	A.S88	De modo a evitar o desperdício de toneladas de alimentos excedentes, o Continente criou os Doces & Chutneys. Feitos a partir de frutas e legumes em fim de vida, os Doces & Chutneys são transformados, ganhando uma nova forma 100% saudável! Estes produtos exclusivos Continente são diferenciadores não só pelos princípios de Economia Circular e sustentabilidade em que assenta o seu processo, como são os primeiros do género lançados por uma cadeia de retalho.
SONAE	A.S89	A Missão Continente apoia diversas entidades que atuam na comunidade e que ajudam nas diferentes necessidades locais. Como? Apoiamos os projetos de várias instituições sociais, porque acreditamos que têm um papel fundamental na construção de uma vida melhor. Eles trabalham áreas importantes como terapias assistidas por cães, o combate à pobreza habitacional, o desporto para jovens com necessidades especiais o apoio a crianças de lares e famílias carenciadas.
SONAE	A.S90	Conheça em detalhe os projetos apoiados pela Missão Continente. Sinta-se contagiado a espalhar alguns sorrisos e ajudar quem mais precisa! Animais, parceiros de uma vida Atividades assistidas por cães em Centros de Dia e Lares de Idosos Não é segredo que os cães são os nossos melhores amigos. Mas sabia que também promovem o bem-estar físico e psicológico de quem os acompanha? A pensar nisso, a DTC Social criou o projeto "60+ Respostas Sociais", que procura trazer emoções positivas à comunidade sénior, através da promoção de atividades assistidas por cães em Centros de Dia e Lares de Idosos. Saiba mais sobre o projeto "60+ Respostas Sociais"! Terapias e intervenções assistidas por Animais A associação Ânimas ensina-nos que conviver, brincar e passear com cães promove o bem-estar psicológico, físico e mental, principalmente em crianças e pessoas com necessidades especiais. Os benefícios das terapias e intervenções assistidas por animais são diversos. Os animais são fonte de motivação, estímulo e foco na aprendizagem, auxiliam os profissionais de saúde na intervenção em situações de trauma e diminuem os níveis de ansiedade e stress.

		Conheça o Vitor no nosso vídeo e descubra como a sua vida ficou mais bonita depois de conhecer a sua melhor amiga Mira! Apoio de cães na integração na Sociedade A DTC Social também criou o projeto Fronteiras, que tem como missão promover o contacto da população reclusa com os animais. Este tipo de contacto melhora a empatia e a autoestima de quem brinca com eles, despertando muitos sorrisos e abraços. Uma nova geração cheia de esperança num futuro melhor Reabilitar casas e reconstruir Vidas A Just a Change é uma organização social que, de norte a sul do país, apoia no combate à pobreza habitacional, ajudando a tornar as casas de quem mais precisa em lugares dignos de serem vividos, onde reina a alegria e a esperança. A reabilitação de uma casa pode ser o ponto de partida para uma nova vida. Transformar a comunidade com talento Todos nós temos um talento e como o usamos faz toda a diferença. O Movimento Transformers tem como missão aumentar o envolvimento das pessoas nas suas comunidades através da partilha e aprendizagem daquilo que mais gostam de fazer. Esta troca de conhecimentos e informação muda a vida de muitos jovens em risco e de idosos isolados. Todos a mexer para o bemestar promover Ginástica para todos A Federação de Ginástica de Portugal criou o projeto Ginástica Solidária para ajudar jovens em situação de vulnerabilidade social ou com necessidades especiais a alcançarem as suas metas. A ginástica traz força e alegria à vida dos mais novos! Nenhuma criança ou jovem deve ser privado de sonhar e lutar pelos seus sonhos e é por esse motivo que a Missão Continente apoia este projeto. Natação adaptada A Missão Continente apoia a ANNP - Associação de Natação do Norte de Portugal com o intuito de levar a natação adaptada mais longe ou, quem sabe, ao pódio de grandes competições mundiais. Conheça aqui o projeto! Gerações futuras mais felizes Oportunidades educativas para os mais novos Com o objetivo de criar as melhores condições possíveis para as gerações futuras, a Missão Continente uniu esforços com a Associação Bagos D'Ouro para oferecer oportunidades educativas a mais de 150 crianças e jovens em situação de carência económica da região do Douro. A educação, aquela que é considerada a arma mais poderosa para mudar o Mundo, tem um papel fundamental na construção de uma sociedade mais justa e equitativa. Casas com Amor A Casa das Cores é um Centro de Acolhimento Temporário para crianças em risco, provenientes de lares disfuncionais. O Centro tem o papel de colorir a vida das crianças, oferecendo-lhes um ambiente saudável e equilibrado que lhes permite crescer como crianças e construir um projeto de vida mais feliz. Inovar para ajudar Apoio social à comunidade O Coração da Cidade é um espaço de espaço de apoio social que abastece de amor o coração dos mais carenciados. Atua em todas as frentes e promove apoio alimentar e clínico, jurídico e psicológico. E com uma mão da Missão Continente, dá a mão a quem mais precisa. Hortas na comunidade escolar Inspirados no ditado “plantamos hoje para colher amanhã”, a Missão Continente associou-se à Câmara Municipal de Esposende e à Noocity para fazer crescer o projeto de Sustentabilidade Alimentar - Geração S em 23 escolas do 1º Ciclo do ensino Básico do concelho.
SONAE	A.S91	Segundo o estudo BrandScore, divulgado pela consultora Scopen, o Continente foi a marca que mais contribuiu para apoiar os consumidores no contexto da crise provocada pela Covid-19, durante os primeiros meses da pandemia. Nomeado em “top-of-mind" por 1,4 milhões de portugueses, o Continente viu assim reconhecida a sua relevância e o contributo dado à sociedade. O apoio à comunidade foi prestado através da Missão Continente, que respondeu às inúmeras solicitações pelos vários setores da sociedade.
SONAE	A.S92	Os Portugueses conseguiam contribuir com 1€ através da chamada de valor acrescentado ou efetuar outras doações por MBWay ou transferência bancária. A Missão Continente acrescentou, ainda, a doação de 150 toneladas de bens alimentares à Cruz Vermelha Portuguesa e à Rede de Emergência Alimentar que fizeram a sua a gestão para os entregar às pessoas em situação de vulnerabilidade.
SONAE	A.S93	Desenvolvida pela Missão Continente e com o objetivo de apoiar a Aldeias de Crianças SOS, esta campanha contou com a venda de Bolas de Natal Solidárias, com mensagens inspiradas no “Amor” e na “Família”. A adesão a esta iniciativa reforçou a confiança e credibilidade dos portugueses na causa defendida pela organização, possibilitando assim a continuação da sua missão: proporcionar às crianças e jovens os cuidados que necessitam e as oportunidades que tanto merecem.
SONAE	A.S94	No âmbito da política de redução do uso de plástico, o Continente, em conjunto com a Sociedade Ponto Verde, está a colocar nos seus produtos instruções para reciclar cada embalagem.
SONAE	A.S95	Promover a cidadania ambiental e o consumo consciente Projeto ativo em todo o país Os recursos naturais esgotam-se, mas os comportamentos responsáveis de cada pessoa devem permanecer. Enquanto agente de mudança, temos de dar continuidade a um desempenho ambiental de excelência, envolvendo todas as pessoas possíveis numa causa comum de sustentabilidade ambiental. Neste sentido, as lojas Continente disponibilizam depósitos próprios que permitem aos clientes depositarem óleos alimentares usados, pilhas usadas, rolhas de cortiça, lâmpadas, equipamentos elétricos e eletrónicos e roupa.
SONAE	A.S96	Exclusivamente dedicado aos plásticos, o novo site do Continente agrega um conjunto de conteúdos informativos e pedagógicos, cujo objetivo principal passa por sensibilizar e esclarecer os consumidores sobre esta temática. O lançamento surge no âmbito do “Compromisso para o Uso Responsável dos Plásticos”, e vem na sequência de um conjunto de iniciativas que têm sido avançadas pelo Continente, entre as quais a assinatura do pacto internacional “New Plastics Economy Global Commitment” liderado pela Fundação Ellen MacArthur, em colaboração com o Programa das Nações Unidas para o Meio Ambiente.
SONAE	A.S97	O Continente foi o 1.º retalhista português a associar-se ao New Plastics Economy Global Commitment – um projeto liderado pela Fundação Ellen MacArthur em colaboração com o Programa das Nações Unidas para o Meio Ambiente, que assenta no fundamento da economia circular para o plástico, pretendendo eliminar de circulação todas as embalagens plásticas consideradas problemáticas ou desnecessárias.
APN	A.S98	Artigo publicado na Acta Portuguesa de Nutrição: TEOR DE AÇÚCAR NOS IOGURTES SÓLIDOS DE AROMA: COMPARAÇÃO ENTRE AS MARCAS COM MAIS VENDAS EM PORTUGAL
APN	A.S99	XXXII Congress of food and nutrition 2023 SIMPÓSIO SATÉLITE | SYMPOSIUM - CONTINENTE PRIVATE LABEL NUTRITION CHALLENGES AND OPPORTUNITIES: THE FUTURE Mayumi Delgado, Continente MODERADORA | CHAIR: Maria Guerreiro, Continente
APN	A.S100	XXXI Congress of food and nutrition 2022 SIMPÓSIO SATÉLITE > CONTINENTE INOVAÇÃO NAS MARCAS PRÓPRIAS: DE MÃOS DADAS COM A NUTRIÇÃO Mayumi Thaís Delgado, Continente Moderadora: Margarida Morão, Continente
APN	A.S101	XXXI Congress of food and nutrition 2022 COMUNICAÇÕES ORAIS > AÇÚCAR NOS IOGURTES SÓLIDOS DE AROMA: A REALIDADE NO MERCADO PORTUGUÊS
APN	A.S102	XXXI Congress of food and nutrition 2022 COMUNICAÇÕES ORAIS > AVALIAÇÃO DO EFEITO DA ADIÇÃO DE INGREDIENTES NA ANÁLISE SENSORIAL DO FIAMBRE
APN	A.S103	Patrocínio Congresso Alimentação e Nutrição 2022

FCNAUP	A.FCNAUP1	Identificam-se algumas das instituições, nacionais e internacionais, que acolheram estágios curriculares de finalistas da FCNAUP: Sonae
PNPAS	A.PNPAS2	Os parceiros do PNPAS | Empresas (projetos locais): Missão Continente, Gang dos frescos LIDL, Sabe bem Pingo Doce
ESB	A.ESB1	A Escola Superior de Biotecnologia desenvolve intercâmbios com múltiplas instituições dentro e fora de Portugal, incluindo centros de investigação, universidades, empresas e unidades de saúde: Modelo Continente Hipermercados, Bimbo, Danone Portugal, Imperial Produtos Alimentares, Nestlé Portugal, Primor Charcutaria Prima, Sonae, Cerealis, Vitacress Portugal
ESB	A.ESB2	A Escola Superior de Biotecnologia tem apostado na criação e desenvolvimento de parcerias com a indústria e outras organizações a nível nacional e internacional, sendo esta uma via privilegiada para fortalecer a transferência de conhecimento e tecnologia e de manter o diálogo com o mundo profissional. O resultado dessa aposta é visível e transparece em múltiplas formas de colaboração entre a ESB e diversas empresas, sempre mediadas pelos nossos estudantes dos vários níveis académicos (licenciatura, mestrado, doutoramento). Abaixo descrevem-se alguns exemplos que justificam o bom reconhecimento dos diplomados da ESB no mercado de trabalho: Cerealis, Continente, Delta, FIPA, SONAE MC,

SUMOL+COMPAL

Website	Code	CPA EXAMPLES
SUMOL+COMPAL	A.SC1	CENTRO DE FRUTOLOGIA COMPAL é um projeto único em Portugal, que visa estimular a inovação no sector frutícola ao longo da cadeia de valor com o objetivo de valorizar a fruta nacional nas vertentes da produção, da transformação e do consumo, através de iniciativas de investigação, formação e/ou sensibilização. Com espírito colaborativo e multidisciplinar, o Centro reúne entidades e organizações do sector, públicas e privadas, governamentais, empresariais e associativas, que ajudam a robustecer todos os conteúdos gerados e partilhados entre os formadores e os fruticultores
SUMOL+COMPAL	A.SC2	E-BOOK "COLHER SABER" A alimentação sustentável e a importância da fruta estão na ordem do dia e pequenas escolhas do nosso dia-a-dia poderão fazer toda a diferença. Para alertar e sensibilizar para esta temática, a Associação Portuguesa de Nutrição (APN) elaborou com o apoio do Centro de Frutologia Compal, o E-Book “Colher Saber” com recomendações úteis para promover uma alimentação saudável e sustentável, através do consumo de fruta
SUMOL+COMPAL	A.SC3	"COLHER SABER" E-BOOK Descubra o e-book desenvolvido pela Associação Portuguesa de Nutricionistas em colaboração com o Centro de Frutologia Compal.
SUMOL+COMPAL	A.SC4	INICIATIVA E INVESTIGAÇÃO O Laboratório do Centro de Frutologia Compal desenvolve iniciativas que, através da investigação, visam produzir conhecimento na área da fruta e dos seus produtos derivados, em diversos âmbitos como: fruticultura, tendências de consumo e evolução dos mercados, nutrição e saúde.
SUMOL+COMPAL	A.SC5	Projetos co!nanciados Projeto cLabel+ O projeto cLabel+ tem como principal objetivo responder aos desa!os que o consumidor coloca à indústria alimentar e pretende desenvolver processos e produtos que sejam entendidos pelo consumidor como consistentes com produtos com ingredientes naturais, com limitada utilização de um número elevado de aditivos, e que permitam, pelas suas propriedades físicas, químicas e nutricionais, proporcionar a experiência esperada, mantendo a segurança alimentar. Realização para projetos parciais (pps) Para o efeito, o projeto cLabel+ intergra um conjunto de projetos parciais (PPS) complementares que se integram na realização do objetivo global, nomeadamente: Valorização nutricional de produtos através dos macronutrientes e das condições de processamento
SUMOL+COMPAL	A.SC6	Projetos co!nanciados SUBA O Projeto SUBA tem como principal objetivo criar bebidas de baixo teor alcoólico, mais naturais e saudáveis do mercado, na forma de sumos fermentados que preservem o valor nutricional da fruta original.
SUMOL+COMPAL	A.SC7	Projetos co!nanciados PBA O Pacto da Bioeconomia Azul prevê desenvolver novos produtos, processos e serviços resultantes da incorporação de bens da bioeconomia azul em novas ou já existentes cadeias de valor, com impacto positivo no ambiente, na vida dos consumidores e nas exportações nacionais. A SUMOL+COMPAL participa no Vertical Algas, que envolve um total de 38 entidades e que visa dotar o setor nacional das algas de capacidade e vantagens competitivas necessárias para competir nos mercados globais e elevar a marca nacional na bioeconomia azul europeia. Mais especi!camente, a SUMOL+COMPAL pretende desenvolver refeições prontas e convenientes, com sensoriais inusitados e que combinem de forma nutricionalmente equilibrada, algas com outros ingredientes de origem vegetal.
SUMOL+COMPAL	A.SC8	SABE QUAIS SÃO OS PRINCIPAIS BENEFÍCIOS DO TOMATE? Descubra os benefícios do tomate.
SUMOL+COMPAL	A.SC9	FRUTAS E LEGUMES DE INVERNO Consumirmos alimentos da época tem vantagens para o ambiente e para o organismo.
SUMOL+COMPAL	A.SC10	CONHECE OS BENEFÍCIOS DA TANGERINA? Conhece as diferenças entre a tangerina, clementina e mandarina?
SUMOL+COMPAL	A.SC11	HÁ ALGUM LIMITE MÁXIMO DE CONSUMO DE SUMOS DE FRUTA? Em primeiro lugar convém reforçar que os sumos e néctares Compal são feitos essencialmente de fruta, um alimento benéfico para a saúde e cujo baixo consumo é apontado pela Organização Mundial de Saúde como uma das principais causas de problemas de saúde. Quando falamos de limites, não podemos dizer que existe um padrão máximo de consumo definido, uma vez que cada pessoa tem o seu quadro de saúde próprio, e os metabolismos diferem bastante entre indivíduos. Esta questão deve ser sempre colocada dentro de um caso específico, e não genérico, pelo que deverão dirigir essas questões ao médico de família, que será a pessoa mais qualificada para saber qual o quadro clínico da pessoa em questão, e se esse mesmo quadro clínico impõe limites ao consumo de determinados alimentos.
SUMOL+COMPAL	A.SC12	POSSO INCLUIR UM SUMO NA RECOMENDAÇÃO DE CONSUMO DE 3 A 5 PEÇAS DE FRUTA POR DIA? Beber sumos e néctares Compal contribui para o consumo diário de fruta. O que não podemos fazer é estabelecer uma relação quantitativa entre litros de sumo e peças de fruta. Para isso, temos Compal Essencial, doses de fruta que garantem a equivalência nutricional a uma peça de fruta.
SUMOL+COMPAL	A.SC13	PROMOVEMOS OS HORTOFRUTÍCOLAS E AS LEGUMINOSAS COMO PARTE DE UMA ALIMENTAÇÃO EQUILIBRADA
SUMOL+COMPAL	A.SC14	4 SUGESTÕES PARA CRIAR UMA ROTINA SAUDÁVEL Com o regresso às aulas e ao trabalho, aprenda a melhor o seu dia.
SUMOL+COMPAL	A.SC15	O QUE COMER ANTES E DEPOIS DO TREINO? Descubra os alimentos que fornecem energia necessária para o treino e melhoram a performance.
SUMOL+COMPAL	A.SC16	SOU DIABÉTICO, POSSO CONSUMIR COMPAL ESSENCIAL? Compal Essencial não tem açúcar adicionado, tem apenas o açúcar da própria fruta correspondentes aos valores que uma dose de fruta tem no seu estado ideal de

		maturação. Deve confirmar com o seu médico/nutricionista as quantidades de fruta/Compal Essencial que deve consumir por dia e por refeição.
SUMOL+COMPAL	A.SC17	FIBRA E SACIEDADE: TUDO O QUE DEVE SABER Conheça algumas das melhores fontes de fibra.
SUMOL+COMPAL	A.SC18	A IMPORTÂNCIA DA FRUTA Comer menos do que três peças de fruta por dia constitui o risco alimentar evitável que mais contribui para a perda de anos de vida saudável, estimando-se em 141 mil os anos de vida potencialmente perdidos pela população portuguesa. Fonte: Direção-Geral de Saúde (2015)
SUMOL+COMPAL	A.SC19	A fruta é um alimento fundamental que contribui para hábitos alimentares saudáveis, contudo nem sempre é fácil incluí-la no ritmo do dia-a-dia. Assim, Compal Essencial traz a Frutologia para o mundo dos snacks com opções de snacking saudáveis e saciantes para consumir a qualquer momento e em qualquer lugar.
SUMOL+COMPAL	A.SC20	ESSENCIAL DOSES DE FRUTA Quando o tempo é pouco, há sempre as Doses de Fruta, feitas com fruta esmagada e nada mais, sem qualquer adição de açúcares. A qualquer hora e em qualquer lugar pode cumprir com a sua necessidade diária de fruta, e de sabor.
SUMOL+COMPAL	A.SC21	TEM CORANTES? E CONSERVANTES? Compal Essencial não tem adição de açúcar, corantes nem conservantes. A cor do produto é tal e qual aquela que a fruta lhe dá (não usamos corantes) e o método de enchimento é asséptico, ou seja, as garrafas são cheias a quente e o produto pasteurizado sem qualquer contacto com o ar, permitindo a não utilização de conservantes. De seguida, o produto segue para câmaras frigoríficas (frio positivo, 0 a 6ºC) e continua em frio em toda a cadeia de transporte até si. Por não utilizarmos conservantes, a cápsula é totalmente celada, garantindo a estanquicidade da embalagem.
SUMOL+COMPAL	A.SC22	A gama Compal Vital é muito mais do que poucas calorias. São diversos os benefícios encontrados nestes produtos, a começar pela riqueza e variedade de frutos e ingredientes utilizados. Permanecem as opções Equilíbrio, com baixas calorias, já que são apenas adoçadas com stevia, um adoçante natural. As opções que fornecem Proteção Antiox, pela presença de vitaminas e minerais com estas propriedades. Veggie, que traz o melhor que os campos têm para oferecer, juntando legumes e fruta para criar sumos com perfis menos doces e o sabor característico dos vegetais. E a grande novidade, Bom Dia, com produtos saciantes, resultantes da combinação entre um elevado teor de fruta e ingredientes distintos. E sem qualquer tipo de açúcar ou edulcorante adicionado.
SUMOL+COMPAL	A.SC23	Os nossos vegetais são feitos com paixão e muita experiência. A sua preparação segue a receita tradicional de cozedura em água e sal, somente com ingredientes naturais, garantindo a sua qualidade e sabor rico, sem corantes, conservantes ou estabilizantes.
SUMOL+COMPAL	A.SC24	COMO SÃO FEITOS OS SUMOS DA COMPAL? A nossa principal matéria-prima é a fruta, os seus concentrados ou polpas. Temos sumos à base de concentrados e outros à base de polpas. Algumas das polpas de sabores populares como o pêssego e a pera são feitas na nossa fábrica, ou seja, recebemos os camiões com fruta directamente do pomar e extraímos a sua polpa, sempre respeitando os mais altos padrões de qualidade. Uma vez obtida a polpa ou o concentrado, fazemos o blend, ou seja, a mistura que irá resultar no sumo final. Finalmente a pasteurização e o embalamento que é feito em rigorosas condições de assepsia. Em suma: Receção da Fruta Seleção Lavagem Transformação em polpa Formulação Pasteurização Enchimento da embalagem Embalamento (embalagem secundária) Paletização Chegada do produto final aos Estabelecimentos
SUMOL+COMPAL	A.SC25	APOIAMOS AS COMUNIDADES LOCAIS Em 2021, apoiámos cerca de 60 organizações não governamentais com mais de 280 000 litros de produtos, contribuindo para o auxílio às famílias das nossas comunidades.
SUMOL+COMPAL	A.SC26	É URGENTE PROMOVER O CONSUMO DE FRUTA PELOS PORTUGUESES ...e com esse propósito o Observatório do Centro de Frutologia Compal propõe-se produzir e disseminar conhecimento sobre a fruta e os seus produtos derivados para sensibilizar e educar para a importância do seu consumo.
SUMOL+COMPAL	A.SC27	POMAR PEDAGÓGICO O Pomar Pedagógico do Centro de Frutologia Compal é o 1.º pomar pedagógico interativo que, em meio urbano, ensina sobre a produção de fruta em Portugal e sobre os benefícios e a importância da fruta para a saúde e para a nutrição. Complementando os formatos de pedagogia em sala, o Centro de Frutologia Compal convida as famílias e as crianças a saírem para o ar livre e para o terreno para adquirirem no Pomar conhecimento sobre fruta.
SUMOL+COMPAL	A.SC28	MAPA DAS FRUTAS DE PORTUGAL O Centro de Frutologia Compal em conjunto com o Centro de Informação Geoespacial do Exército (CIGeoE) desenvolveu o primeiro Mapa das Frutas de Portugal para que todas as crianças conheçam a origem da fruta portuguesa. O Mapa das Frutas de Portugal representa as áreas das frutas de Denominação de Origem Protegida e Indicação Geográfica Protegida que existem em Portugal. À semelhança de exemplos como o Mapa dos Rios ou o Mapa das Serras de Portugal, o Centro de Frutologia Compal alegrase por apresentar o primeiro Mapa das Frutas de Portugal feito com todo o rigor científico e em simultâneo orientado para a utilização pedagógica em sala de aula (ou fora dela). Apoio institucional da Direção Geral de Agricultura e Desenvolvimento Rural.
SUMOL+COMPAL	A.SC29	COMPAL DOA 1000 PEQUENOS-ALMOÇOS A QUEM MAIS PRECISA Jogo da Compal permitiu aos utilizadores entregar pequenos-almoços a crianças desfavorecidas.
SUMOL+COMPAL	A.SC30	A nossa Agenda subscreve 8 dos 17 Objetivos de Desenvolvimento Sustentável das Nações Unidas para 2030.
SUMOL+COMPAL	A.SC31	Chegou o mais Fresco dos sumos embalados. Feito de fruta exclusivamente portuguesa, Compal Fresco sabe ao que sabe a fruta acabada de espremer. E numa embalagem única produzida com material 100% reciclado, o que é natural fica ainda melhor.
SUMOL+COMPAL	A.SC32	O QUE É UMA DOSE DE FRUTA? Uma dose de fruta é definida entre 110-130g de fruta. No caso de Compal Essencial Dose de Fruta, consideramos a equivalência a 115g de fruta.
SUMOL+COMPAL	A.SC33	100% FRUTA É fruta e nada mais do que fruta, sem tirar nem pôr. Só assim garantimos o sabor inconfundível e 100% original da fruta cuidadosamente selecionada com todo o amor à Natureza. Sem açúcares adicionados.
SUMOL+COMPAL	A.SC34	BOM DIA Para dias desafiantes, manhãs fortalecedoras. A gama Bom Dia conta com uma variedade de produtos saciantes resultado da combinação entre um elevado teor de fruta e ingredientes distintos. Sem qualquer tipo de açúcar ou edulcorante adicionado.
SUMOL+COMPAL	A.SC35	2021 ANO INTERNACIONAL DAS FRUTAS E HORTÍCOLAS : ciclo de webinares em parceria com a Compal [APN]
SUMOL+COMPAL	A.SC36	JOGOS DIDÁTICOS DESENVOLVIDOS EM PARCERIA COM A COMPAL PARA A PORMOÇÃO DO CONSUMO DE LEGUMINOSAS [APN]
SUMOL+COMPAL	A.SC38	Patrocínio Congresso Alimentação e Nutrição 2022
SUMOL+COMPAL	A.SC39	Patrocínio Congresso Alimentação e Nutrição 2023
SUMOL+COMPAL	A.SC25	APOIAMOS AS COMUNIDADES LOCAIS Em 2021, apoiámos cerca de 60 organizações não governamentais com mais de 280 000 litros de produtos, contribuindo para o auxílio às famílias das nossas comunidades.

SUMOL+COMPAL	A.SC26	É URGENTE PROMOVER O CONSUMO DE FRUTA PELOS PORTUGUESES ...e com esse propósito o Observatório do Centro de Frutologia Compal propõe-se produzir e disseminar conhecimento sobre a fruta e os seus produtos derivados para sensibilizar e educar para a importância do seu consumo.
SUMOL+COMPAL	A.SC27	POMAR PEDAGÓGICO O Pomar Pedagógico do Centro de Frutologia Compal é o 1.º pomar pedagógico interativo que, em meio urbano, ensina sobre a produção de fruta em Portugal e sobre os benefícios e a importância da fruta para a saúde e para a nutrição. Complementando os formatos de pedagogia em sala, o Centro de Frutologia Compal convida as famílias e as crianças a saírem para o ar livre e para o terreno para adquirirem no Pomar conhecimento sobre fruta.
SUMOL+COMPAL	A.SC28	MAPA DAS FRUTAS DE PORTUGAL O Centro de Frutologia Compal em conjunto com o Centro de Informação Geoespacial do Exército (CIGeoE) desenvolveu o primeiro Mapa das Frutas de Portugal para que todas as crianças conheçam a origem da fruta portuguesa. O Mapa das Frutas de Portugal representa as áreas das frutas de Denominação de Origem Protegida e Indicação Geográfica Protegida que existem em Portugal. À semelhança de exemplos como o Mapa dos Rios ou o Mapa das Serras de Portugal, o Centro de Frutologia Compal alegrase por apresentar o primeiro Mapa das Frutas de Portugal feito com todo o rigor científico e em simultâneo orientado para a utilização pedagógica em sala de aula (ou fora dela). Apoio institucional da Direção Geral de Agricultura e Desenvolvimento Rural.
SUMOL+COMPAL	A.SC29	COMPAL DOA 1000 PEQUENOS-ALMOÇOS A QUEM MAIS PRECISA Jogo da Compal permitiu aos utilizadores entregar pequenos-almoços a crianças desfavorecidas.
SUMOL+COMPAL	A.SC30	A nossa Agenda subscreve 8 dos 17 Objetivos de Desenvolvimento Sustentável das Nações Unidas para 2030.
SUMOL+COMPAL	A.SC31	Chegou o mais Fresco dos sumos embalados. Feito de fruta exclusivamente portuguesa, Compal Fresco sabe ao que sabe a fruta acabada de espremer. E numa embalagem única produzida com material 100% reciclado, o que é natural fica ainda melhor.
SUMOL+COMPAL	A.SC32	O QUE É UMA DOSE DE FRUTA? Uma dose de fruta é definida entre 110-130g de fruta. No caso de Compal Essencial Dose de Fruta, consideramos a equivalência a 115g de fruta.
SUMOL+COMPAL	A.SC33	100% FRUTA É fruta e nada mais do que fruta, sem tirar nem pôr. Só assim garantimos o sabor inconfundível e 100% original da fruta cuidadosamente selecionada com todo o amor à Natureza. Sem açúcares adicionados.
SUMOL+COMPAL	A.SC34	BOM DIA Para dias desafiantes, manhãs fortalecedoras. A gama Bom Dia conta com uma variedade de produtos saciantes resultado da combinação entre um elevado teor de fruta e ingredientes distintos. Sem qualquer tipo de açúcar ou edulcorante adicionado.
SUMOL+COMPAL	A.SC35	2021 ANO INTERNACIONAL DAS FRUTAS E HORTÍCOLAS : ciclo de webinares em parceria com a Compal [APN]
SUMOL+COMPAL	A.SC36	JOGOS DIDÁTICOS DESENVOLVIDOS EM PARCERIA COM A COMPAL PARA A PORMOÇÃO DO CONSUMO DE LEGUMINOSAS [APN]
SUMOL+COMPAL	A.SC38	Patrocínio Congresso Alimentação e Nutrição 2022
SUMOL+COMPAL	A.SC39	Patrocínio Congresso Alimentação e Nutrição 2023
SUMOL+COMPAL	A.SC33	100% FRUTA É fruta e nada mais do que fruta, sem tirar nem pôr. Só assim garantimos o sabor inconfundível e 100% original da fruta cuidadosamente selecionada com todo o amor à Natureza. Sem açúcares adicionados.
SUMOL+COMPAL	A.SC34	BOM DIA Para dias desafiantes, manhãs fortalecedoras. A gama Bom Dia conta com uma variedade de produtos saciantes resultado da combinação entre um elevado teor de fruta e ingredientes distintos. Sem qualquer tipo de açúcar ou edulcorante adicionado.
APN	A.SC35	2021 ANO INTERNACIONAL DAS FRUTAS E HORTÍCOLAS : ciclo de webinares em parceria com a Compal [APN]
APN	A.SC36	JOGOS DIDÁTICOS DESENVOLVIDOS EM PARCERIA COM A COMPAL PARA A PORMOÇÃO DO CONSUMO DE LEGUMINOSAS [APN]
APN	A.SC38	Patrocínio Congresso Alimentação e Nutrição 2022
APN	A.SC39	Patrocínio Congresso Alimentação e Nutrição 2023

UNILEVER

Website	Code	CPA EXAMPLES
UNILEVER	A.U1	Ainda antes de 1949, já Jerónimo Martins comercializava em Portugal vários produtos da Unilever. Em Agosto de 1949, aquela relação comercial, transformou-se numa parceria que perdura num exemplo de cooperação comercial até hoje.
UNILEVER	A.U2	Além de conduzir a nossa própria pesquisa científica pioneira, trabalhamos intimamente com as principais autoridades mundiais, incluindo reguladores, cientistas governamentais e peritos académicos. Esta colaboração garante que utilizamos sempre os avanços científicos mais recentes dentro das nossas avaliações de segurança e sustentabilidade ambiental.
UNILEVER	A.U3	Conservantes Os conservantes, como o nome sugere, ajudam a manter as coisas frescas. Nós usamos conservantes para ajudar a proteger os nossos produtos de bactérias, fermentos e bolor. Sem eles, os nossos produtos poderiam começar a cheirar mal, mudar de cor ou ganhar bolor.
UNILEVER	A.U4	Listamos abaixo em ordem alfabética todas as várias funções que os nossos ingredientes desempenham - de fragrâncias a conservantes, emulsificantes a solventes.
UNILEVER	A.U5	A FIFA e a Unilever: marcas poderosas e o futebol feminino
UNILEVER	A.U6	Ir mais longe: seis formas que estamos a adotar para atingir as zero emissões
UNILEVER	A.U7	Como estamos a ajudar a virar o jogo das emissões do transporte marítimo
UNILEVER	A.U8	Somos orientados pelo nosso propósito: contribuir para que todas as pessoas vivam de forma sustentável
UNILEVER	A.U9	A Unilever possui um Centro de Garantia de Segurança e Ambiental (SEAC - "Safety & Environmental Assurance Centre") que emprega cerca de 200 pessoas, incluindo cientistas reconhecidos como líderes no seu campo.
UNILEVER	A.U10	Ação climática As alterações climáticas são agora uma crise climática. Temos um plano para nos levar até 2039 a emissões líquidas zero. Estamos a transicionar para a energia renovável em todas as nossas operações, ao encontrar novos ingredientes com menos carbono e expandir a nossa gama de produtos à base de plantas.

VITACRESS

Website	Number	CPA EXAMPLES

VITACRESS	A.V1	A Vitacress assume a sua responsabilidade na relação com as pessoas e com a comunidade envolvente através de protocolos de colaboração com várias instituições a nível nacional. É neste sentido que doamos produtos para cabazes sociais, integramos pessoas portadoras de deficiência em contexto de trabalho, instalamos hortas pedagógicas, apoiamos eventos de desenvolvimento local e projetos de proteção ambiental, apoiamos a formação curricular de jovens em idade escolar e envolvemonos em projetos de investigação científica.
VITACRESS	A.V2	Há 16 anos que começámos a produzir em modo biológico e continuamos a apurar a nossa técnica todos os dias: apesar de em 2018 este tipo de cultura representar 11% do total, temos em curso a conversão de solos que nos permitirá triplicar este valor nos próximos 3 a 4 anos.
VITACRESS	A.V3	Dedicamos ainda 10% dos nossos terrenos ao aumento da biodiversidade através de plantas que produzem tanto alimento como abrigo para várias espécies animais, nomeadamente insetos, e que por isso são auxiliares no controlo de pragas indesejáveis.
VITACRESS	A.V4	Ambiente (engloba a água, os resíduos, a energia, solos, proteção das culturas e a biodiversidade): Saúde e segurança no trabalho: Para nós, só existe um caminho: preservar o que é natural. A paixão e o respeito pelo ambiente faz-nos querer ir mais longe em tudo o que fazemos. Comprometemo-nos a utilizar sustentadamente os recursos naturais que temos ao nosso dispor, apostando nas melhores técnicas e boas práticas agrícolas e de produção, garantindo a sustentabilidade ambiental e do ecossistema, reconhecidas pelos standards da Global G.A.P., LEAF e ISO 14001 onde somos certificados. Ver política ambiental A Vitacress assume o compromisso de gerir os seus recursos assegurando a segurança, a saúde e o bem estar de todas as pessoas que são abrangidas pelo exercício da sua atividade, atuando sempre na perspectiva da protecção e prevenção, quer a nível da ocorrência de incidentes quer de doenças profissionais, promovendo a formação, informação e comunicação interna focada no desenvolvimento sustentável da cultura de segurança e saúde no trabalho, reconhecido pela certificação OSHAS 18001.
VITACRESS	A.V5	Qualidade e segurança alimentar Com o objectivo de fornecer os produtos mais frescos, saborosos e saudáveis ao longo de todo o ano a Vitacress, vê as suas boas práticas agrícolas e de processamento de saladas reconhecidas pelas certificações Global G.A.P. e BRC, garantindo os elevados níveis de qualidade e segurança do produto de forma consistente e em concordância com os requisitos legais e standards nacionais, internacionais e de cliente. Orgulhamo-nos por termos colaboradores responsáveis e que tudo fazem para garantir a proteção da natureza, o sucesso do nosso negócio e a qualidade de vida da comunidade em que nos integramos.
VITACRESS	A.V6	No âmbito da Responsabilidade Social a Vitacress assumiu protocolos de colaboração com várias instituições a nível nacional. Esta colaboração centra-se: No donativo de produtos destinados a cabazes sociais e à confeção de refeições. Na integração de pessoas portadoras de deficiência em contexto de trabalho. Na instalação de hortas pedagógicas. No apoio a eventos de desenvolvimento local. No apoio a projetos de proteção ambiental. No apoio à formação curricular de jovens em idade escolar. No apoio a projetos de investigação.
VITACRESS	A.V7	A sustentabilidade está na nossa natureza: Na Vitacress, há mais de 40 anos que cultivamos uma visão de futuro sustentável. Agora, através do Programa Second Nature, formalizamos o nosso compromisso de sustentabilidade rumo a um objectivo: Alcançar a neutralidade líquida de carbono (Net Carbon Zero) até 2040, 10 anos antes das metas da União Europeia.
VITACRESS	A.V8	Patrocínio Congresso Alimentação e Nutrição 2022
VITACRESS	A.V9	Patrocínio Congresso Alimentação e Nutrição 2023
ESB	A.ESB1	A Escola Superior de Biotecnologia desenvolve intercâmbios com múltiplas instituições dentro e fora de Portugal, incluindo centros de investigação, universidades, empresas e unidades de saúde: Modelo Continente Hipermercados, Bimbo, Danone Portugal, Imperial Produtos Alimentares, Nestlé Portugal, Primor Charcutaria Prima, Sonae, Cerealis, Vitacress Portugal
ESB	A.ESB4	A Semana Aberta é uma iniciativa da Escola Superior de Biotecnologia que se realiza desde 1992 e se repete todos os anos na primavera. Em 2024 a 32ª edição decorre nos dias 10, 11 e 12 de abril e está disponível sem custos para as escolas secundárias – todos os alunos dos cursos de ciências e tecnologias são bem vindos. As inscrições devem decorrer preferencialmente até 22 de março de 2024. Patrocinadores: Cerealis, Nestlé, Sovena
