# Supplementary material for: Reputation, Co-Regulation, and "Soft Capture": Corporate Political Activity of the Portuguese Food Industry
Source: Int J Health Policy Manag. 2026 Jun 1;15:9495. doi: 10.34172/ijhpm.9495 (PMC13729813; doi:10.34172/ijhpm.9495)
Supplement: Supplementary file 3 [file ijhpm-15-9495-s003.pdf]

Article title: Reputation, Co-Regulation, and "Soft Capture": Corporate Political Activity of the Portuguese Food Industry

Journal name: International Journal of Health Policy and Management (IJHPM)

Authors' information: Sofia da Costa[1,2]*, Mélissa Mialon[3,4], Marta Figueira[2,5], Maria João Gregório[1,2,5], Pedro Graça[1,2]

[1]Epidemiology Research Unit (EPIUnit), Institute of Public Health, University of Porto, Porto, Portugal.

[2]Faculty of Nutrition and Food Sciences, University of Porto, Porto, Portugal.

[3]Inserm, Université Paris Cité, Paris, France.

[4]School of Medicine, Trinity College Dublin, Dublin, Ireland.

[5]National Healthy Eating Promotion Program, Directorate-General of Health, Lisbon, Portugal.

***Correspondence to:** Sofia da Costa; Email: sofiacarvalhodacosta@gmail.com

Citation: da Costa S, Mialon M, Figueira M, Gregório MJ, Graça P. Reputation, co-regulation, and "soft capture": corporate political activity of the Portuguese food industry. Int J Health Policy Manag. 2026;15:9495.doi:10.34172/ijhpm.9495

Supplementary file 3. Interview Guide

Interview guide

Thank you very much for taking part in this research project, ***"Food and other unhealthy commodity industries in Portugal: corporate political activity, synergies, and government-led counter-strategies"***. I really appreciate your time and contribution.

Researcher: Before we start, are you in a private space where you feel comfortable, so that we can ensure your confidentiality?
The interview should last around 45 minutes to one hour.

The aim of this project is to better understand how industries, especially the food and retail sectors, can influence public health policies. This is particularly important in the context of preventing and controlling non-communicable diseases such as diabetes, cancer, and obesity.

When I say *"food industry"*, I'm referring broadly to large food processing companies, retailers, soft drink companies, fast food restaurants, food producers, trade associations, public relations firms, and other groups or individuals connected with food companies.

You were invited to participate because of your professional experience and your observations or interactions with the food industry. We believe you can provide valuable insights into its practices. Do you have any questions before we begin?

I will personally transcribe this interview and, if you would like, I can share the transcript with you to confirm its accuracy. All data will be securely stored at the Institute of Public Health of the University of Porto for six years, after which it will be deleted. Your name will not appear

anywhere in the study, and none of the information you provide will be identifiable as coming from you.

I will record only the audio of this interview and also take notes, to make sure your words are reproduced accurately. Please feel free to ask me to pause the recording at any time.
If you haven't already, please sign the consent form.

I'll now start the recording; shall we begin?

<u>Key Quesns</u>

- Could you tell me what kind of interactions you have had—or currently have—with the food industry in your professional life?

  (For example: your current job, previous positions, meetings, partnerships, or funding.)

- In which specific roles or situations have you observed or interacted with the food industry or its representatives?

- Have you ever felt that any of these interactions might pose a risk to public health? Why or why not?

- The literature shows strong evidence that certain practices by the food industry can directly or indirectly influence public health policy. I have a list of such practices here.

  Go through the strategies (in the separate document) one by one.

  - Looking at this list, have you personally observed or experienced any of them?

    Practices described in the literature

  - Do you feel these practices could pose a risk to public health? Why?
  - Are there any practices you've observed that are not on this list?
  - Which practices do you think are most influential on public health? And which are least influential?

- I'll now move on to another part of the project. The aim here is to systematically identify and monitor these practices using only publicly available information. The idea is to increase transparency and accountability in the food industry.

  - Do you think this type of work could be useful? Why?

  - What would you recommend as good ways of identifying such practices, beyond interviews like this?

Websites, registries, conferences, etc.

- We would also like to interview others who may have insights into this topic. Is there anyone you would suggest—such as former politicians, former food industry professionals, or other people with extensive experience?

- Is there anything else you would like to add that we haven't covered?

Thank you once again for sharing your valuable insights. Your contribution is very important for the success of this study.

Stop recording.

Guia de Entrevista

Começo por agradecer a sua participação no projeto de investigação *"**Food and other unhealthy commodity industries in Portugal: corporate political activity, synergies, and government-led counter-strategies**"*. Muito obrigada pelo seu tempo e contributo neste projeto.

Investigador: Encontra-se num espaço privado, e onde se sente à vontade, para que possamos assim proteger a sua confidencialidade?
Espero que a entrevista tenha a duração de cerca de 45 minutos a uma hora.

O objetivo deste projeto é a prevenção e o controlo de doenças não transmissíveis como diabetes, cancro, obesidade, entre outras. A indústria e o retalho alimentar têm sido considerados como um dos principais fatores de influência das políticas de saúde pública, sendo estas diretas ou não, em todo o mundo. O objetivo deste projeto visa desenvolver uma abordagem para identificar, compreender e monitorizar sistematicamente essas práticas.

Vou usar o termo "indústria alimentar" aqui para me referir a: grandes indústrias de processamento de alimentos, retalho alimentar, empresas de refrigerantes, restaurantes de *fast food*, bem como produtores de alimentos, sindicatos, empresas de relações públicas, e outros indivíduos ou grupos afiliados a empresas de alimentos.

Foi selecionado para participar neste estudo por causa da sua experiência profissional e observação ou interação com a indústria alimentar, e acreditamos que nos pode fornecer algumas informações críticas sobre as práticas da mesma. Tem alguma dúvida/questão até agora?

Eu mesma vou transcrever esta entrevista e, se estiver interessado, posso-lhe enviar a mesma para confirmar todas as informações escritas. Todos os dados relacionados a esta entrevista serão armazenados de forma segura no Instituto de Saúde Pública da Universidade do Porto, por um período de 6 anos após eu ter analisado todas as entrevistas e relatado os resultados na minha tese de doutoramento. Todas as informações que fornecer nesta entrevista não estarão vinculadas ao seu nome e não serão identificáveis como tendo sido fornecidas por si.

Eu vou gravar o áudio da entrevista (sem gravação de vídeo) e fazer anotações, para garantir a correta reprodução das suas declarações. Por favor, não hesite em pedir a interrupção da gravação em qualquer altura.
Por favor, assine o formulário de consentimento informado se ainda não o fez.
Vou dar início à gravação.
Eu tenho as perguntas aqui; podemos começar...?

<u>Perguntas-chave</u>

• Pode dizer-me que tipo de interação teve/tem com a indústria alimentar, como parte de sua profissão?

Emprego atual, empregos anteriores, reuniões, parcerias, fundos de investimento

• E especificamente, em quais funções profissionais e em que situações interagiu/observou a indústria alimentar ou seus representantes?

• Alguma vez sentiu que algum dos eventos/interações que mencionou podem representar um risco para a saúde pública? Por quê?

• Há forte evidência, na literatura, de que algumas das práticas da indústria de alimentos podem influenciar as políticas de saúde pública, direta ou indiretamente. Eu tenho uma lista de tais práticas aqui.

Apresente as estratégias (documento separado) uma a uma

- o Olhando para esta lista, pode indicar-me se experienciou ou observou algumas destas práticas? Estou interessada na sua própria experiência profissional aqui. Sente que essas práticas podem representar um risco para a saúde pública e por quê?

  Práticas atuais identificadas na literatura

- o Existe alguma outra prática que observou, e que não é mencionada aqui?

- o Consegue indicar quais dessas práticas considera mais influentes na saúde pública? E quais são menos influentes?

• Vou passar agora para outra parte do projeto, que tem como objetivo identificar e monitorizar sistematicamente todas essas práticas, usando apenas informações publicamente disponíveis – para aumentar a transparência e a responsabilidade da indústria alimentar.

- o Acha que este trabalho pode ser útil? Por quê?

- o Que recomendações tem sobre como identificar as práticas que observou além deste tipo de entrevistas?

  Sites, registros, conferências

• Finalmente, gostaríamos de entrevistar outras pessoas que possam fornecer informações sobre esta área. Lembra-se de alguém que possamos entrevistar sobre este assunto?

por exemplo, ex-políticos, ex-indústria alimentar, outras pessoas com vasta experiência nesta área

• Há mais alguma coisa que gostaria de acrescentar?

Agradeço uma vez mais a sua valiosa visão sobre esta área e pelo seu contributo para o sucesso deste estudo. Muito obrigada pela sua ajuda.

Desligue o gravador.

Action strategies

Action Strategy	Mechanisms	Example Practices
Access and influence policy-making	A-P1. Access policy-makers and policy Spaces	- Give incentives: provide finances, resources, hospitality and gifts to politicians, officials, political parties, election campaigns, government departments, politicians'selected charities, global organisations; give bribes.<br>- Make threats: threaten policy-making bodies at all levels with loss of corporate investment, corporate relocation or job and revenue losses.<br>- Use the 'revolving door' and create conflicts of interest: secure membership of or control policy-making bodies, working/technical/ advisory groups and committees, public-private economic and policy forums, national trade and conference delegations; conversely, recruit ex-officials/politicians to industry positions.<br>- Seek regulatory capture: a form of corporate incursion into government with corporate representatives and their interests playing a central role in regulatory body/ government decision-making.<br>- Access standard-setting fora: use corporate consultants (scientific/health and safety) to access and enter into dialogue with standard-setting bodies.
	A-P2. Attempt to influence policy processes and outcomes	- Gather intelligence: collect information on policies and politicians to inform lobbying.<br>- Deliver policy: offer dialogue and expertise or to take responsibility for designing and drafting policy.<br>- Lobby the executive: seek relationships and direct contact with relevant policy-makers to shape policy processes and secure industry-favourable outcomes.<br>- Lobby the legislature: use legislators to influence the introduction, advancement or modification of legislation and to deliver corporate-friendly testimonies.<br>- Lobby through internal leverage: seek to influence and oppose health departments/regulators via other departments or committees such as for business, trade, agriculture.<br>- Use written consultation submissions: imply authoritativeness and consensus, misreport research, overwhelm the process by large volume of submissions.<br>- Use administrative barriers to undermine adopted policy: seek to have implementation assigned to hostile/apathetic agencies or divert policy-mandated funds to other/less effective tasks and purposes.<br>- Engage in non-compliance: counter policy with promotions and discounts, fail to implement policy, use arguments/falsehoods and product availability to encourage non-compliance by businesses and the public.
	A-P3. Manage policy venues	- Use venue shifting: ensure legislation occurs at regulatory jurisdictions more favourable to industry.<br>- Use pre-emption at local, national and global levels: use the authority of higher-jurisdiction bodies to constrain public health policy-making at lower jurisdictions.
Use the law to obstruct policies	A-L1. Use legal challenges to policy pre- and post-adoption	- Threaten/take legal action: make (often false) claims of illegality under domestic laws or international trade and investment agreements and use countries to bring dispute cases to WTO against other countries' policies.<br>- Create 'regulatory chill': use (pending) legal threats and action in other countries as precedents to deter or shape new policies.
	A-L2. Use the law to undermine policy-making/public health community	- Interfere with institutions: attempt to remove powers from regulatory body; lobby judges/lawyers to influence proceedings.<br>- Obstruct public health campaigners: threaten or use legal action or injunctions to stop health advocates' campaigns.
Manufacture public support for corporate positions	A-S1. Coordinate and manage industry strategies	- Conduct professionally managed campaigns: engage consultants and legal, public relations and market research companies to manage strategies and to amplify corporate messages.
	A-S2. Form business alliances	- Joining forces with directly affected businesses: coordinate strategies and share resources with other manufacturers, supply chain businesses, trade associations, employee organisations.<br>- Secure support of indirectly affected businesses: recruit as allies other industries, eg, hospitality, packaging, printing, advertising, media (both large companies and SMEs) and other sectors, eg, farming, sports and chambers of commerce.
	A-S3. Secure support beyond business	- Form alliances with key individuals and organisations: buy and engineer support of influential individuals/experts, civil society organisations and foreign governments through payments/donations/help, false statements about policy and claims to represent disparate interests.
	A-S4. Fabricate allies	- Create front groups and others: set up front groups, astroturf,a SAPROs and others (eg, think-tanks) to use as campaign tools.
	A-S5. Operate through third parties	- Use allies (A-S2, A-S3, A-S4) to enact industry campaigns: direct all forms of allies to engage in campaign activities including recruiting other allies, producing and disseminating information, media advocacy, responding to policy consultations, lobbying, initiating legal action and agitating on behalf of corporations.

		- Create impression of independence: use varying degrees of concealment and opaqueness to hide links between allies (S2, S3, S4) and corporations to render their messages, actions and evidence more credible and acceptable.
	A-S6. Maximise corporate – favourable media content	- Access media through financial ties and relationships: direct ownership, board membership, funding, relationships with and training and payments to journalists.<br>- Access media by providing content: advertising, advertorials, paid for content, press releases, arguing principle of 'balanced' reporting.
Shape evidence to manufacture doubt	A-E1. Undermine and marginalise unfavourable research/information	- Produce pseudo-scientific critique: criticise (independent) research unfavourable to corporations using unachievable evidentiary standards and non-rigorous methods.<br>- Misrepresent evidence: misreport, 'cherry-pick' and misinterpret research and information unfavourable to corporations.<br>- Marginalise unfavourable evidence: reduce visibility and representation of (independent) research and information in the body of evidence by ignoring it or blocking publication.<br>- Hide evidence: hide unfavourable evidence produced or funded by corporations.<br>- Misrepresent scientific norms: over-emphasize complexity, uncertainty and disagreement among researchers.
	A-E2. Produce or sponsor favourable research/ information	- Create parallel scientific literatures: produce or commission external institutions and scientists to create a self-referential body of alternative research that contradicts the international research literature.<br>- Create information materials: produce diverse materials targeting a variety of audiences to promote evidence favourable to corporations.<br>- Promote falsehoods: present false or inaccurate information in materials produced and in public discourse.
	A-E3. Amplify and blend corporate-favourable evidence into public record and discourse	- Promote favourable evidence: widely disseminate favourable research and information using a variety of media and input from business and civil society allies.<br>- (Self)-reference: cite corporate-supporting and corporate-produced studies widely in (peer-reviewed) journal articles and other information sources.<br>- Engage in questionable citation practices: cite inaccessible, unpublished, unverifiable or non-peer-reviewed evidence.<br>- Participate at scientific events: use legitimate scientific platforms to showcase corporate-sponsored research, using independent scientists.
Displace and usurp public health	PH1. Undermine the rationale for statutory policies on corporate practices	- Seek policy substitution: initiate self-regulation and voluntary codes relating to, for example, labelling, 'conscious' advertising, 'responsible' marketing, reformulation, etc, to prevent binding regulation.
	PH2. Deliver individual-level Interventions	- Normalise less effective interventions: use SAPROs, CSR and partnerships with NGOs, professional organisations, governments and global organisations to contribute to funding, planning, delivery and evaluation of life-style information and education interventions that also emphasize responsible consumption.<br>- Divert attention to secondary issues: deliver interventions that do not impact on the sale of products (eg, in obesity, interventions designed to encourage exercise instead of changing consumption of food).
	PH3. Promote 'harm reduction' as public health goal	- Develop 'reduced harm' products: narrow the focus of interventions and market 'healthier' versions of products (eg, Diet Coke, e-cigarettes or low-alcohol drink), nutraceuticals, etc, as substitutes.
	PH4. Deliver education and training to public health professionals	- Provide public education: produce educational materials, books, guidelines and organise workshops for health professionals in partnership with civil society, patient and professional organisations, government departments and global organisations.
	PH5. Weaken the public health community	- Fragment the public health community: create divisions ('extremists' and 'moderates'), distract or overwhelm the public health community.<br>- Monitor and intimidate opponents: infiltrate/monitor public health advocacy groups and independent researchers, threaten to withdraw support, intimidate individuals.
Manage reputations to corporate advantage	R1. Repair and nurture corporate reputations	- Highlight CSR and good deeds: conduct CSR and philanthropy, providing funds and sponsorship to a variety of causes including (public) health organisations.<br>- Substitute for weak government: use operational scale and resources to deliver welfare and other services to populations.<br>- Seek respectability by association: publicly associate with respected individuals and organisations.
	R2. Discredit public health community	- Attack and defame: defame public health researchers, advocates and organisations through attacks on their work and personal integrity.

Abbreviations: SME, small and medium enterprises; SAPROs, social aspect public relations organisations; CSR, corporate social responsibility; NGOs, non-governmental organisations; WTO, World Trade Organization.

[a] Astroturf: fake grassroots organisations.

Estratégias de ação

Estratégias de ação	Mecanismos	Exemplos práticos
Acesso e influência na formulação de políticas	A-P1. Aceder a decisores políticos e a espaços de decisão	- Conceder incentivos: fornecer financiamento, recursos, hospitalidade e ofertas a políticos, funcionários públicos, partidos políticos, campanhas eleitorais, departamentos governamentais, instituições de caridade selecionadas por políticos ou organizações globais; oferecer subornos.<br>- Fazer ameaças: ameaçar organismos decisores com perda de investimento corporativo, deslocalização de empresas ou perdas de emprego e receitas.<br>- Utilizar a "porta giratória" e criar conflitos de interesses: garantir a participação ou controlo de órgãos decisores, grupos de trabalho/técnicos/consultivos, fóruns público-privados económicos e políticos, delegações nacionais em feiras e conferências; ou, inversamente, recrutar ex-responsáveis políticos ou ex-funcionários públicos para cargos na indústria.<br>- Procurar captura regulatória: forma de incursão corporativa no Estado em que representantes da indústria e os seus interesses desempenham um papel central nos organismos reguladores ou nas decisões governamentais.<br>- Aceder a fóruns de definição de normas: recorrer a consultores corporativos (científicos, de saúde e segurança) para aceder e dialogar com entidades responsáveis pela definição de normas.
	A-P2. Tentar influenciar processos e resultados políticos	- Recolher informação estratégica: recolher informação sobre políticas e decisores políticos para orientar atividades de lobbying.<br>- Oferecer elaboração de políticas: disponibilizar diálogo, "expertise" ou assumir a responsabilidade pela conceção e redação de políticas.<br>- Fazer lobbying junto do executivo: procurar relações e contactos diretos com decisores relevantes para moldar processos políticos e assegurar resultados favoráveis à indústria.<br>- Fazer lobbying junto do legislativo: utilizar deputados para influenciar a introdução, progressão ou modificação de legislação e apresentar testemunhos favoráveis à indústria.<br>- Fazer lobbying por via de alavancagem interna: tentar influenciar ou contornar departamentos de saúde ou entidades reguladoras através de outros ministérios ou comissões (por exemplo, economia, comércio, agricultura).<br>- Utilizar contributos escritos em consultas públicas: sugerir autoridade e consenso, deturpar investigação, ou sobrecarregar o processo com um elevado volume de submissões.<br>- Utilizar barreiras administrativas para minar políticas adotadas: procurar que a implementação seja atribuída a entidades hostis ou apáticas, ou desviar fundos legalmente previstos para outras tarefas menos eficazes.<br>- Envolver-se em incumprimento: contrariar políticas através de promoções e descontos, falhar na implementação, ou usar argumentos falsos e estratégias de disponibilidade de produtos para incentivar o incumprimento por empresas e pelo público.
	A-P3. Gerir espaços políticos	- Alteração de espaços decisórios: garantir que a legislação ocorre em jurisdições regulatórias mais favoráveis à indústria.<br>- Pré-empção a nível local, nacional e global: utilizar a autoridade de entidades de nível superior para restringir a formulação de políticas de saúde pública em níveis inferiores.
Utilizar a lei para obstruir políticas	A-L1. Utilizar ações legais antes e após a adoção de políticas	- Ameaçar ou intentar ações judiciais: alegar (frequentemente de forma infundada) ilegalidades ao abrigo do direito nacional ou de acordos internacionais de comércio e investimento, incluindo o recurso a Estados para instaurar litígios na OMC contra políticas de outros países.<br>- Criar "regulatory chill": usar ameaças legais pendentes ou ações judiciais noutros países como precedentes para dissuadir ou moldar novas políticas.
	A-L2. Utilizar a lei para minar decisores políticos e a comunidade de saúde pública	- Interferir com instituições: tentar retirar poderes a entidades reguladoras; exercer influência sobre juízes ou advogados para condicionar processos.<br>- Obstruir defensores da saúde pública: ameaçar ou utilizar ações judiciais ou providências cautelares para travar campanhas de defesa da saúde.
Fabricar apoio público a posições corporativas	A-S1. Coordenar e gerir estratégias da indústria	- Conduzir campanhas profissionalmente geridas: contratar consultores e empresas jurídicas, de relações públicas e de estudos de mercado para gerir estratégias e amplificar mensagens corporativas.
	A-S2. Formar alianças empresariais	- Unir-se a empresas diretamente afetadas: coordenar estratégias e partilhar recursos com outros fabricantes, empresas da cadeia de abastecimento, associações empresariais e organizações de trabalhadores.<br>- Garantir apoio de empresas indiretamente afetadas: recrutar como aliadas outras indústrias (por exemplo, hotelaria, embalagens, impressão, publicidade, media), bem como setores como agricultura, desporto e câmaras de comércio.
	A-S3. Garantir apoio fora do setor empresarial	- Formar alianças com indivíduos e organizações influentes: comprar ou engenheirar apoio de indivíduos influentes, peritos, organizações da sociedade civil e governos estrangeiros através de pagamentos, doações ou apoio, declarações falsas sobre políticas ou alegações de representação de interesses diversos.

	A-S4. Fabricar aliados	- Criar organizações-fachada: criar grupos de fachada, *astroturfing*, SAPROs e outras entidades (por exemplo, think tanks) como instrumentos de campanha.
	A-S5. Operar através de terceiros	- Utilizar aliados (A-S2, A-S3, A-S4) para executar campanhas: direcionar aliados para recrutar outros aliados, produzir e disseminar informação, fazer advocacy mediática, responder a consultas públicas, fazer lobbying, iniciar ações legais e mobilizar apoio em nome das empresas.<br>- Criar aparência de independência: utilizar diferentes graus de ocultação e opacidade para esconder ligações entre aliados e empresas, tornando mensagens, ações e "evidência" mais credíveis e aceitáveis.
	A-S6. Maximizar cobertura mediática favorável à empresa	- Aceder aos media através de relações financeiras: propriedade direta, participação em conselhos, financiamento, relações com jornalistas, formação e pagamentos.<br>- Aceder aos media fornecendo conteúdos: publicidade, publirreportagens, conteúdos pagos, comunicados de imprensa, invocando o princípio do "equilíbrio" noticioso.
Moldar a evidência para fabricar dúvida	A-E1. Minar e marginalizar investigação ou informação desfavorável	- Produzir críticas pseudo-científicas: criticar investigação independente desfavorável utilizando padrões probatórios inatingíveis e métodos pouco rigorosos.<br>- Deturpar evidência: selecionar seletivamente ("cherry-picking"), interpretar incorretamente ou deturpar investigação desfavorável.<br>- Marginalizar evidência desfavorável: reduzir a visibilidade ou representação de investigação independente ignorando-a ou bloqueando a sua publicação.<br>- Ocultar evidência: esconder resultados desfavoráveis produzidos ou financiados pela indústria.<br>- Deturpar normas científicas: exagerar complexidade, incerteza e desacordo entre investigadores.
	A-E2. Produzir ou financiar investigação ou informação favoráve	- Criar literaturas científicas paralelas: produzir ou encomendar a instituições e cientistas externos um corpo de investigação autorreferencial que contradiz a literatura internacional.<br>- Criar materiais informativos: produzir materiais dirigidos a diferentes públicos para promover evidência favorável à indústria.<br>- Promover falsidades: apresentar informação falsa ou imprecisa em materiais produzidos e no discurso público.
	A-E3. Amplificar e integrar evidência favorável à empresa no registo público e no discurso	- Promover evidência favorável: disseminar amplamente investigação e informação favoráveis através de vários meios e com apoio de aliados empresariais e da sociedade civil.<br>- (Auto)-referenciação: citar amplamente estudos produzidos ou financiados pela indústria em artigos científicos e outras fontes.<br>- Práticas de citação questionáveis: citar evidência inacessível, não publicada, não verificável ou não sujeita a revisão por pares.<br>- Participar em eventos científicos: utilizar plataformas científicas legítimas para divulgar investigação patrocinada pela indústria, recorrendo a cientistas independentes.
Displace and usurp public Health Substituir e usurpar funções da saúde pública	PH1. Minar a justificação de políticas estatutárias sobre práticas corporativas	- Procurar substituição de políticas: iniciar autorregulação e códigos voluntários (por exemplo, rotulagem, publicidade "consciente", marketing "responsável", reformulação) para evitar regulação vinculativa.
	PH2. Implementar intervenções ao nível individual	- Normalizar intervenções menos eficazes: utilizar SAPROs, CSR e parcerias com ONG, organizações profissionais, governos e organizações globais para financiar, planear, executar e avaliar intervenções educativas que enfatizam o consumo responsável.<br>- Desviar a atenção para questões secundárias: implementar intervenções que não afetam as vendas (por exemplo, promover exercício físico em vez de alterar o consumo alimentar).
	PH3. Promover a "redução de danos" como objetivo de saúde pública	- Desenvolver produtos de "menor risco": comercializar versões "mais saudáveis" de produtos (por exemplo, bebidas dietéticas, produtos com menos açúcar, etc.) como substitutos.
	PH4. Fornecer educação e formação a profissionais de saúde pública	- Fornecer educação pública: produzir materiais educativos, livros, orientações e organizar workshops para profissionais de saúde em parceria com organizações da sociedade civil, entidades profissionais, departamentos governamentais e organizações globais.
	PH5. Enfraquecer a comunidade de saúde pública	- Fragmentar a comunidade: criar divisões entre "extremistas" e "moderados", distrair ou sobrecarregar a comunidade de saúde pública.<br>- Monitorizar e intimidar opositores: infiltrar-se ou monitorizar grupos de advocacia e investigadores independentes, ameaçar retirar apoio ou intimidar indivíduos.

Gerir reputações para vantagem corporativa	R1. Reparar e fortalecer reputações corporativas	- Destacar CSR e boas práticas: desenvolver ações de responsabilidade social e filantropia, financiando e patrocinando diversas causas, incluindo organizações de saúde pública.<br>- Substituir o Estado quando este é fraco: utilizar escala operacional e recursos para prestar serviços sociais ou de bem-estar.<br>- Procurar respeitabilidade por associação: associar-se publicamente a indivíduos e organizações respeitadas.
	R2. Desacreditar a comunidade de saúde pública	- Atacar e difamar: desacreditar investigadores, defensores e organizações de saúde pública através de ataques ao seu trabalho e integridade pessoal.

Abreviações: PME – pequenas e médias empresas; SAPROs – organizações de relações públicas focadas em "aspetos sociais"; CSR – responsabilidade social corporativa; ONG – organizações não-governamentais; OMC – Organização Mundial do Comércio.
[a] *Astroturfing:* criação de falsas organizações de base *("grassroots")*.
